# Catalytic β C–H amination *via* an imidate radical relay†
†Electronic supplementary information (ESI) available. See DOI: 10.1039/c8sc05685d


**DOI:** 10.1039/c8sc05685d

**Published:** 2019-01-17

**Authors:** Leah M. Stateman, Ethan A. Wappes, Kohki M. Nakafuku, Kara M. Edwards, David A. Nagib

**Affiliations:** a The Ohio State University , Department of Chemistry and Biochemistry , Columbus , OH 43210 , USA . Email: nagib.1@osu.edu

## Abstract

The first catalytic strategy to harness imidate radicals for C–H functionalization has been developed. This iodine-catalyzed approach enables β C–H amination of alcohols by an imidate-mediated radical relay. In contrast to our first-generation, (super)stoichiometric protocol, this catalytic method enables faster and more efficient reactivity. Furthermore, lower oxidant concentration affords broader functional group tolerance, including alkenes, alkynes, alcohols, carbonyls, and heteroarenes. Mechanistic experiments interrogating the electronic nature of the key 1,5 H-atom transfer event are included, as well as probes for chemo-, regio-, and stereo-selectivity.

## Abstract 2

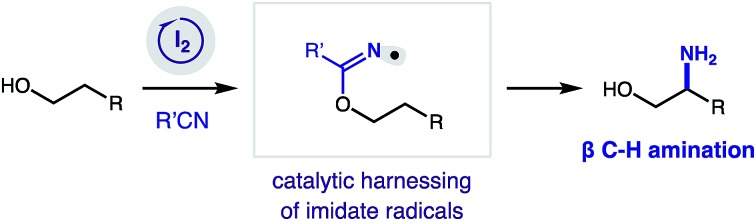
An iodine-catalyzed strategy for β C–H amination of alcohols is enabled by a chemo-, regio-, and stereo-selective H-atom transfer mechanism.

## Introduction

**Fig. 1:**
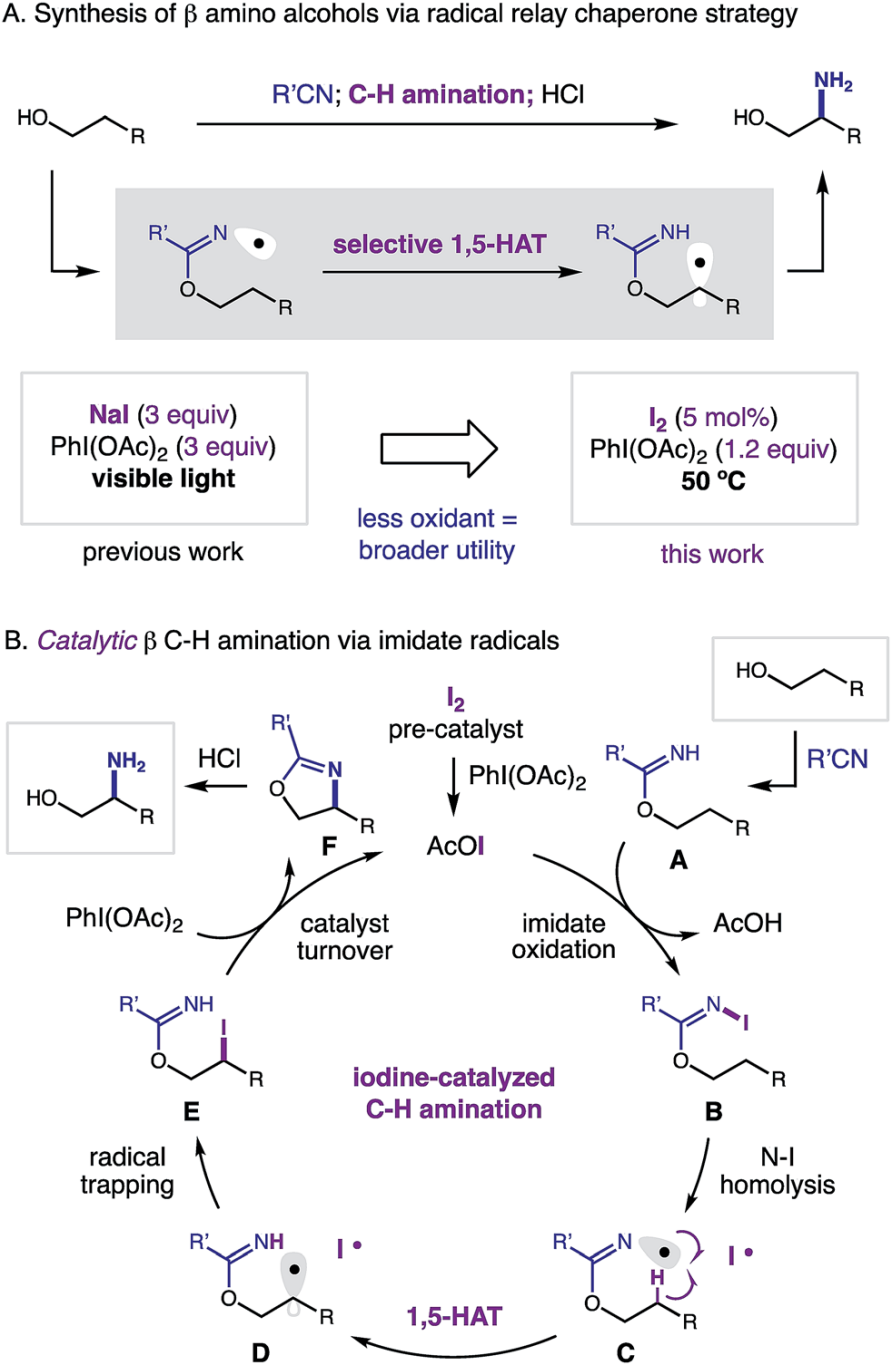
Radical relay strategy for β C–H amination of alcohols: (a) catalytic *vs* stoichiometric. (b) Iodine-catalyzed mechanism.

At the frontier of organic synthesis, the selective replacement of an unbiased C–H bond with a more valuable chemical motif remains a vital challenge.^1^ Specifically, incorporation of a nitrogen atom by C–H amination is an especially important goal in medicinal chemistry.^2^,^3^ Among recent advances toward directed sp^3^ C–H functionalization of abundant alcohol derivatives,^4^ there remain few methods to synthesize β amino alcohols (a privileged motif in medicine)^5^ by C–H amination.^6^ To complement state-of-the-art, metal-catalyzed nitrenoid and C–H insertion pathways for remote C–H amination,^7^ we sought to employ a radical-based approach that entails δ selective, hydrogen atom transfer (HAT).^8^,^9^ Despite recent advances in δ C–H amination *via* HAT,^10^ there remain few catalytic examples of this transformation.^11^ Having recently disclosed the first method for directed β C–H amination of alcohols by a complementary imidate radical relay,^12^,^13^ we sought to develop an improved, catalytic strategy (Fig. 1).

In our radical relay chaperone strategy, alcohols are readily converted to imidates by addition to nitriles (Fig. 1a). Upon combination with stoichiometric oxidant (NaI, PhI(OAc)_2_; 3 equiv. each), a transient sp^2^ N-centered radical^14^,^15^ is generated that undergoes selective 1,5-HAT to afford a C-centered radical – β to the imidate. Subsequent radical trapping and acidic hydrolysis yields β amino alcohols in a rapid, selective, and efficient fashion. With the hopes of expanding the synthetic utility of this new strategy, we proposed development of a catalytic variant (Fig. 1b). To complement our first-generation, photo-mediated method, we hypothesized the key, radical-generating iodine atom, which is not incorporated in the product, could be continually recycled and ultimately employed in a catalytic fashion.

In an alternate, thermally initiated sequence, we envisioned a substoichiometric quantity of I_2_ may undergo ligand substitution with PhI(OAc)_2_ (1 equiv. only) to generate AcOI. In the presence of an alcohol-derived imidate (A), selective formation of a weak N–I bond (B)^16^,^17^ would enable thermal homolysis to an N-centered radical (C). This transient species (typically accessed by photolysis)^18^ should undergo regio-selective 1,5-HAT to yield β radical (D). Rapid radical recombination (or chain-propagation with AcOI or N–I) would yield β alkyl iodide (E). In the presence of PhI(OAc)_2_ as a terminal oxidant, we proposed oxazoline (F) formation may accompany regeneration of the AcOI catalyst by one of two mechanisms: (1) iodide displacement by the imidate, and re-oxidation of I^–^ to I^+^,^19^ or (2) alkyl hypervalent iodane formation, and amination *via* an I(iii)/I(i) pathway.^20^ Importantly, we proposed thermal initiation of this catalytic cycle under low concentrations of I_2_ (or AcOI) may improve reaction efficiency and chemoselectivity by precluding byproduct-forming pathways associated with photolysis of these promiscuous oxidants.^21^

## Results and discussion

In accord with our design, we were pleased to find the *catalytic* β C–H amination of imidate **1** by HAT is indeed possible with 5 mol% I_2_ and 1.2 equiv. PhI(OAc)_2_, affording **2** in 95% yield (Table 1). Crucially, this thermal protocol requires polar, aprotic solvents (*e.g.* DMF, MeCN), whereas other solvents (*e.g.* CH_2_Cl_2_, PhMe) afford inferior yields (entries 1–4). Although rigorous degassing is not essential, an N_2_ atmosphere was found to be superior to an aerobic one (entry 5). Although alkali iodide salts (*e.g.* NaI, CsI) are competent sources of iodine for this reaction, they are less efficient than more soluble I_2_ reagent (entries 6 and 7). Finally, photolysis (entry 8) or non-photolytic initiation at room temperature (entry 9) afford reactivity, albeit with less efficiency than standard thermal initiation at 50 °C.

**Table 1:** Development of a catalytic C–H amination of imidates

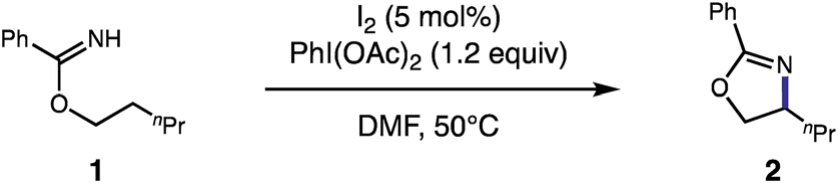		
Entry	Changes from standard conditions	Yield (%)
1	None	95
2	CH_2_Cl_2_ instead of DMF	31
3	PhMe instead of DMF	33
4	MeCN instead of DMF	94
5	Air atmosphere instead of N_2_	61
6	NaI instead of I_2_	67
7	CsI instead of I_2_	76
8	2 × 23 W CFL	50
9	Dark, room temperature	40

**Fig. 2:**
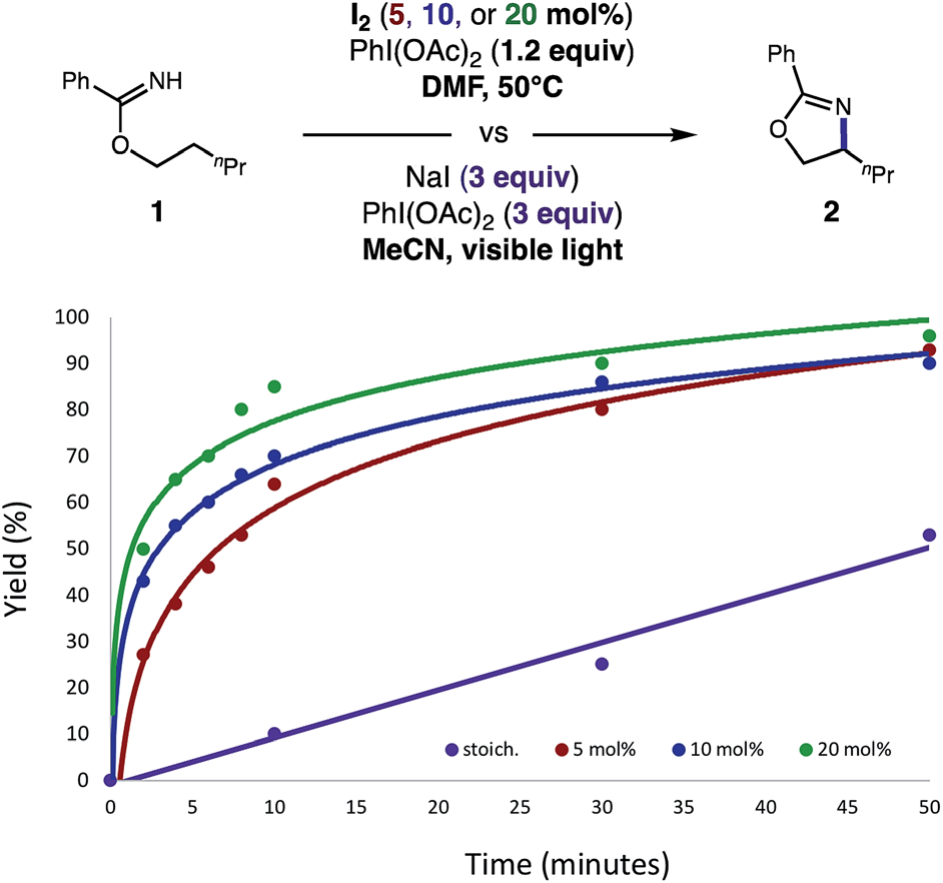
Comparison of the catalytic C–H amination of imidates with the previous stoichiometric version.

Interestingly, we noted this catalytic reaction is completed at a significantly faster rate than the first-generation, photo-initiated conditions. As shown in Fig. 2, our previous conditions, which are super-stoichiometric in NaI and PhI(OAc)_2_, required several hours for reaction completion (purple line). Conversely, 80% yield is observed in 30 minutes with 5–10% I_2_ (red and blue lines) or even in as little as 10 minutes with 20% I_2_ (green line). Although a mere 1% I_2_ provides full conversion in 6 hours, we found these longer reaction times to be less practical than the 1–2 hours needed for 5% catalyst loading. Given that less soluble sources of iodide (*e.g.* NaI, CsI) do not afford product as rapidly or efficiently (likely due to slower, incomplete generation of I_2_), we presume greater solubility of I_2_ affords a higher initial concentration of the active oxidant, AcOI. Taken together, these data suggest the faster rates shown in Fig. 2 are consistent with a higher initial concentration of reactive AcOI, as proposed in the mechanism shown in Fig. 1. Moreover, less terminal oxidant, and thermal (*vs.* photolytic) initiation, may be responsible for ensuring AcOI-based, two-electron reactivity is more selective for the desired reaction pathway.

### Synthetic scope

**Fig. 3:**
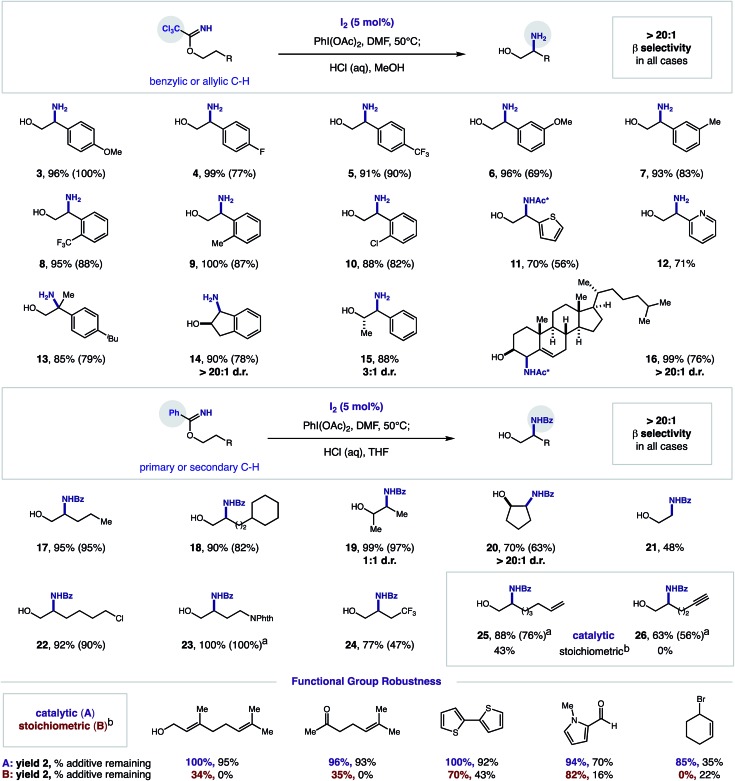
Synthetic utility of iodine-catalyzed β C–H amination of imidates. *Conditions:* 0.4 mmol imidate, I_2_ (5 mol%), PhI(OAc)_2_ (1.2 equiv.), DMF (0.2 M), 50 °C. ^1^H NMR yield of oxazoline determined *vs.* standard. Hydrolysis with HCl (2 M) affords amino alcohol (isolated yield, in parenthesis). ^a^Isolated yield of oxazoline. ^b^Stoichiometric NaI (ref. 12). Ac* refers to trichloroacetamide. *Functional group robustness:* C–H amination yield, % additive remaining.

In order to explore the synthetic utility of our new thermally initiated, I_2_-catalyzed protocol, we subjected a series of imidates to these β C–H amination conditions (5% I_2_, 1.2 equiv. PhI(OAc)_2_, DMF, 50 °C). Upon reaction completion, acidic hydrolysis of the resulting oxazoline with *aq.* HCl yielded the respective β amino alcohol. As shown in Fig. 3, a wide range of imidates undergo the radical relay mechanism *via* these catalytic conditions. For trichloroacetimidates (derived from combination of alcohols and Cl_3_C–CN), a range of electronically diverse 2-phenylethanol derivatives could be selectively aminated at the β position. These benzylic C–H aminations (**3–10**) are amenable to both electronically rich and deficient substituents (OMe, Me, F, CF_3_) as well as *ortho*, *meta*, and *para* substitution. Additionally, medicinally relevant heteroarenes (thiophene, pyridine, **11–12**) are tolerated, as well as the tertiary C–H of an ibuprofen analog (**13**). Secondary alcohols are efficiently aminated with excellent diastereoselectivity (up to >20 : 1 d.r.; **14–15**). Finally, the allylic C–H of a cholesterol analog is also efficiently and stereo-selectively aminated (**16**). To promote the β amination of stronger, aliphatic C–H bonds, we employed benzimidates (derived from combination of alcohols with Ph-CN or with Ph(CN)OCH_2_CF_3_). This catalytic protocol is also suitable for the regioselective amination of primary, secondary, and tertiary C–H bonds (**17–21**). Similarly, secondary alcohols are tolerated, although greater diastereoselectivity is observed for cyclic *versus* acyclic cases (>20 : 1 d.r. *vs.* 1 : 1 d.r.).

As a testament to the synthetic utility provided by these mild, catalytic conditions, several functional groups that were not previously tolerated in our stoichiometric protocol can now be aminated (**22–26**). Most interestingly, the reactive π-systems of alkenes and alkynes, which are prone to deleterious reaction with large amounts of oxidant, are now amenable as substrates. For example, whereas alkene **25** was previously accessible in only 43% yield under the stoichiometric, photochemical protocol, these catalytic, thermal conditions provide amination in 88% yield. Additionally, alkynes, which had previously not been tolerated (0%), are now suitable substrates for this β C–H amination (**26**, 63% yield).

To further probe this improved functional group tolerance, we conducted an additive robustness screen^22^ for both the stoichiometric and catalytic protocols – employing synthetically and medicinally relevant functionalities that appeared unlikely to withstand strong oxidative conditions. As shown in Fig. 3, this catalytic method is superior for the β C–H amination of imidate **1** to **2** in the presence of various functional groups, including alkenes, alcohols, ketones, aldehydes, thiophenes, pyrroles, and halides (catalytic: 85–100%; stoichiometric: 0–82% yield). Illustrating the mildness of the new catalytic protocol, these additives are recovered in up to 95% yield, whereas they are frequently decomposed in the highly oxidative environment of the stoichiometric conditions (0–43%) (see ESI† for complete table of functional groups tolerated, including those that are tolerated in both conditions).

### Mechanistic investigations

**Fig. 4:**
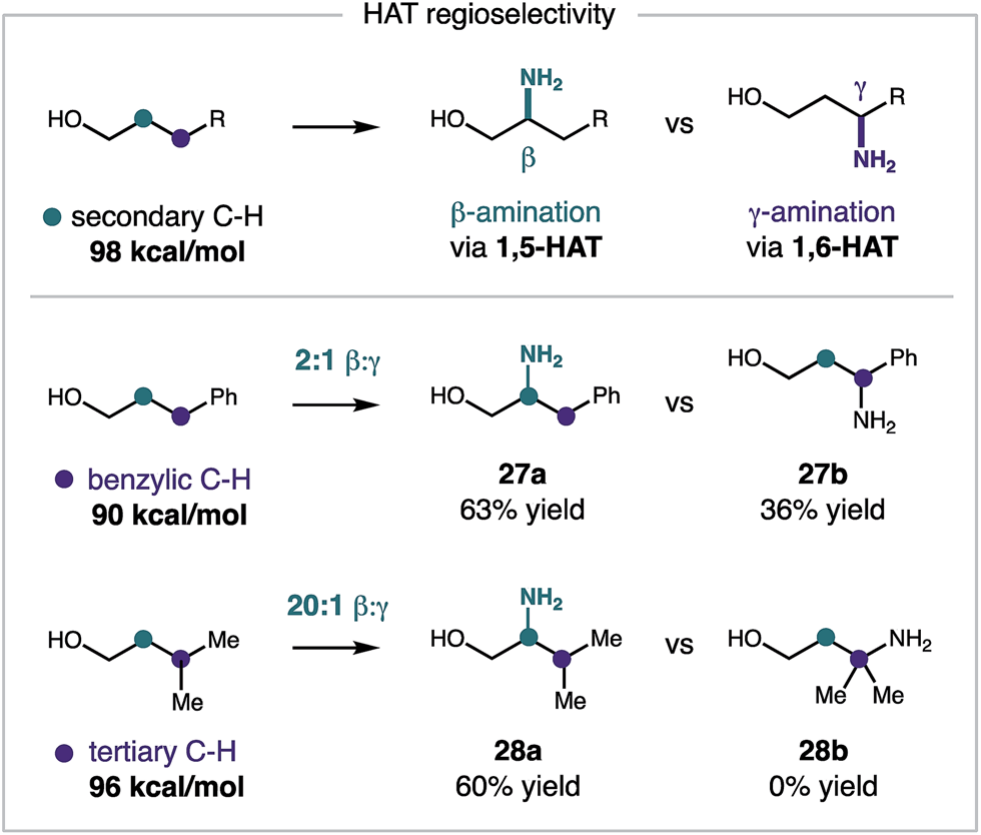
Regioselectivity probes of β selectivity *via* 1,5-HAT.

To gain a deeper understanding of this reaction mechanism, we conducted a series of competitive rate studies interrogating various stereoelectronic effects. First, we probed the regioselectivity of the imidate radical-mediated amination in the presence of weaker C–H bonds. Although our reaction design is based on the entropic and enthalpic favorability of 1,5-HAT,8*a* there are notable examples of 1,6-HAT mediated pathways that are governed by substrate geometry^23^ or thermodynamics.^24^ To test the influence of the latter, the β selectivity of this C–H amination was investigated for alcohols bearing a weaker γ C–H bond (Fig. 4). In each case, β selectivity (*via* 1,5-HAT) was observed in preference to γ selectivity (*via* 1,6-HAT). When the γ C–H bond is significantly weaker (benzylic: 90 *vs.* secondary: 98 kcal mol^–1^),^25^ the β amine **27** is still preferentially formed (2 : 1 β : γ selectivity). However, when γ C–H bond is only marginally weaker (3°: 96 *vs.* 2°: 98 kcal mol^–1^),^25^ the β amine **28** is obtained exclusively (>20 : 1 β : γ selectivity).

**Fig. 5:**
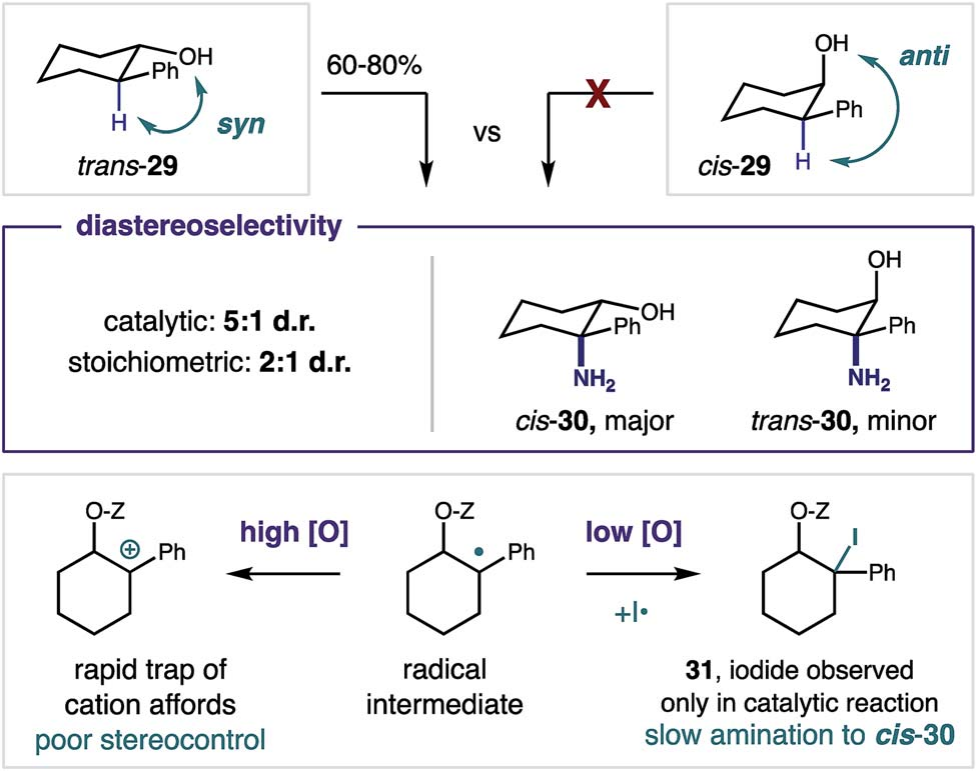
Diastereoselectivity *via* divergent trapping mechanisms.

Next, we investigated the observed diastereoselectivity of the β C–H amination by employing *cis* and *trans* isomers of 2-phenyl-cyclohexanol **29** as stereochemical probes in the formation of β amino alcohol **30** (Fig. 5). Whereas the trichloroacetimidate of *cis*-**29** does not afford C–H amination (likely because the imidate radical is conformationally constrained to the opposite side of the ring), *trans*-**29** efficiently undergoes HAT (since the imidate radical and β C–H are *syn* to one another). Interestingly, these catalytic conditions afford greater diastereoselectivity (5 : 1 d.r.) than the stoichiometric protocol (2 : 1 d.r.). Moreover, β benzyl iodide intermediate **31** was observed for the first time, only in the catalytic case. Taken together, these results suggest divergent mechanisms are operative in the radical trapping steps of these two protocols. A possible explanation is that the higher oxidant concentration of the (super)stoichiometric method more rapidly oxidizes the benzyl radical to a cation, which is unselectively cyclized to afford the thermodynamically favored *cis* product in only a 2 : 1 excess. On the other hand, a stepwise iodine trapping and subsequent cyclization mechanism under the low oxidant concentration of the catalytic conditions allow for greater 5 : 1 diastereoselectivity. This likely occurs *via* slower conversion of the observed alkyl iodide intermediate **31**, which enables greater, overall stereocontrol. Oxidation of benzyl iodide **31** to its hypervalent iodane nucleofuge may also afford cyclization – with either retention or inversion.^26^

**Fig. 6:**
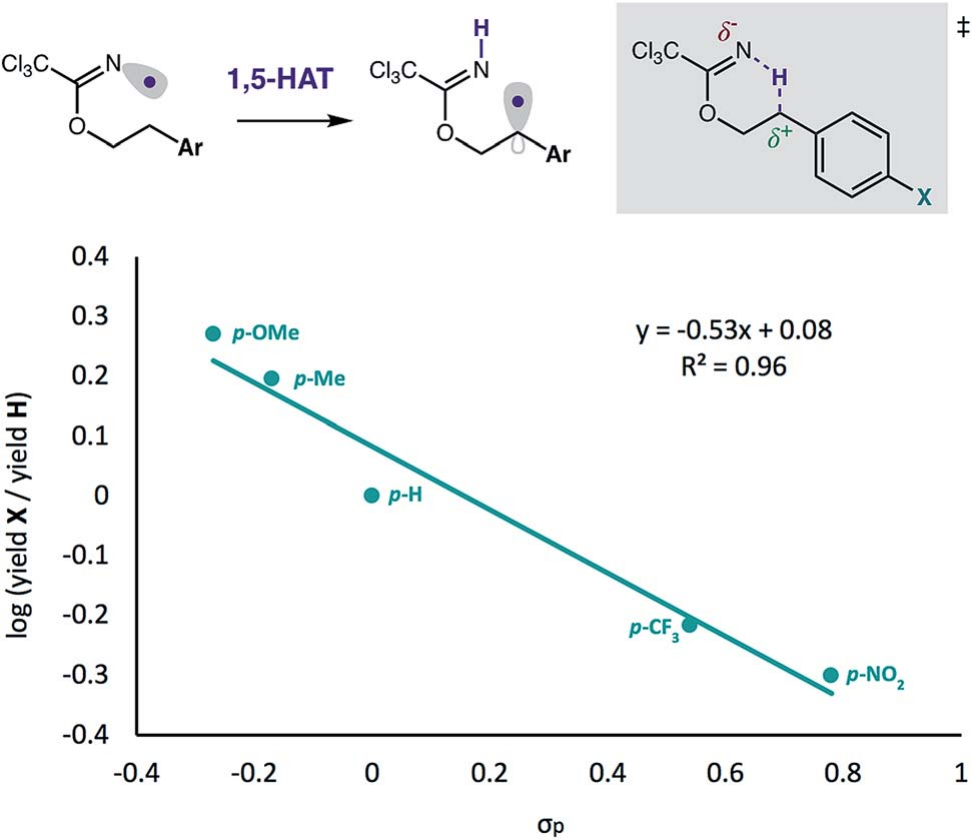
Hammett plot correlates to a cation-like transition state.

Finally, we examined the nature of the hydrogen atom transfer mechanism *via* a Hammett study (Fig. 6). By varying substituents of 2-arylethanol imidates, we determined a linear free-energy relationship exists between initial reaction rates and the electronics of the *para*-substituents. As shown in Fig. 6, we observed reaction acceleration with *p*-OMe and *p*-Me groups, whereas *p*-CF_3_ and *p*-NO_2_ substituents decrease product formation relative to the parent 2-Ph-ethanol. The resulting negative slope (*ρ*) of the Hammett equation is consistent with other HAT-mediated C–H functionalizations.^27^ In this case, we propose intramolecular HAT (which we have shown to be rate-limiting, with primary KIE values up to 8)^12^ is enabled by an electrophilic N-centered radical, gaining electron density in the transition state, as an N–H σ bond is formed. At the same time, the carbon atom loses electron density in the transition state as the ensuing C-centered radical is formed. Thus, electron-releasing groups at the *para*-position stabilize this transition state by electron donation, while electron-withdrawing groups have the opposite effect. The resultant stabilization by donating groups thus reasonably explain the observed reaction rate acceleration.

## Conclusions

In summary, we have developed the first catalytic variant of our radical chaperone strategy for converting alcohols into β amino alcohols *via* HAT. This conversion of ubiquitous motifs into privileged pharmacophores is a synthetically valuable method enabled by a radical relay cascade. Through a new, I_2_-catalyzed protocol, this β C–H amination sequence now has significantly broadened synthetic utility. We expect additional mechanistic insights provided herein (on reaction rates, as well as chemo-, regio- and stereo-selectivity) will enable further applications of the imidate-mediated HAT in regioselective C–H functionalizations.

## Conflicts of interest

There are no conflicts to declare.

## Supplementary Material

Supplementary information

Supplementary information

## References

[cit1] Yi H., Zhang G., Wang H., Huang Z., Wang J., Singh A. K., Lei A. (2017). Chem. Rev..

[cit2] Davies H. M. L., Manning J. R. (2008). Nature.

[cit3] Cernak T., Dykstra K. D., Tyagarajan S., Vachal P., Krska S. W. (2016). Chem. Soc. Rev..

[cit4] Breslow R., Winnik M. A. (1969). J. Am. Chem. Soc..

[cit5] Vitaku E., Smith D. T., Njardarson J. T. (2014). J. Med. Chem..

[cit6] Espino C. G., Wehn P. M., Chow J., Du Bois J. (2001). J. Am. Chem. Soc..

[cit7] Breslow R., Gellman S. H. (1983). J. Am. Chem. Soc..

[cit8] Stateman L. M., Nakafuku K. M., Nagib D. A. (2018). Synthesis.

[cit9] Betancor C., Concepción J. I., Hernández R., Salazar J. A., Suárez E. (1983). J. Org. Chem..

[cit10] Liu T., Mei T.-S., Yu J.-Q. (2015). J. Am. Chem. Soc..

[cit11] Qin Q., Yu S. (2015). Org. Lett..

[cit12] Wappes E. A., Nakafuku K. M., Nagib D. A. (2017). J. Am. Chem. Soc..

[cit13] Mou X. Q., Chen X. Y., Chen G., He G. (2018). Chem. Commun..

[cit14] Zard S. Z. (2008). Chem. Soc. Rev..

[cit15] Forrester A. R., Gill M., Thomson R. H. (1979). J. Chem. Soc., Perkin Trans. 1.

[cit16] Since AcOI is electrophilic at I, combination with an imidate likely forms N–I *vs.* N–OAc; Chen E. M. Keefer R. M. Andrews L. J., J. Am. Chem. Soc., 1967, 89 , 428 –430 .

[cit17] An N–OAc oxime imidate is also an unlikely intermediate since it is stable to thermal decomposition (up to 110 °C), and it does not afford product under these reaction conditions. See ESI for details

[cit18] Glover S. A. Hammond G. P. Harman D. G. Mills J. G. Rowbottom C. A., Aust. J. Chem., 1993, 46 , 1213 –1228 , . See also ref. 12 and 13*b*–*d* .

[cit19] (b) Uyanik M. Hayashi H. Ishihara K., Science, 2014, 345 , 291 –294 , . See also ref. 11*d* . 25035486 10.1126/science.1254976

[cit20] Yoshimura A. Zhdankin V. V., Chem. Rev., 2016, 116 , 3328 –3435 , . See also ref. 11*c* . 26861673 10.1021/acs.chemrev.5b00547

[cit21] Courtneidge J. L., Lusztyk J., Pagé D. (1994). Tetrahedron Lett..

[cit22] Collins K. D., Glorius F. (2013). Nat. Chem..

[cit23] Short M. A., Blackburn J. M., Roizen J. L. (2018). Angew. Chem., Int. Ed..

[cit24] Koag M., Lee S. (2011). Org. Lett..

[cit25] Luo Y. R., Comprehensive Handbook of Chemical Bond Energies, Taylor & Francis, Boca Raton, FL, 2010.

[cit26] Cambie R. C., Chambers D., Lindsay B. G., Rutledge P. S., Woodgate P. D. (1980). J. Chem. Soc., Perkin Trans. 1.

[cit27] Coniglio A., Galli C., Gentili P., Vadalà R. (2009). Org. Biomol. Chem..

