# Supplementary figures and images for: Catalytic β C–H amination via an imidate radical relay
Source: Chem Sci. 2019 Jan 17;10(9):2693–9. doi: 10.1039/c8sc05685d (PMC6419930; doi:10.1039/c8sc05685d)

# NMR Spectra

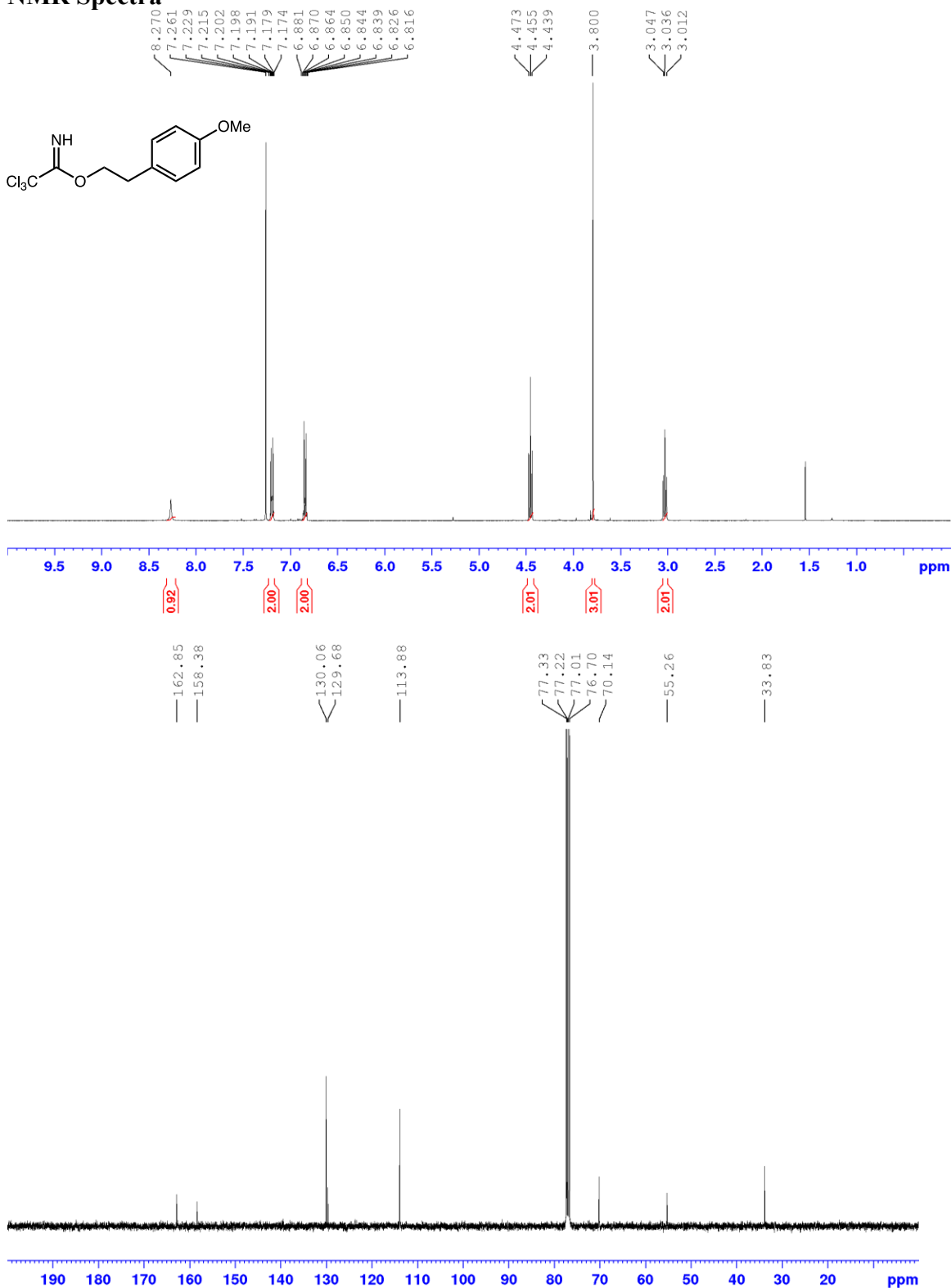

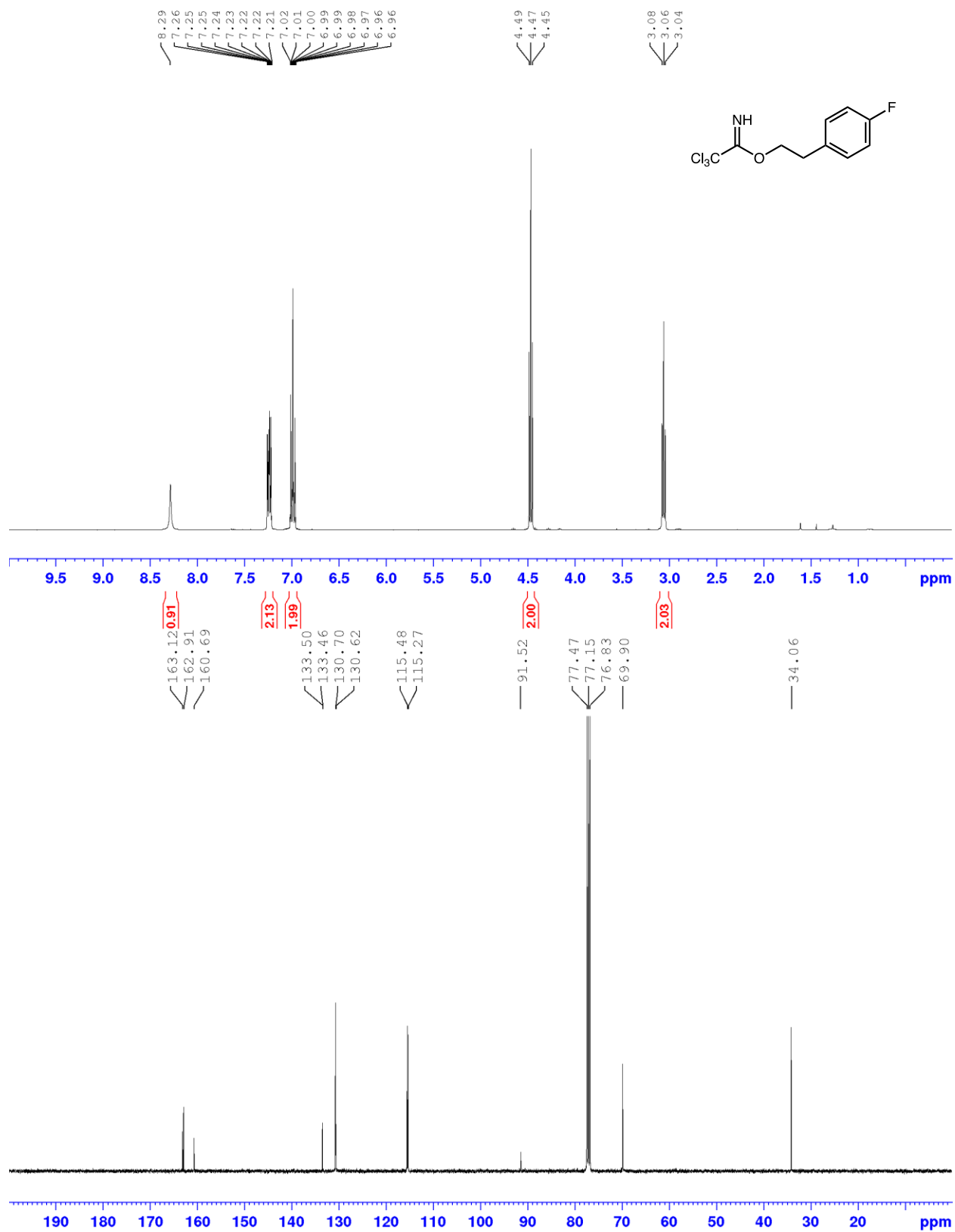

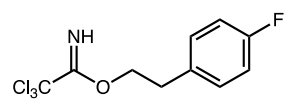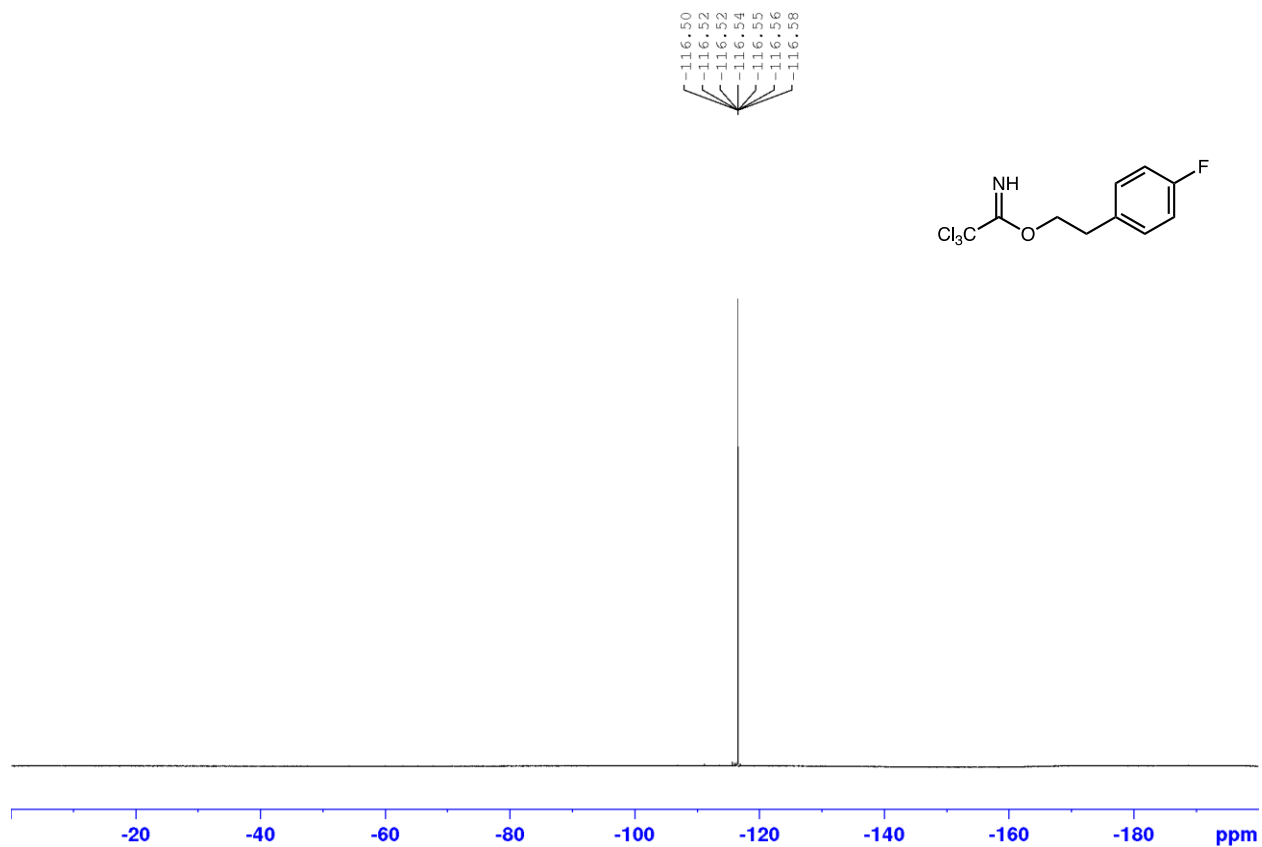

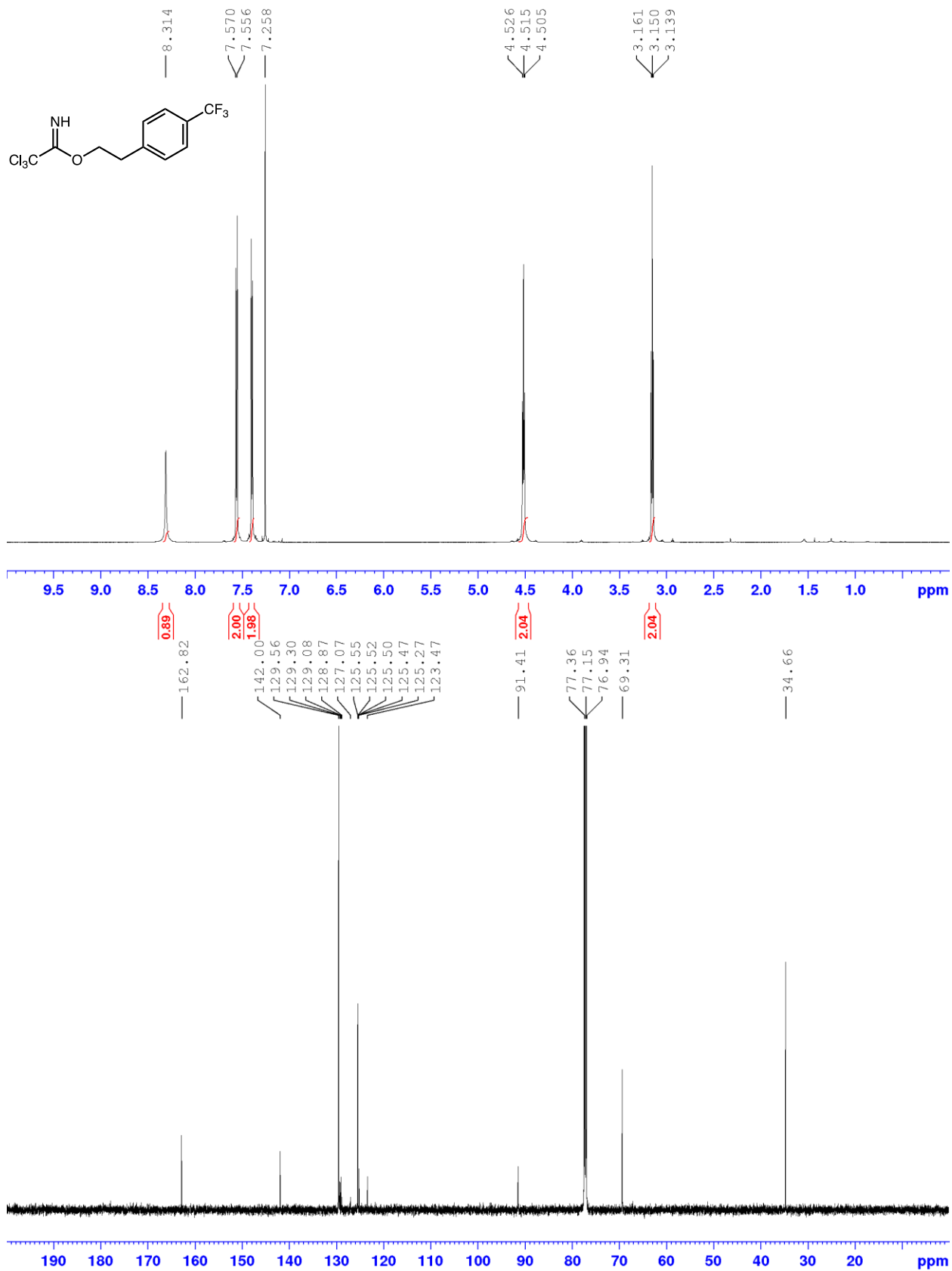

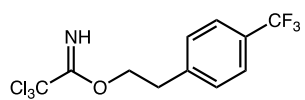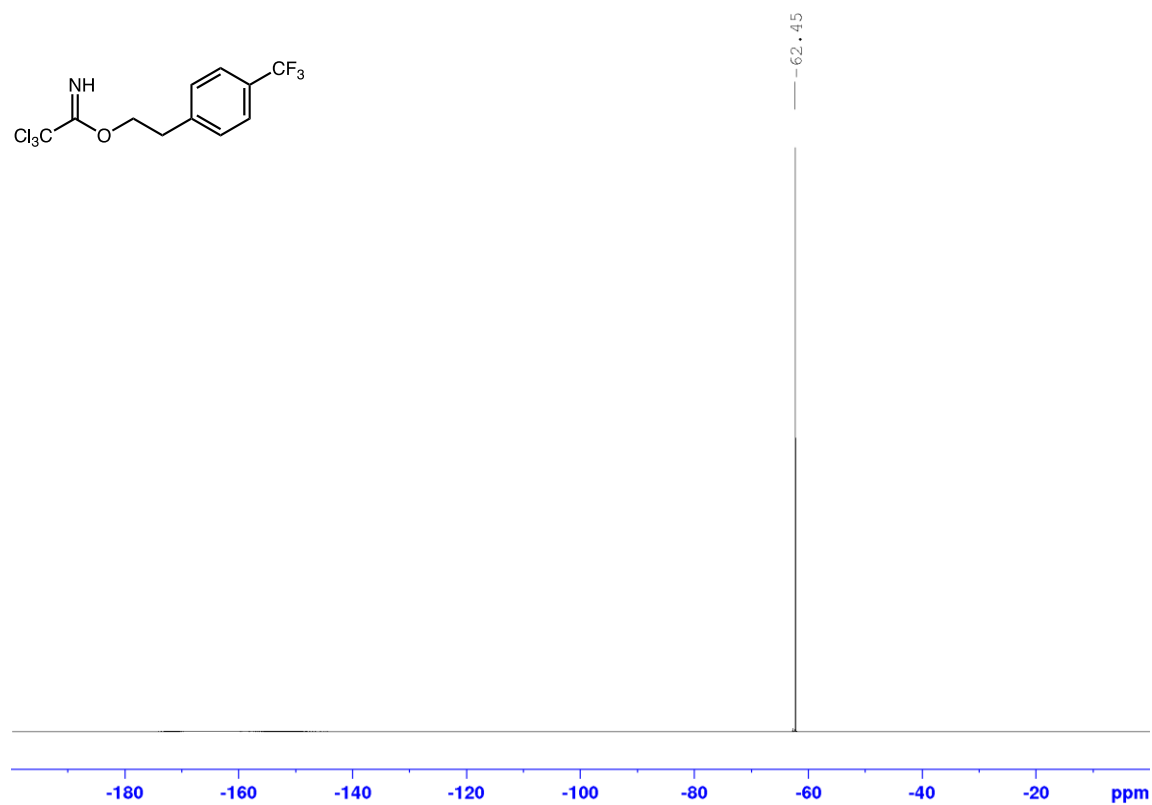

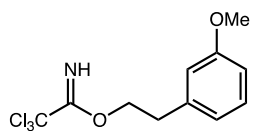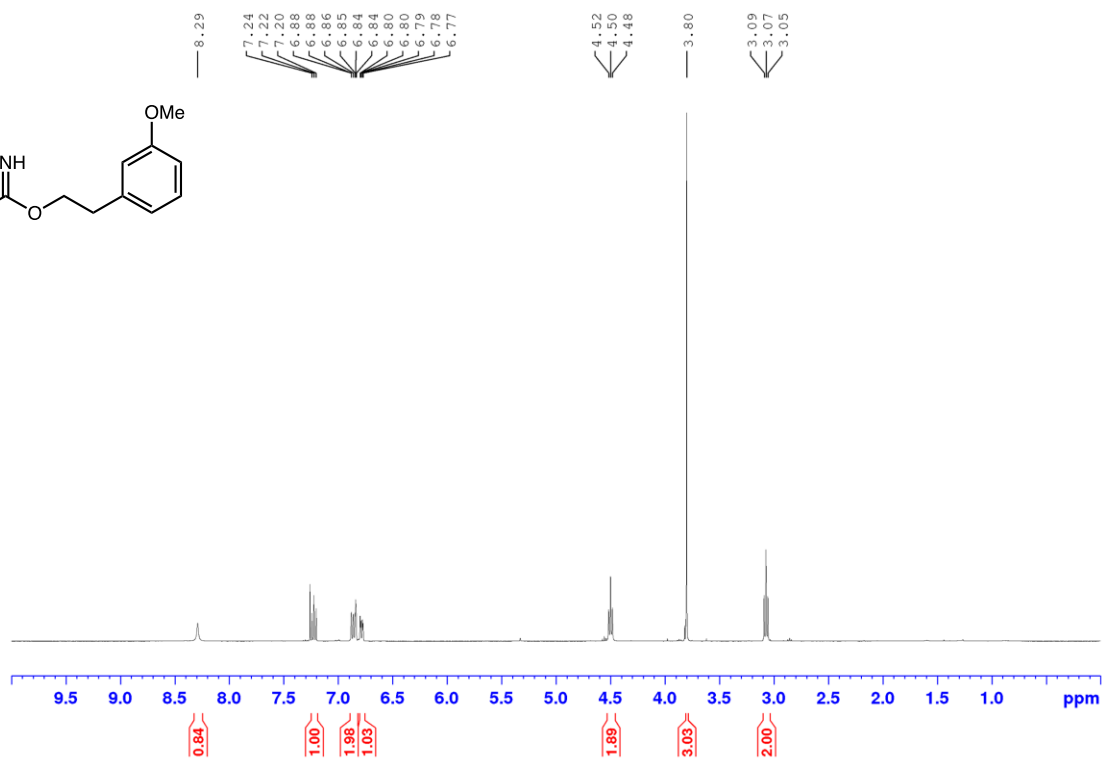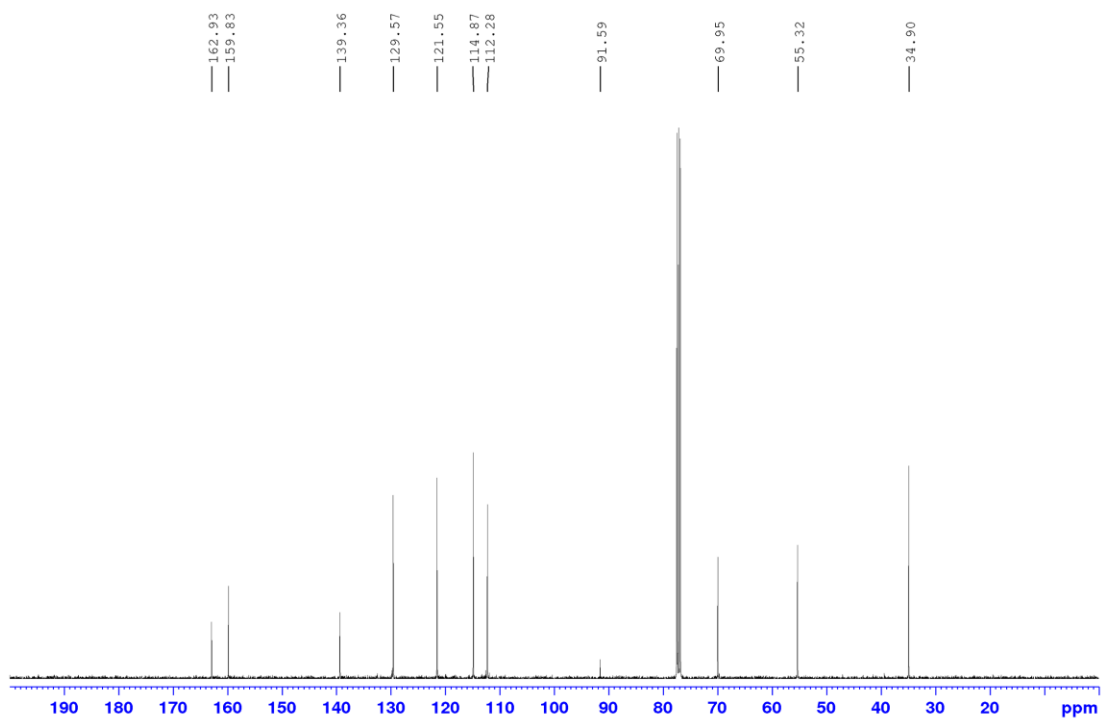

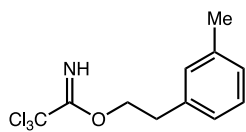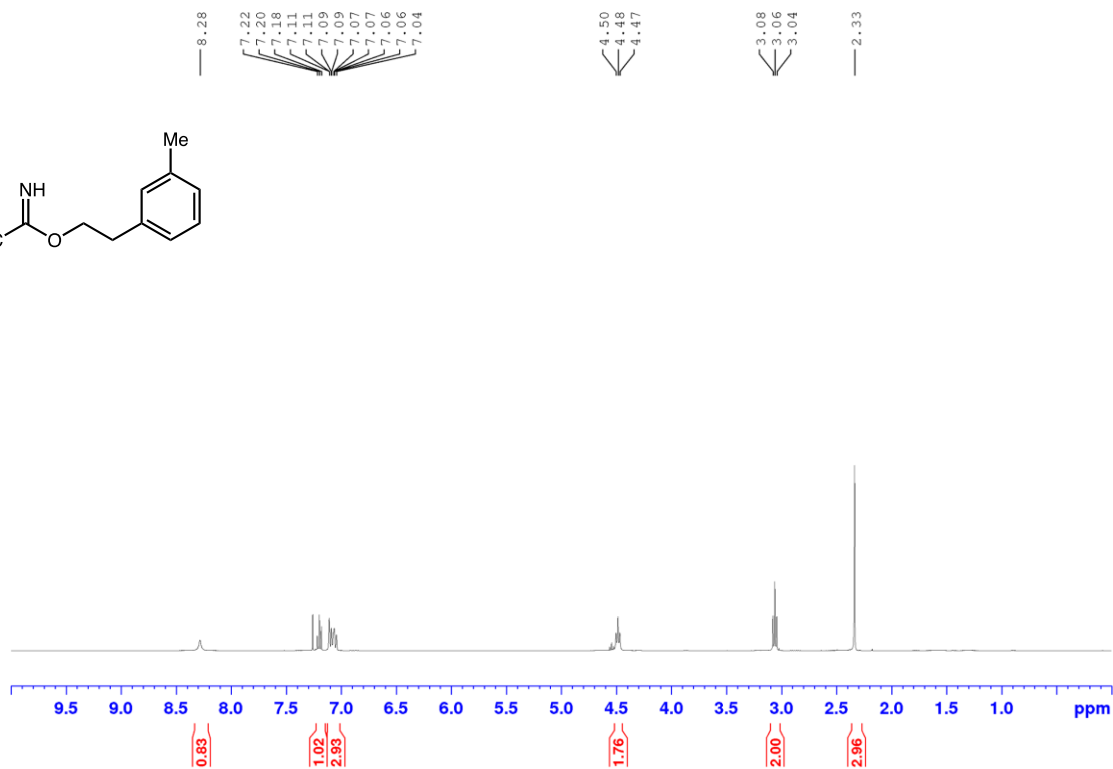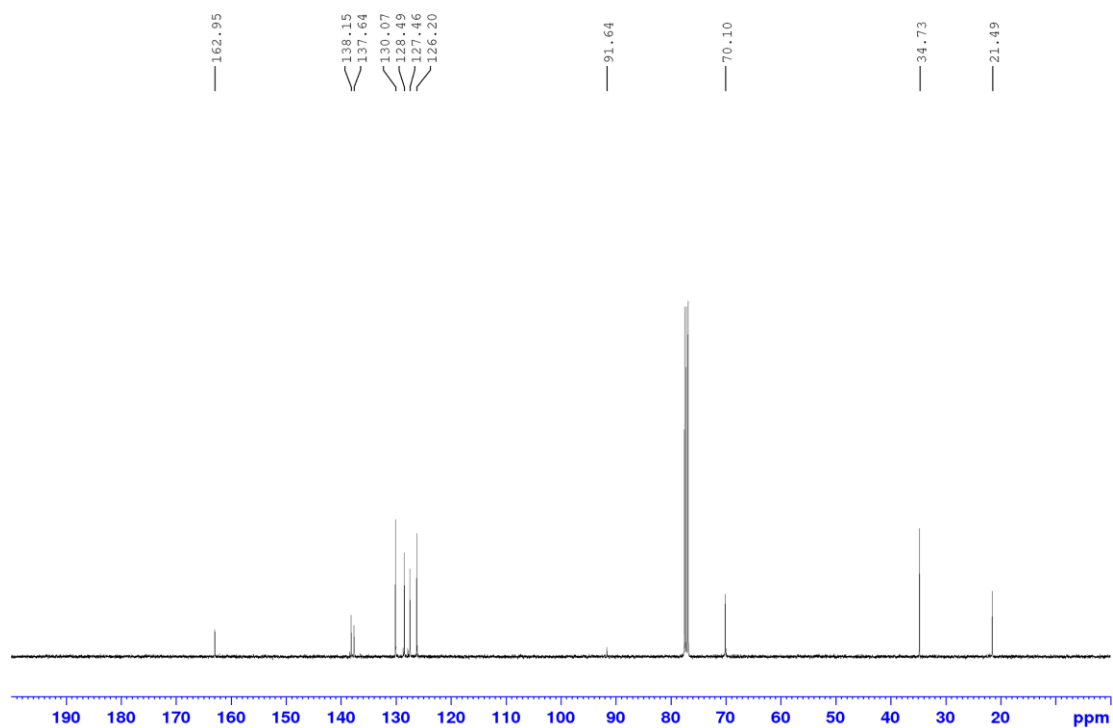

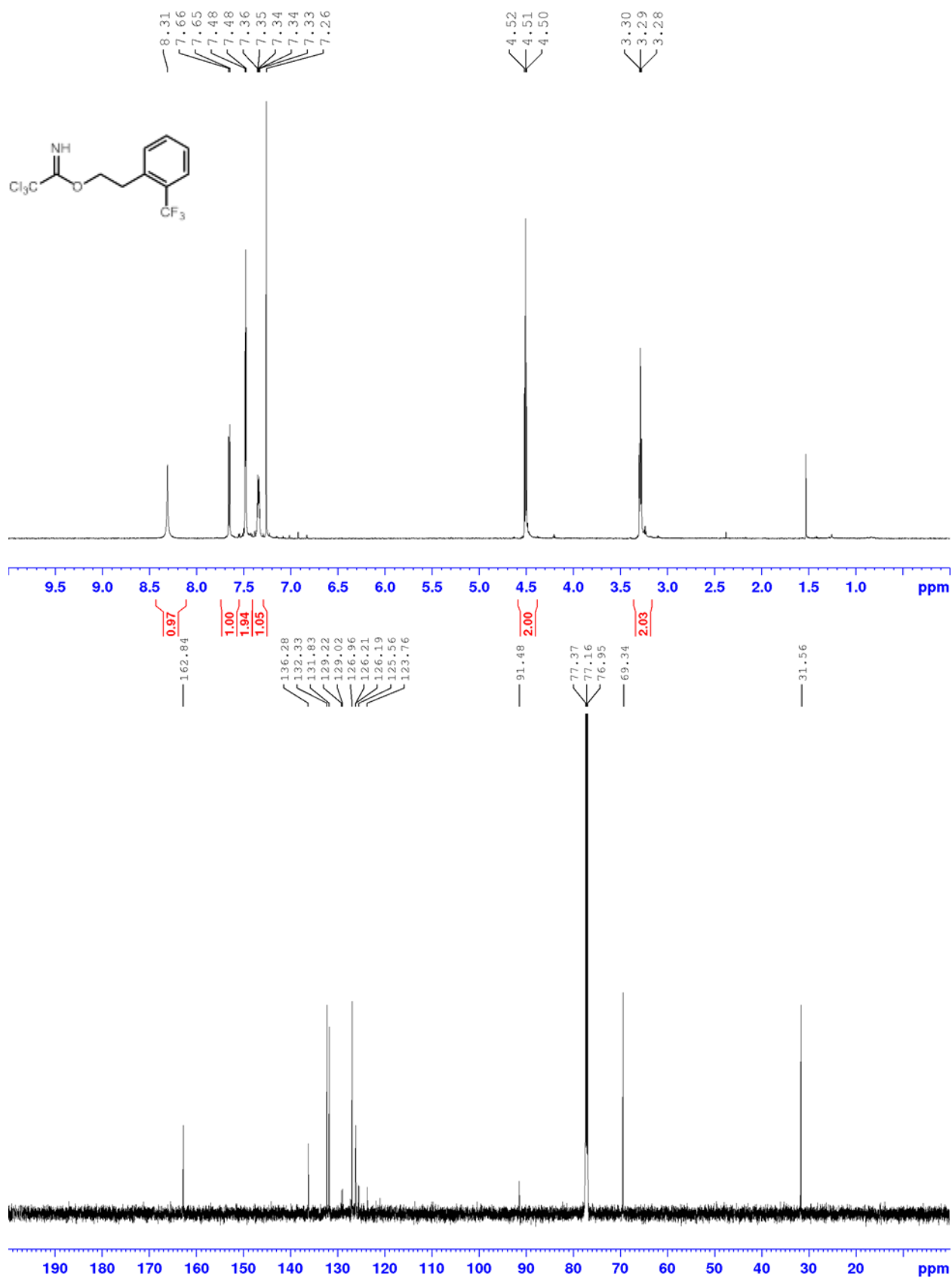

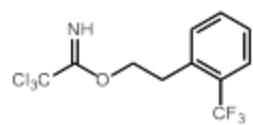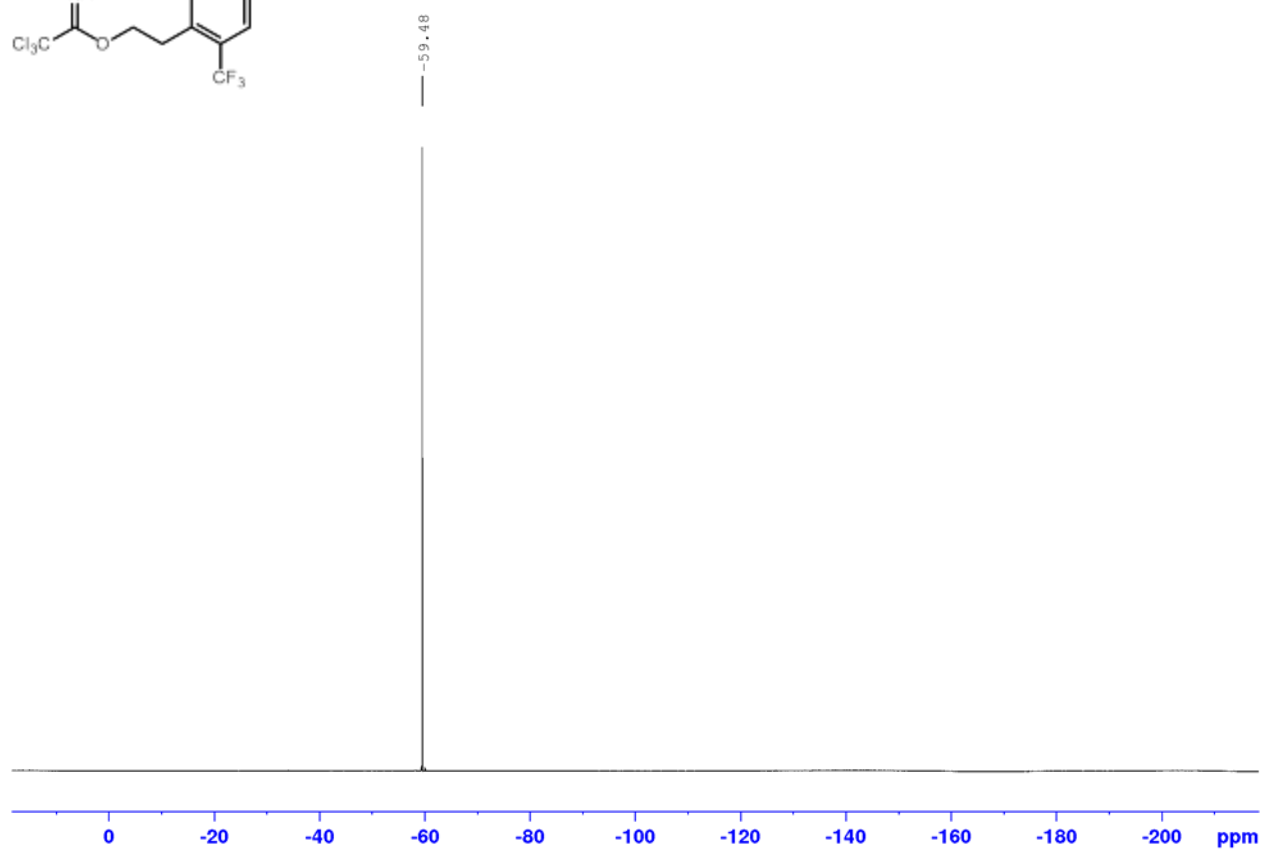

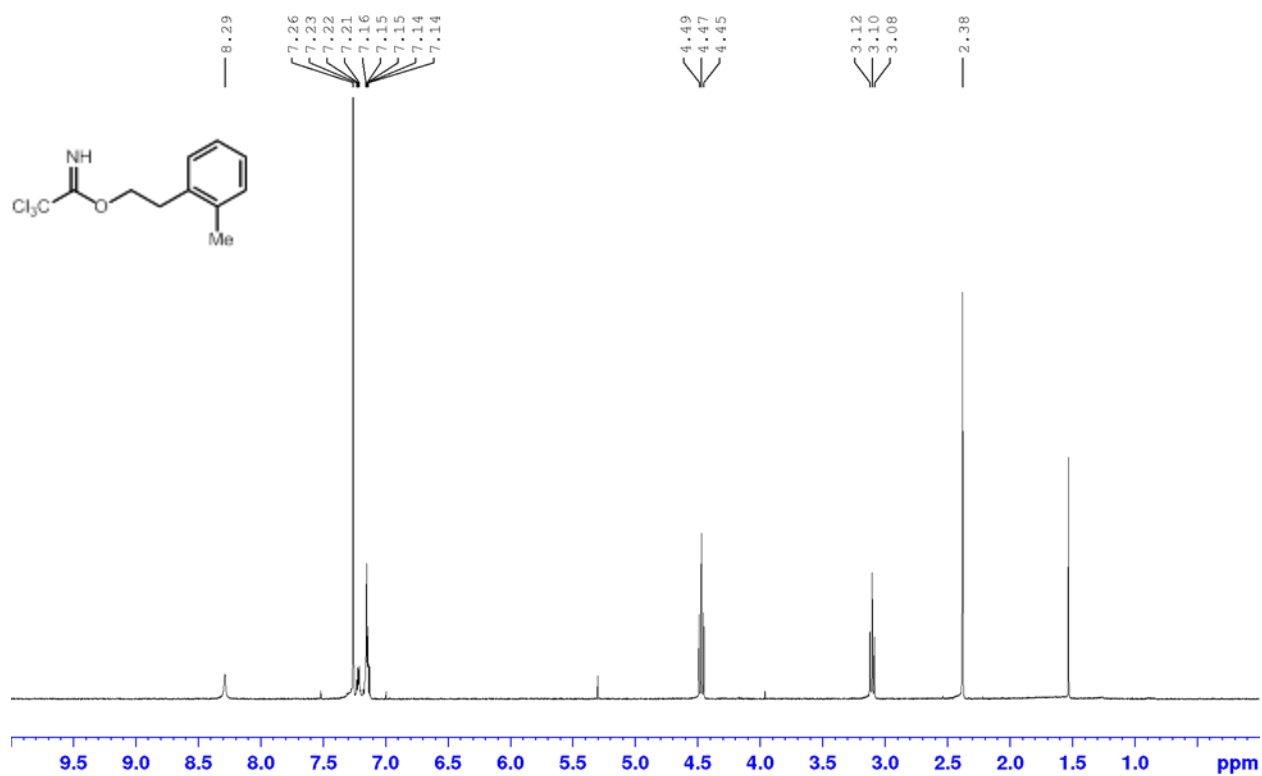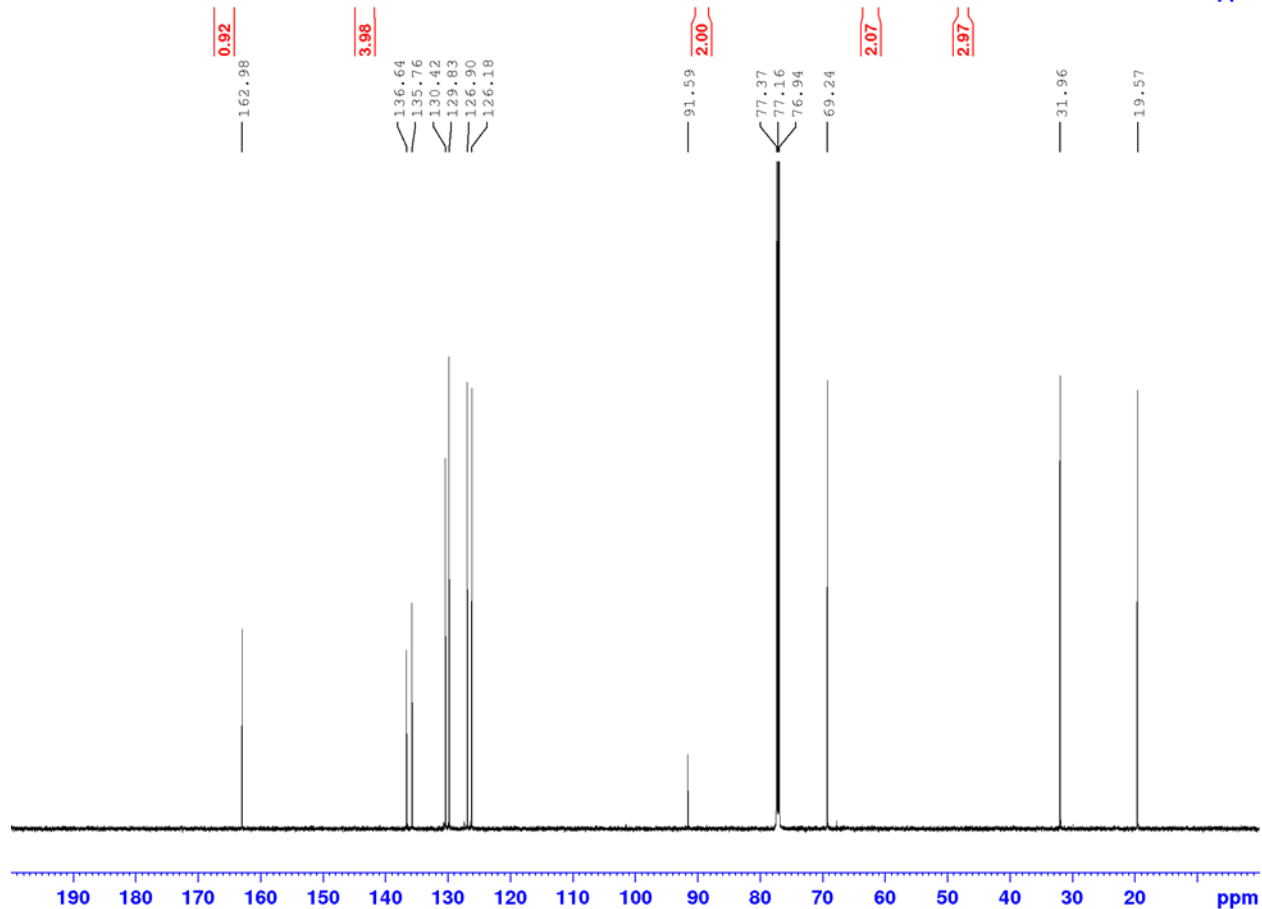

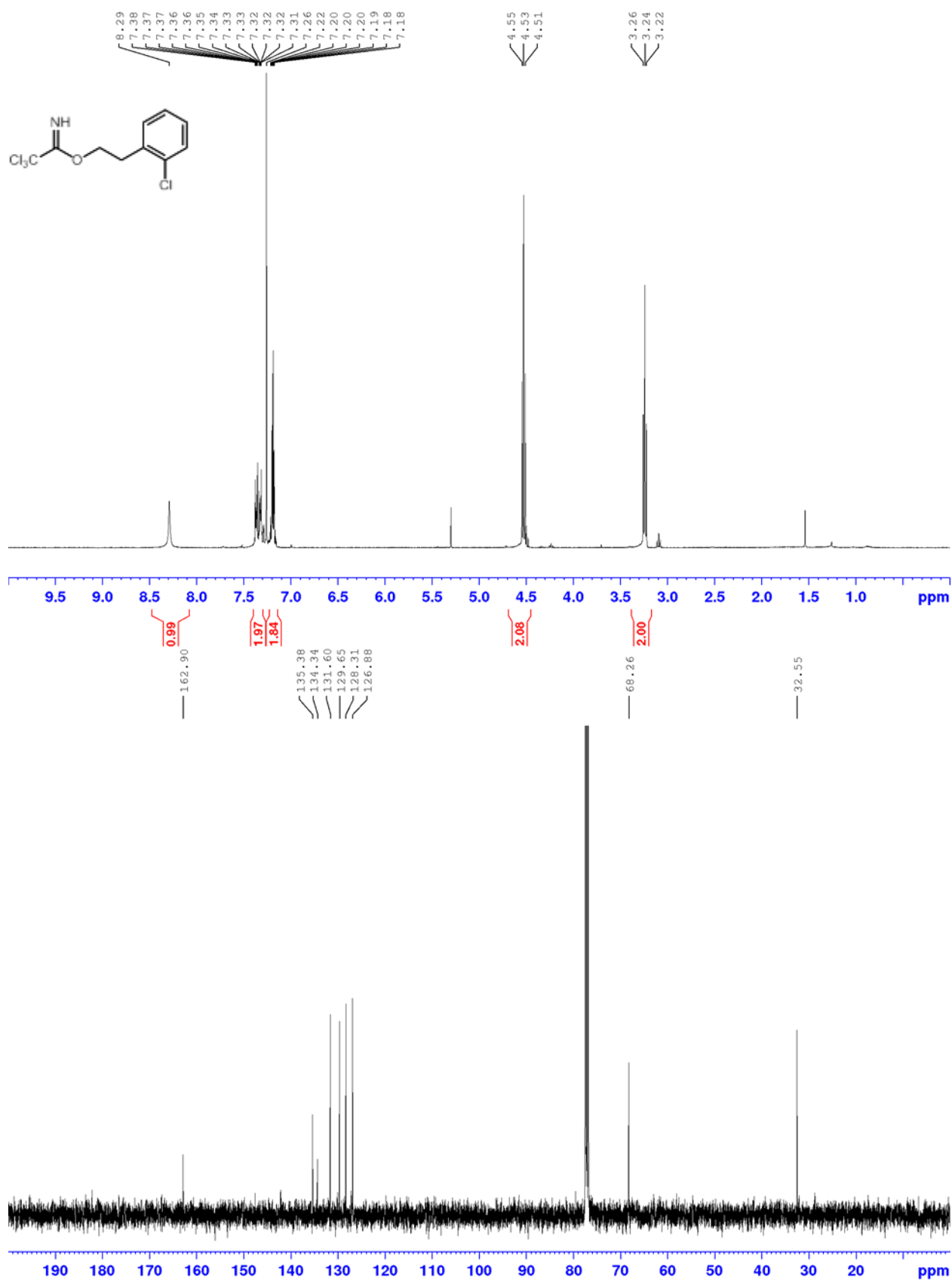

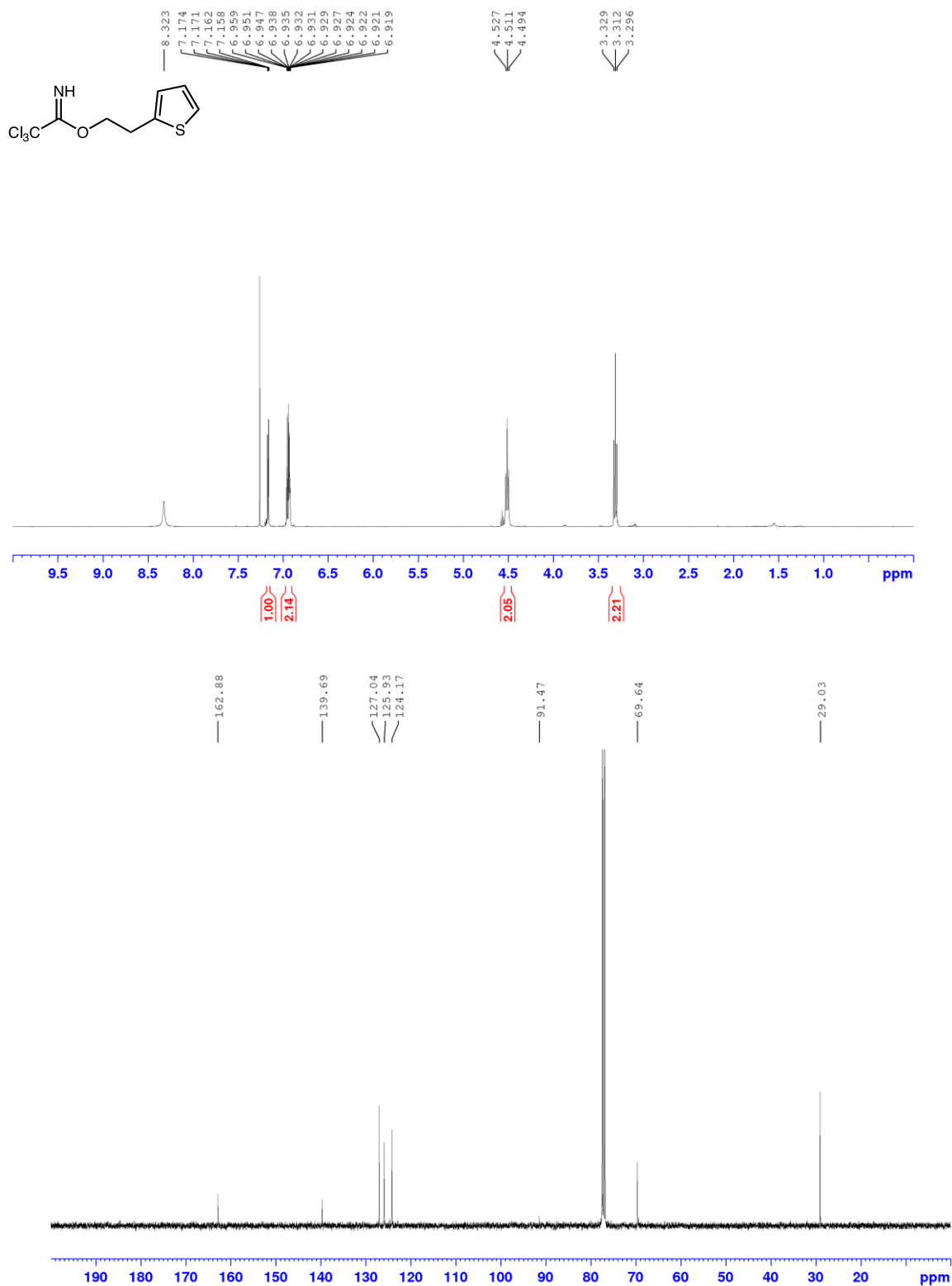

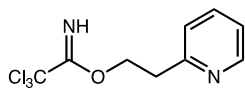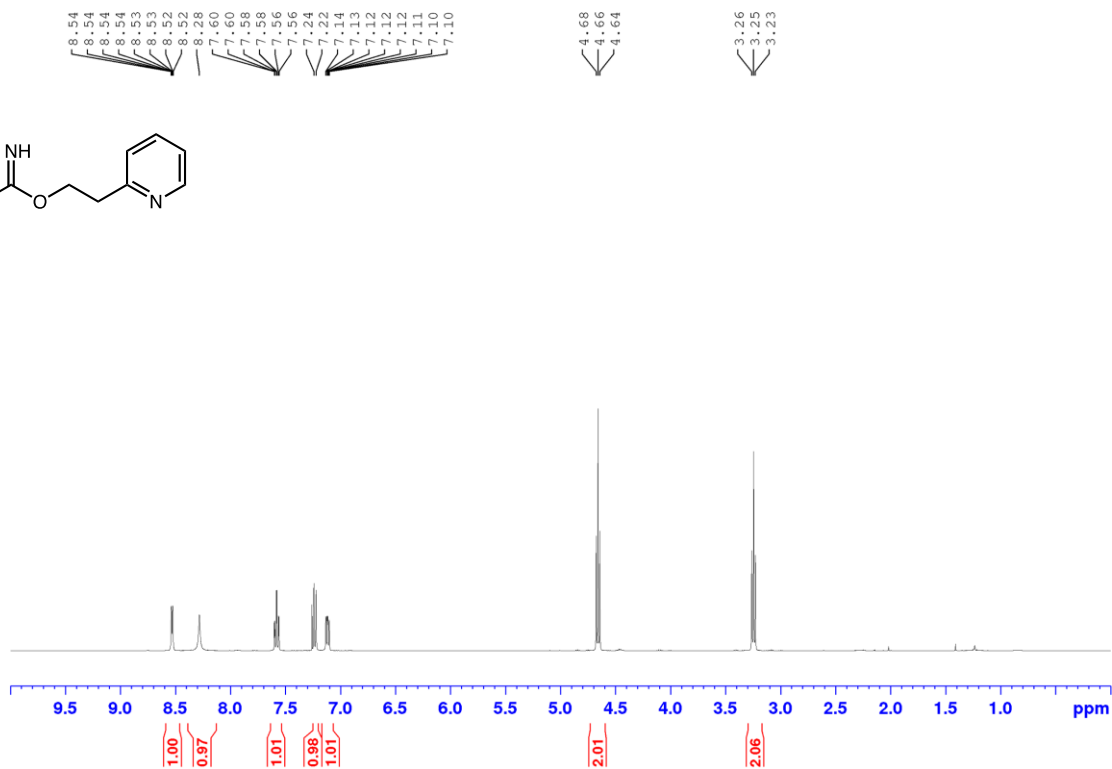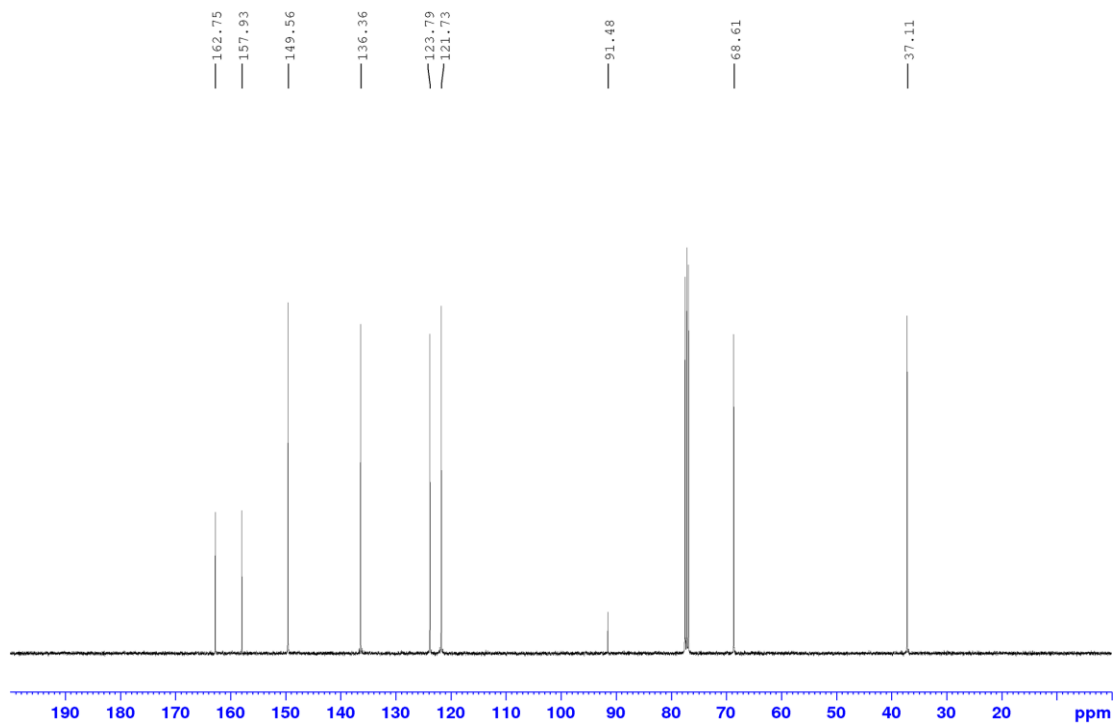

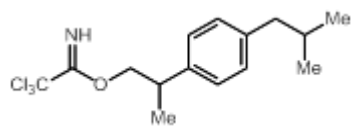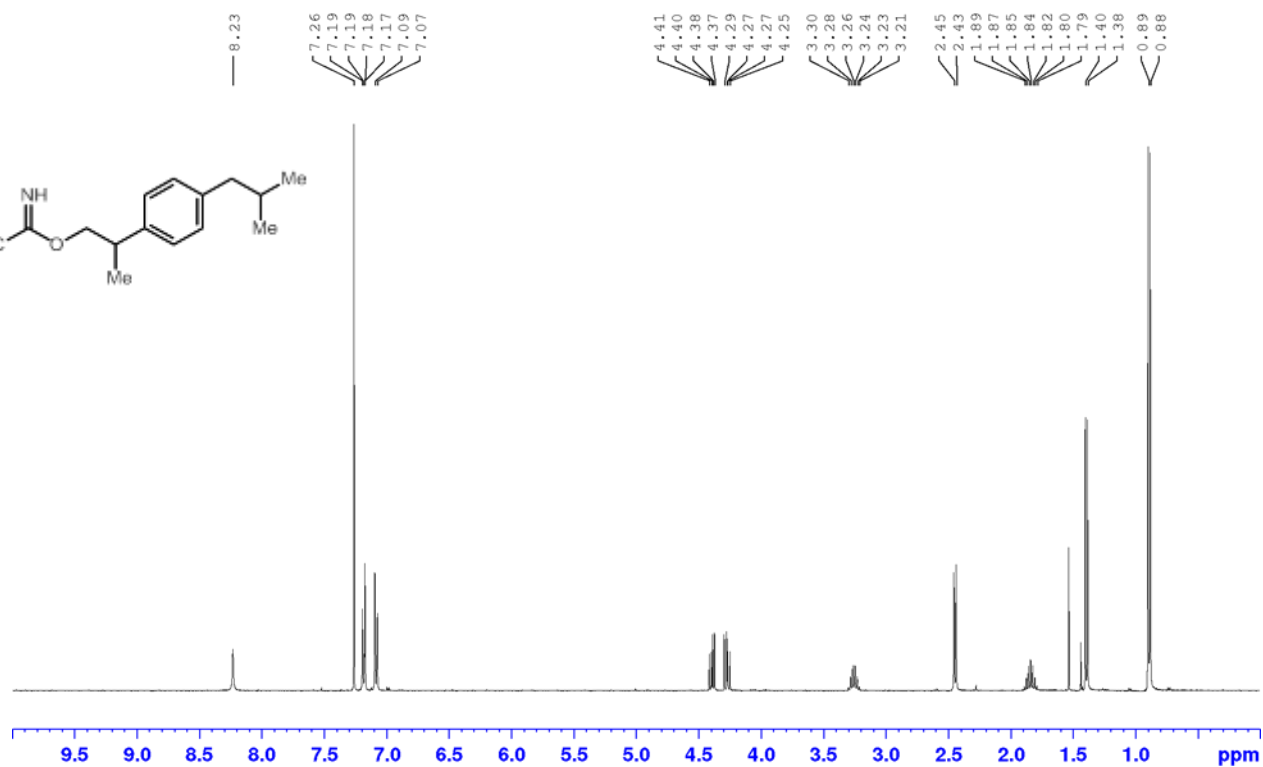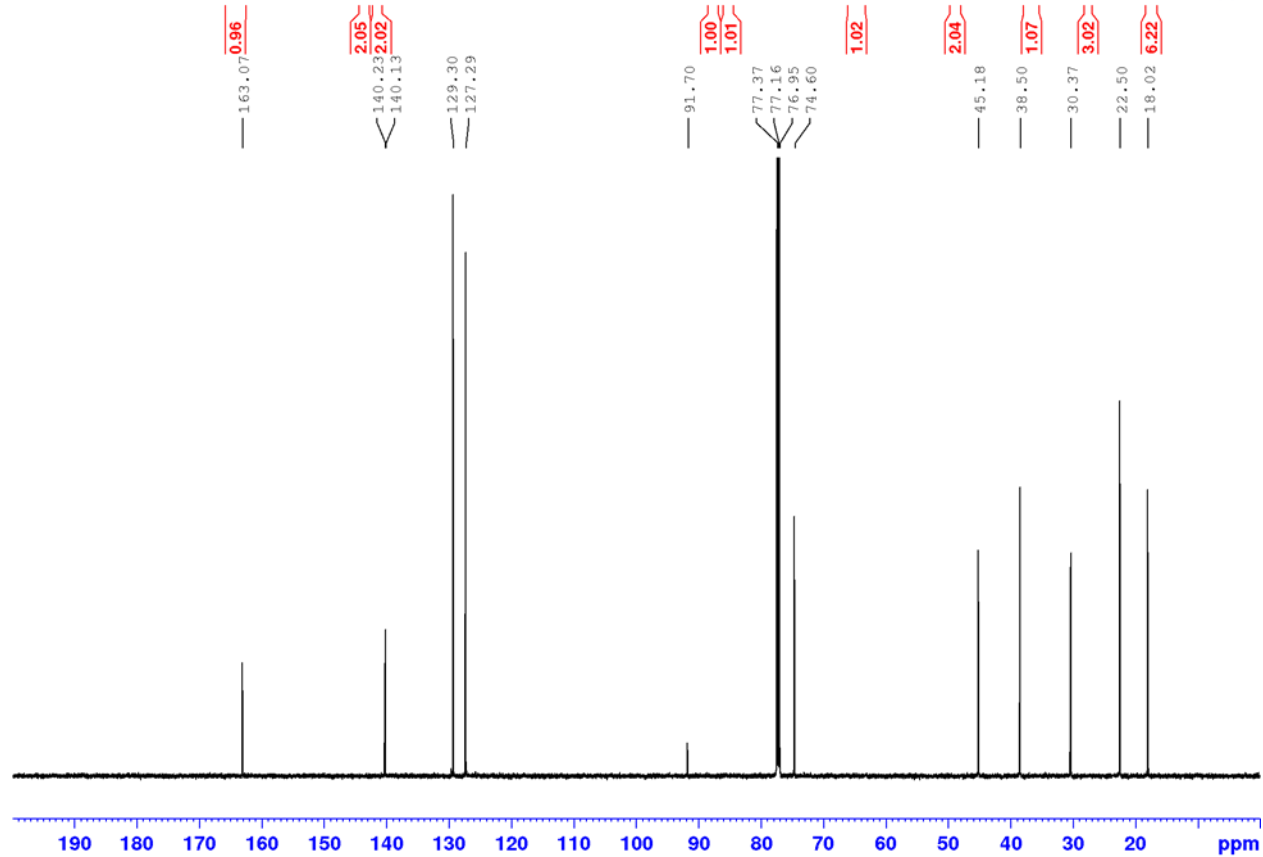

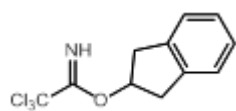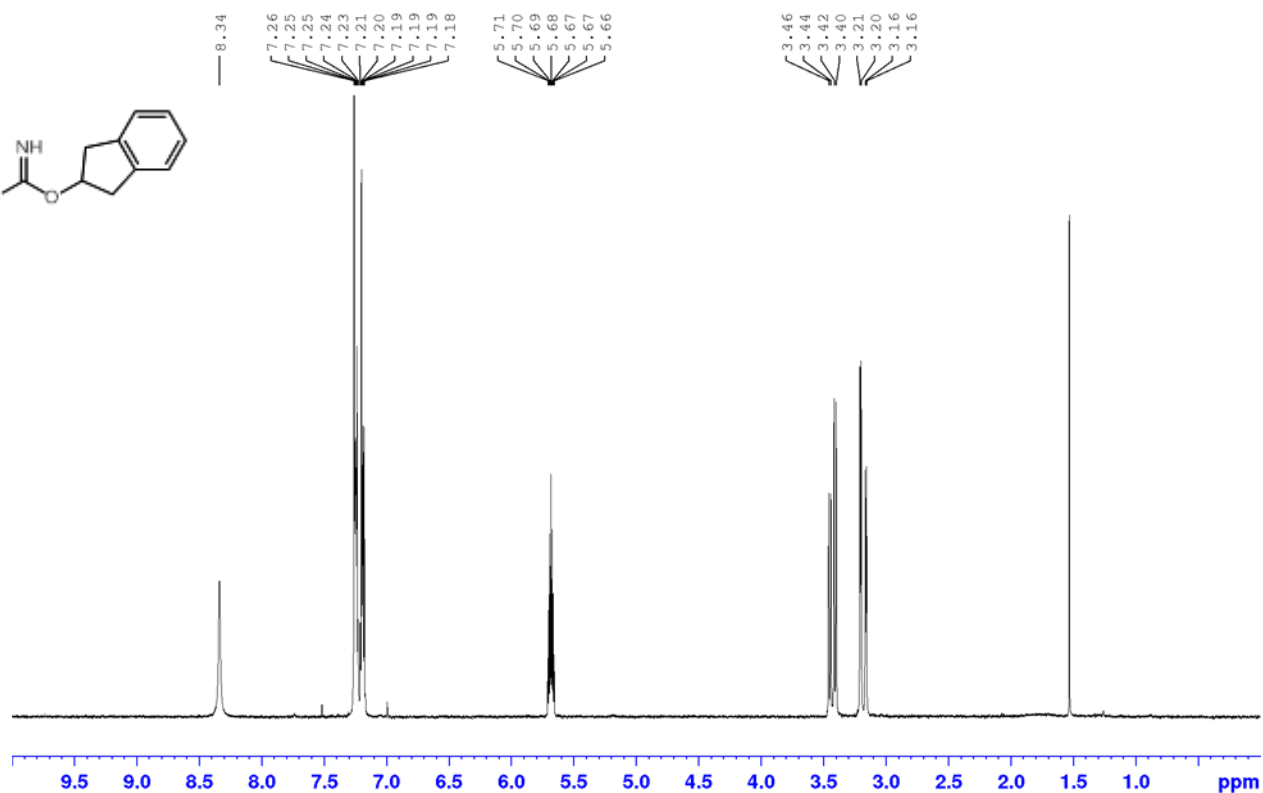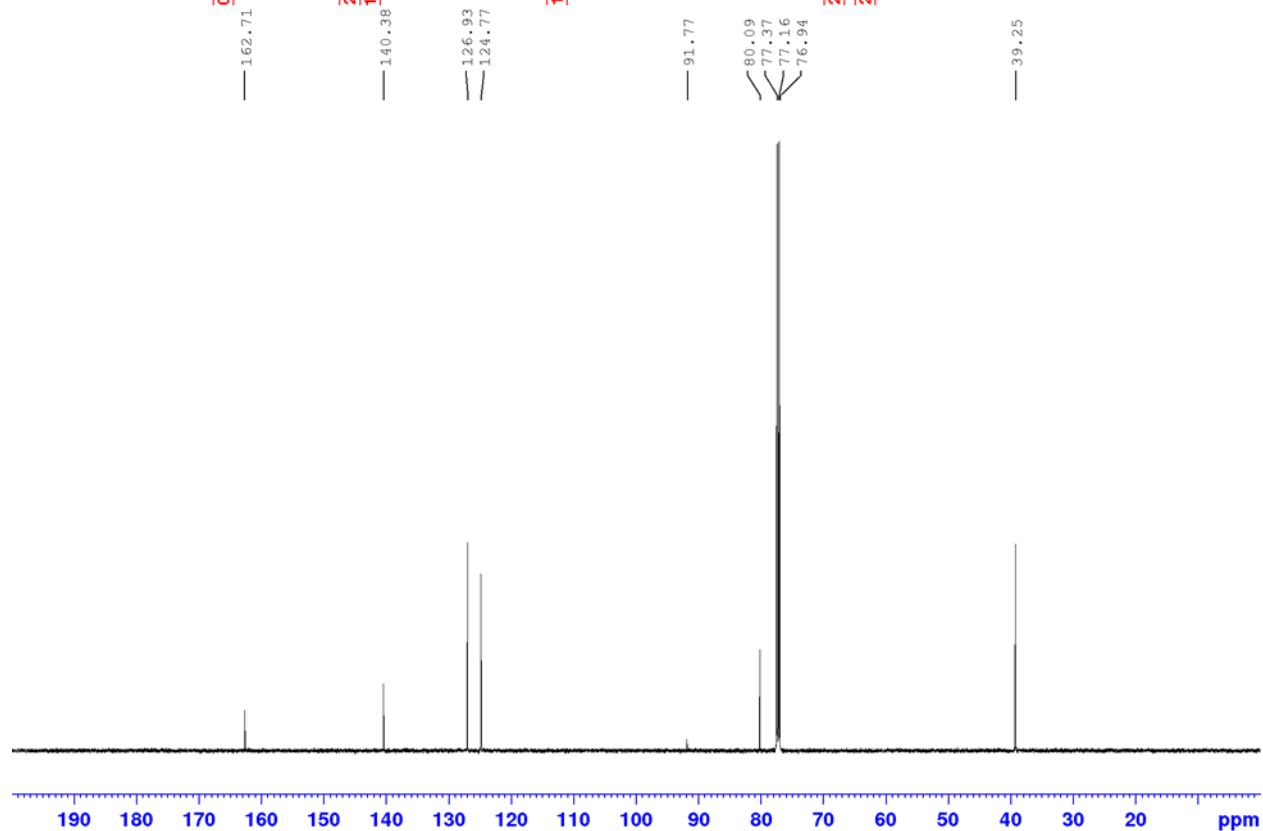

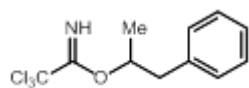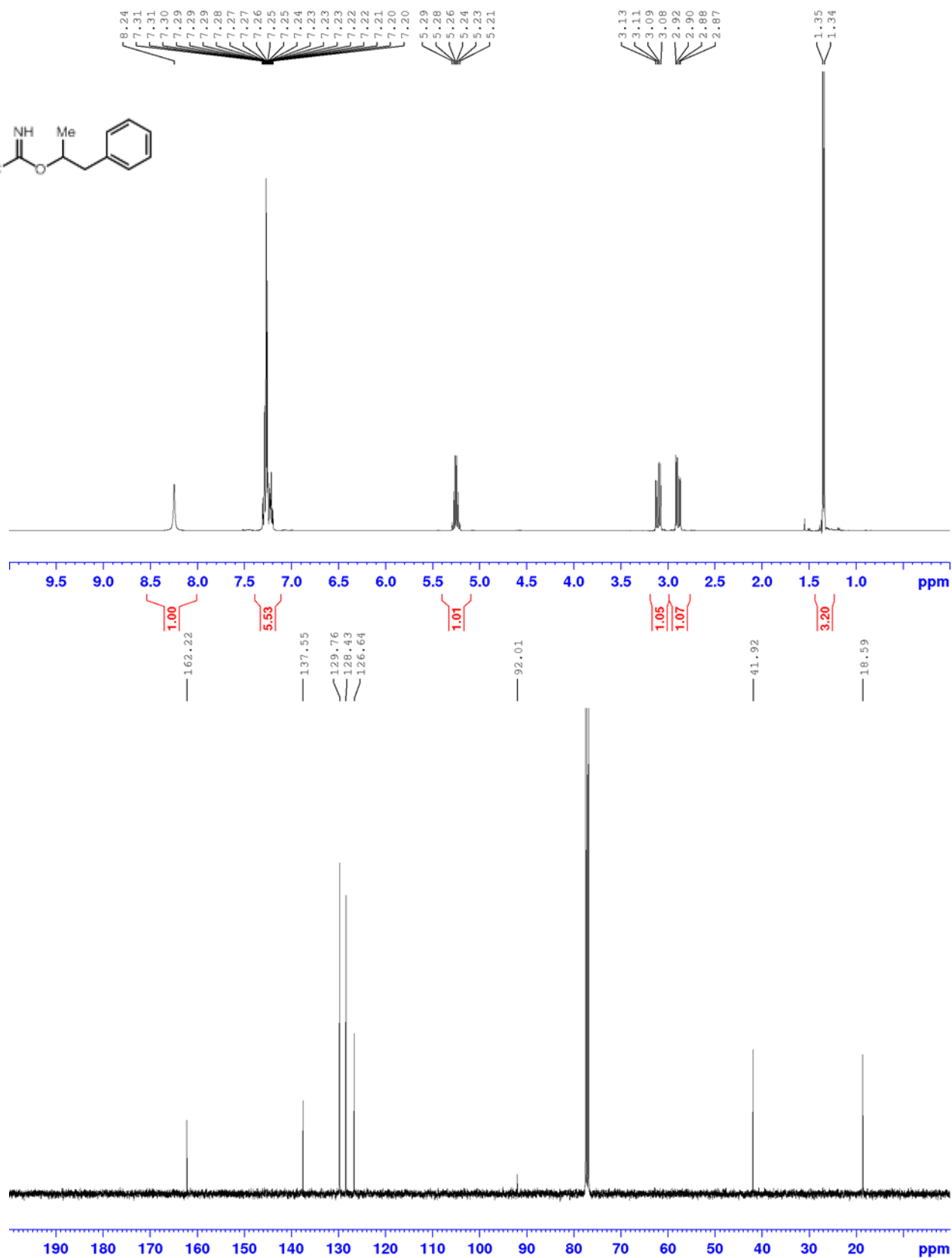

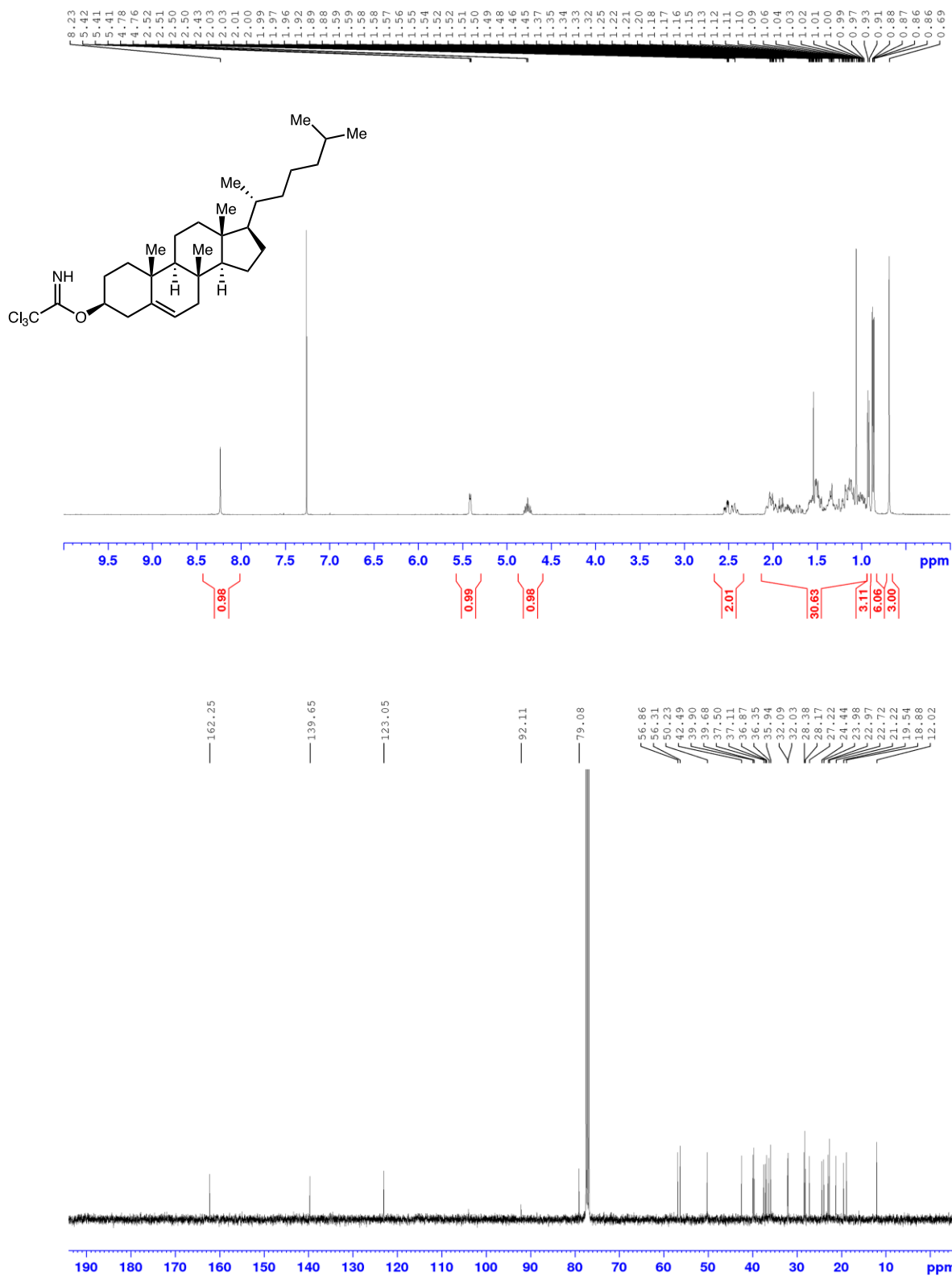

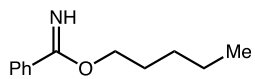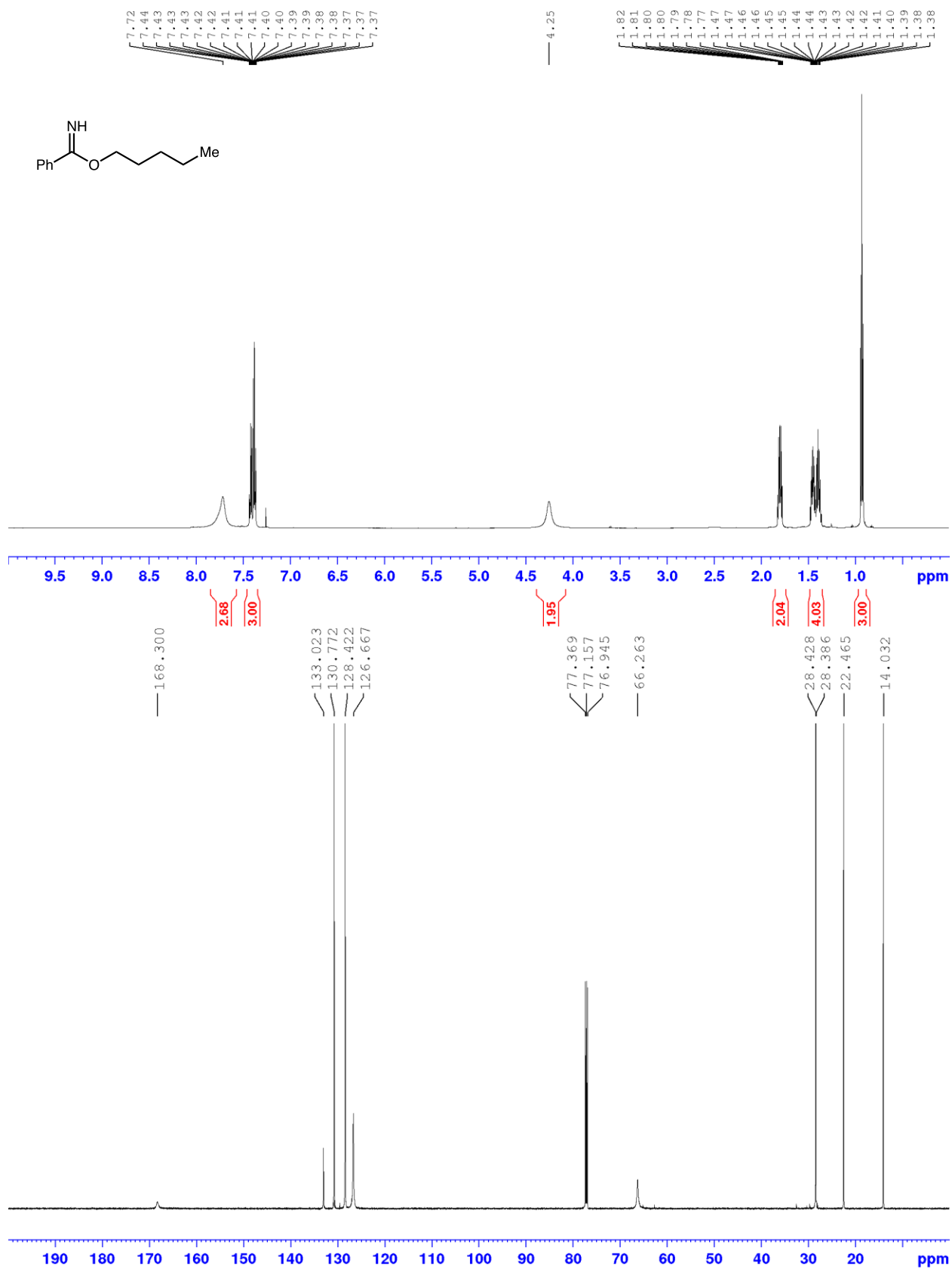

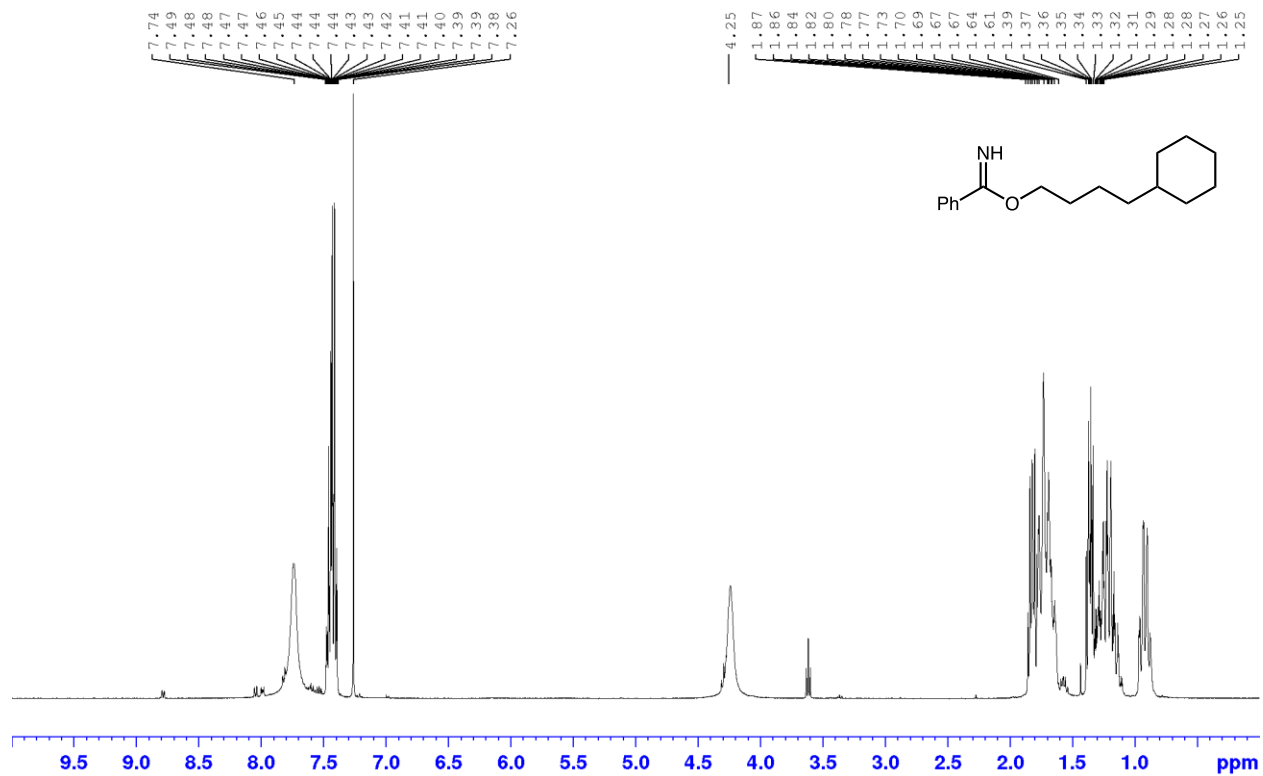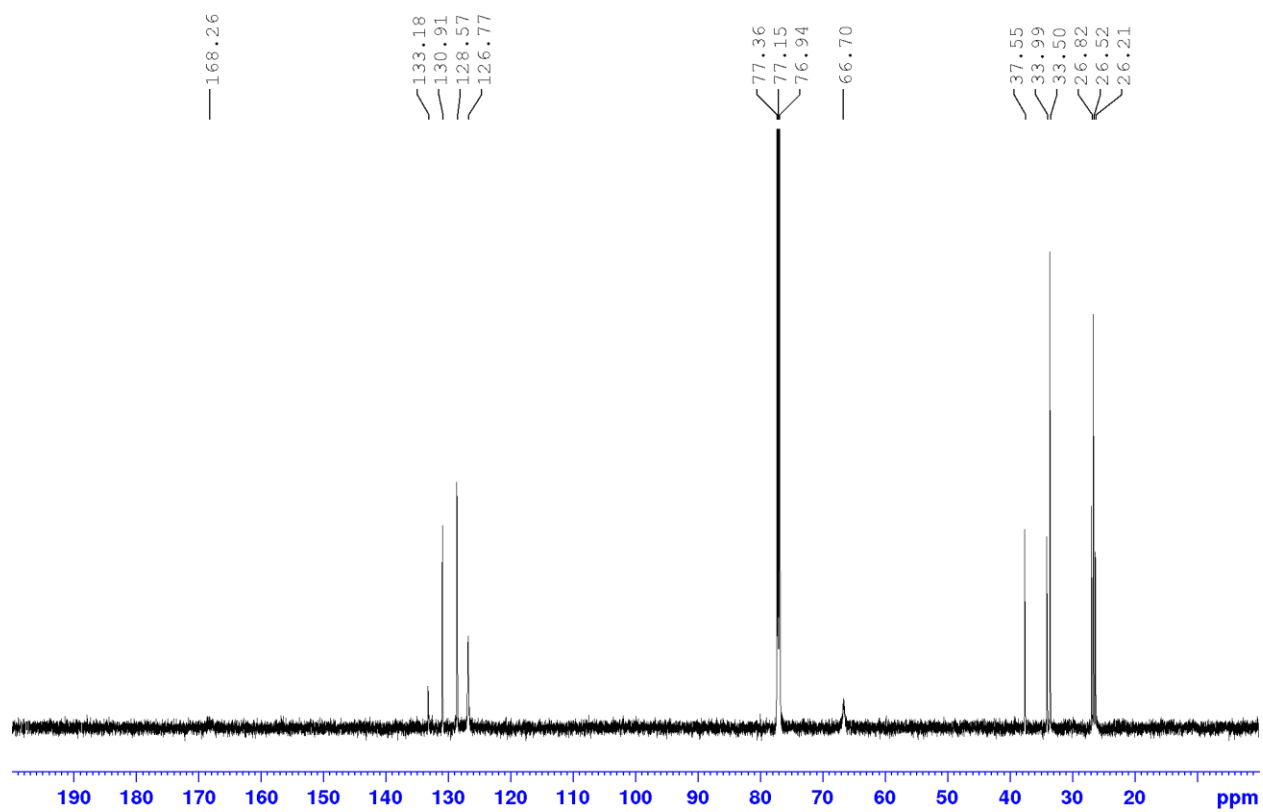

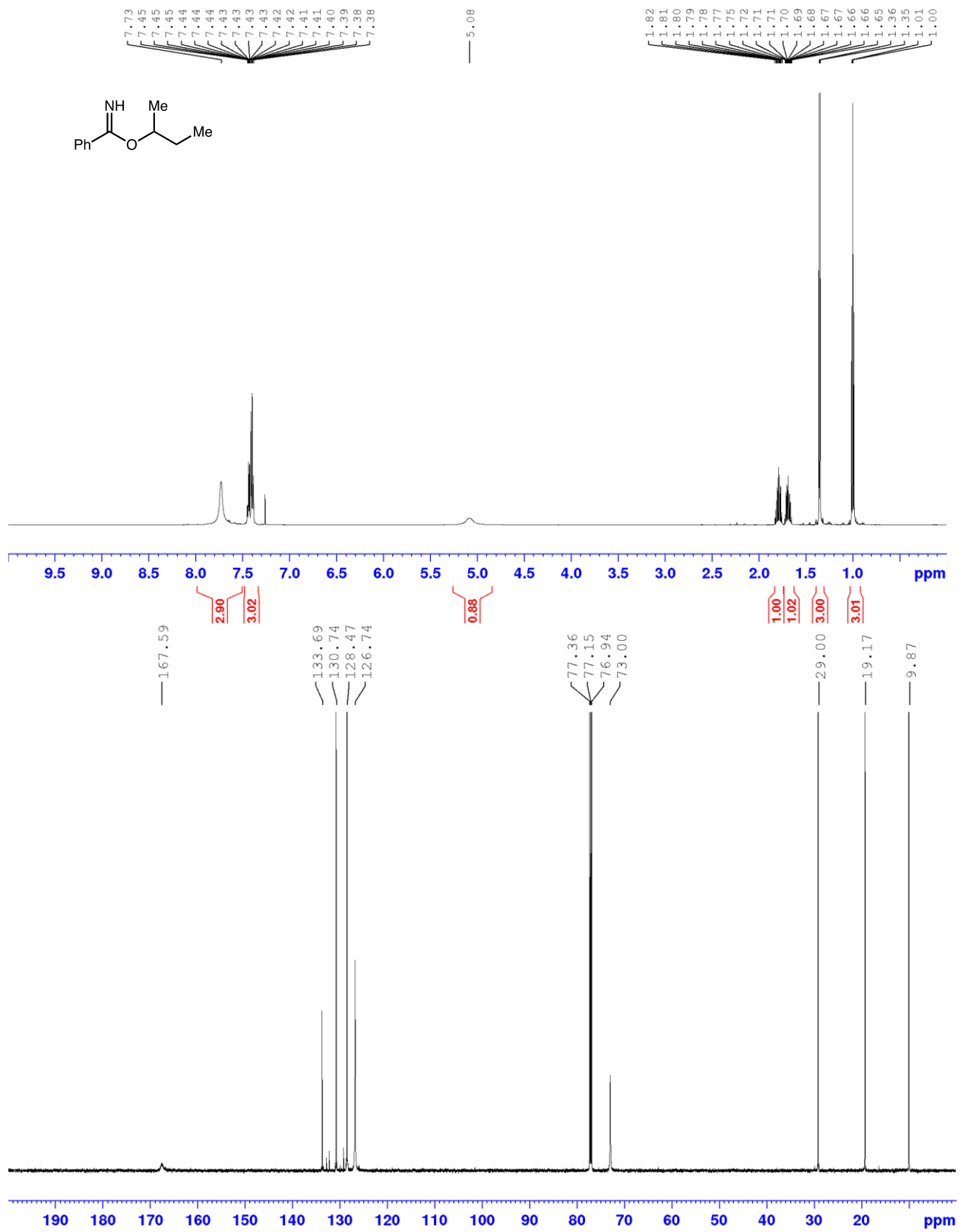

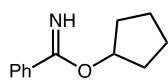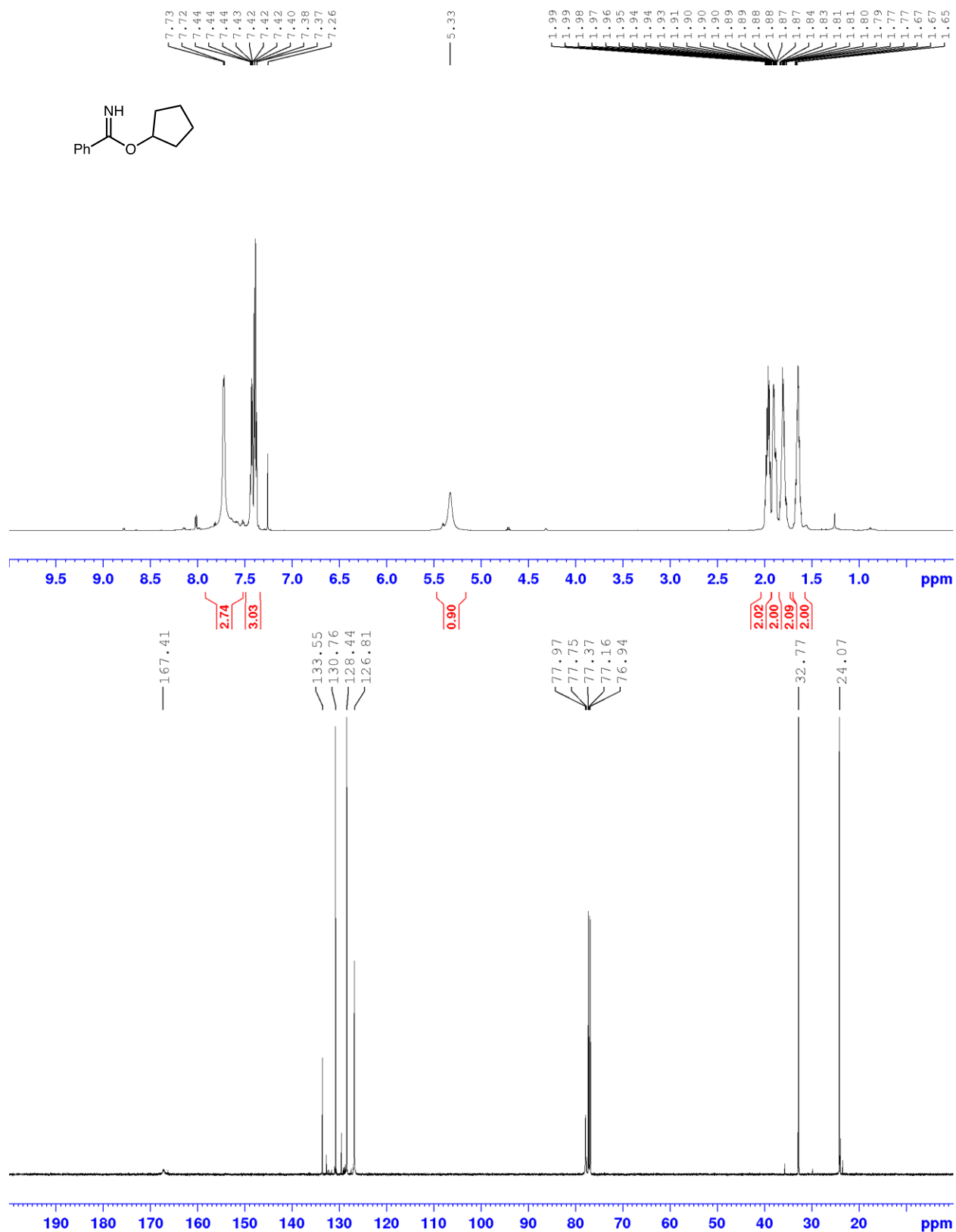

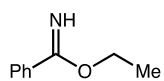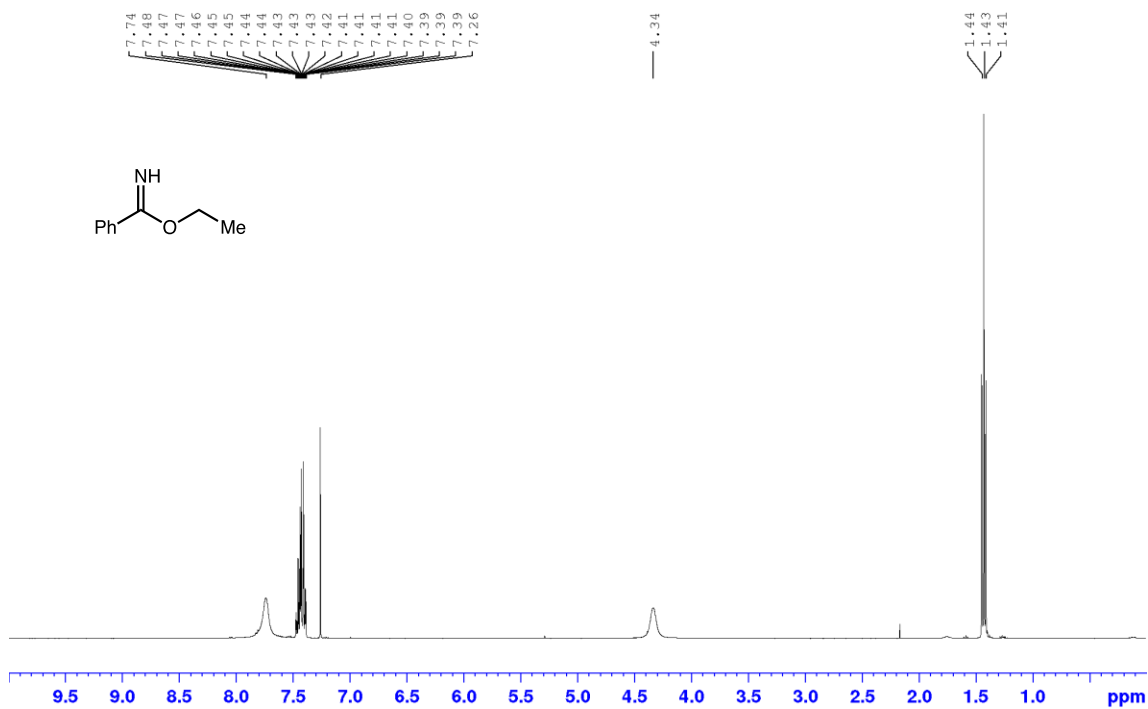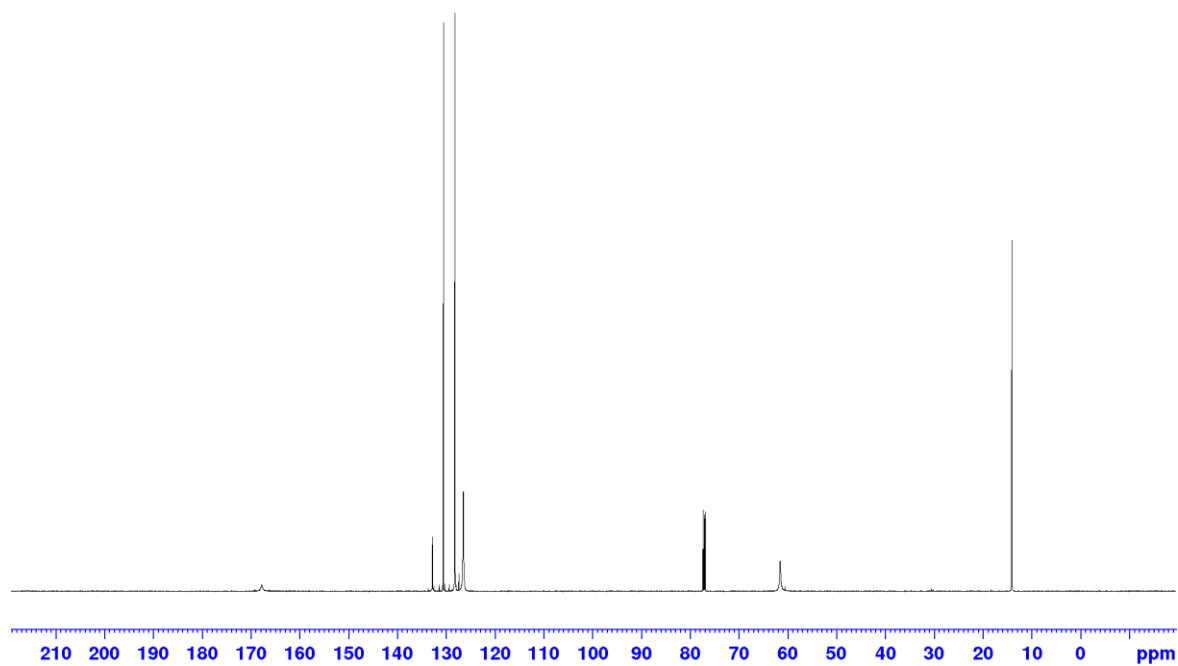

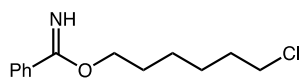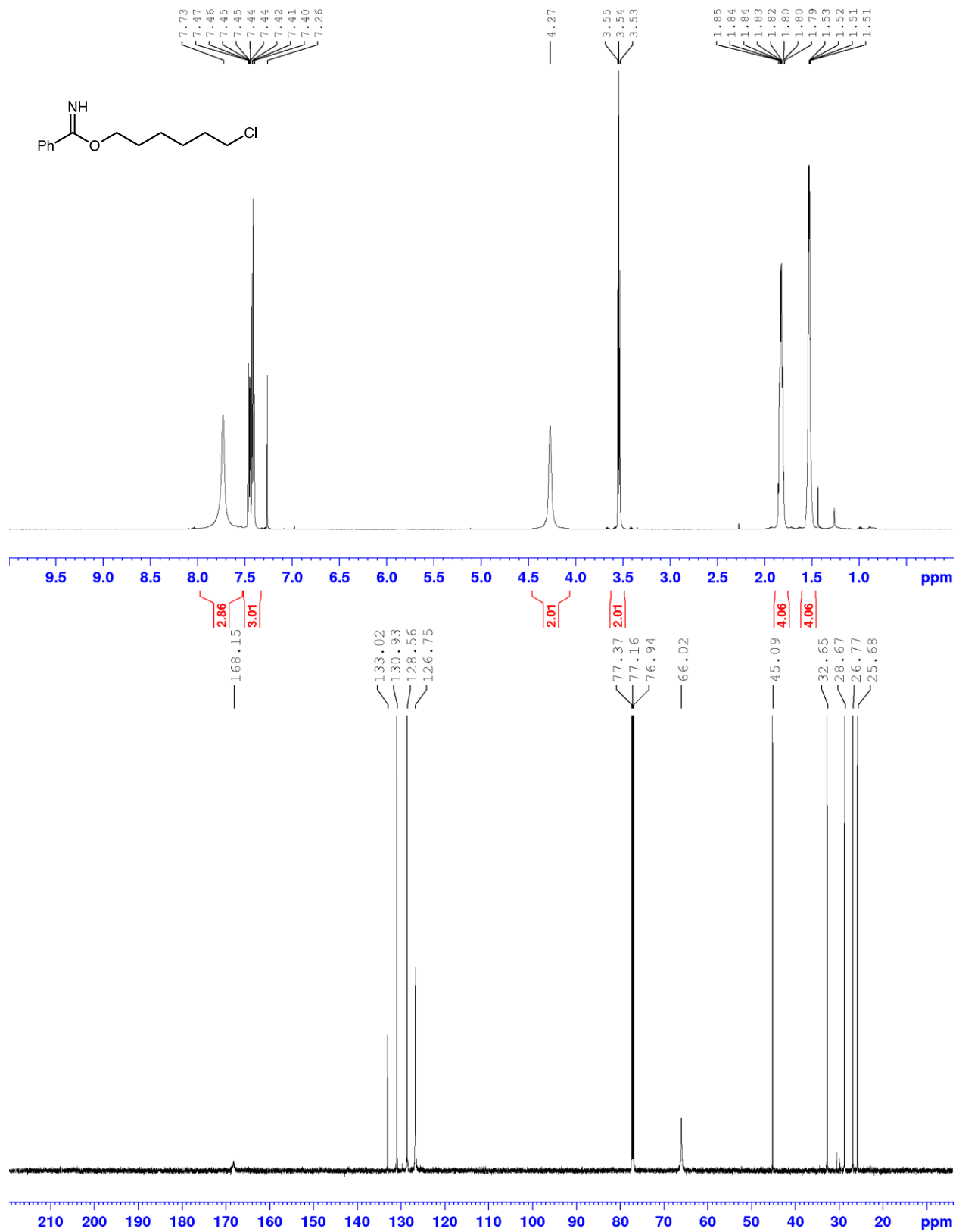

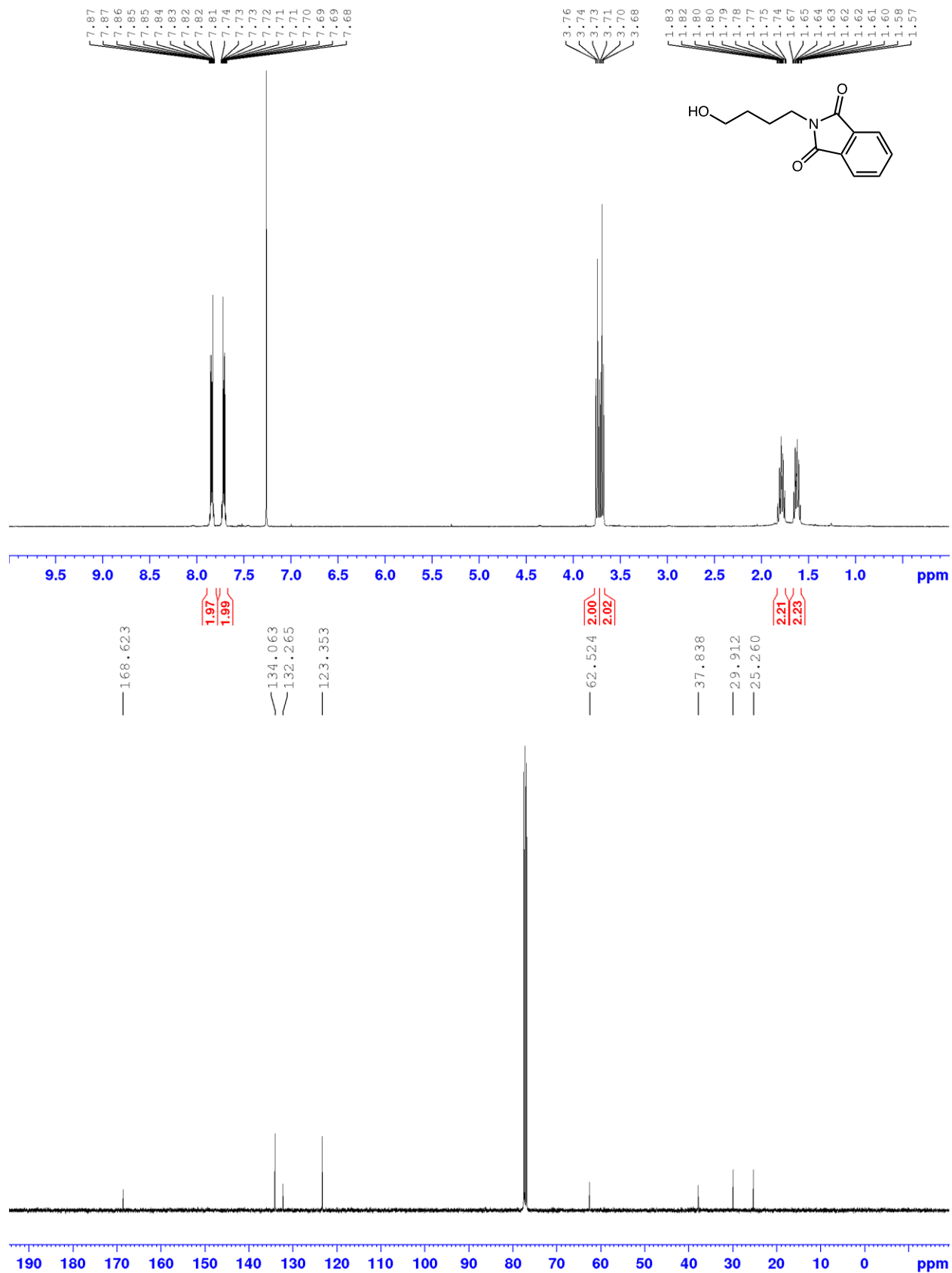

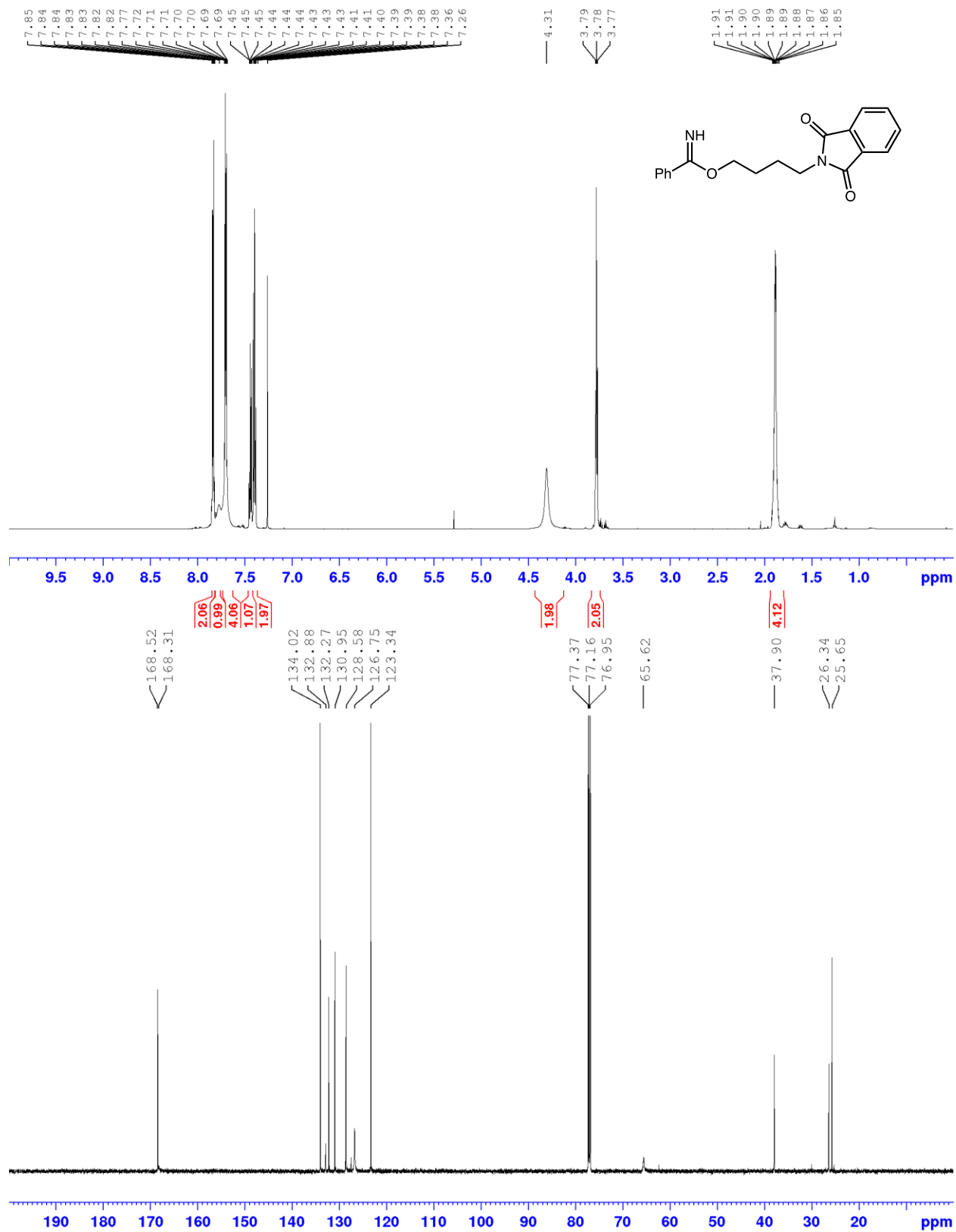

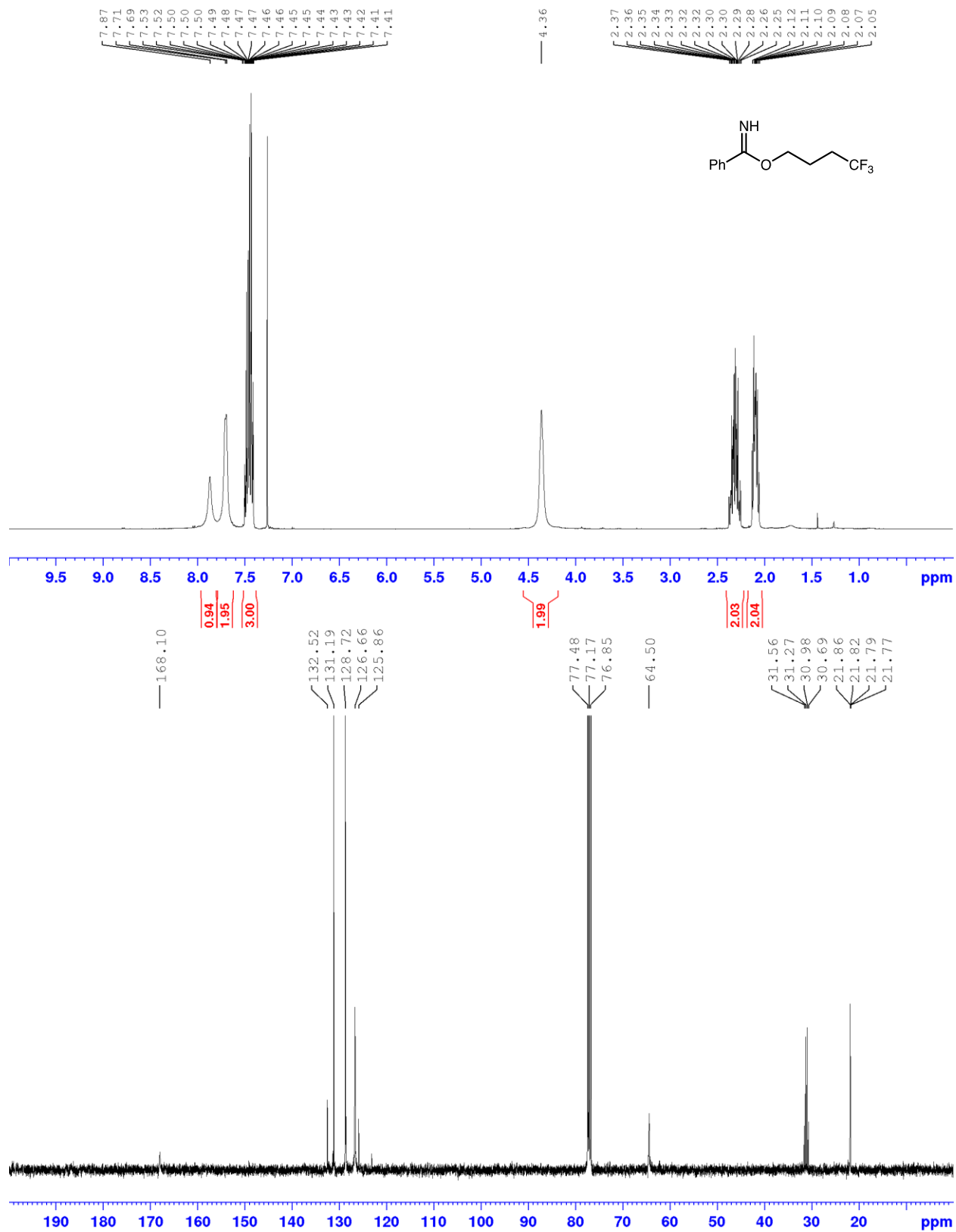

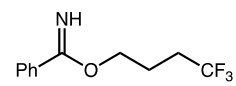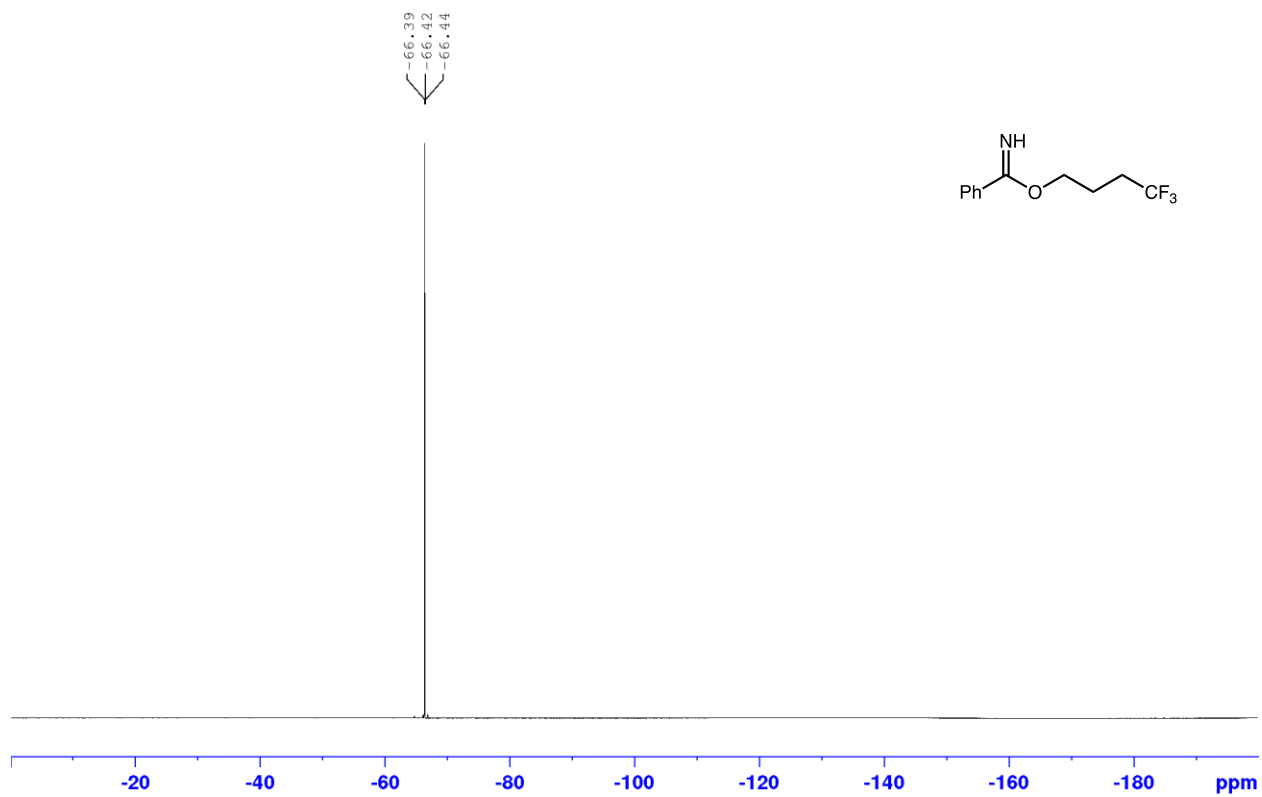

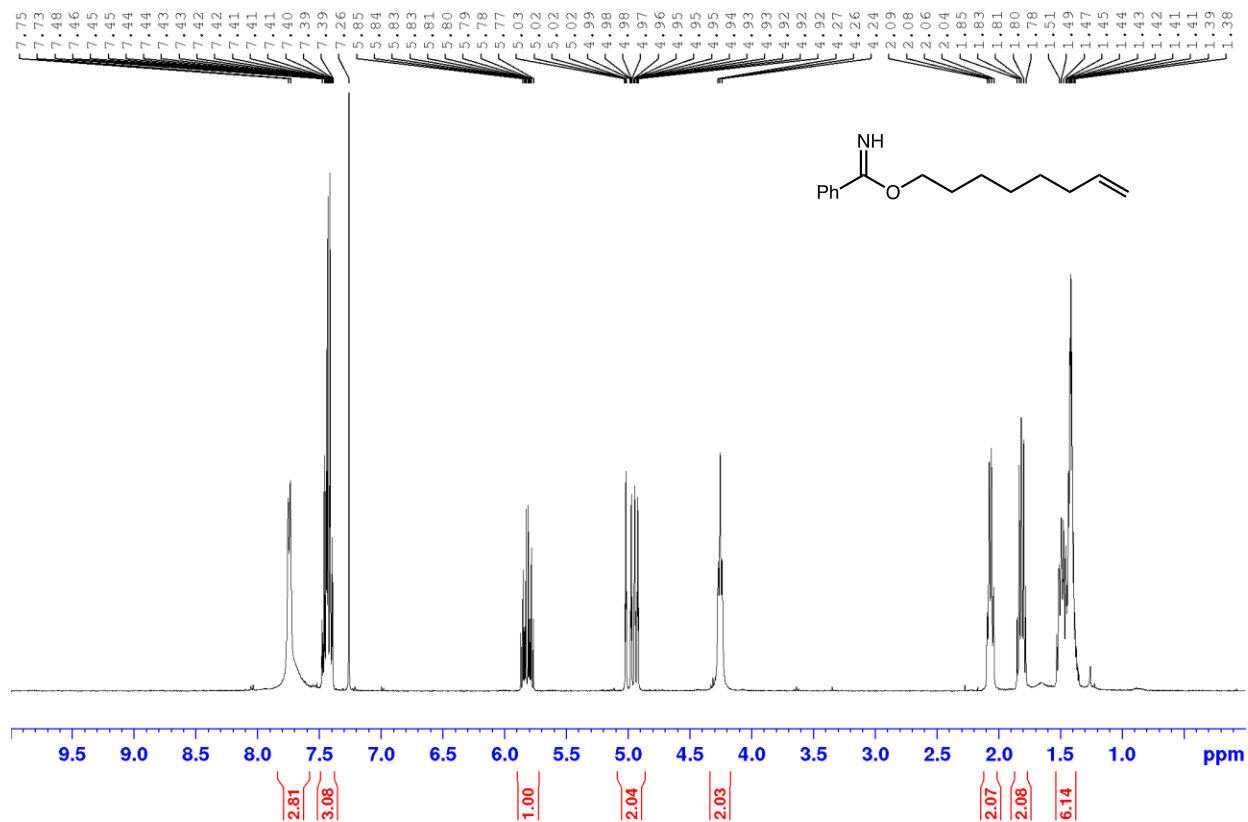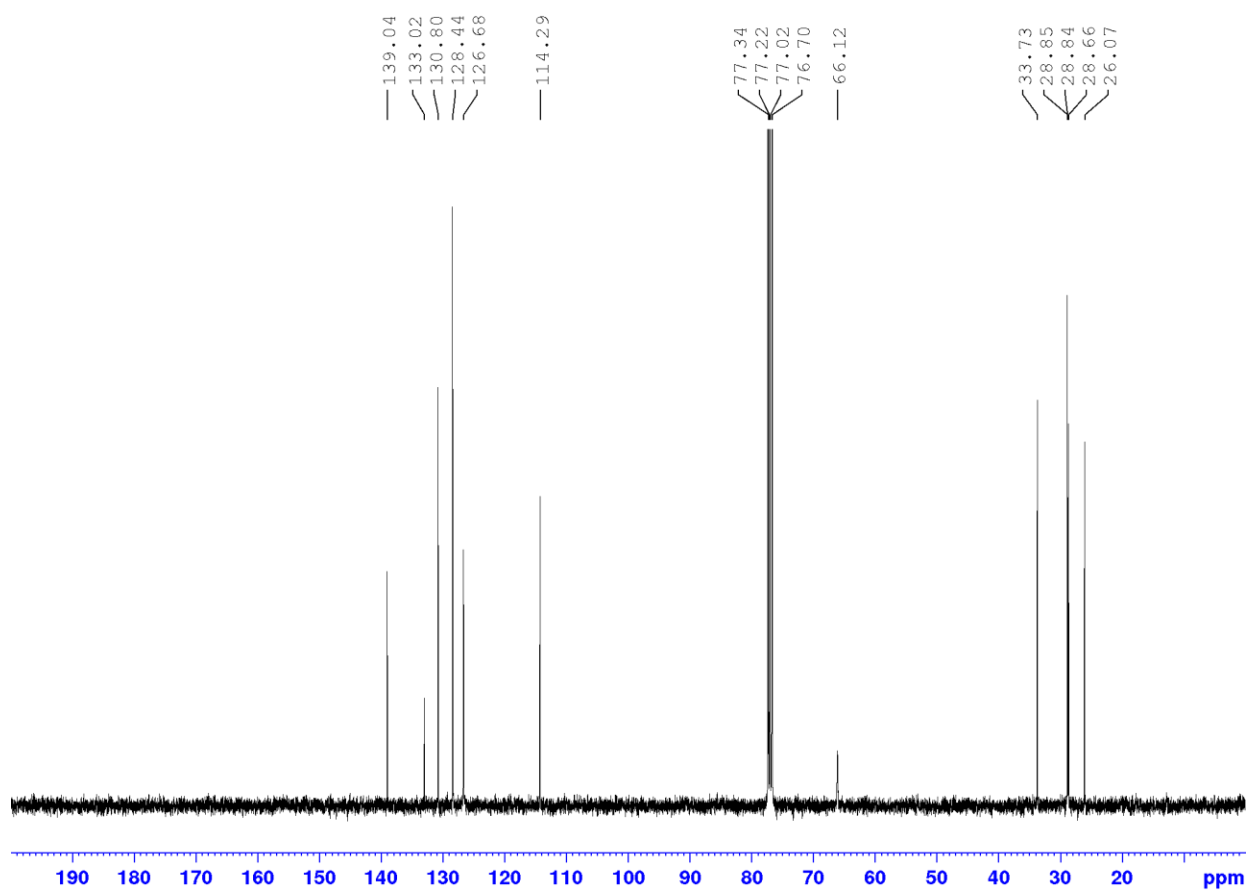

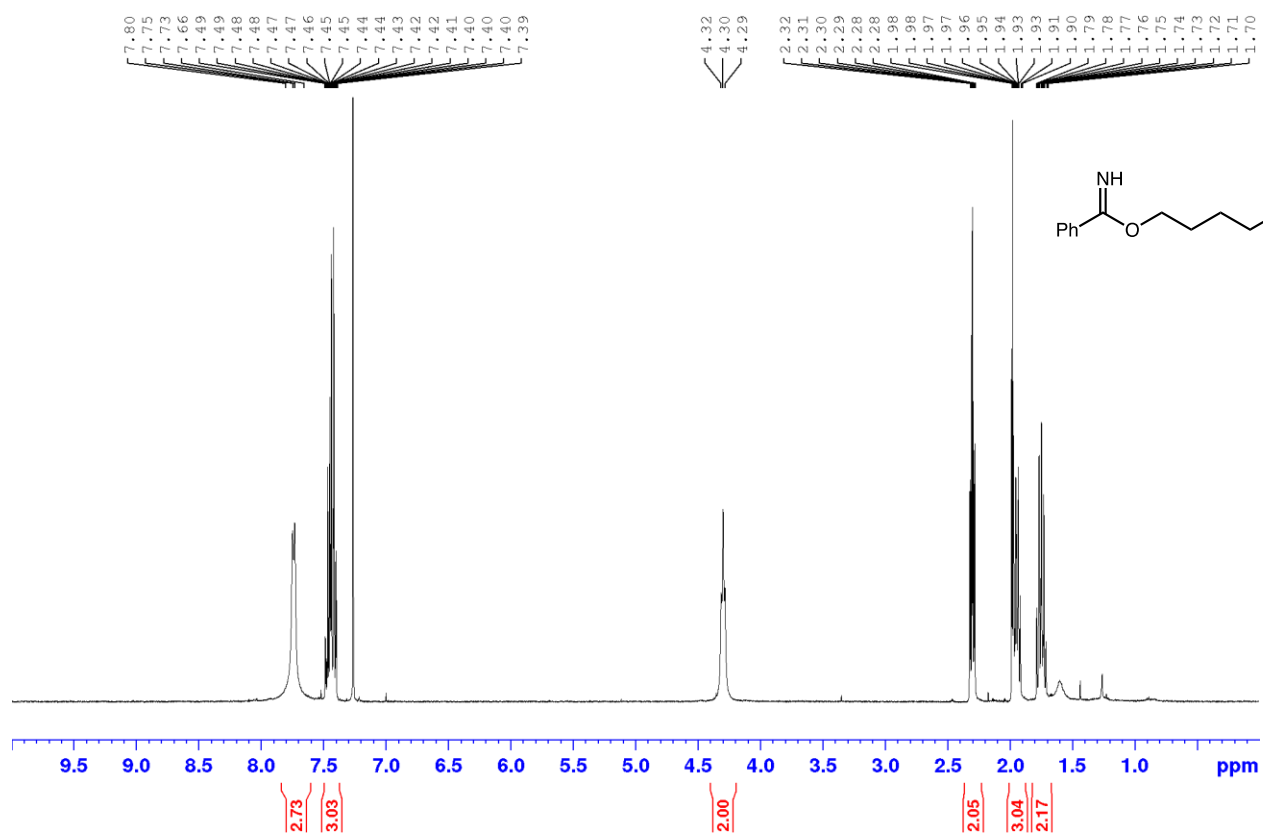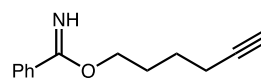

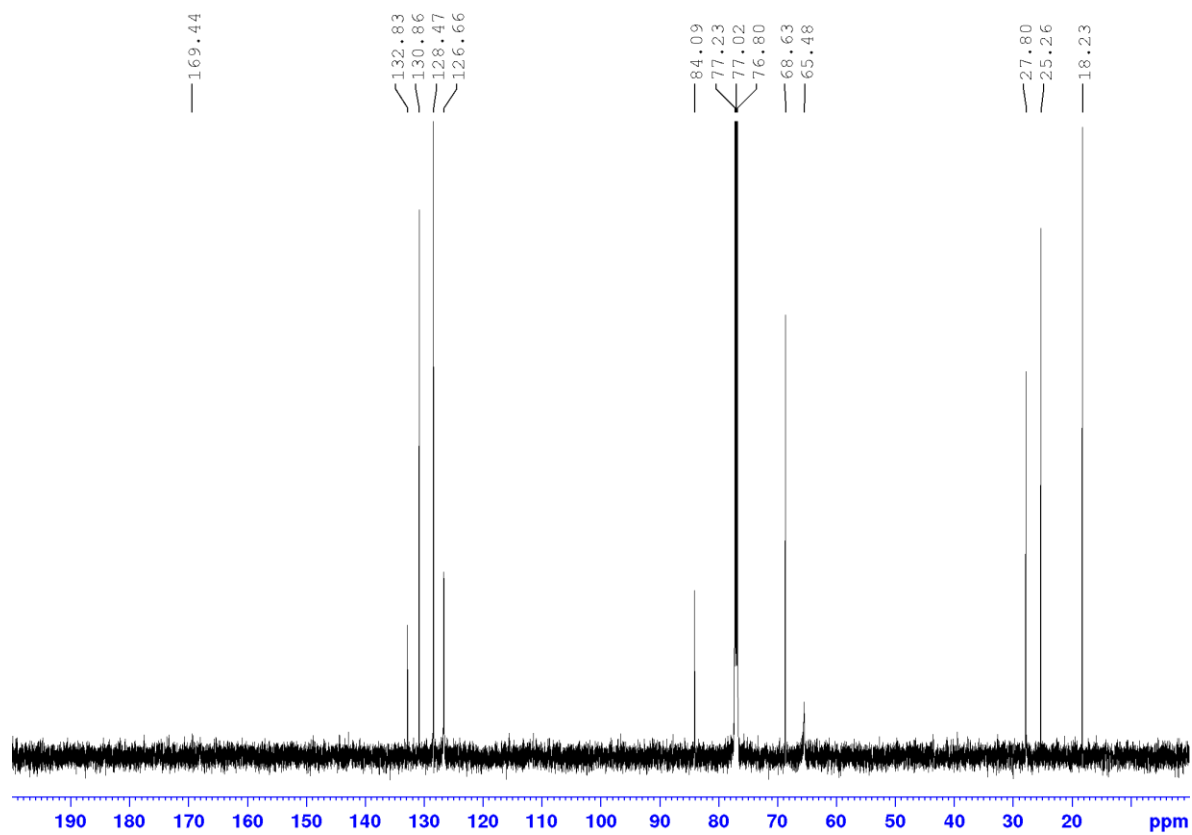

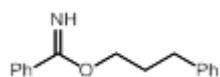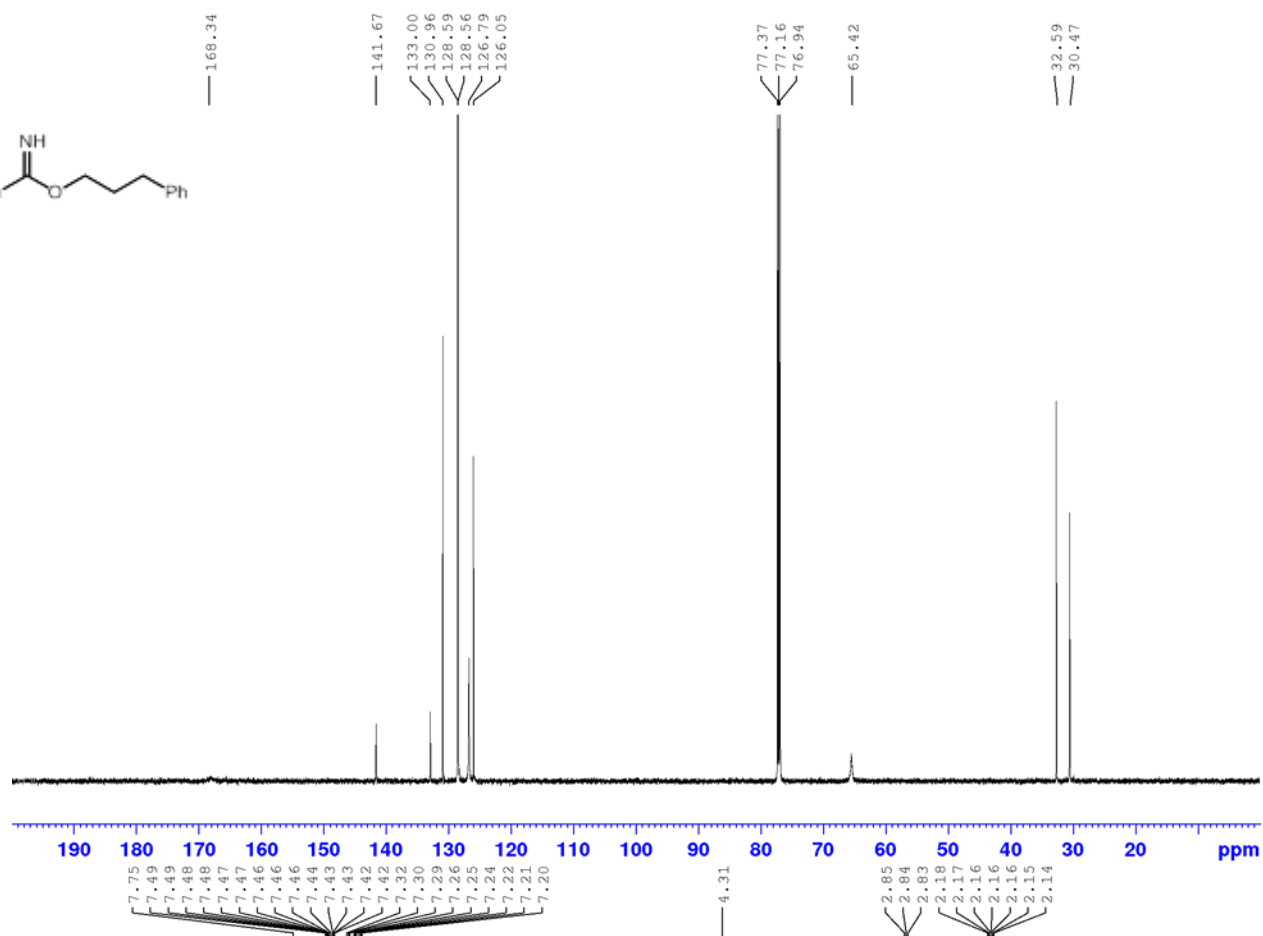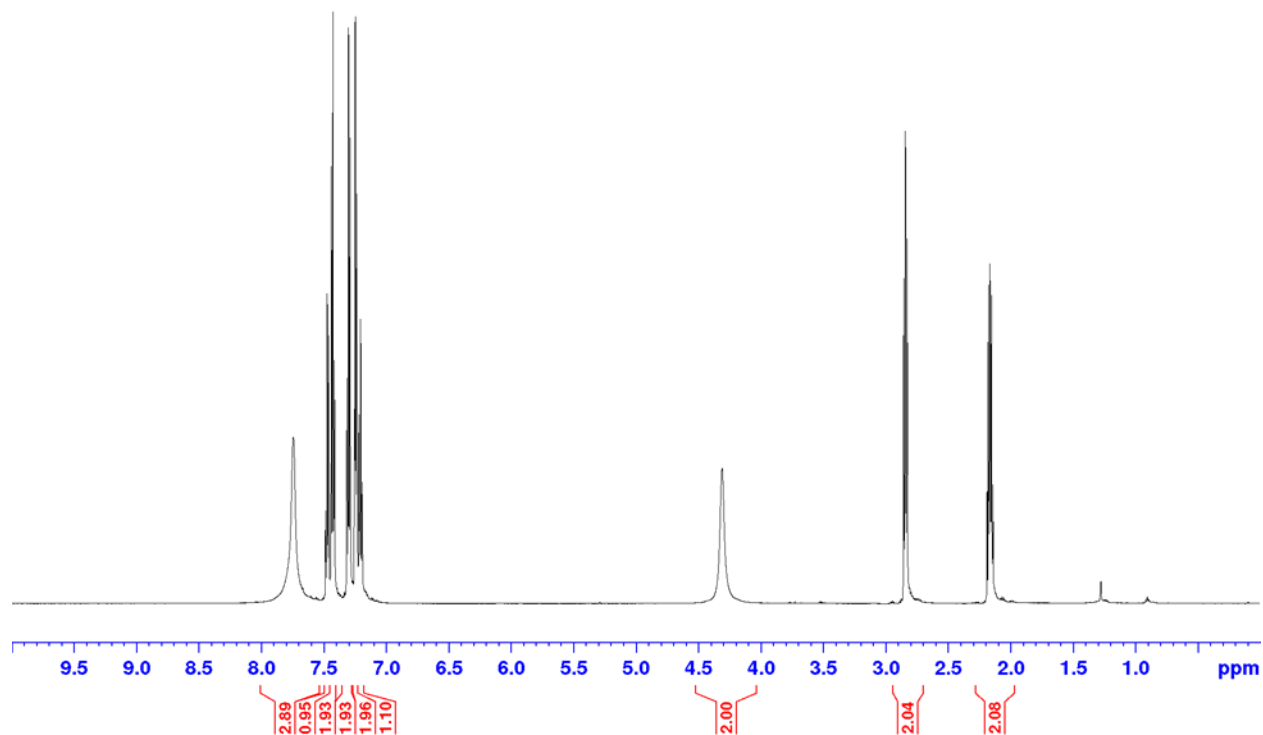

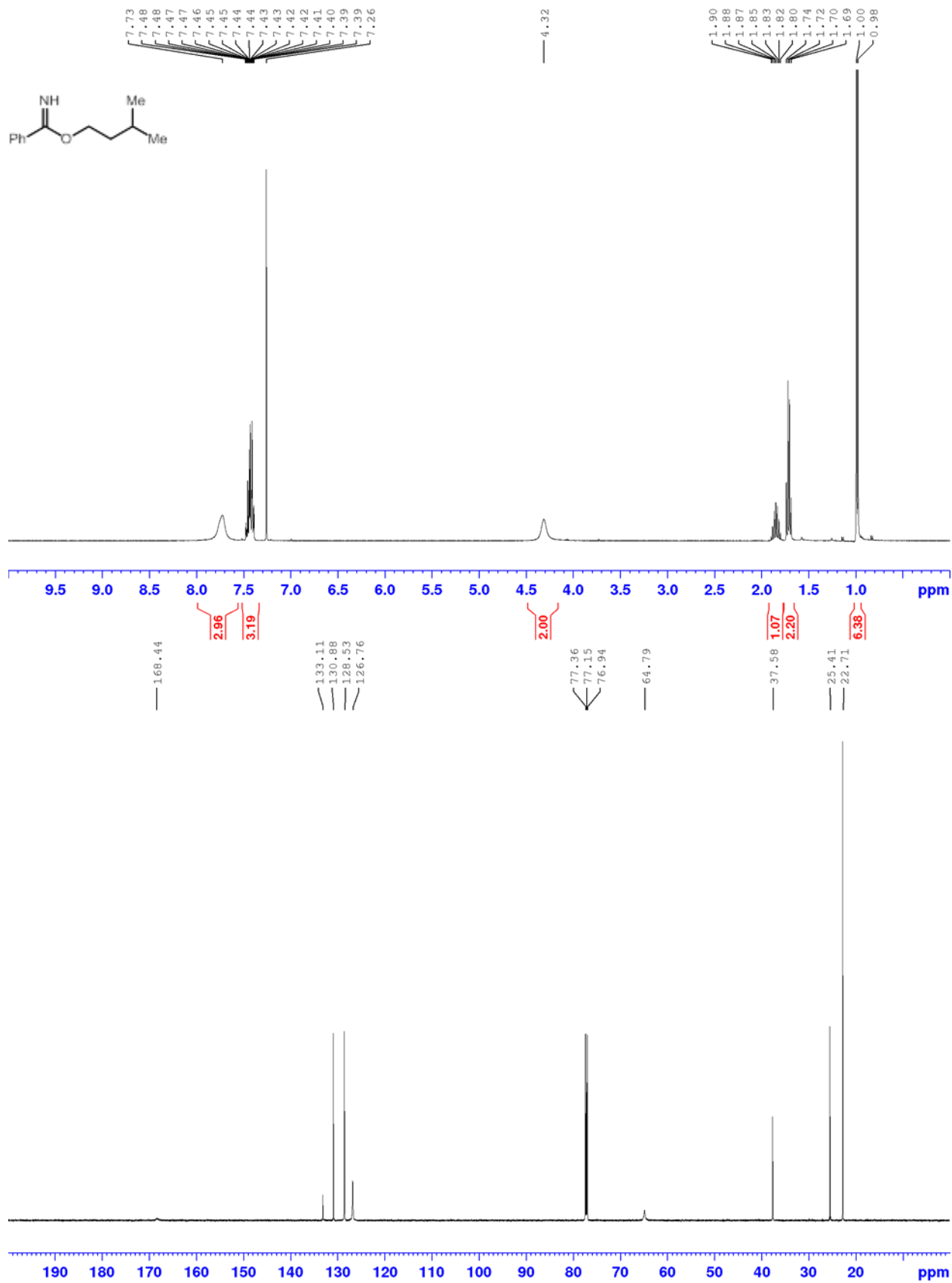

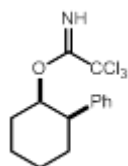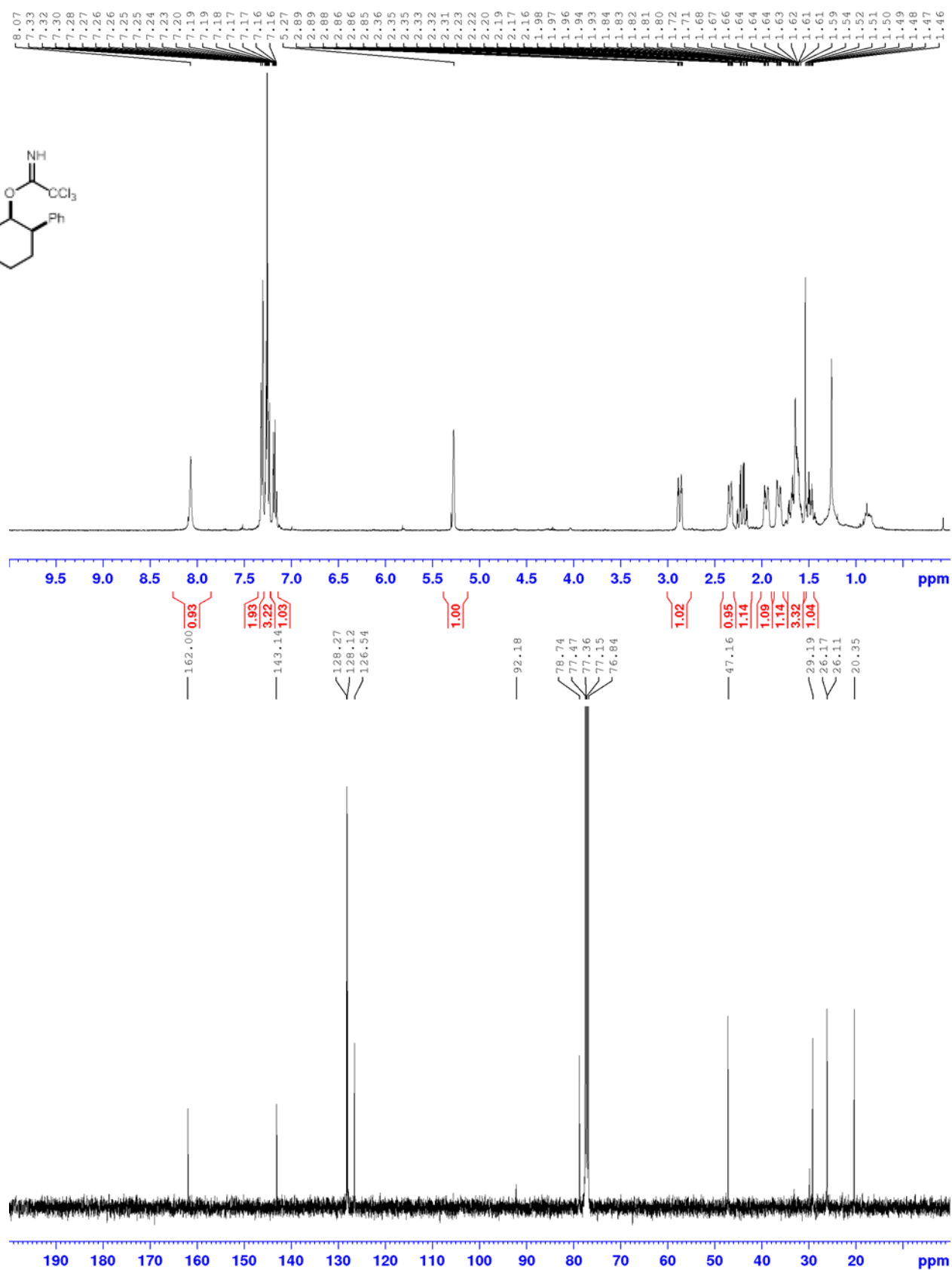

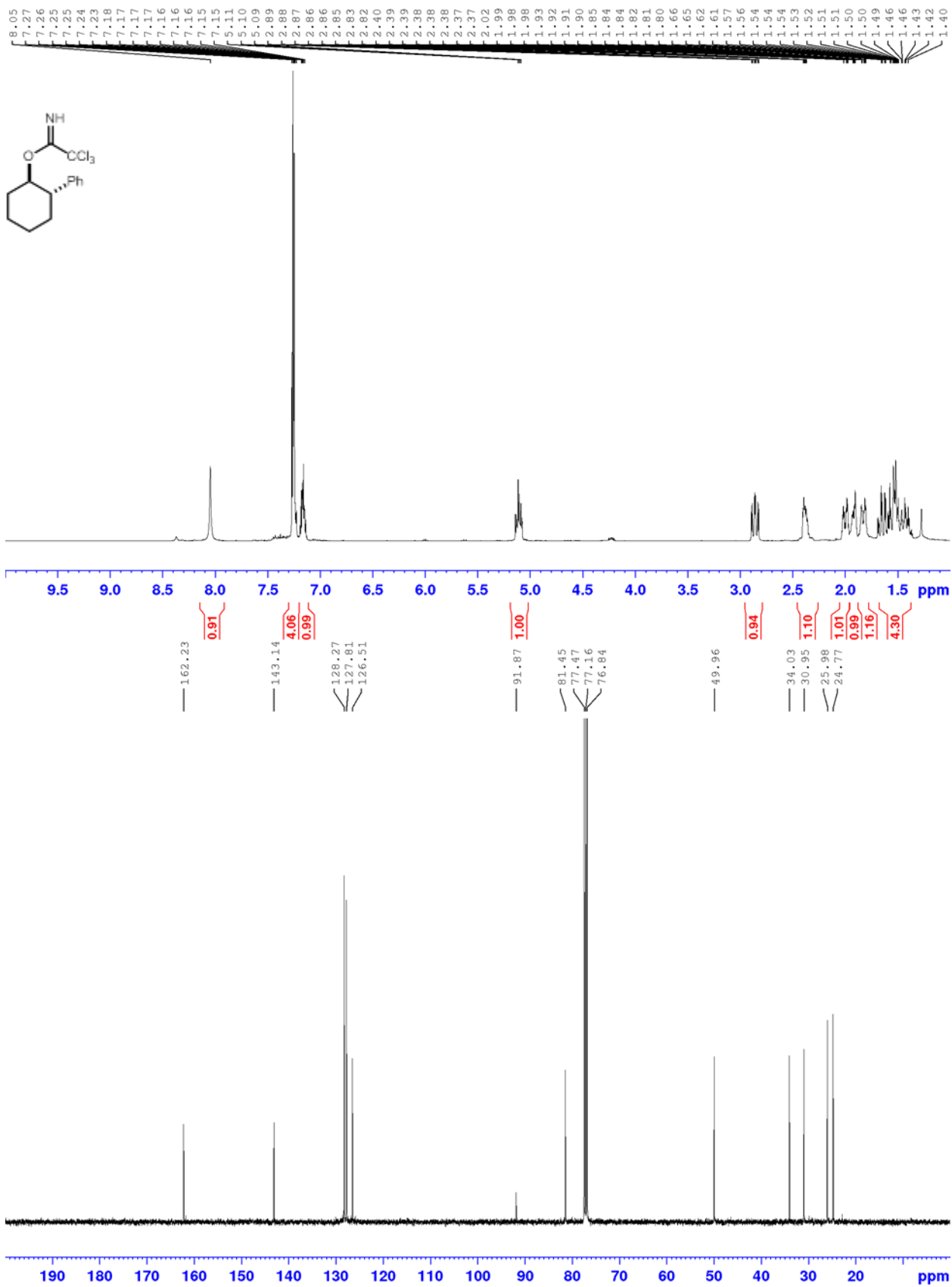

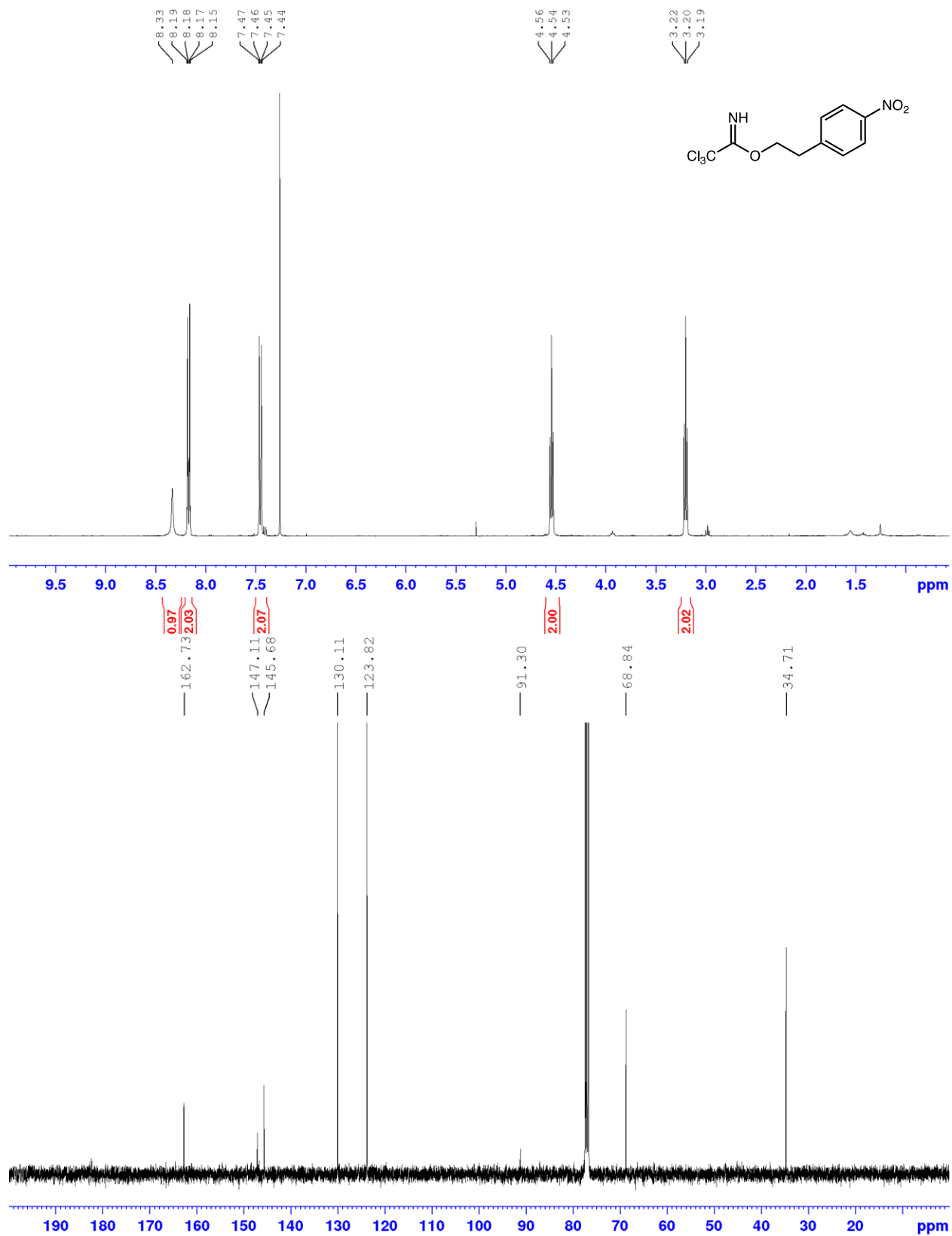

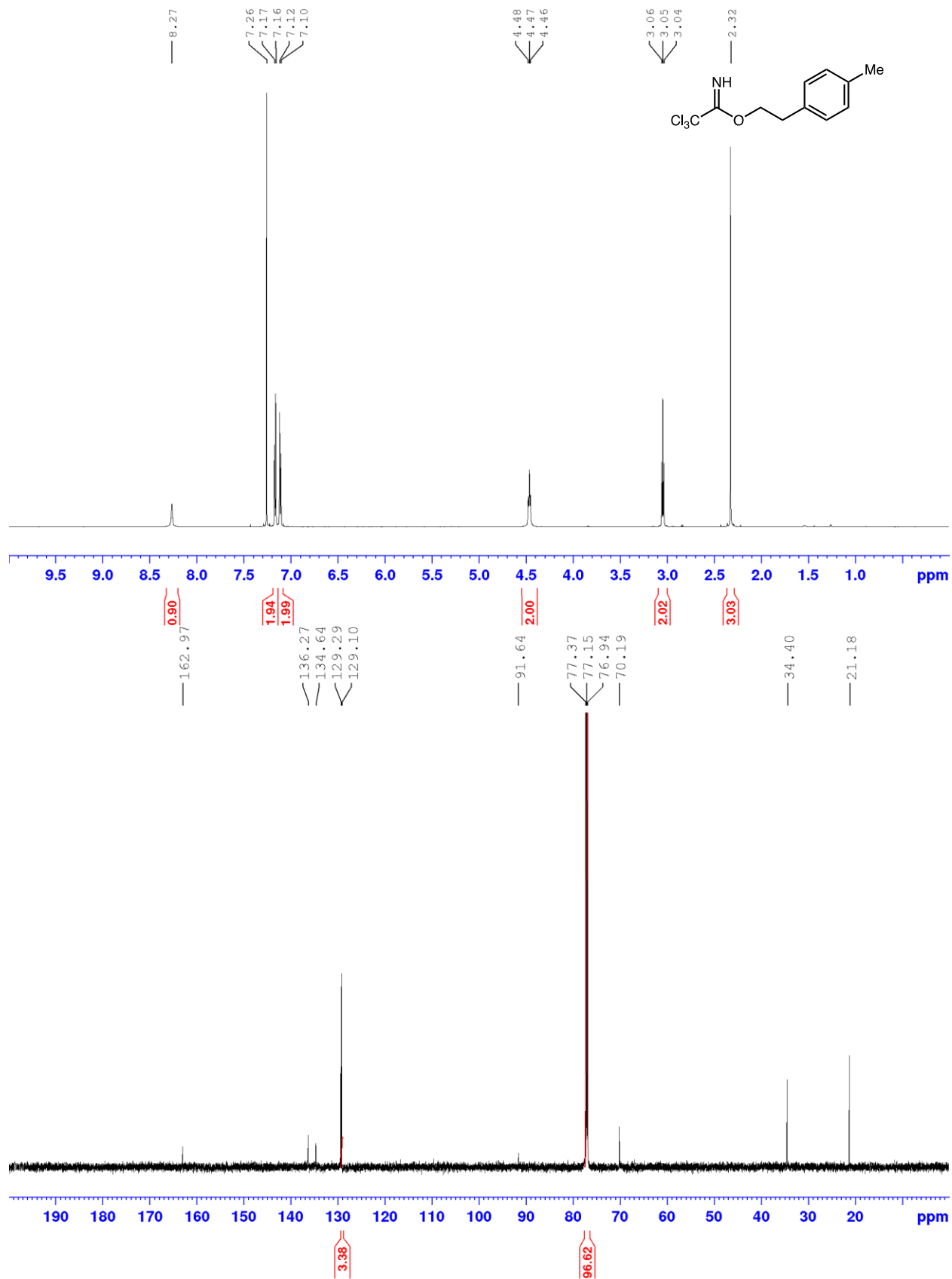

# Amino Alcohols

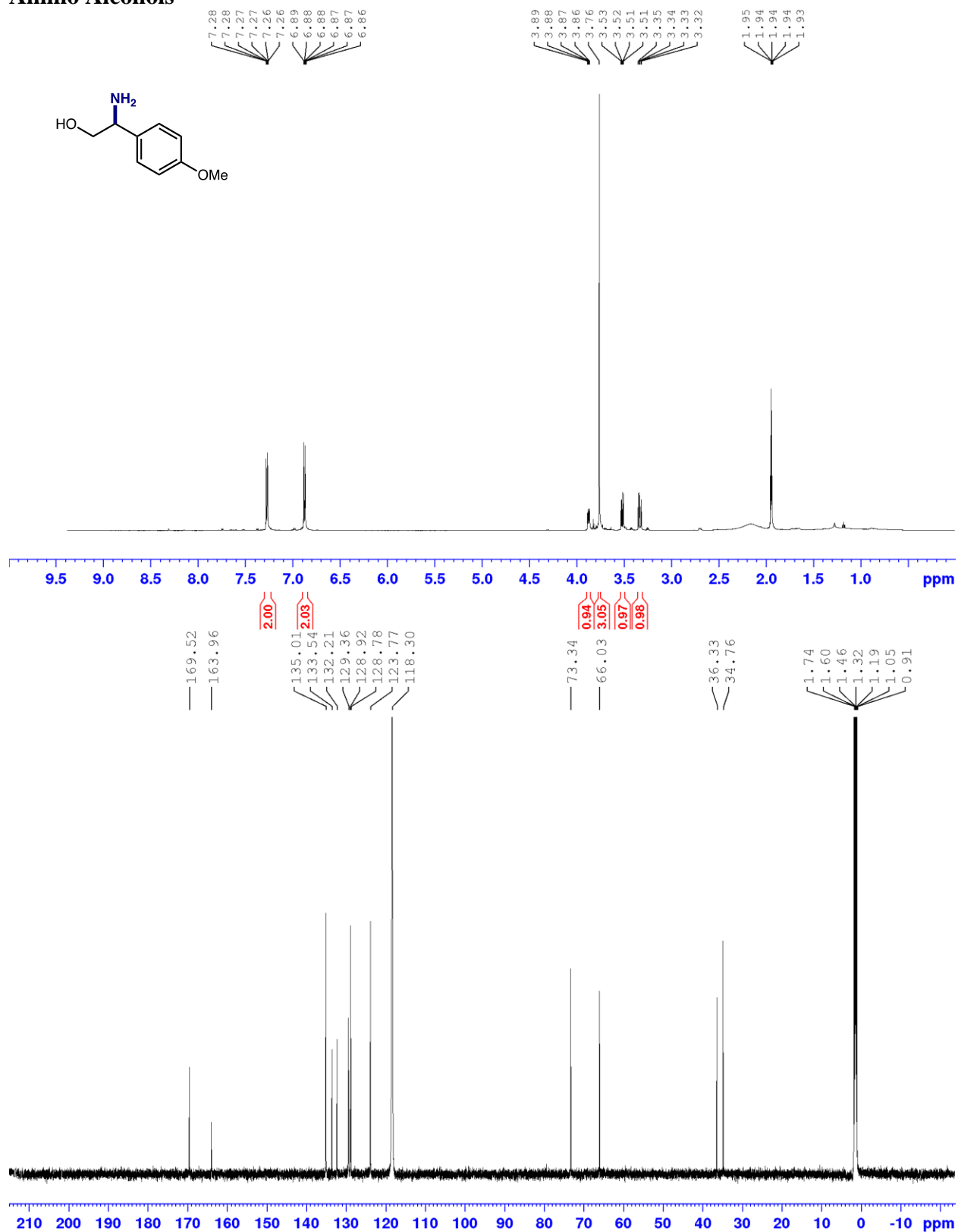

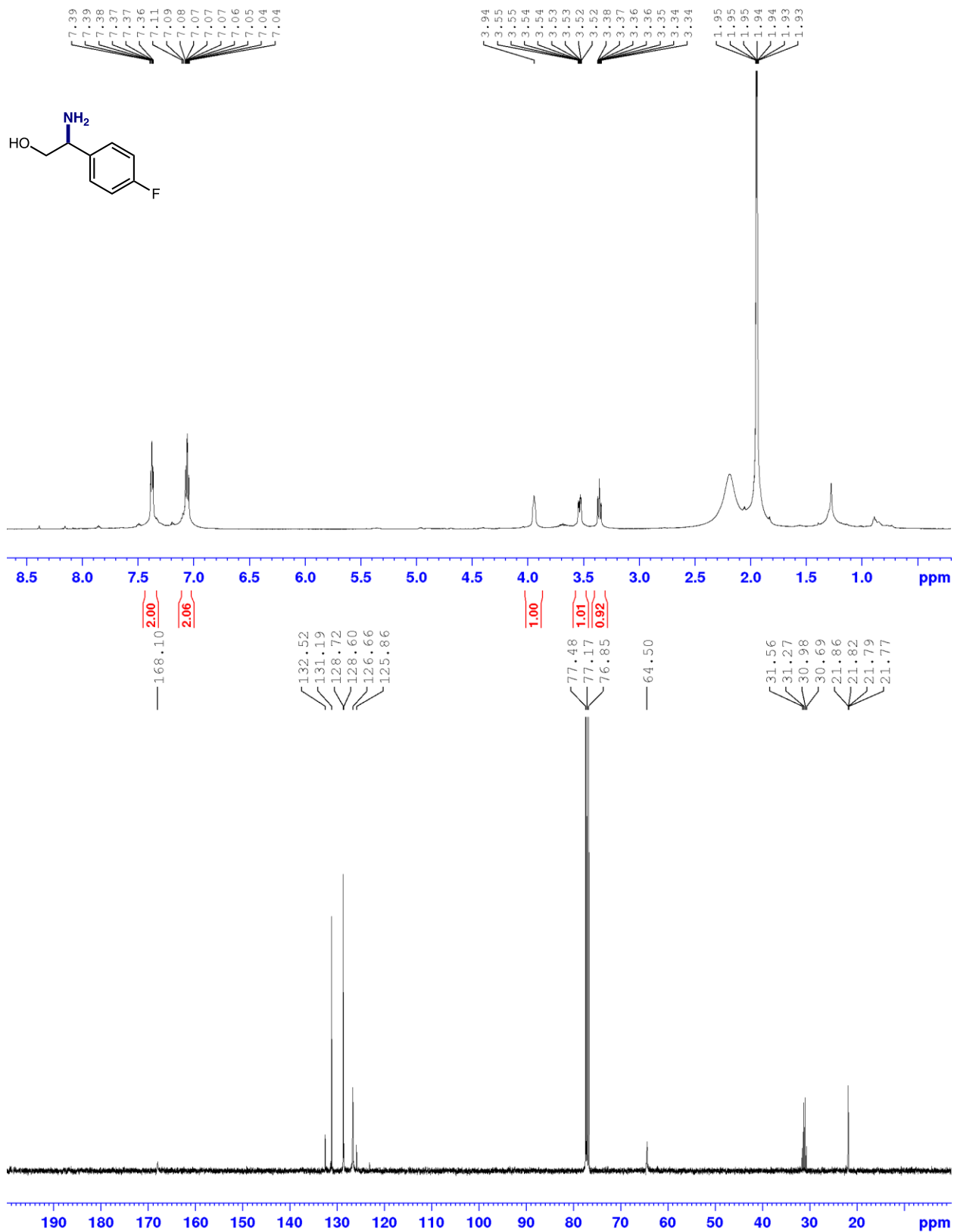

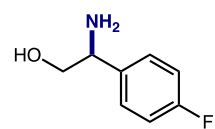

-112.86  
-112.85  
-112.84  
-112.83  
-112.82  
-112.82

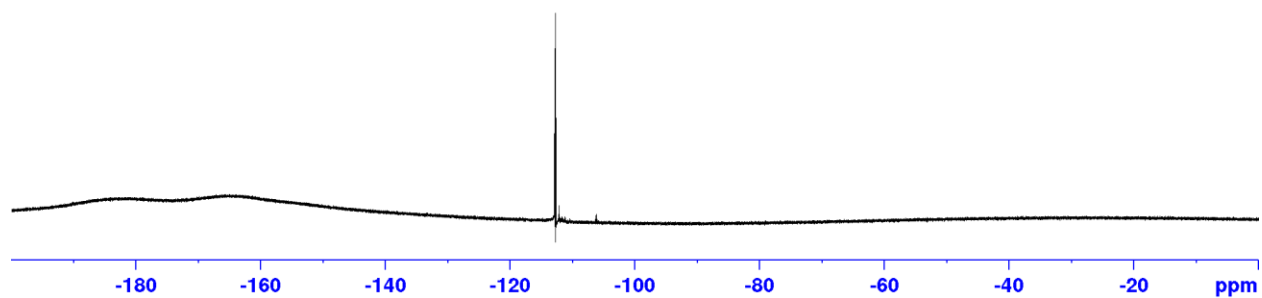

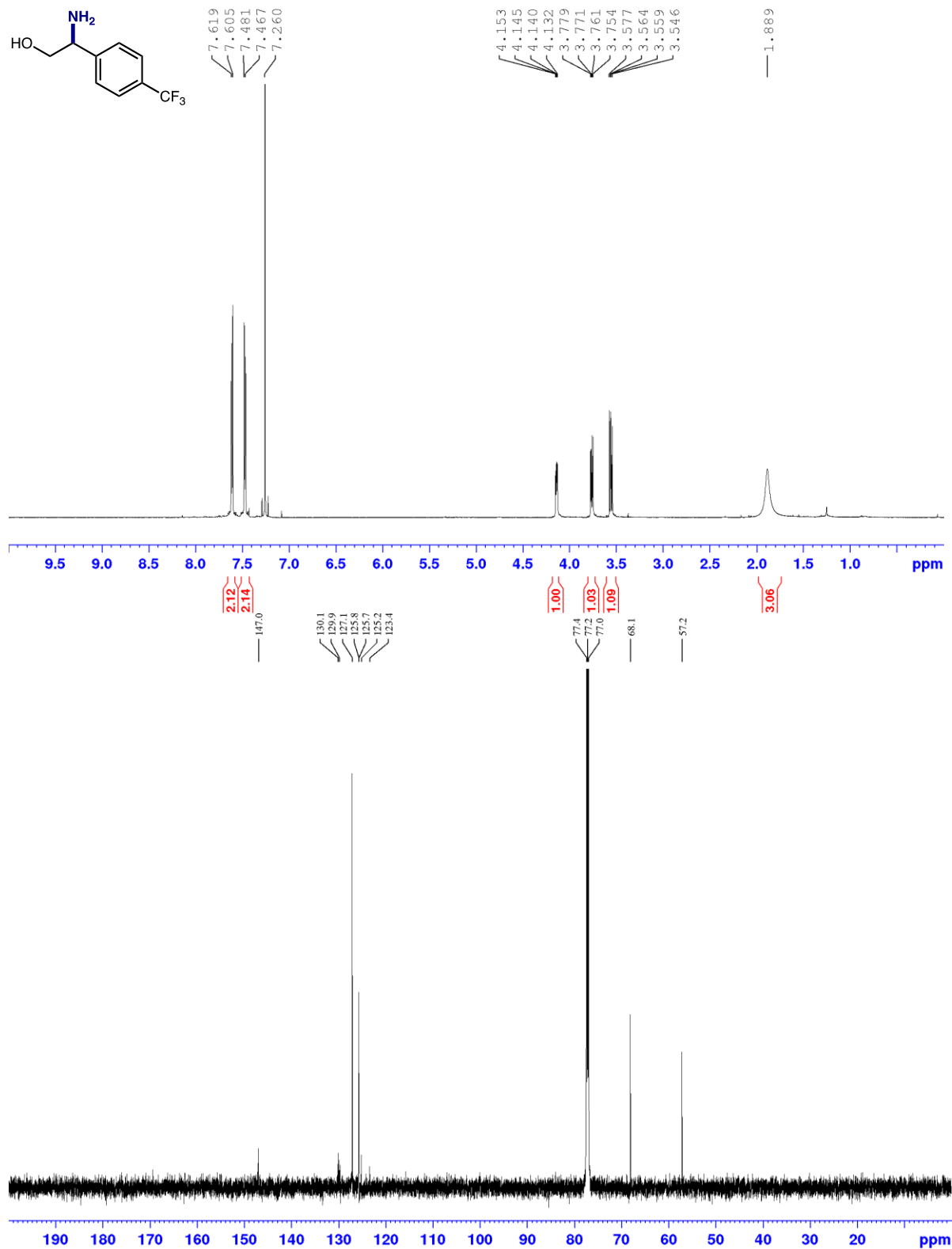

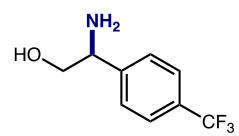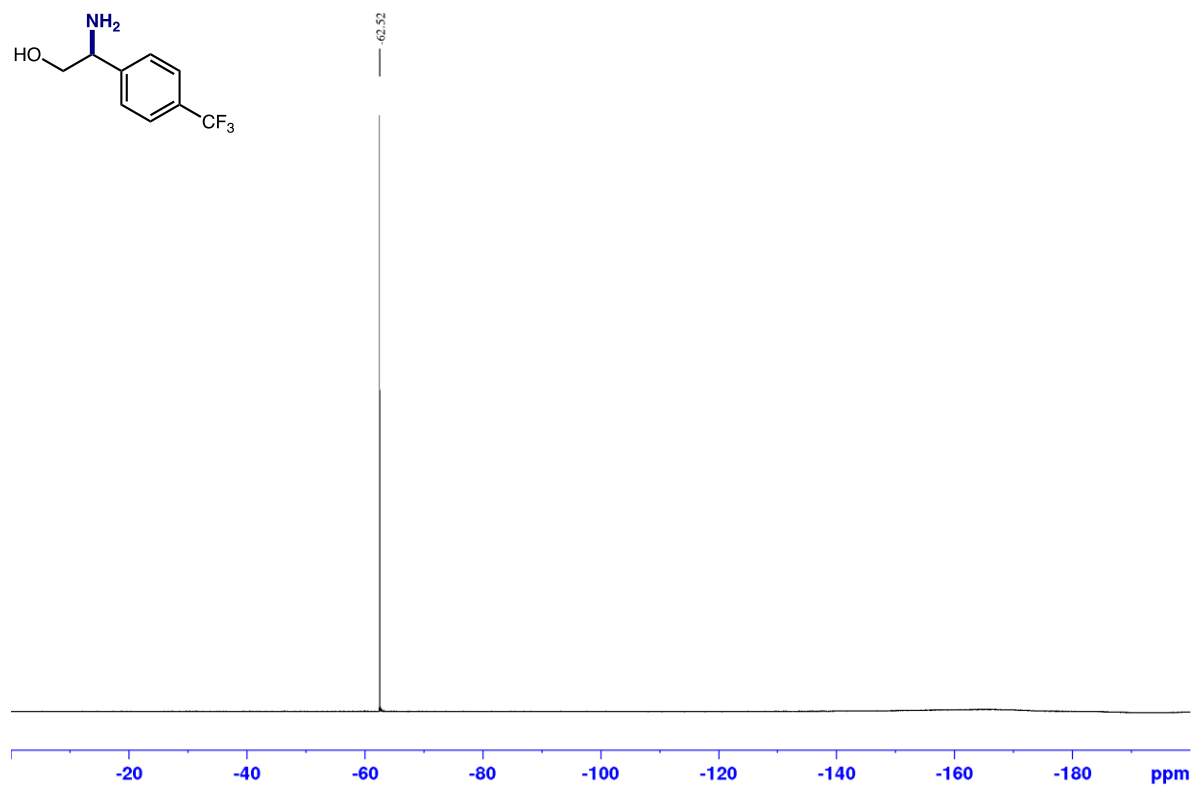

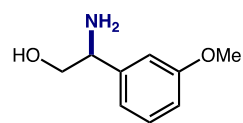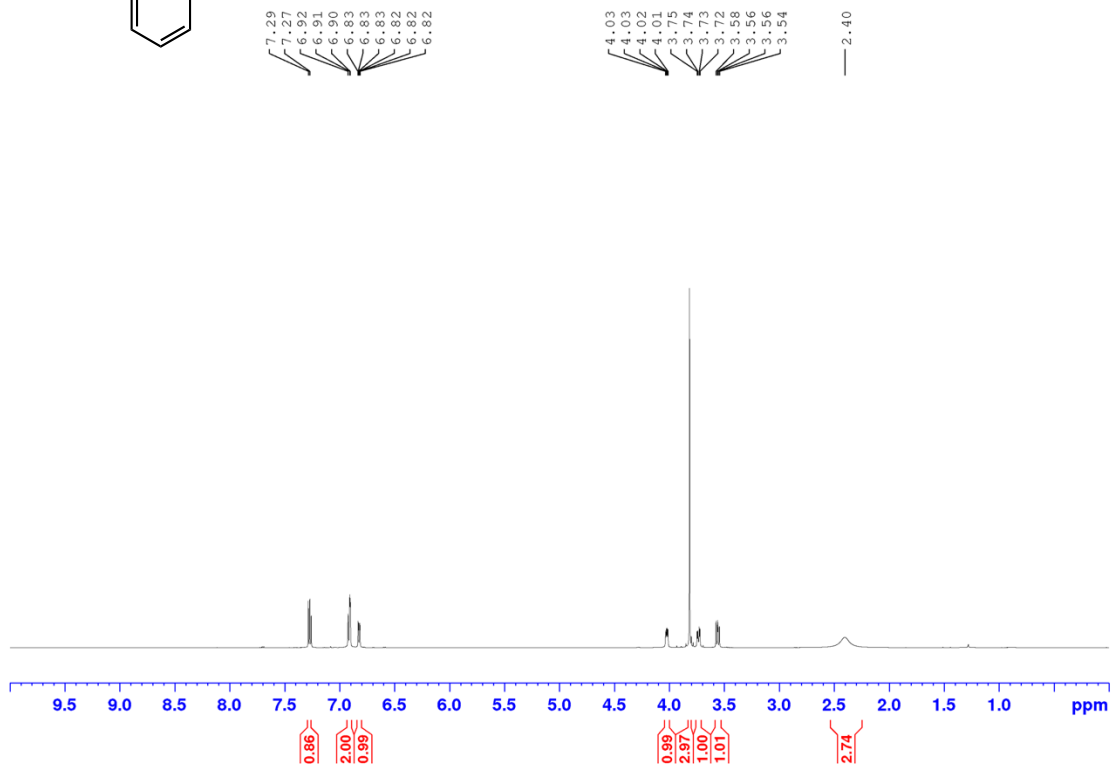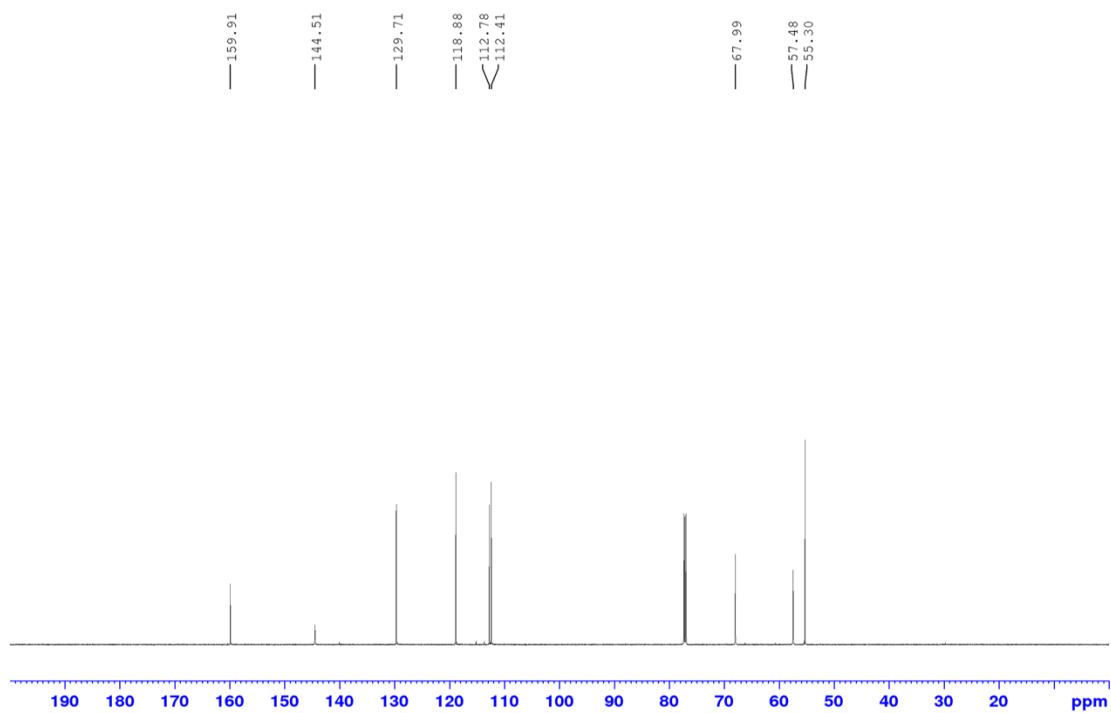

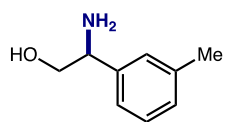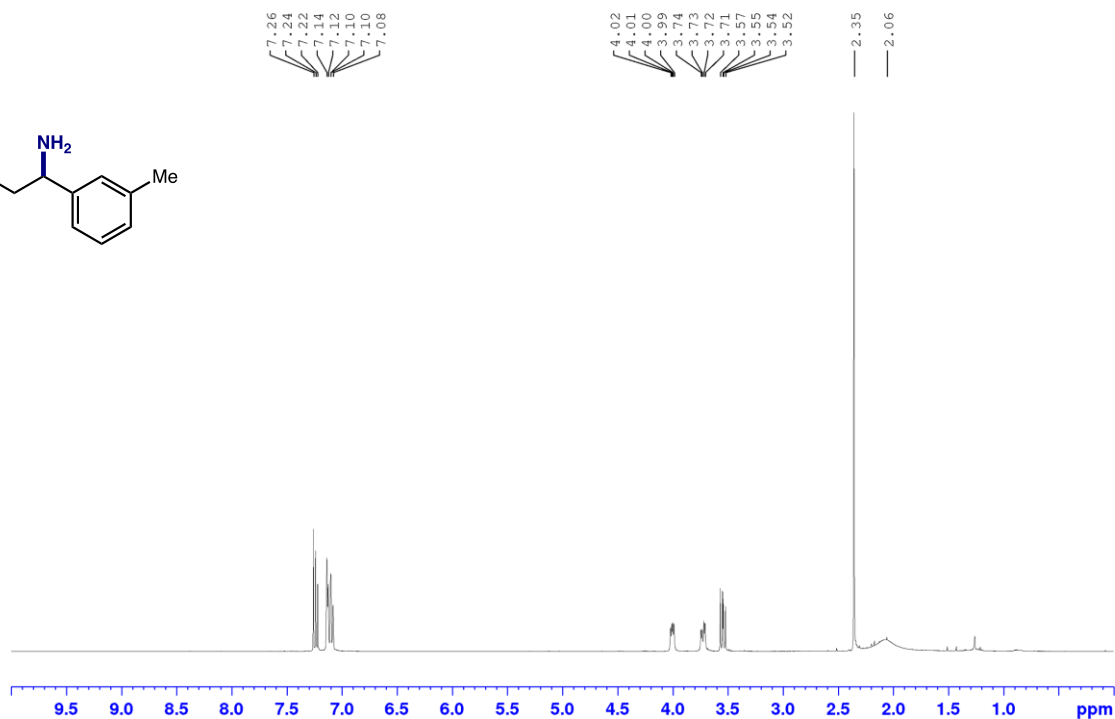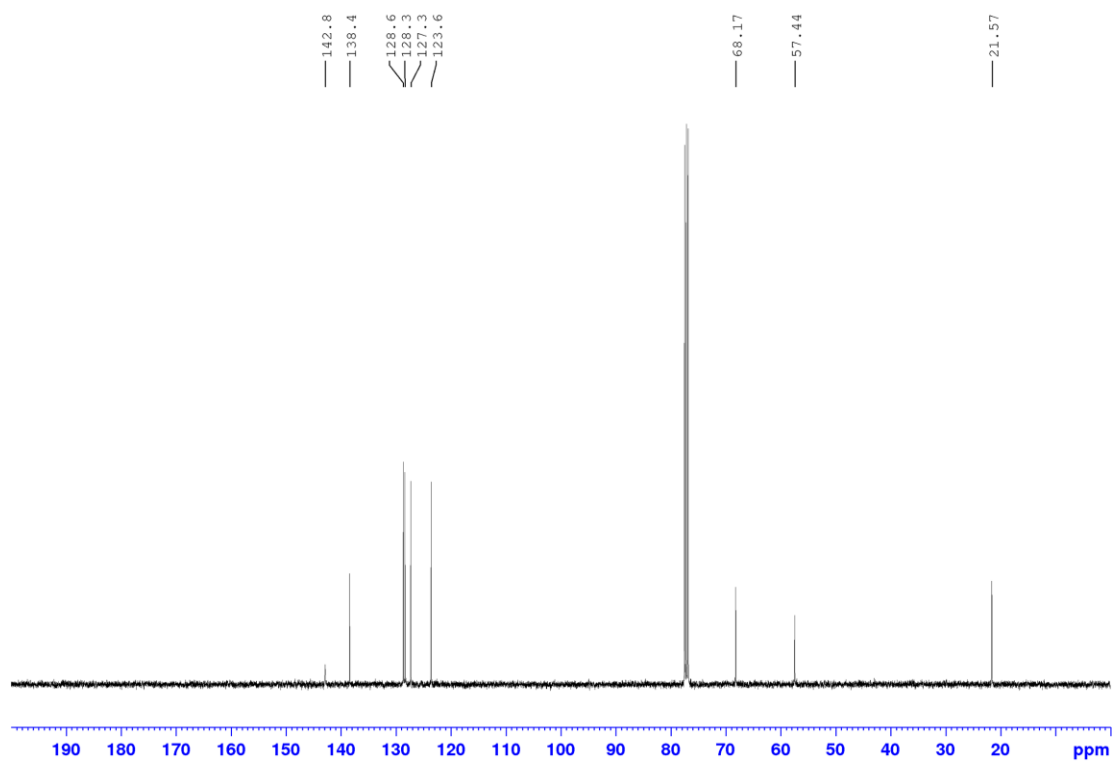

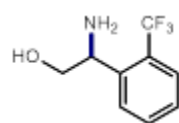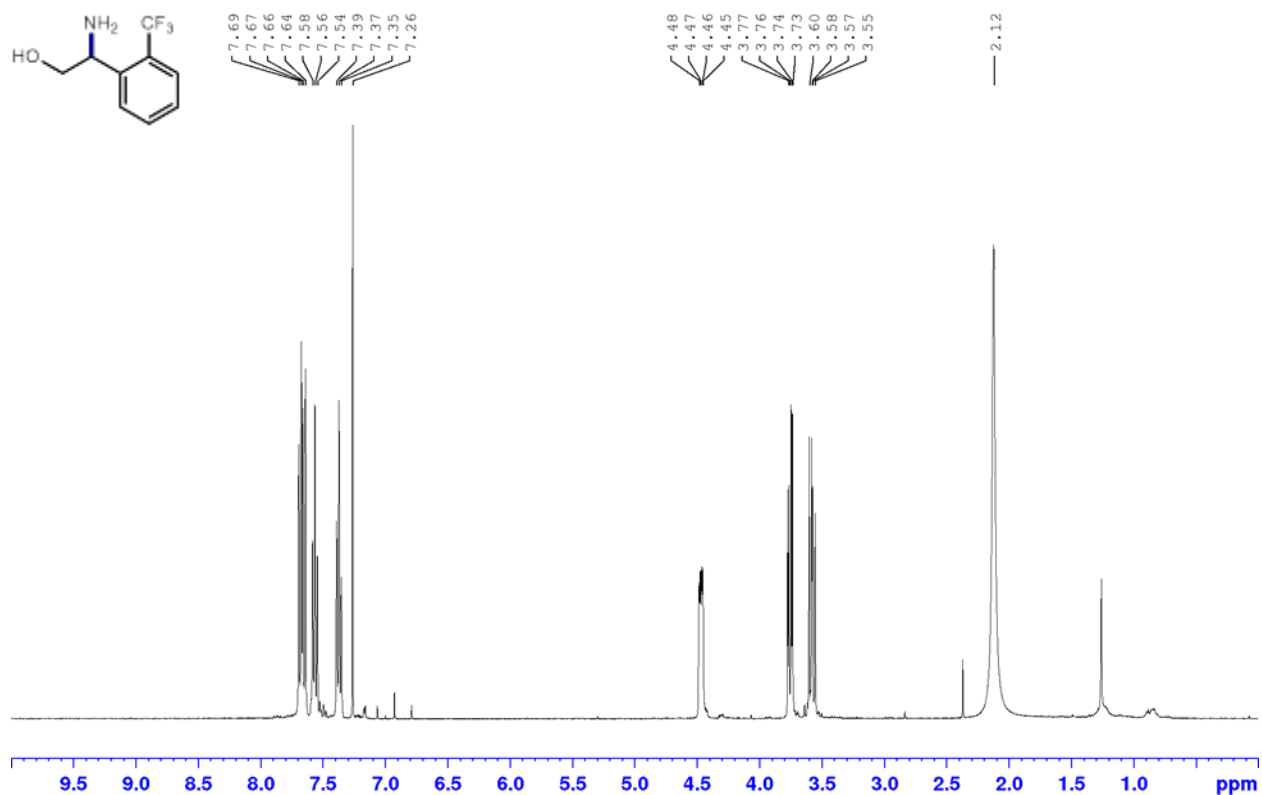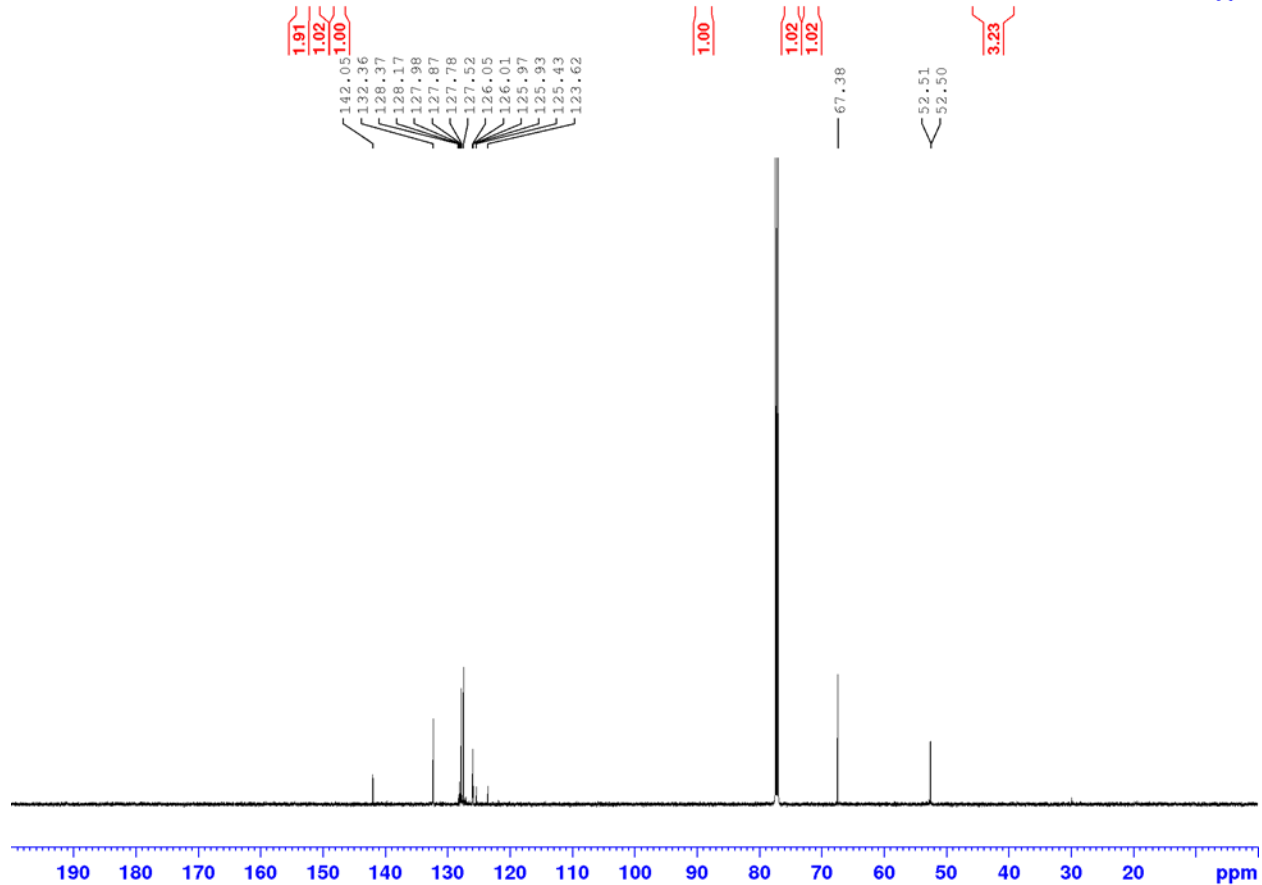

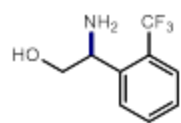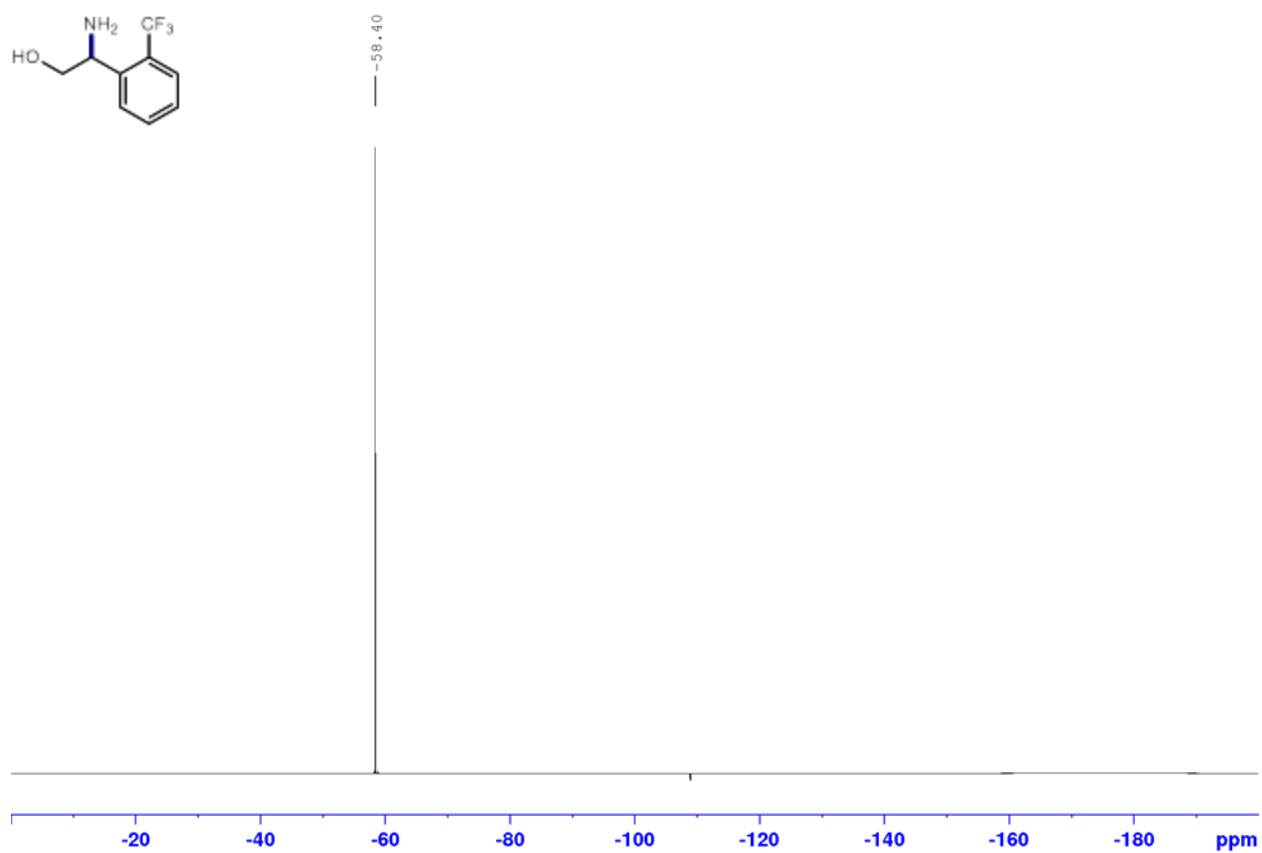

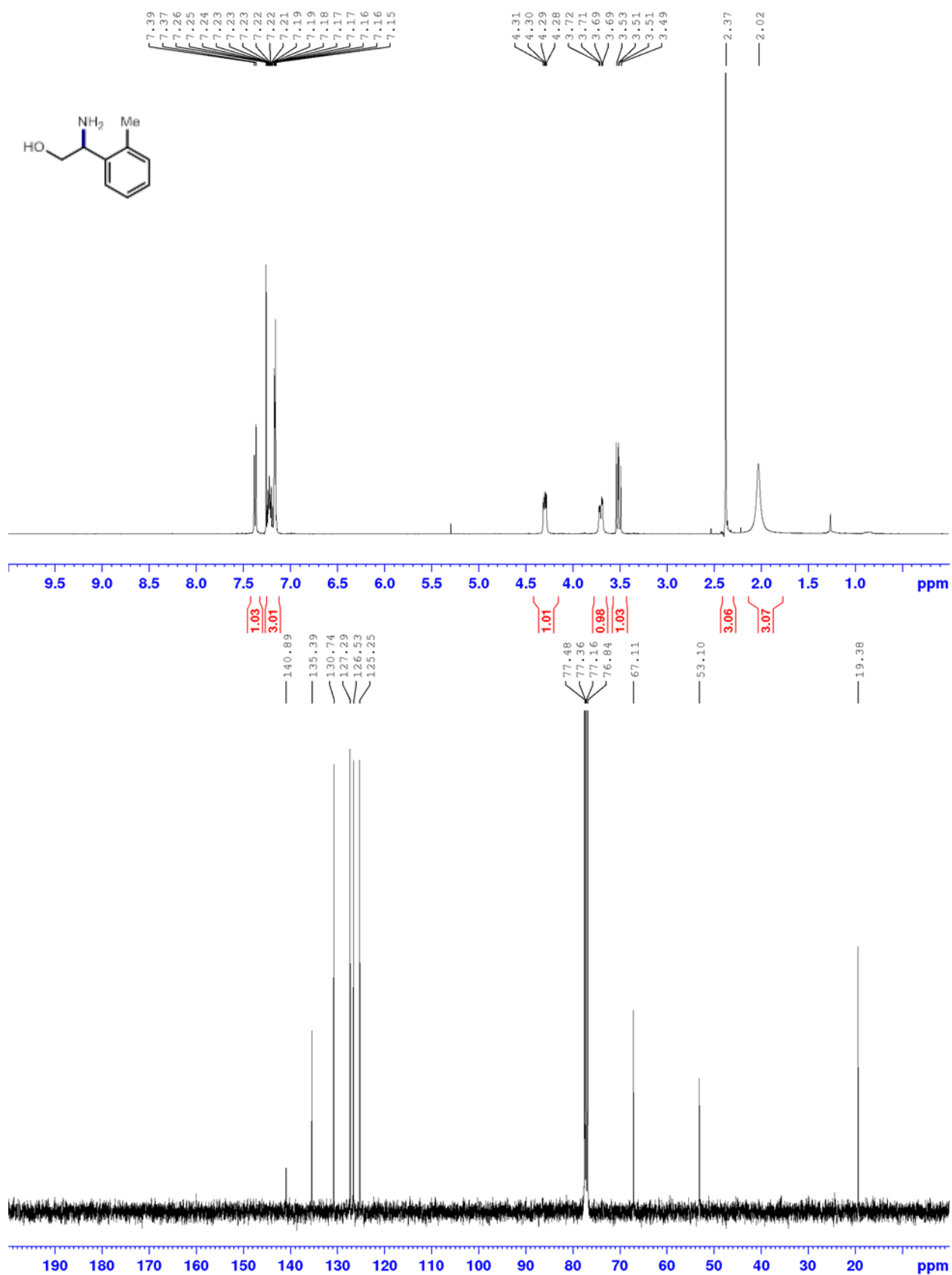

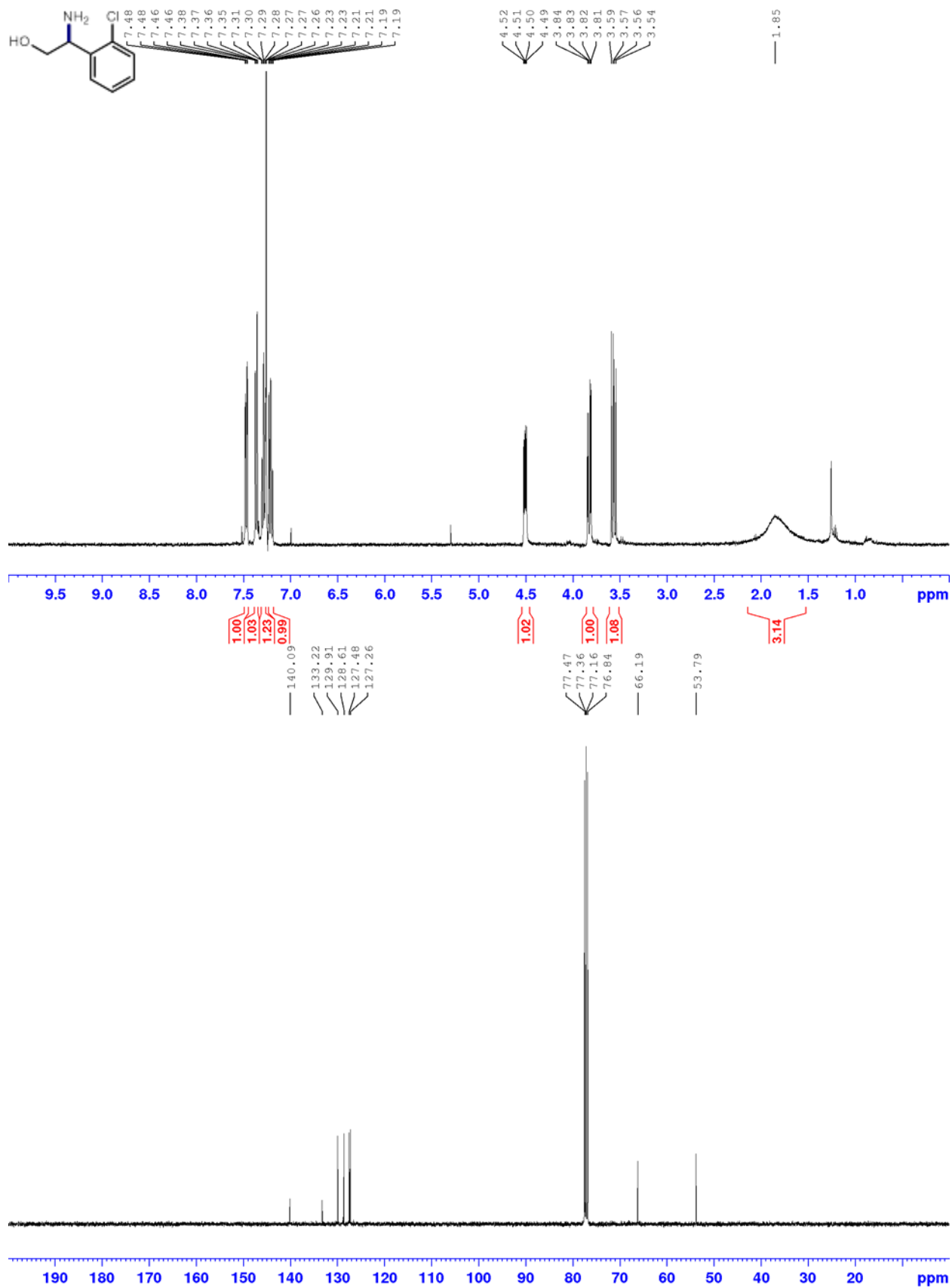

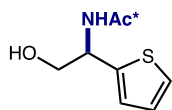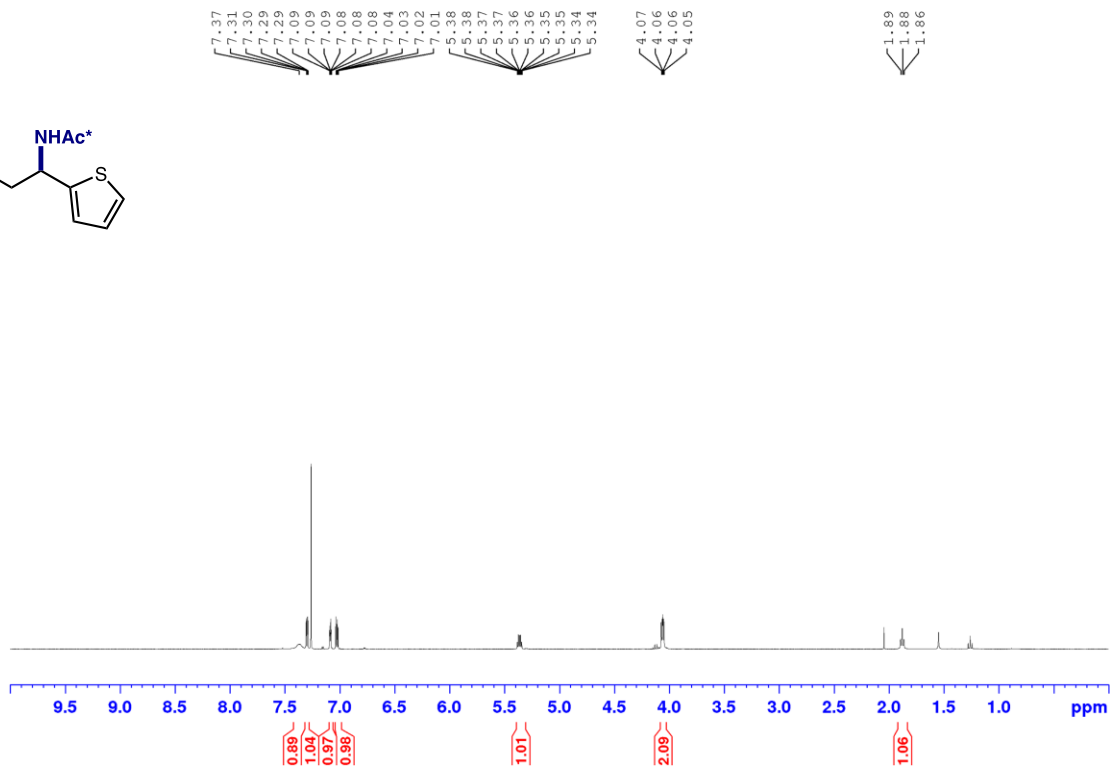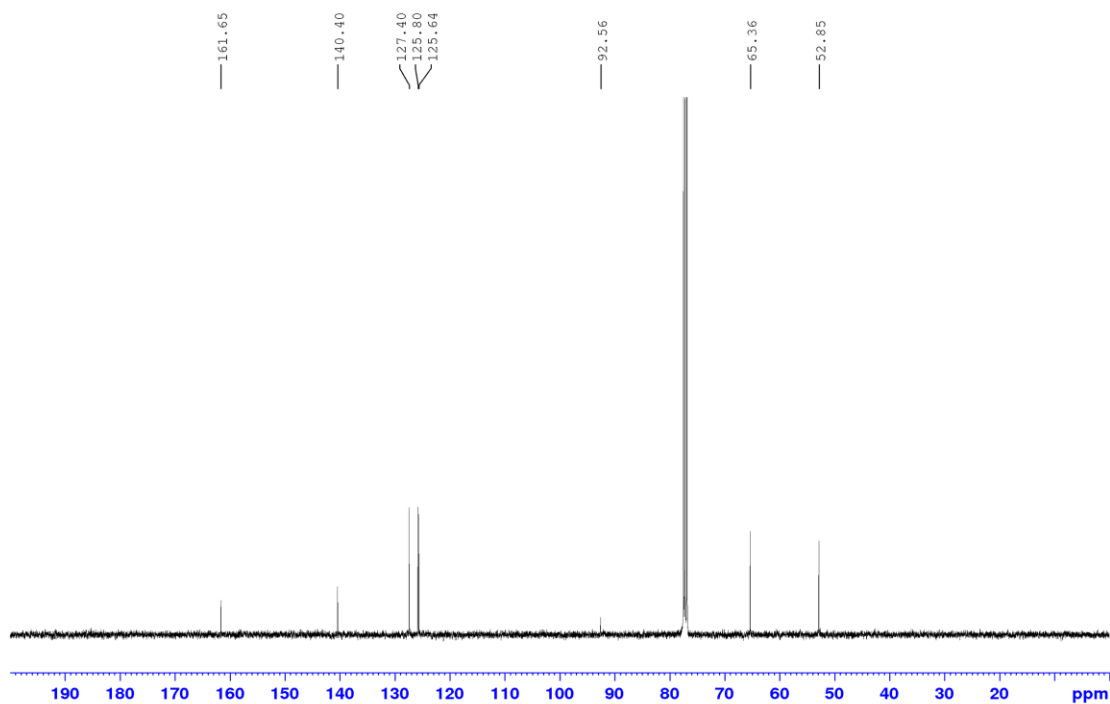

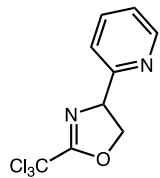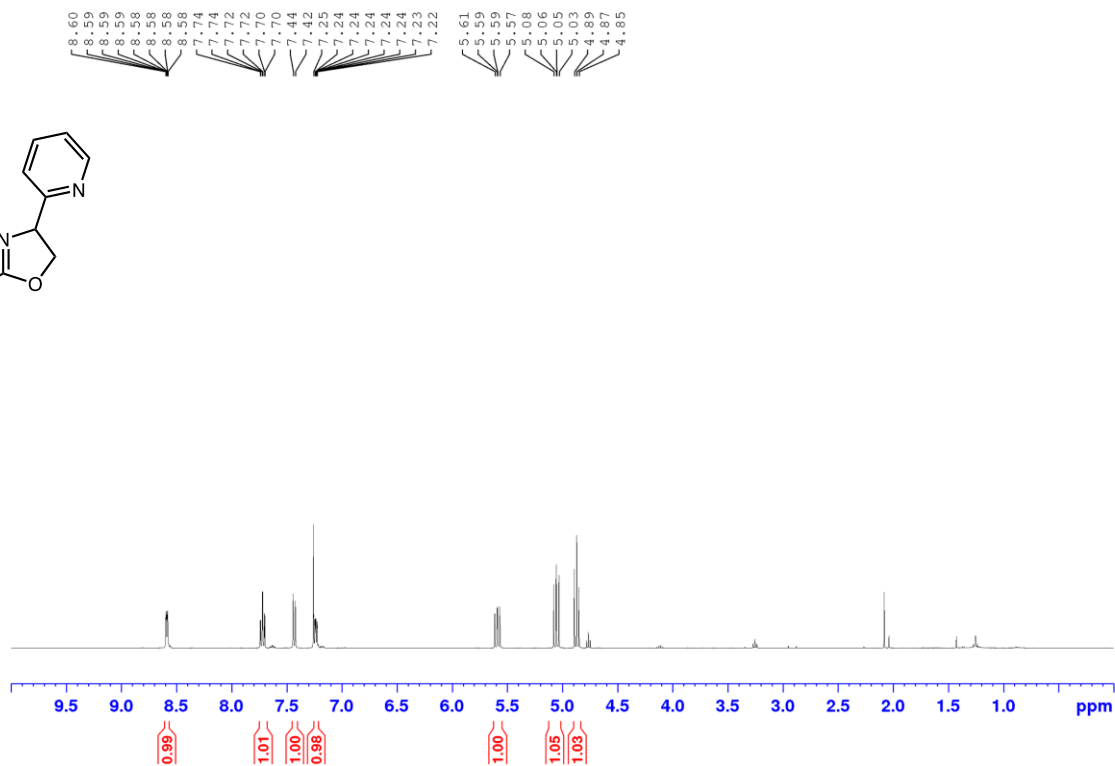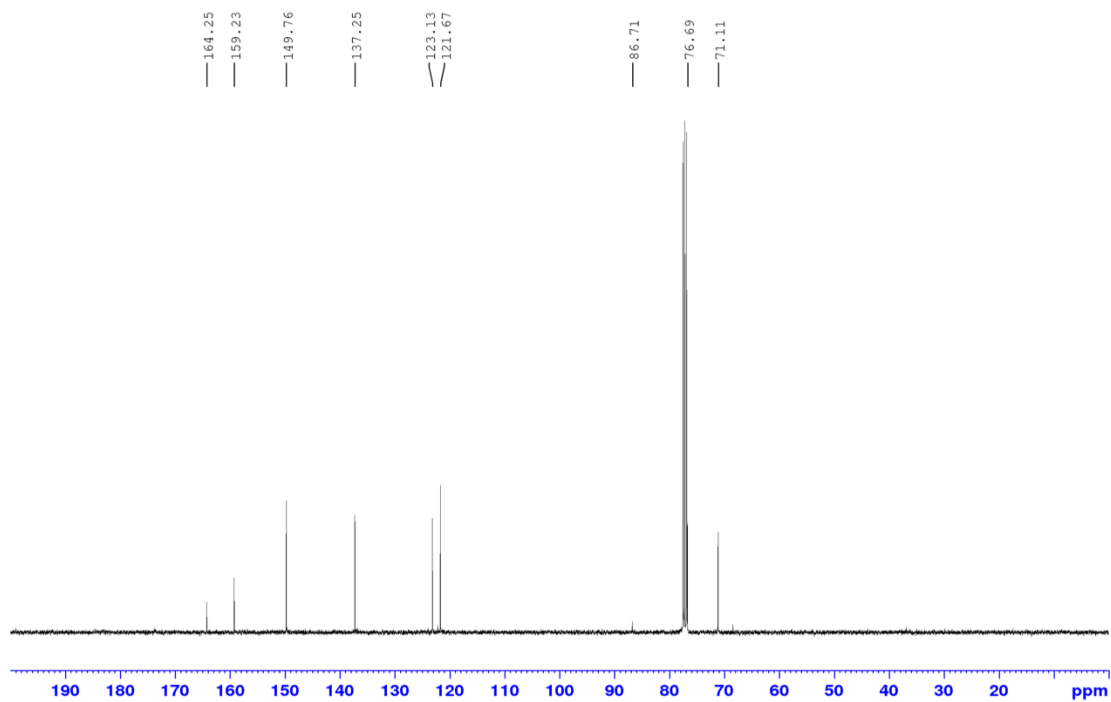

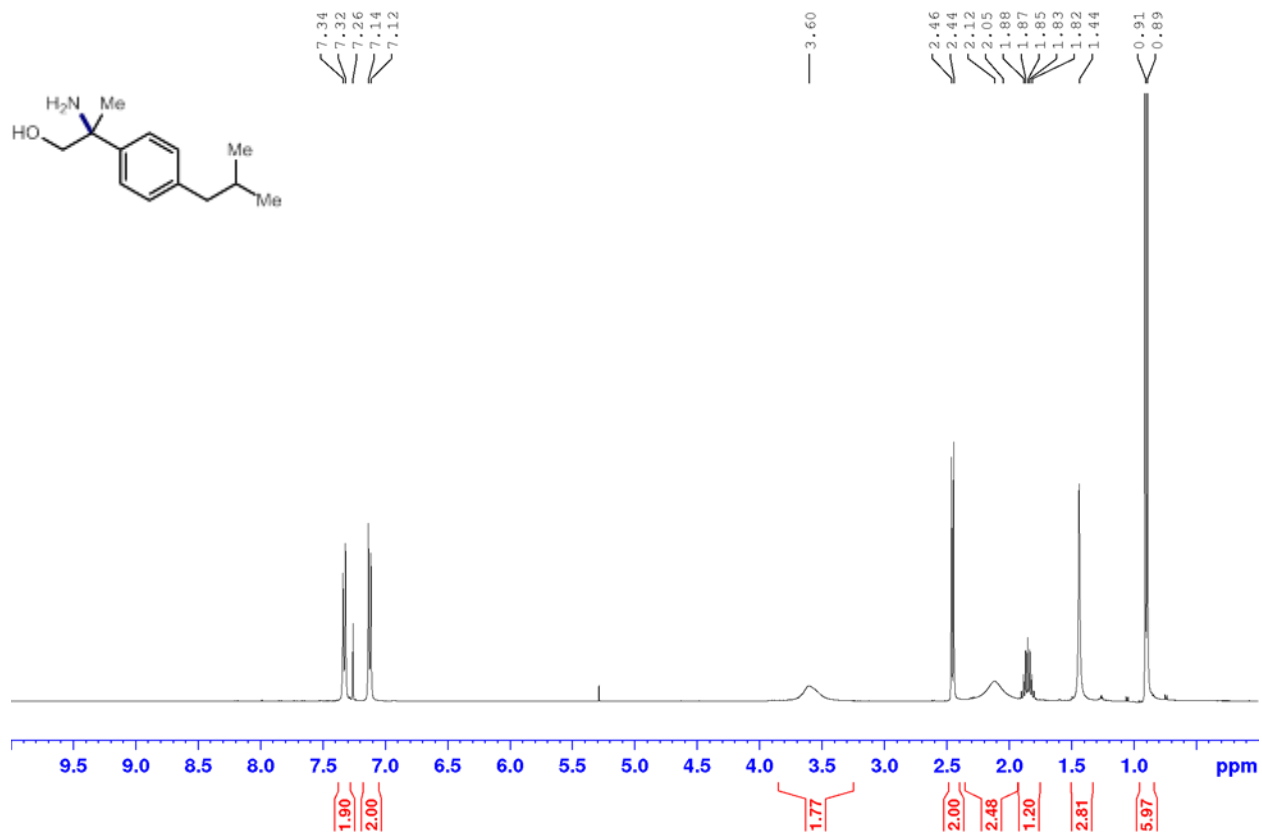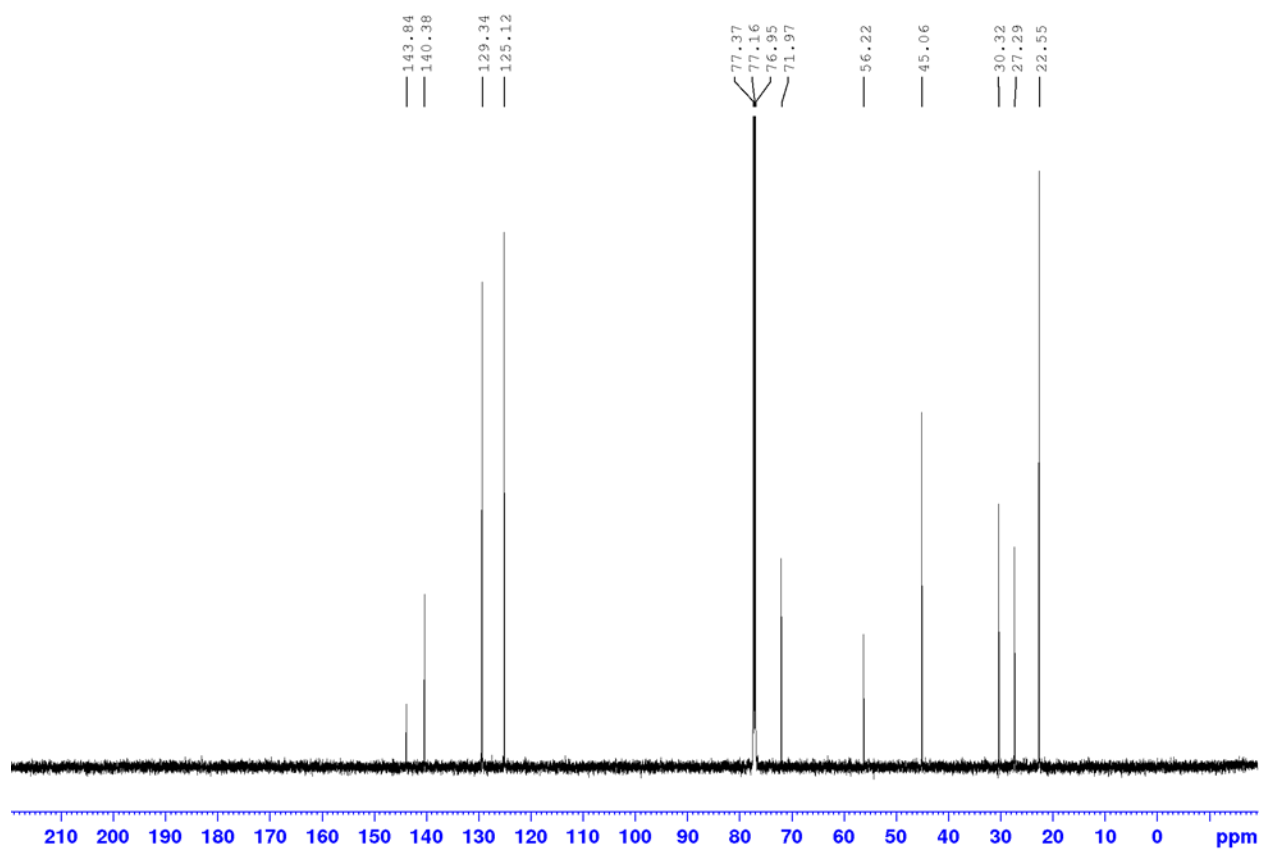

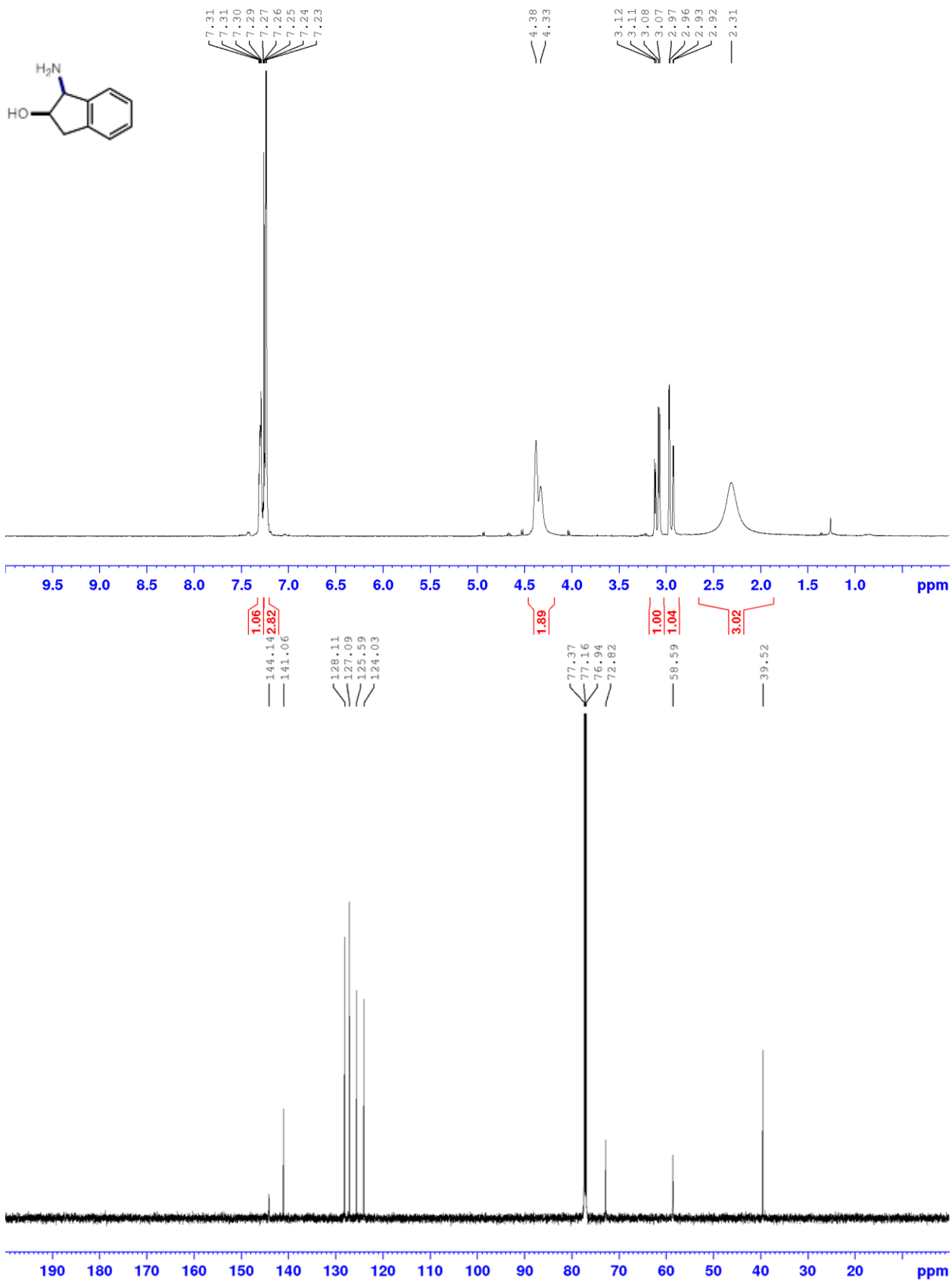

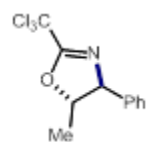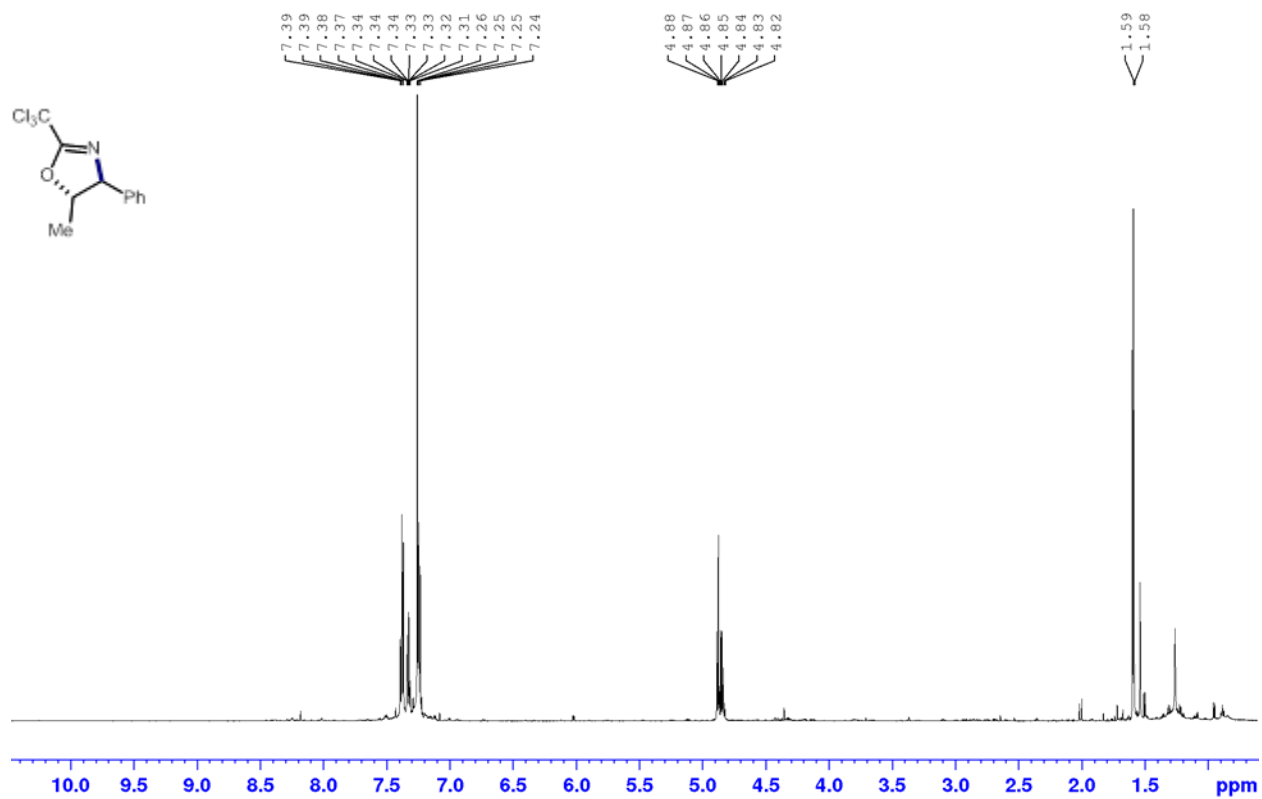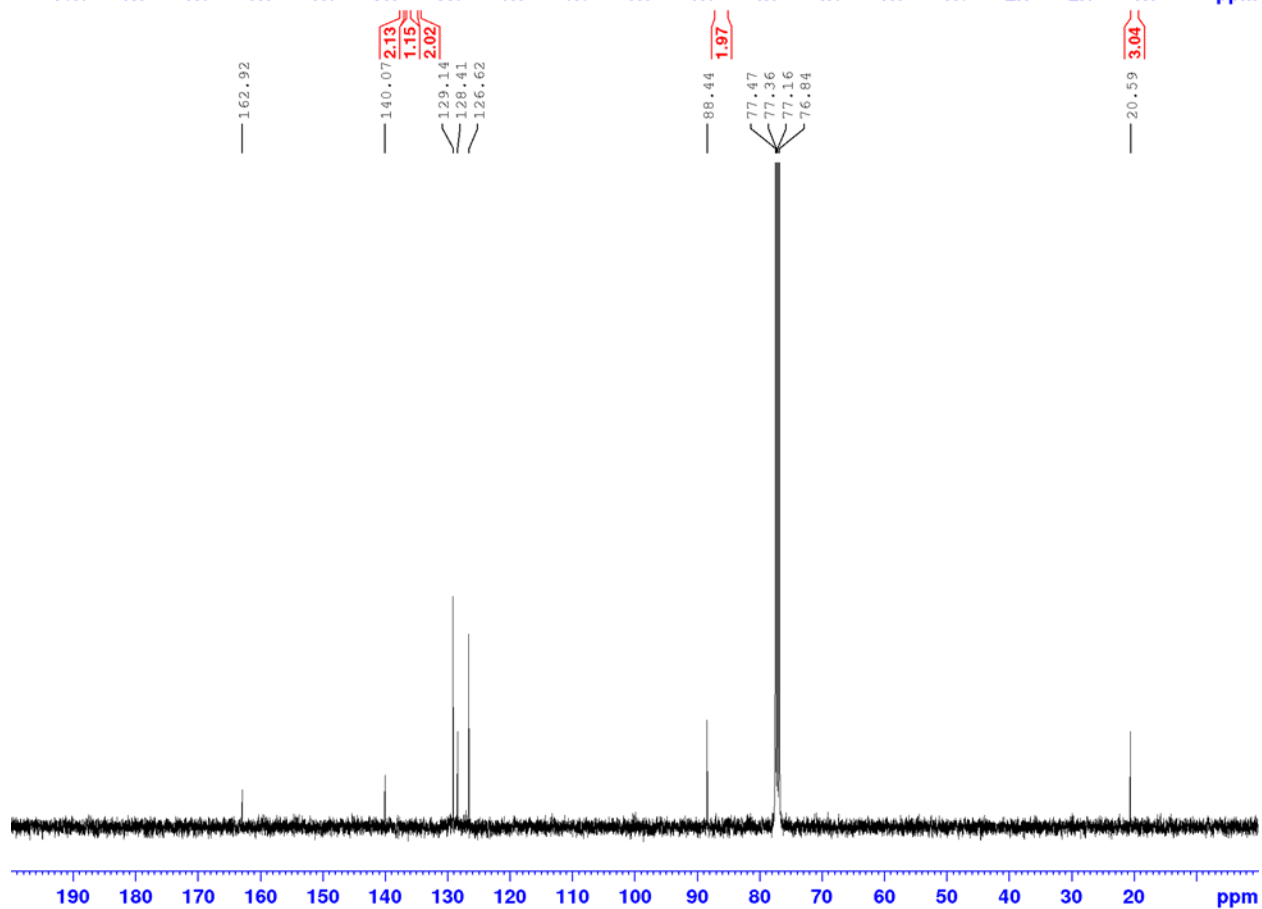

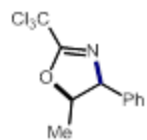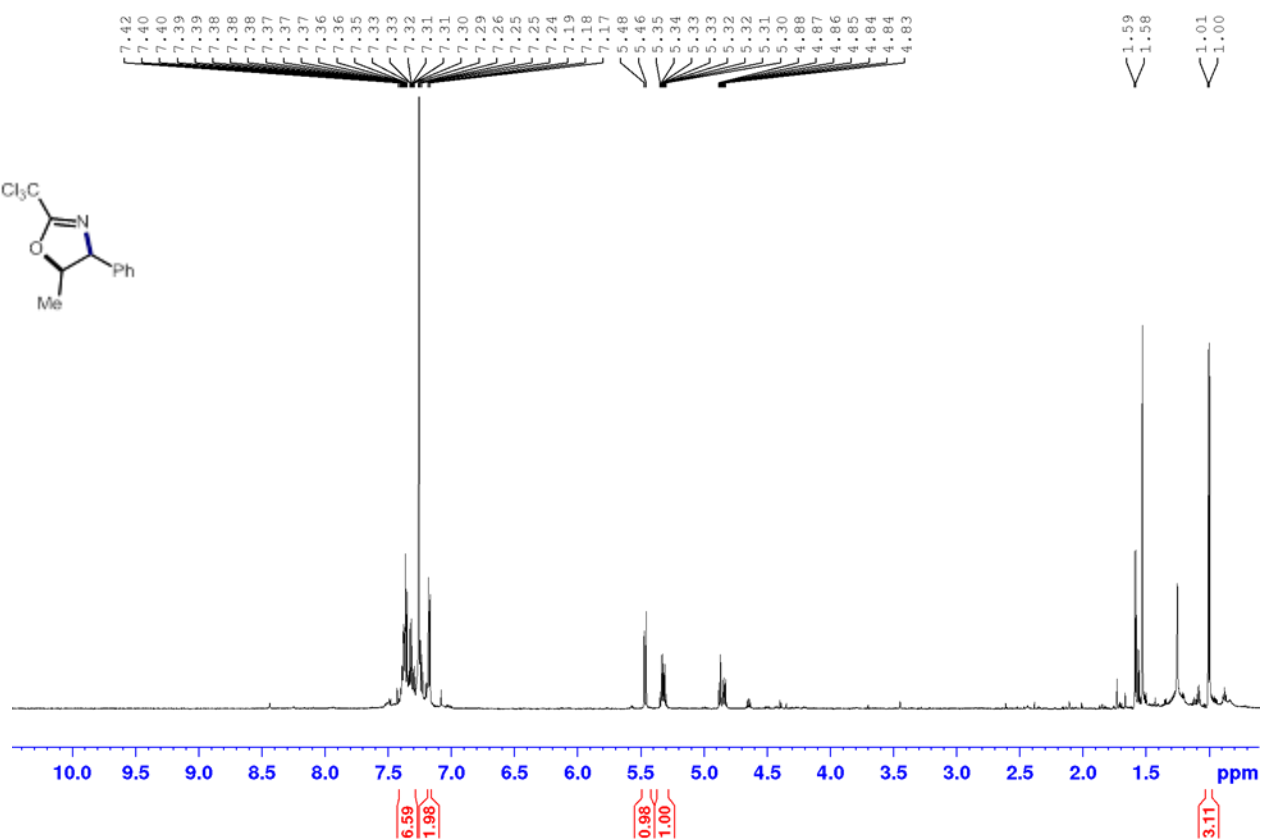

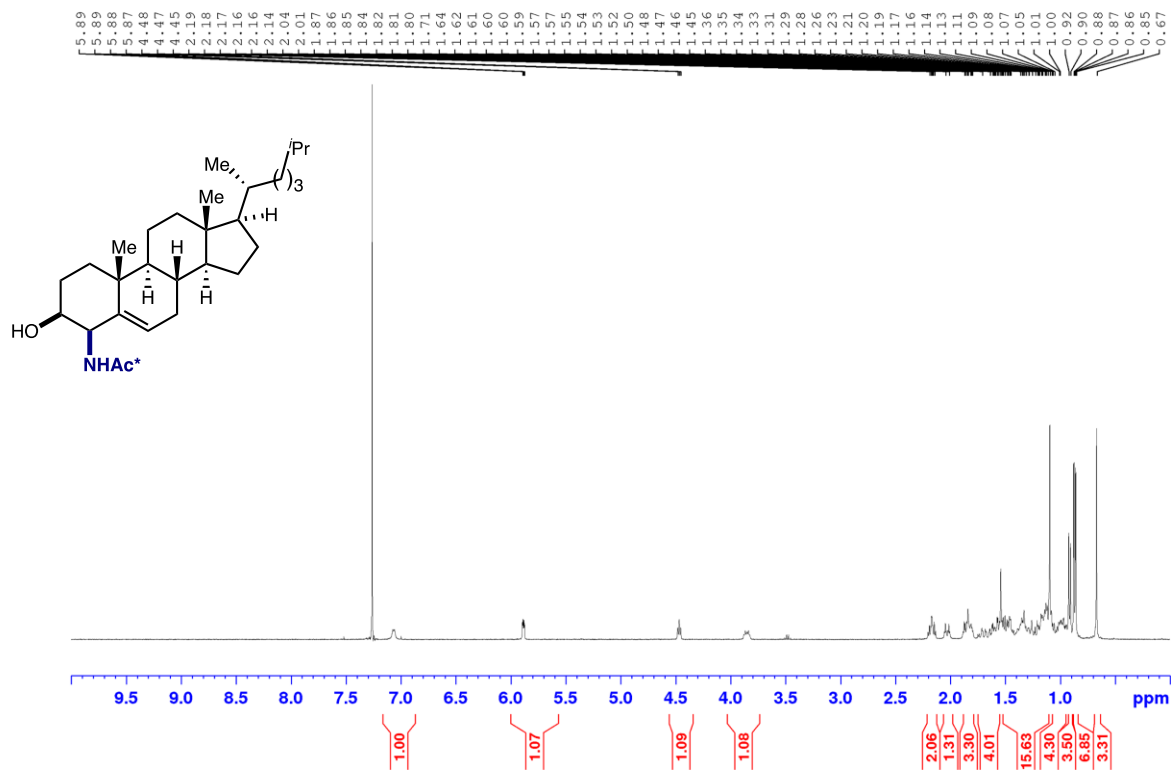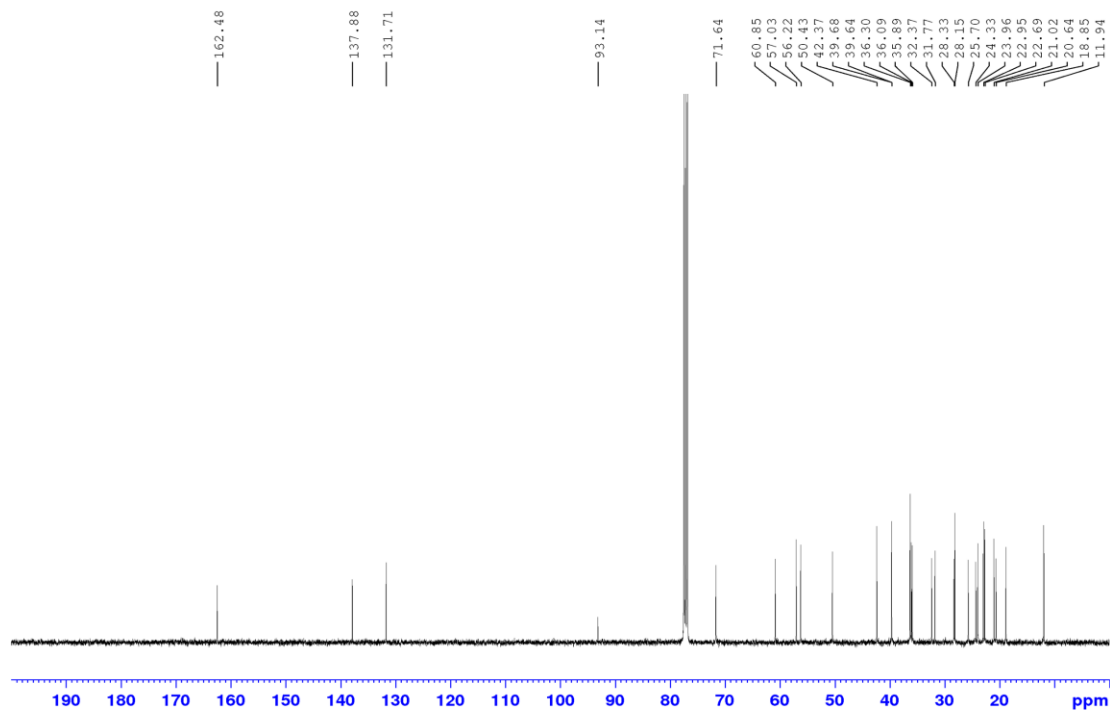

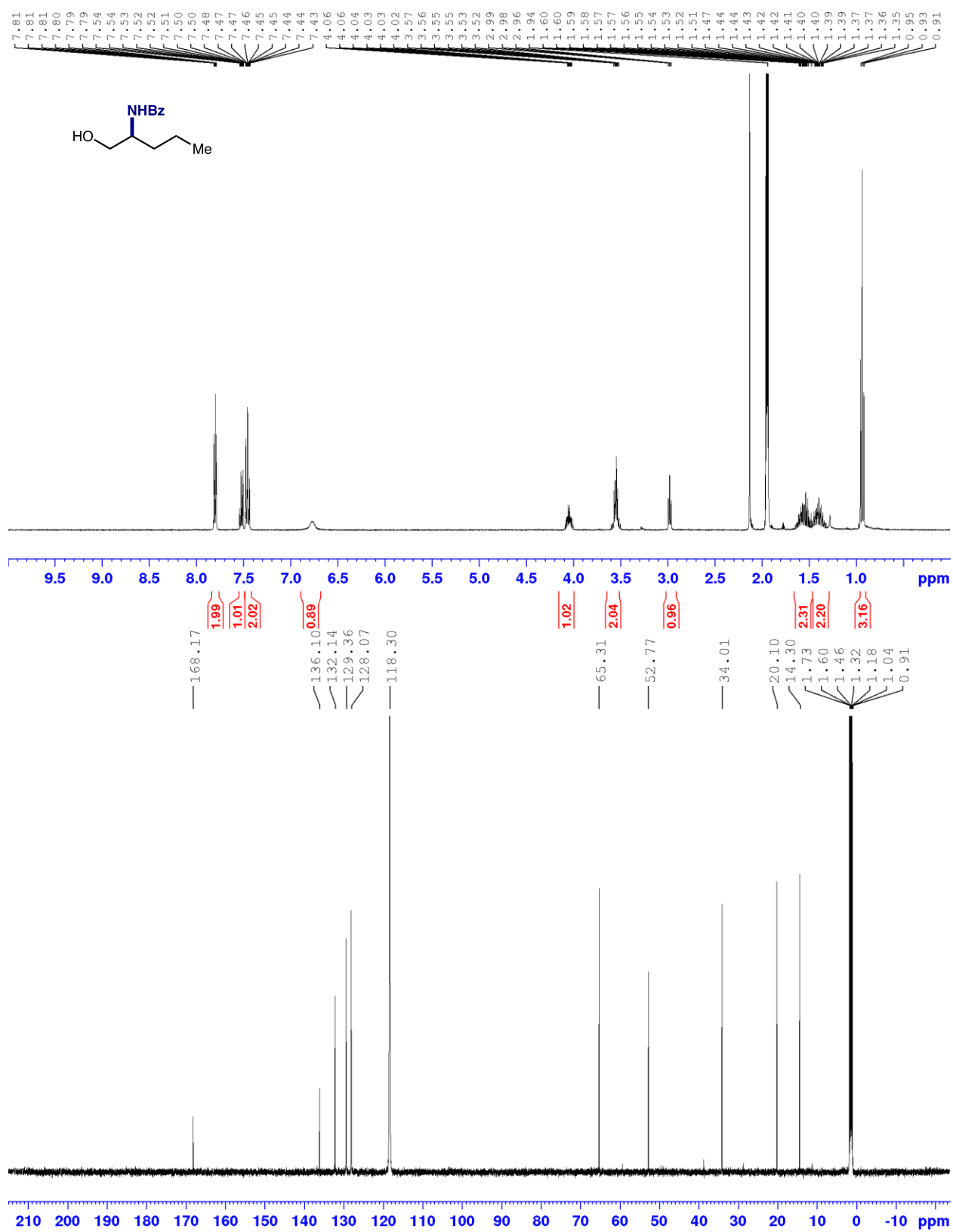

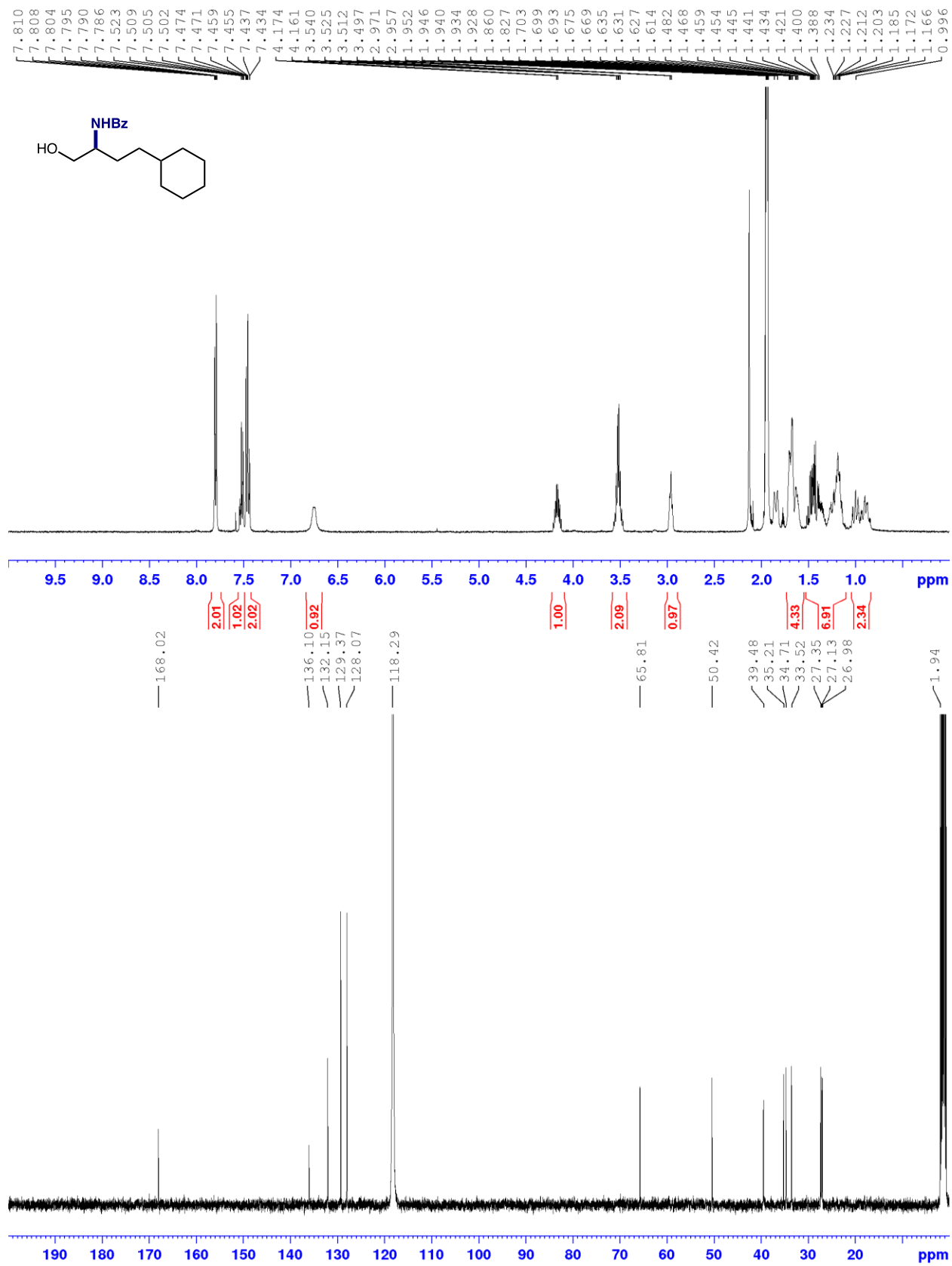

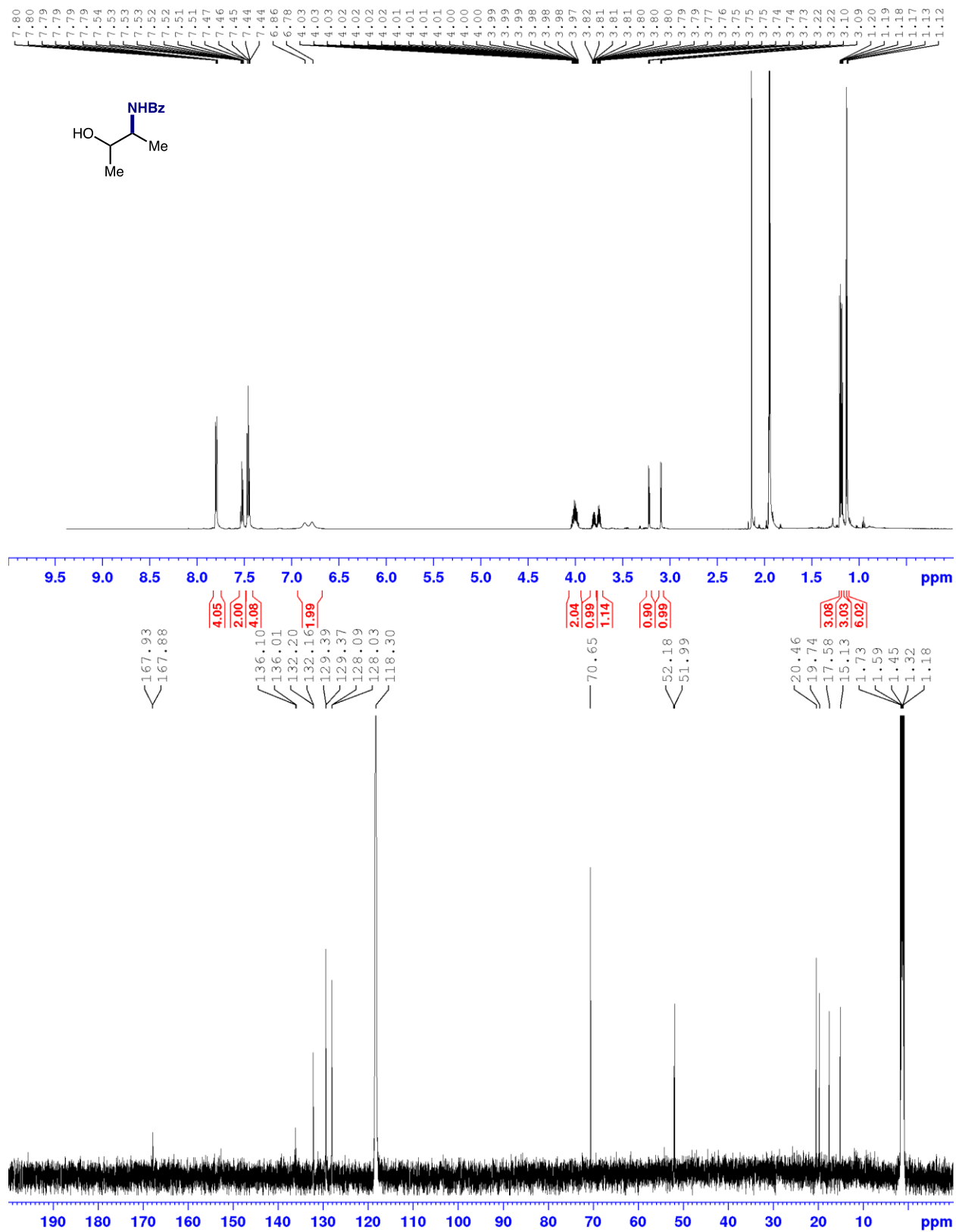

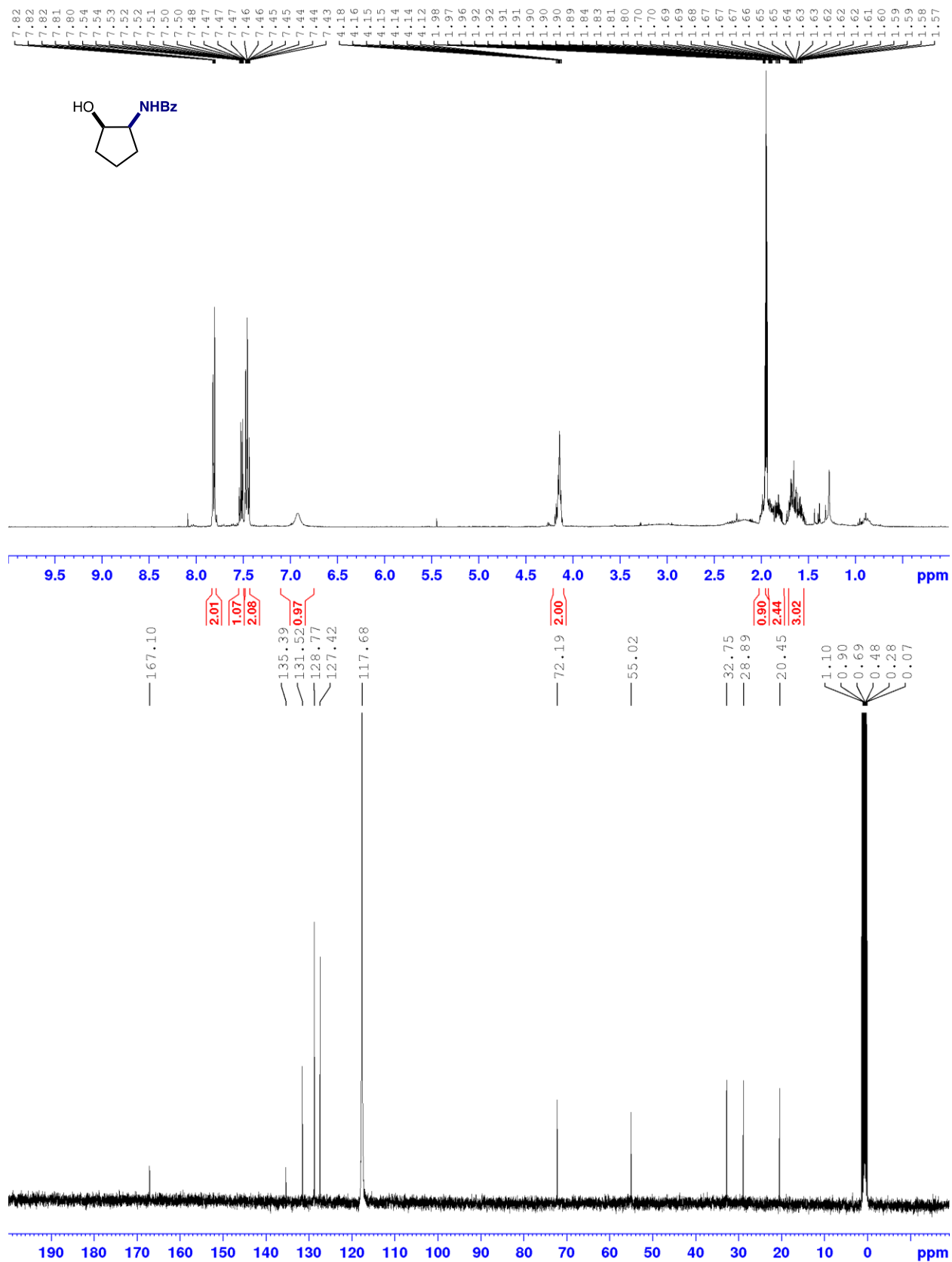

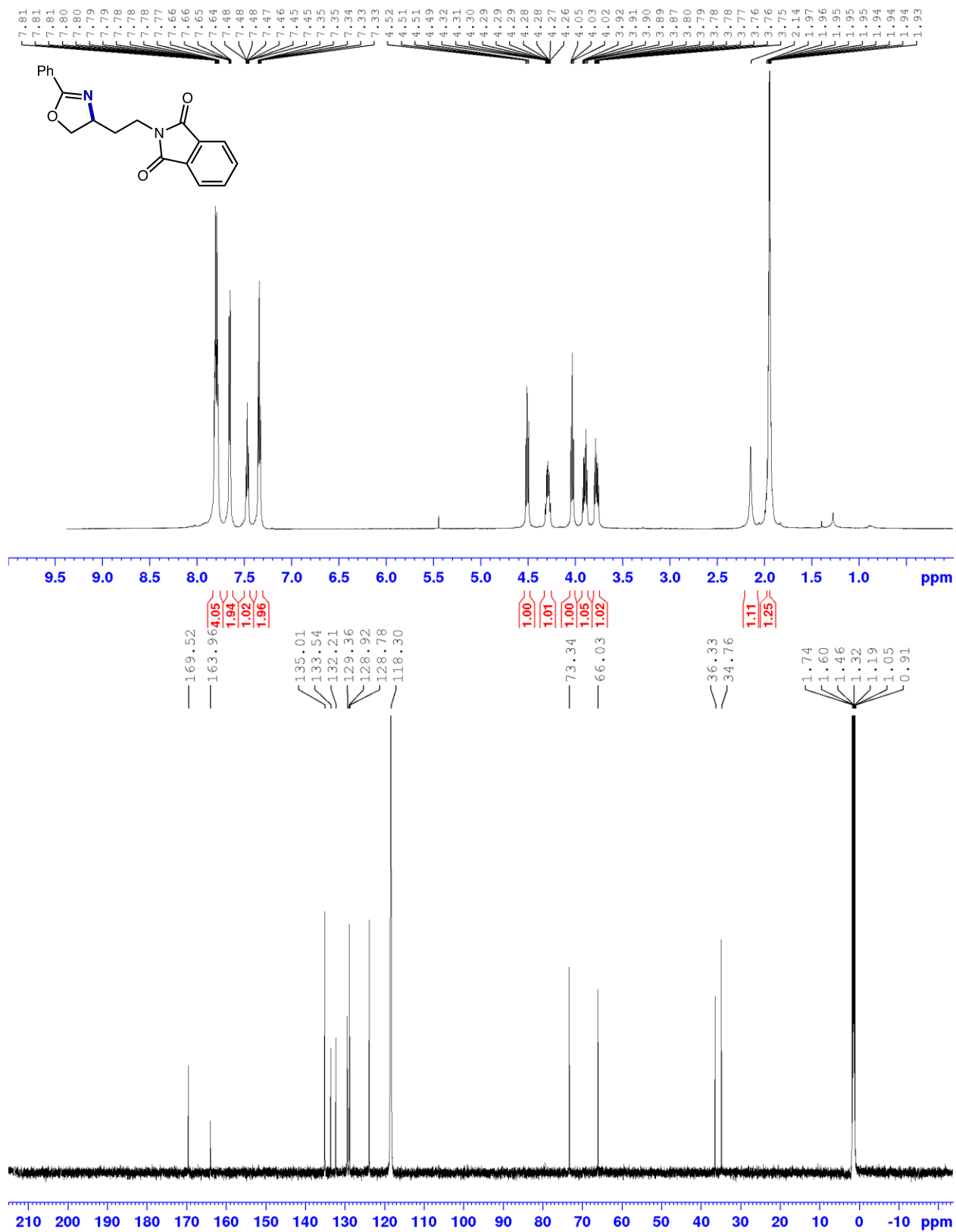

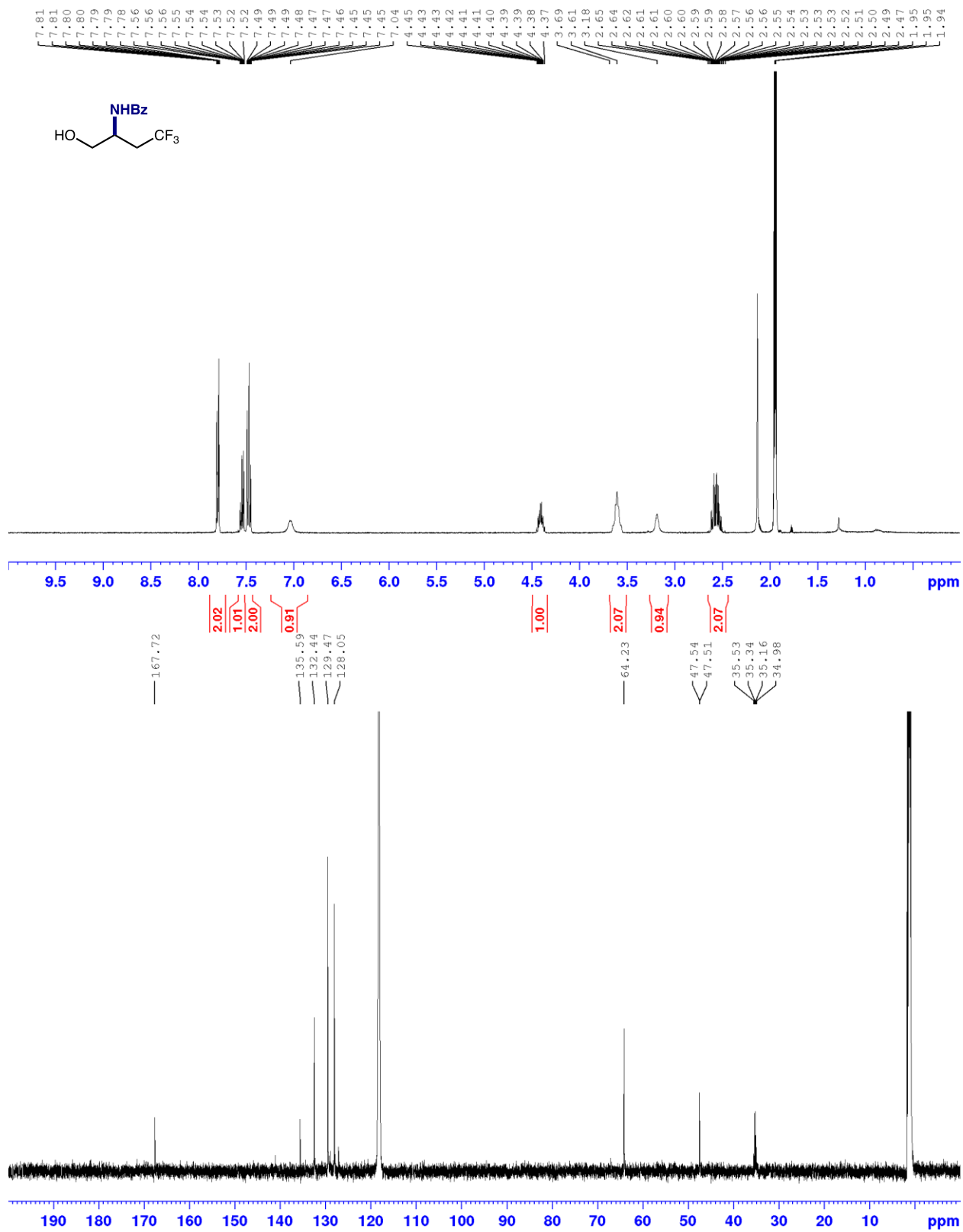

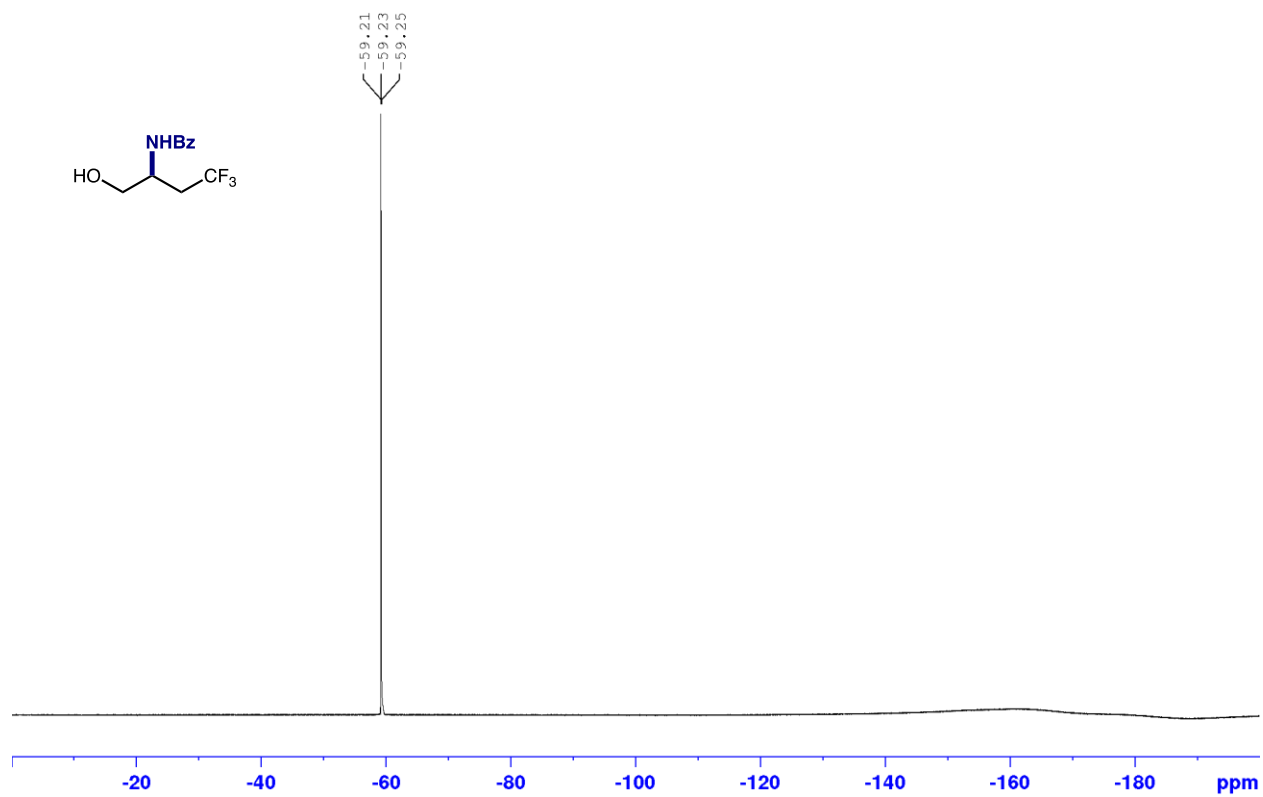

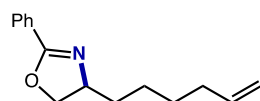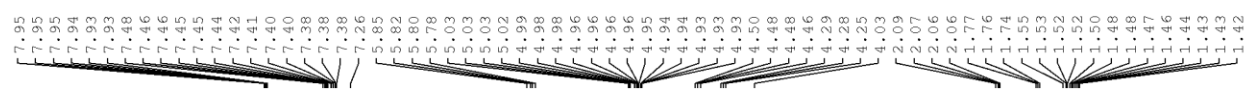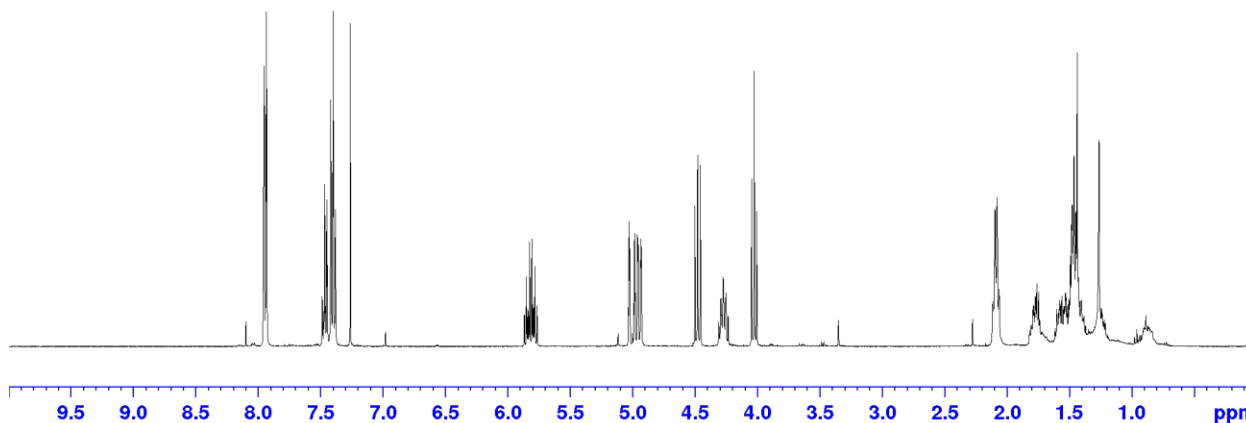

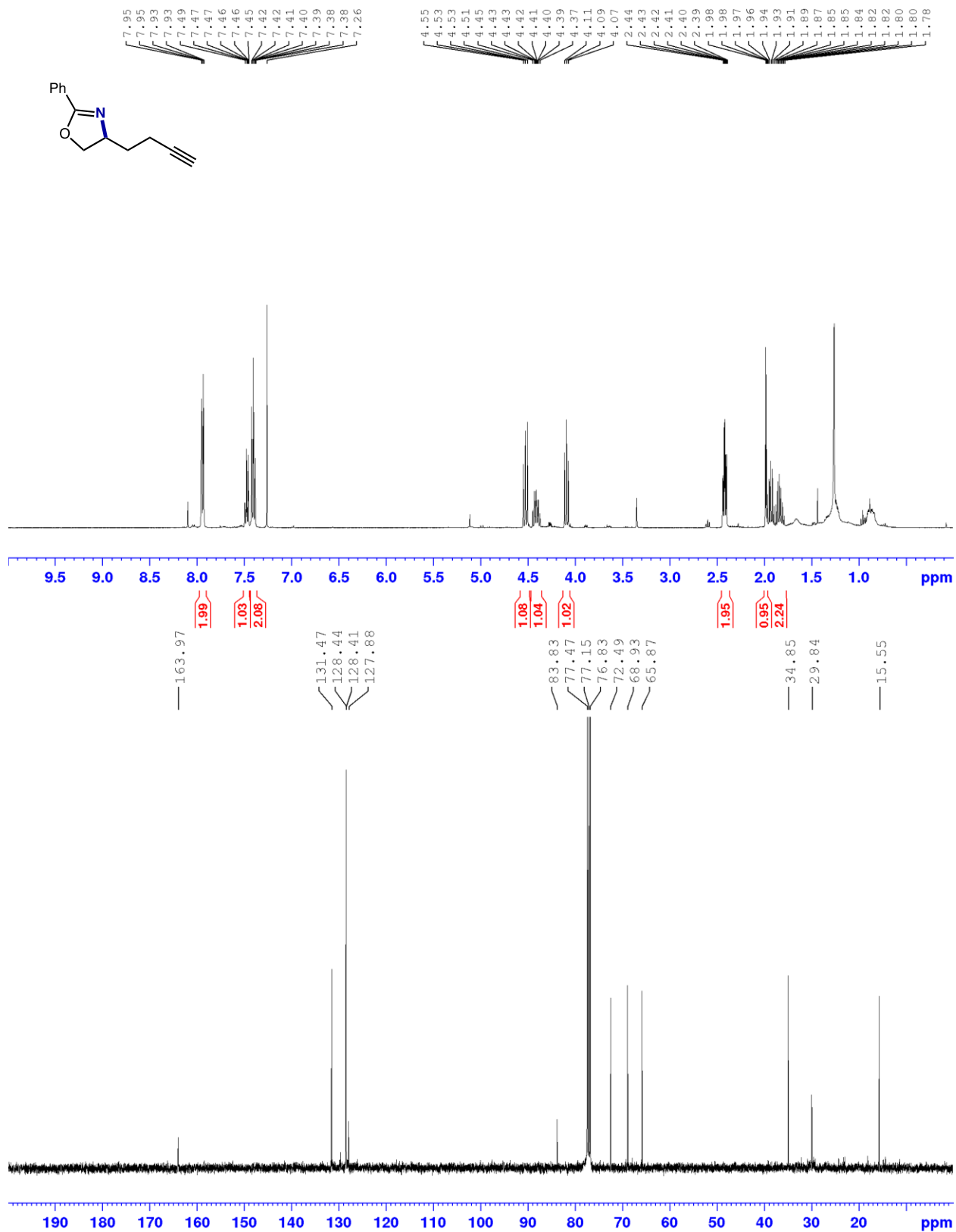

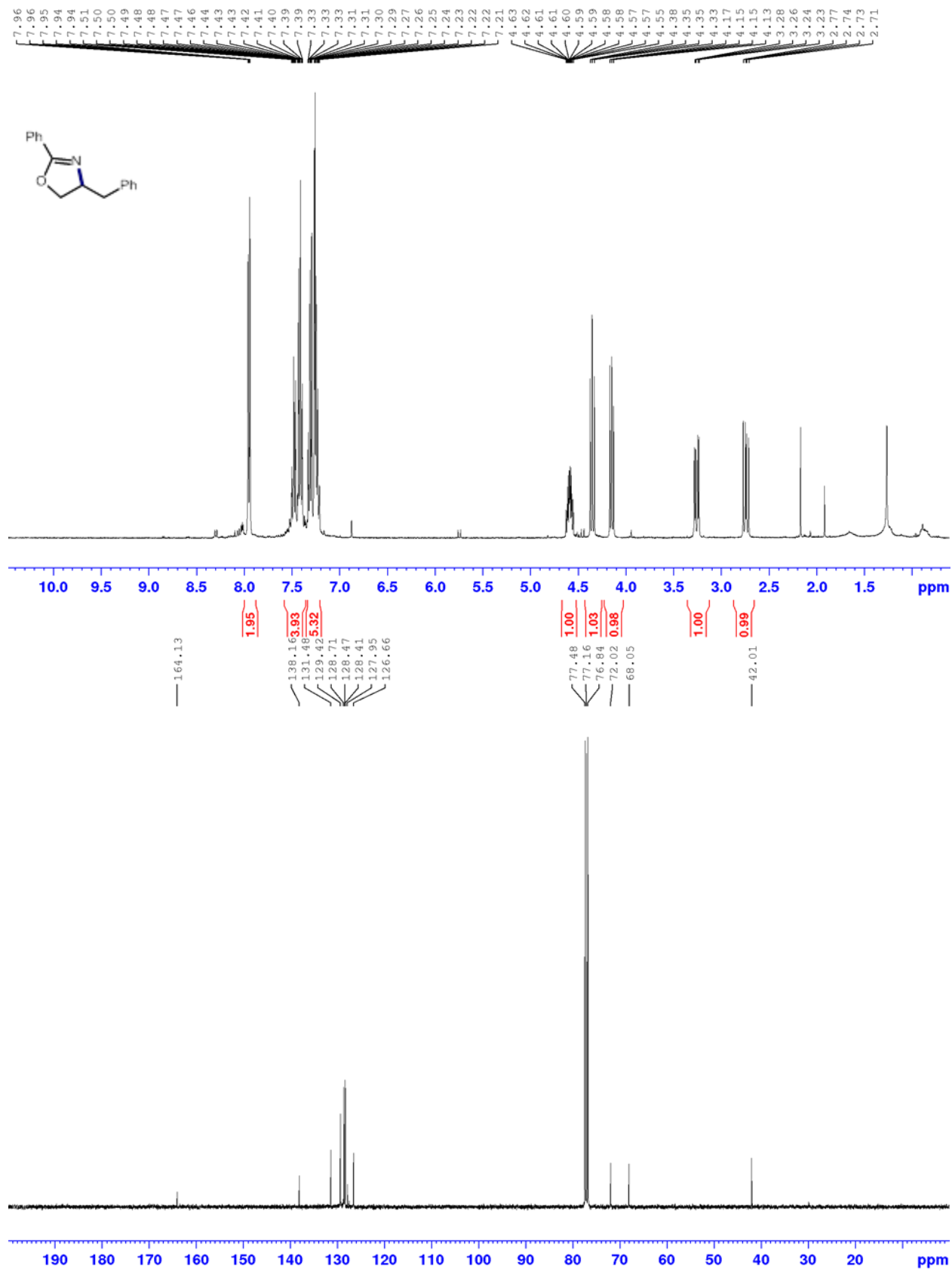

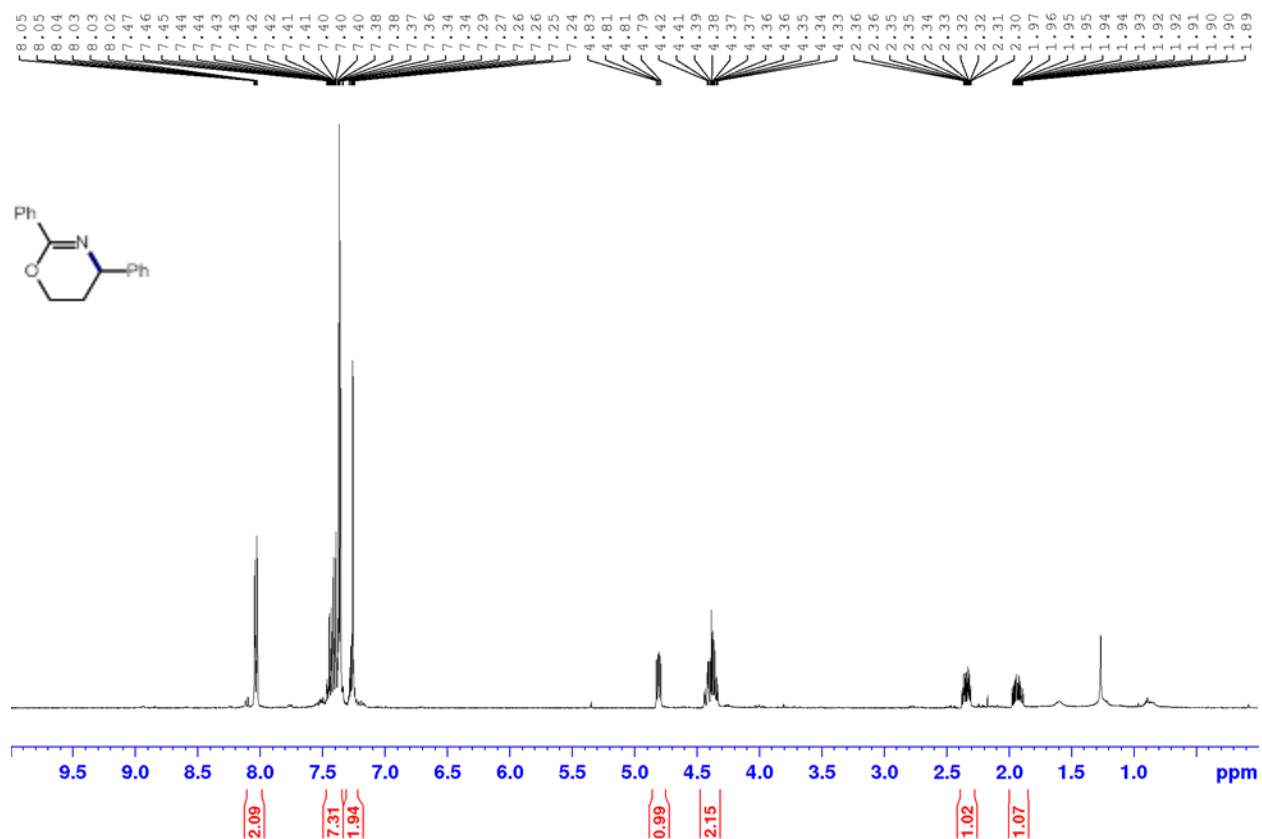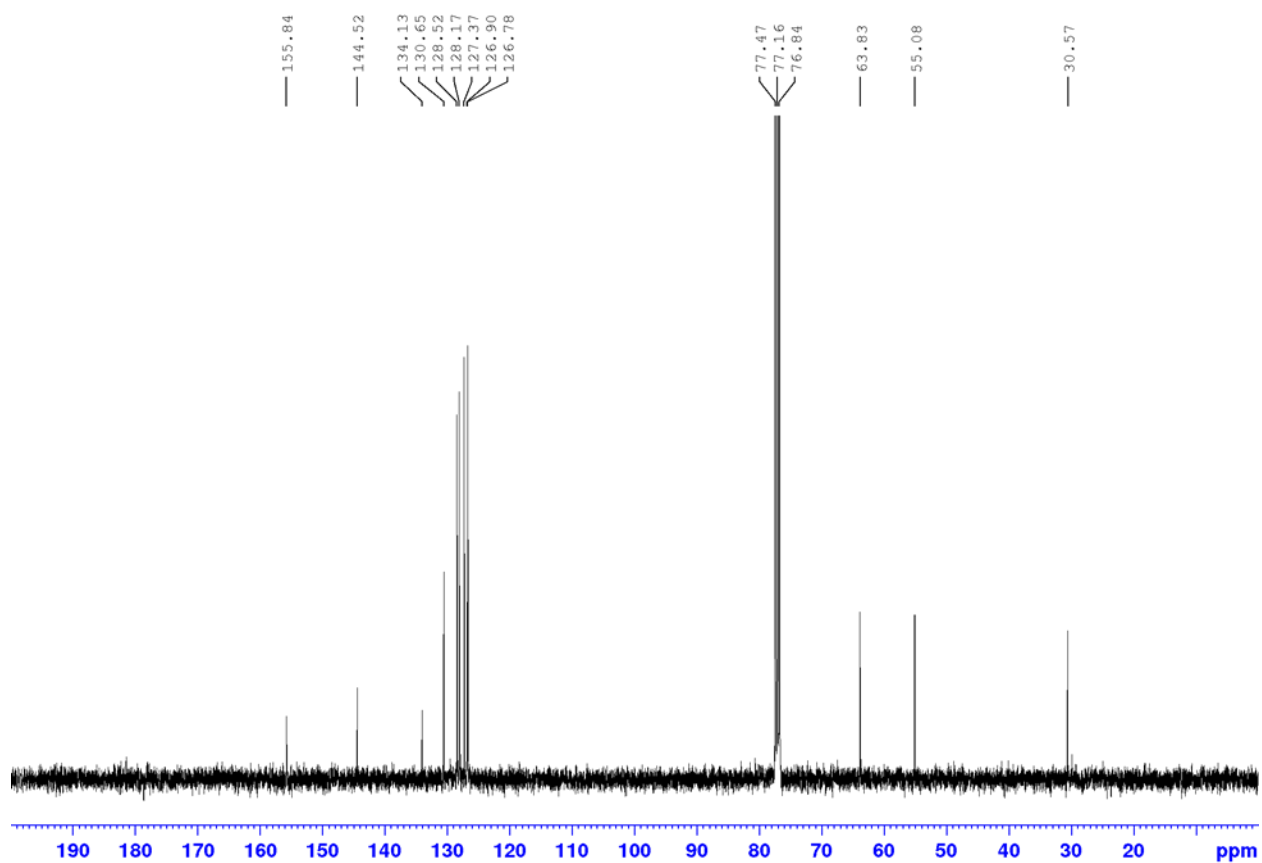

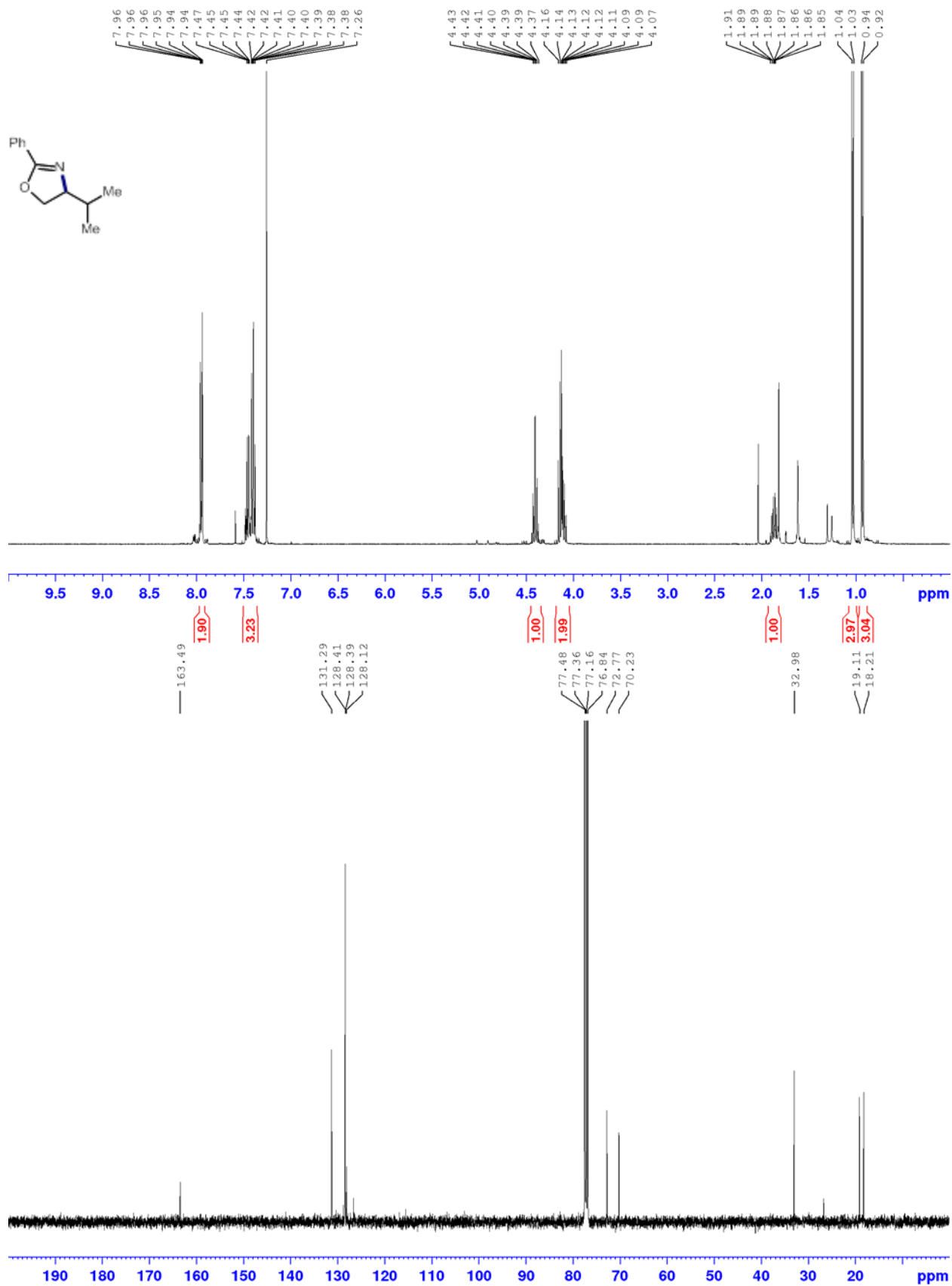

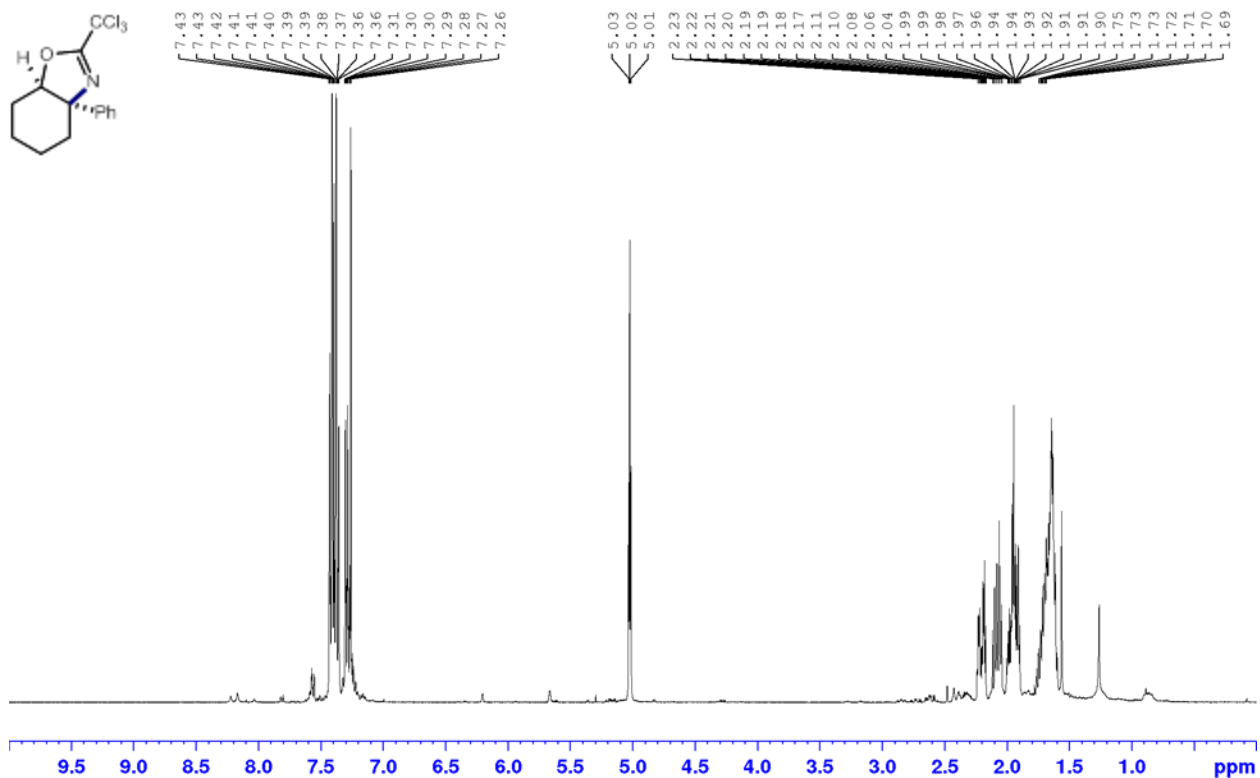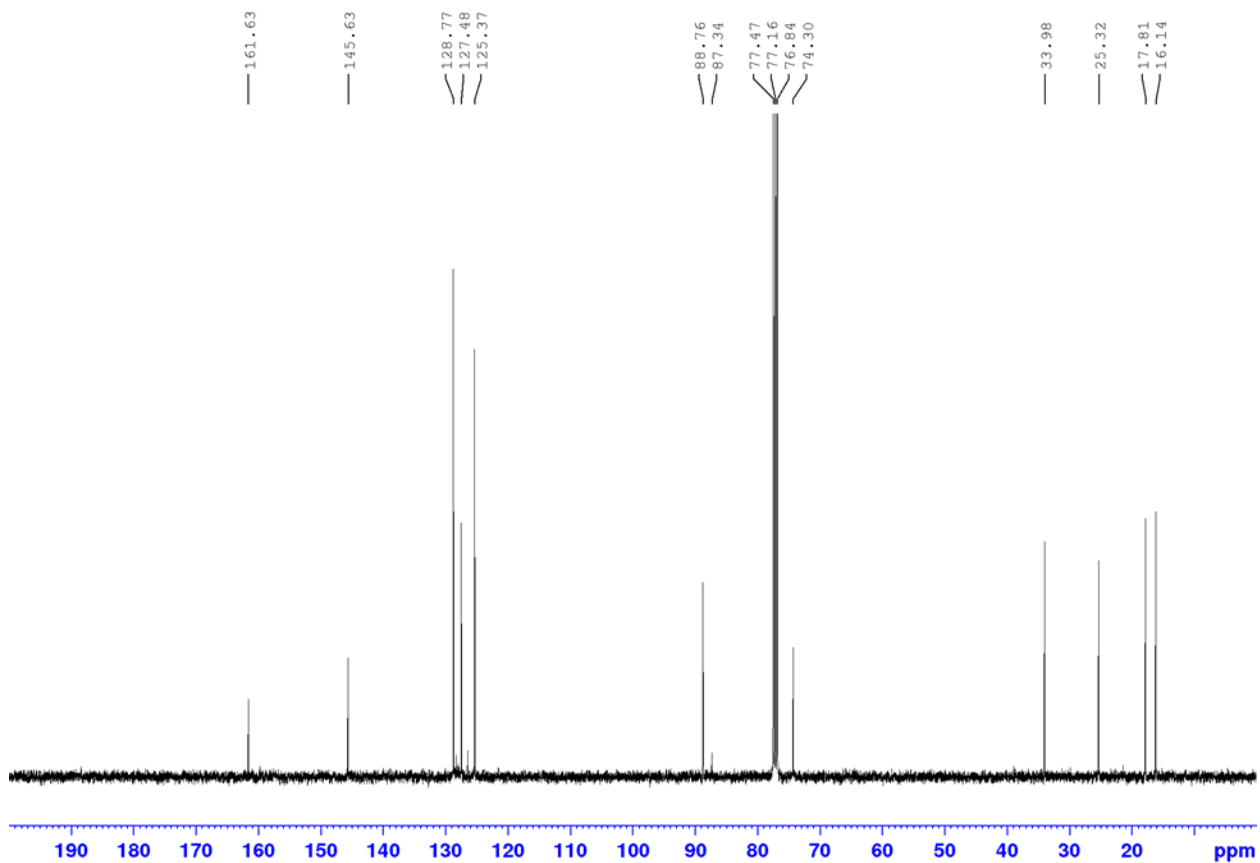

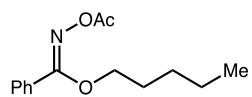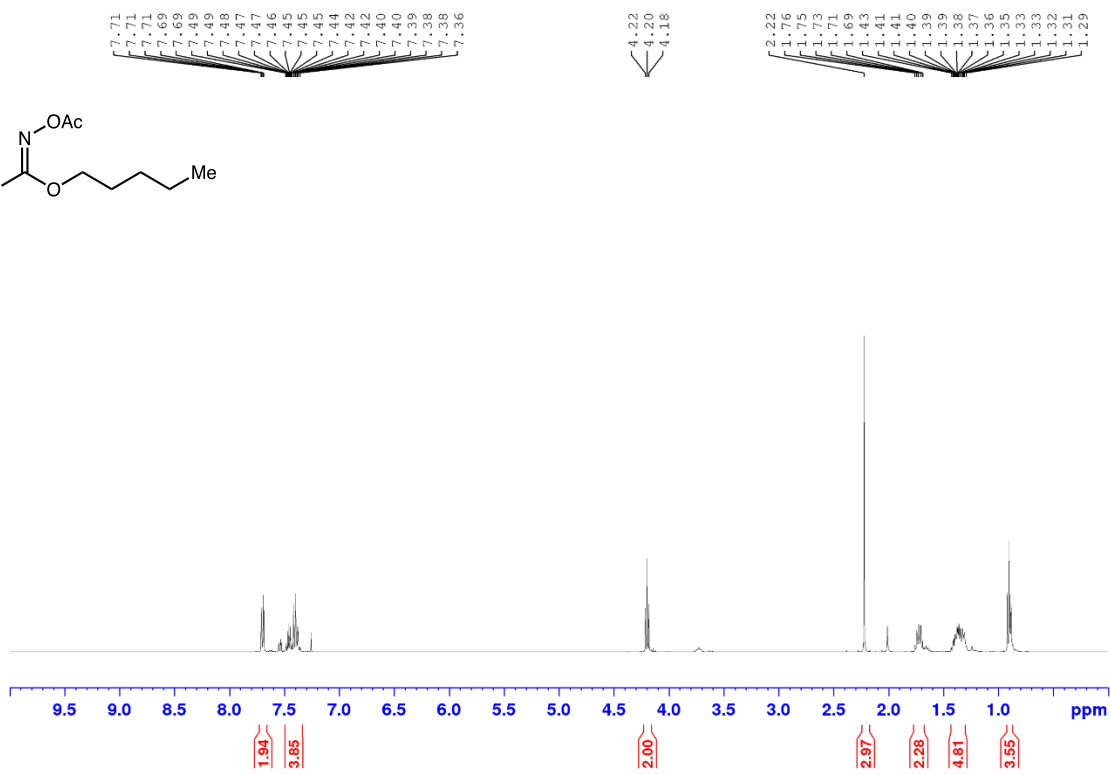

## Crude $^1\text{H}$ NMR: C-H Amination

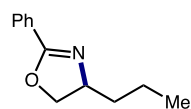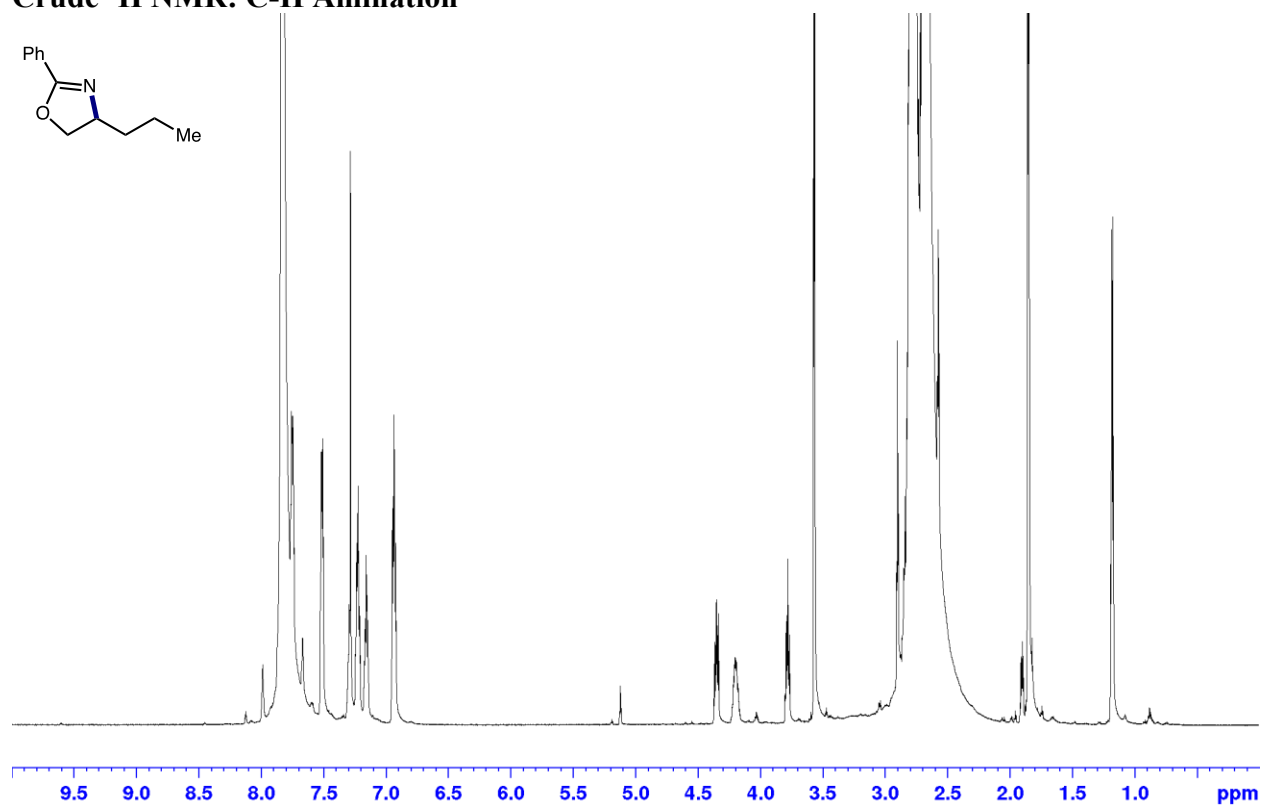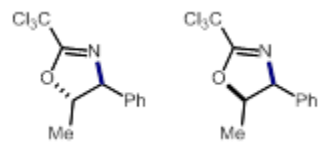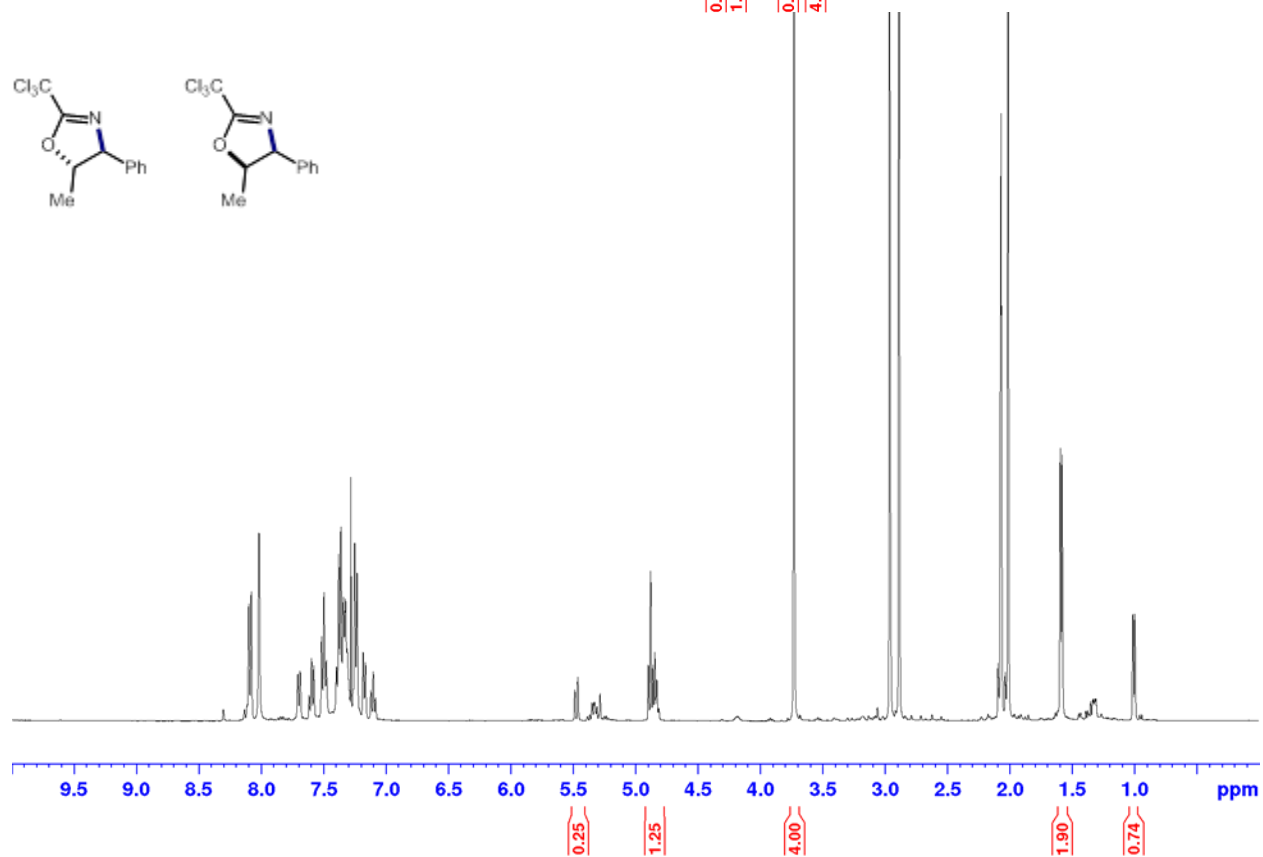

Supplement: Supplementary file 1 [file SC-010-C8SC05685D-s001.pdf]
