# Supplementary material for: Catalytic β C–H amination via an imidate radical relay
Source: Chem Sci. 2019 Jan 17;10(9):2693–9. doi: 10.1039/c8sc05685d (PMC6419930; doi:10.1039/c8sc05685d)
Supplement: Supplementary file 2 [file SC-010-C8SC05685D-s002.pdf]

Electronic Supplementary Material (ESI) for Chemical Science.
This journal is © The Royal Society of Chemistry 2019

Supporting Information for:

Catalytic β C-H Amination via an Imidate Radical Relay

Leah M. Stateman, Ethan A. Wappes,
Kohki M. Nakafuku, Kara M. Edwards, David A. Nagib

Department of Chemistry and Biochemistry, The Ohio State University,
151 W. Woodruff Ave., Columbus, OH, 43210, United States

Corresponding Author
*E-mail: nagib.1@osu.edu

Table of Contents

(with hyperlinks)

Section		Page
I.	General Information	S3
II.	General Procedure	S4
III.	Substrate Syntheses	S6
IV.	Optimization	S17
V.	Robustness Screen	S18
VI.	C-H Amination	S19
VII.	Mechanistic Studies	
	a. Time Studies	S28
	b. HAT Selectivity	S30
	c. Stereochemical Probe	S33
	d. Hammett Plot	S35
	e. Intermediate Probe	S37
VIII.	References	S38

General Information

All chemicals and reagents were purchased from Sigma-Aldrich, Alfa Aesar, Acros, TCI, or ChemImpex. $PhI(OAc)_2$ was ground and dried under high vacuum prior to use. Acetonitrile and triethylamine were distilled over calcium hydride before use. CH_2Cl_2, THF, and DMF were dried and degassed with nitrogen using an Innovative Technology solvent system. Silicycle F60 (230-400 mesh) silica gel was used or a CombiFlash® Automated Flash Chromatograph for flash column chromatography. Thin layer chromatography (TLC) analyses were performed using Merck silica gel 60 F254 plates and visualized under UV (254 nm), $KMnO_4$, or iodine stain. Melting points were determined using an Electrotherman IA9000. ^1H, ^{19}F, ^{13}C NMR spectra were recorded using a Bruker AVIII 400 or AVIII 600 MHz NMR spectrometer. ^1H NMR and ^{13}C NMR chemical shifts are reported in parts per million and referenced with respect to $CDCl_3$ (^1H: residual $CHCl_3$ at δ 7.26, ^{13}C: $CDCl_3$ triplet at δ 77.16). ^1H NMR data are reported as chemical shifts (δ ppm), multiplicity (s = singlet, bs = broad singlet, d = doublet, t = triplet, q = quartet, quint = quintet, m = multiplet, app t = apparent triplet, app q = apparent quartet, app qd = apparent quartet of doublets), coupling constant (Hz), relative integral. ^{19}F NMR data are reported as chemical shifts (δ ppm). High resolution mass spectra were obtained using Bruker MicrOTOF (ESI). IR spectra were recorded using a Thermo Fisher Nicolet iS10 FT-IR and are reported in terms of frequency of absorption (cm–1). Previously reported imidates and amino alcohols were characterized by ^1H and ^{13}C NMR and matched previous spectra.[1]

I. General Procedure

Trichloroacetimidate Formation – General Procedure (GP1)

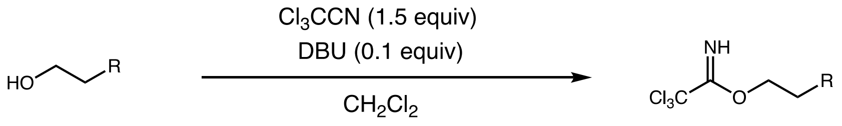

To a round-bottom flask containing a stir bar, alcohol (1 equiv.), and CH_2Cl_2 (0.1 M) was added trichloroacetonitrile (1.5 equiv.) and DBU (0.1 equiv.). The solution was stirred and monitored by TLC until consumption of alcohol. Upon completion, the solution was concentrated and directly loaded onto silica gel and purified.

Benzimidate Formation – General Procedure (GP2)

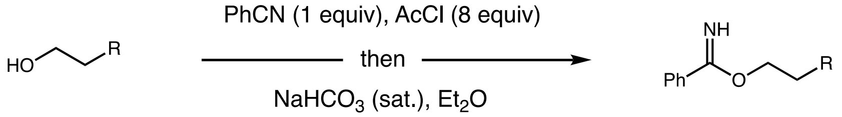

To a 50 mL round bottom flask was added benzonitrile (1 equiv.), alcohol (12 equiv.), and a stir bar. The solution was cooled to 0 °C, an addition funnel containing acetyl chloride (8 equiv.) was attached, and the apparatus was sealed. The acetyl chloride was added dropwise over a half an hour and then the solution was warmed to room temperature (*warning: HCl gas is evolved during this addition, poorly sealed glassware will allow gas to escape)*. The reaction was stirred at room temperature for 24 hours and then evaporated under reduced pressure to yield the imidate•HCl salt. Free base protocol. The salt was suspended in Et_2O and a saturated solution of $NaHCO_3$ was added dropwise until the salt dissolved completely. Upon dissolution, the solution was stirred for five minutes and then diluted with H_2O. The organic phase was separated and the aqueous phase was extracted with Et_2O (2 x 10 mL). The combined organic phase was dried over $MgSO_4$ and concentrated. In most cases, the imidate was analytically pure upon isolation. Alternatively, the crude material could be purified by column chromatography.

Benzimidate Formation – General Procedure (GP3)

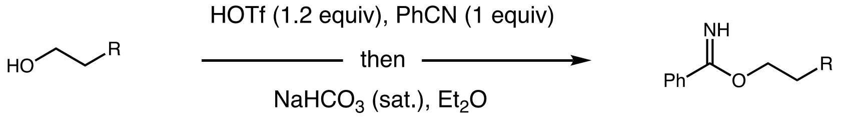

To a 2-dram vial equipped with a stir bar was added the desired alcohol (1 equiv.) and benzonitrile (1 equiv.) and dry solvent (toluene or dichloroethane, 0.5 M). Triflic acid (1.2 equiv.) was added to the solution, the vial was sealed, and heated to reflux for 24 hours. Upon completion, the vial was cooled to room temperature and evaporated to dryness. The resulting solid was suspended in Et_2O and subjected to the free base protocol from **GP2**. The product was purified by column chromatography.

Transimidation – General Procedure (GP4)

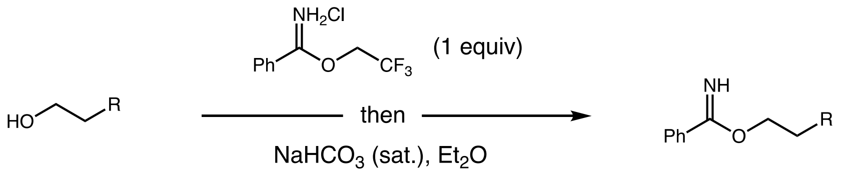

To a 2-dram vial equipped with a stir bar was added the trifluoroethyl benzimidate hydrochloride[1] (1 equiv.), the desired alcohol (1 equiv), and MeCN (0.2 M). The reaction was heated to 50 °C and stirred until consumption of starting imidate (monitored by ^{1}H NMR). Upon completion, the solution was concentrated and the resulting crude solid was suspended in Et_2O and subjected to the free base protocol from **GP2**. The crude reaction mixture was loaded onto silica gel and purified.

C-H Amination – General Procedure (GP5)

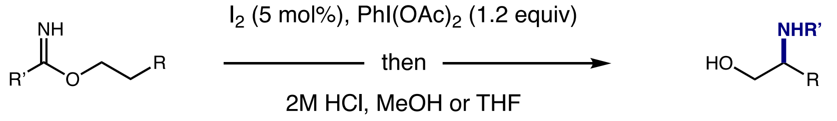

To a 2-dram vial equipped with PTFE septa cap and magnetic stir bar was added imidate (0.4 mmol, 1 equiv.) and $PhI(OAc)_2$ (154.6 mg, 0.48 mmol, 1.2 equiv.). This vial was evacuated and backfilled with N_2 (3x). A degassed stock solution of I_2 in dry DMF (2 mL, 0.01 M, 0.05 equiv.) was added to the vial under N_2. The reaction was heated to 50°C (by placing vial in an aluminum heating block) and stirred for 4 hours. Upon completion, the reaction was cooled, transferred to a round-bottom flask, and concentrated *in vacuo*. *Hydrolysis and acid/base extraction.* To the flask, was added methanol (4 mL) and 2M HCl (0.8 mL). After stirring for 2 hours, 25 mL of $CHCl_3$ and 10 mL of H_2O was added. The aqueous layer was washed with $CHCl_3$ (5 x 25 mL). The combined organic fractions were rewashed with H_2O (10 mL). The combined aqueous layer was poured into a round bottom, diluted with $CHCl_3$ (25 mL), and finally 6M NaOH (10 mL) was added and stirred for 30 minutes. The aqueous layer was washed with $CHCl_3$ (5 x 25 mL) and the combined organic solution was dried over $MgSO_4$ and concentrated to yield the pure amino alcohol.

I_2 Stock Solution (0.01 M in DMF)

To a flame-dried Schlenk flask equipped with a stir bar, was added I_2 (50.8 mg, 0.2 mmol) and dry DMF (20 mL). The resulting solution was degassed via a free-pump-thawed protocol three times. The solution was warmed to room temperature under N_2 and used directly in the amination protocol.

Notes

1. $PhI(OAc)_2$ was ground and dried under vacuum prior to use.
2. To avoid incident light, the stock solution flask was covered with aluminum foil.
3. Large quantities of residual DMF during hydrolysis lead to suppressed isolated yields.
4. Although the reaction is typically complete in one hour, to ensure completion for all substrates the standard reaction time was made four hours.

III. Substrate Synthesis

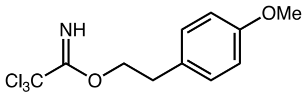

4-methoxyphenethyl 2,2,2-trichloroacetimidate (S3)

4-methoxyphenethyl alcohol (0.1 g, 0.7 mmol) was subjected to **GP1**. Following reaction completion, the solvent was removed *in vacuo* and the crude mixture was purified (silica gel, hexanes with 1% Et_3N) to yield imidate **S3** (0.18 g, 92%) as a colorless solid. R_f: 0.31 (10% Ethyl acetate/hexanes). ^{1}H NMR (400 MHz, $CDCl_3$) δ: 8.27 (bs, 1H), 7.23 – 7.17 (m, 2H), 6.88 – 6.82 (m, 2H), 4.45 (t, J = 7.0 Hz, 2H), 3.80 (s, 3H), 3.03 (t, J = 6.8 Hz, 2H). ^{13}C NMR (100 MHz, $CDCl_3$) δ: 163.0, 158.5, 130.2, 129.8, 114.0, 91.6, 70.3, 55.4, 34.0.

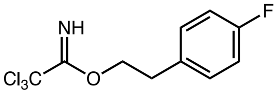

4-fluorophenethyl 2,2,2-trichloroacetimidate (S4)

4-fluorophenethyl alcohol (701 mg, 0.625 mL, 5 mmol) was subjected to **GP1**. Following reaction completion, the solvent was removed *in vacuo* and the crude mixture was purified (silica gel, hexanes with 1% Et_3N) to yield imidate **S4** (1.42 g, 99%) as a colorless oil. R_f: 0.38 (10% Ethyl acetate/hexanes). ^{1}H NMR (400 MHz, $CDCl_3$) δ: 8.29, (bs, 1H), 7.27 – 7.22 (m, 2H), 7.01 – 6.97 (m, 2H), 4.47 (t, J = 6.8 Hz, 2H), 3.06 (t, J = 6.8 Hz, 2H). ^{13}C NMR (100 MHz, $CDCl_3$) δ: 162.9, 161.9 (d, $^1J_{CF}$ = 244.3 Hz), 133.5 (d, $^4J_{CF}$ = 3.3 Hz), 130.7 (d, $^3J_{CF}$ = 7.8 Hz), 115.4 (d, $^2J_{CF}$ = 21.2 Hz), 91.5, 69.9, 34.1. ^{19}F NMR (100 MHz, $CDCl_3$): –116.5 – (–116.6) (m).

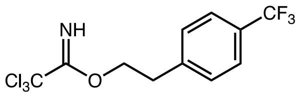

4-(trifluoromethyl)phenethyl 2,2,2-trichloroacetimidate (S5)

4-(trifluoromethyl)phenethyl alcohol (735 mg, 4 mmol) was subjected to **GP1**. Following reaction completion, the solvent was removed *in vacuo* and the crude mixture was purified (silica gel, hexanes with 1% Et_3N) to yield imidate **S5** (1.10 g, 83%) as a colorless solid. R_f: 0.25 (10% Ethyl acetate/hexanes). ^{1}H NMR (600 MHz, $CDCl_3$) δ: 8.32 (bs, 1H), 7.56 (d, J = 7.9 Hz, 2H), 7.40 (d, J = 7.9 Hz, 2H), 4.52 (t, J = 6.6 Hz, 2H), 3.15 (t, J = 7.9 Hz, 2H). ^{13}C NMR (150 MHz, $CDCl_3$) δ: 162.8, 142.0, 129.56, 129.5 (q, $^2J_{CF}$ = 31.0 Hz), 124.4 (q, $^3J_{CF}$ = 3.6 Hz), 124.4 (q, $^1J_{CF}$ = 271.8 Hz), 91.4, 69.3, 34.7.

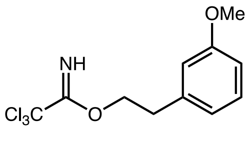

3-methoxyphenethyl 2,2,2-trichloroacetimidate (S6)

2-(3-methoxyphenyl)ethan-1-ol (0.2 g, 1.3 mmol) was subjected to **GP1**. Following reaction completion, the solvent was removed *in vacuo* and the crude mixture was purified (silica gel, hexanes with 1% Et_3N) to yield imidate **S6** (0.37 g, 95%) as a clear colorless oil. R_f: 0.36 (10% Ethyl acetate/hexanes). ^1H NMR (400 MHz, $CDCl_3$) δ: 8.29 (bs, 1H), 7.22 (t, J = 7.8 Hz, 1H), 6.88 – 6.84 (m, 2H), 6.88 – 6.84 (m, 1H), 4.50 (t, J = 6.9 Hz, 2H), 3.80 (s, 3H), 3.07 (t, J = 6.9 Hz, 2H). ^{13}C NMR (100 MHz, $CDCl_3$) δ: 162.9, 159.8, 139.4, 129.6, 121.5, 114.9, 112.3, 91.6, 69.9, 55.3, 34.9.

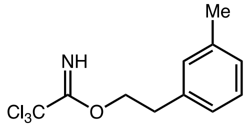

3-methylphenethyl 2,2,2-trichloroacetimidate (S7)

2-(3-methylphenyl)ethanol (613 mg, 4.5 mmol) was subjected to **GP1**. Following reaction completion, the solvent was removed *in vacuo* and the crude mixture was purified (silica gel, hexanes with 1% Et_3N) to yield imidate **S7** (1.2 g, 95%) as a clear colorless oil. R_f: 0.61 (20% Ethyl acetate/hexanes). ^1H NMR (400 MHz, $CDCl_3$) δ: 8.28 (bs, 1H), 7.20 (t, J = 7.5 Hz, 1H), 7.11 – 7.04 (m, 3H), 4.48 (t, J = 6.9 Hz, 2H), 3.06 (t, J = 7.0 Hz, 2H), 2.33 (s, 3H). ^{13}C NMR (100 MHz, $CDCl_3$) δ: 163.0, 138.2, 137.6, 130.1, 128.5, 127.5, 126.2, 91.6, 70.1, 34.7, 21.5.

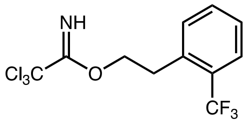

2-(trifluoromethyl)phenethyl 2,2,2-trichloroacetimidate (S8)

2-(2-(trifluoromethyl)phenyl)ethan-1-ol was subjected to **GP1**. After concentration, the crude mixture was purified (silica gel, hexanes with 1% Et_3N) to yield trichloroacetimidate **S8** (1.56 g, 48%) as a clear oil. R_f: 0.41 (10% Ethyl acetate/hexanes). ^1H NMR (600 MHz, $CDCl_3$) δ: 8.31 (bs, 1H), 7.65 (d, J = 7.9 Hz, 2H), 7.48 (d, J = 4.7 Hz, 2H), 7.36 – 7.33 (m, 1H), 4.51 (t, J = 6.7 Hz, 2H), 3.29 (t, J = 6.7 Hz, 2H). ^{13}C NMR (150 MHz, $CDCl_3$) δ: 162.8, 136.3, 132.3, 131.8, 129.1 (q, $^2J_{CF}$ = 29.5 Hz), 127.0, 126.2 (q, $^3J_{CF}$ = 6.6 Hz), 124.7 (q, $^1J_{CF}$ = 274.2 Hz), 91.5, 69.3, 31.6. ^{19}F (376 MHz, $CDCl_3$) δ: – 59.5.

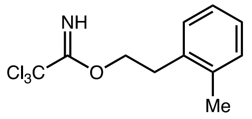

2-methylphenethyl 2,2,2-trichloroacetimidate (S9)

2-(*o*-tolyl)ethanol was subjected to **GP1**. After concentration, the crude mixture was purified (silica gel, hexanes with 1% Et_3N) to yield trichloroacetimidate **S9** (0.76 g, 84%) as a clear oil. R_f: 0.50 (10% Ethyl acetate/hexanes). ^1H NMR (400 MHz, $CDCl_3$) δ: 8.29 (bs, 1H), 7.26 – 7.14 (m, 4H), 4.47 (t, *J* = 7.2 Hz, 2H), 3.10 (t, *J* = 7.2 Hz, 2H). ^{13}C NMR (150 MHz, $CDCl_3$) δ: 163.0, 136.6, 135.8, 130.4, 129.8, 126.9, 126.2, 91.6, 69.2, 32.0, 19.6.

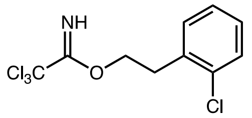

2-chlorophenethyl 2,2,2-trichloroacetimidate (S10)

2-(2-chlorophenyl)ethan-1-ol was subjected to **GP1**. After concentration, the crude mixture was purified (silica gel, hexanes with 1% Et_3N) to yield trichloroacetimidate **S10** (0.8 g, 87%) as a clear oil. R_f: 0.50 (10% Ethyl acetate/hexanes). ^1H NMR (400 MHz, $CDCl_3$) δ: 8.29 (bs, 1H), 7.38 – 7.31 (m, 2H), 7.22 – 7.18 (m, 2H), 4.53 (t, *J* = 6.7 Hz, 2H), 3.24 (t, *J* = 6.7 Hz, 2H). ^{13}C NMR (100 MHz, $CDCl_3$) δ: 162.9, 135.4, 134.3, 131.6, 129.6, 128.3, 126.9, 91.5, 68.3, 32.5.

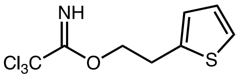

2-(thiophen-2-yl)ethyl 2,2,2-trichloroacetimidate (S11)

2-(thiophen-2-yl)ethan-1-ol (577 mg, 1.6 mmol) was subjected to **GP1**. Following reaction completion, the solvent was removed *in vacuo* and the crude mixture was purified (silica gel, hexanes with 1% Et_3N) to yield imidate **S11** (366 mg, 86%) as a clear colorless oil. R_f: 0.63 (20% Ethyl acetate/hexanes). ^1H NMR (400 MHz, $CDCl_3$) δ: 8.32 (bs, 1H), 7.17 (dd, *J* = 5.0, 1.4 Hz, 1H), 6.96 – 6.92 (m, 2H), 4.51 (t, *J* = 6.7 Hz, 2H), 3.31 (t, *J* = 6.7 Hz, 2H). ^{13}C NMR (100 MHz, $CDCl_3$) δ: 162.9, 139.7, 127.0, 125.9, 124.2, 91.5, 69.6, 29.0.

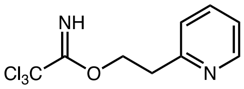

2-(pyridin-2-yl)ethyl 2,2,2-trichloroacetimidate (S12)

2-(2-pyridyl)ethanol (0.55 g, 0.51 mL, 4.5 mmol) was subjected to **GP1**. Following reaction completion, the solvent was removed *in vacuo* and the crude mixture was purified (silica gel, hexanes → 40% ethyl acetate/hexanes) to yield imidate **S12** (1.16 g, 96%) as a clear colorless oil. Rf: 0.59 (10% Isopropanol/CH_2Cl_2). ^1H NMR (400 MHz, $CDCl_3$) δ: 8.54 – 8.52 (m, 1H), 8.28 (bs, 1H), 7.58 (td, *J* = 7.7, 1.8 Hz, 1H), 7.24 – 7.22 (m, 1H), 7.14 – 7.10 (m, 1H), 4.66 (t, *J* = 6.6 Hz,

2H), 3.24 (t, J = 6.7 Hz, 2H). ^{13}C NMR (100 MHz, $CDCl_3$) δ: 162.8, 157.9, 149.6, 136.4, 123.8, 121.7, 91.5, 68.6, 37.1.

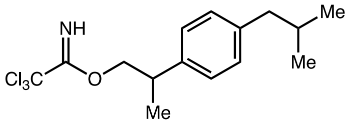

2-(4-isobutylphenyl)propyl 2,2,2-trichloroacetimidate (S13)

2-(4-isobutylphenyl)propan-1-ol was subjected to **GP1**. After concentration, the crude mixture was purified (silica gel, hexanes with 1% Et_3N) to yield trichloroacetimidate **S13** (1.35 g, 83%) as a clear oil. R_f: 0.42 (10% Ethyl acetate/hexanes). ^{1}H NMR (400 MHz, $CDCl_3$) δ: 8.23 (bs, 1H), 7.19 – 7.17 (m, 2H), 7.09 – 7.07 (m, 2H), 4.39 (dd, J = 9.8, 6.1 Hz, 1H), 4.27 (dd, J = 10.2, 7.8 Hz, 1H), 3.30 – 3.21 (m, 1H), 2.44 (d, J = 7.1 Hz, 2H), 1.89 – 1.79 (m, 1H), 1.39 (d, J = 7.0 Hz, 3H), 0.89 (d, J = 6.4 Hz, 6H). ^{13}C NMR (100 MHz, $CDCl_3$) δ: 163.1, 140.2, 140.1, 139.3, 127.3, 91.7, 74.6, 45.2, 38.5, 30.4, 22.5, 18.0.

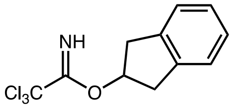

2,3-dihydro-1H-inden-2-yl 2,2,2-trichloroacetimidate (S14)

2,3-dihydro-1H-inden-2-ol was subjected to **GP1**. After concentration, the crude mixture was purified (silica gel, hexanes with 1% Et_3N) to yield trichloroacetimidate **S14** (1.5 g, 72%) as a low melting solid. R_f: 0.35 (10% Ethyl acetate/hexanes). ^{1}H NMR (400 MHz, $CDCl_3$) δ: 8.34 (bs, 1H), 7.26 – 7.23 (m, 2H), 7.21 – 7.18 (m, 2H), 5.71 – 5.66 (m, 1H), 3.43 (dd, J = 17.1, 6.7 Hz, 2H), 3.18 (dd, J = 17.1, 3.4 Hz, 2H). ^{13}C NMR (150 MHz, $CDCl_3$) δ: 162.7, 140.4, 126.9, 124.8, 91.8, 80.1, 39.3.

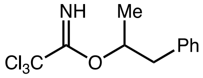

1-phenylpropan-2-yl 2,2,2-trichloroacetimidate (S15)

1-phenylpropan-2-ol was subjected to GP1. After concentration, the crude mixture was purified (silica gel, hexanes with 1% Et_3N) to yield trichloroacetimidate **S15** (1.77 g, 86%) as a clear oil. R_f: 0.42 (10% Ethyl acetate/hexanes) ^{1}H NMR (400 MHz, $CDCl_3$) δ: 8.24 (bs, 1H), 7.31 – 7.20 (m, 5H), 5.29 – 5.21 (m, 1H), 3.10 (dd, J = 13.8, 6.7 Hz, 1H), 2.89 (dd, J = 13.8, 6.2 Hz, 1H), 1.35 (d, J = 5.9 Hz, 3H). ^{13}C NMR (100 MHz, $CDCl_3$) δ: 162.2, 137.6, 129.8, 128.4, 126.6, 92.0, 41.9, 18.6. Spectral data matches those previously reported.[2]

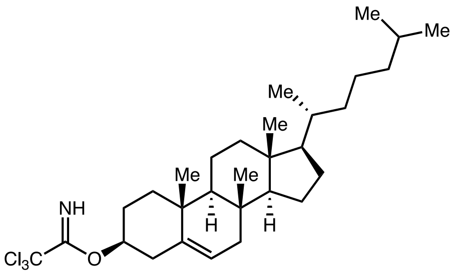

3-trichloroacetimidatyl cholesterol (S16)

Cholesterol (193 mg, 0.5 mmol) was subjected to **GP1**. Following reaction completion, the solvent was removed *in vacuo* and the crude mixture was purified (silica gel, hexanes with 1% Et_3N) to yield imidate **S16** (220 mg, 83%) as a white solid. Rf: 0.72 (20% Ethyl acetate/hexanes). ^{1}H NMR (400 MHz, $CDCl_3$) δ: 8.23 (bs, 1H), 5.42 – 5.41 (m, 1H), 4.81 – 4.72 (m, 1H), 2.55 – 2.40 (m, 2H), 2.08 – 0.97 (m, 31H), 0.92 (d, *J* = 6.6 Hz, 3H), 0.88 (d, *J* = 1.8 Hz, 3H), 0.86 (d, *J* = 1.7 Hz, 3H), 0.69 (s, 3H). ^{13}C NMR (100 MHz, $CDCl_3$) δ: 162.2, 139.6, 123.1, 92.1, 79.1, 56.9, 56.3, 50.2, 42.5, 39.9, 39.7, 37.5, 37.1, 36.9, 36.3, 35.9, 32.1, 32.0, 28.4, 28.2, 27.2, 24.4, 24.0, 23.0, 22.7, 21.2, 19.5, 18.9, 12.0.

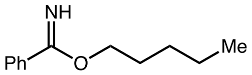

Pentyl benzimidate (1)

1-pentanol was subjected to **GP2**. Upon reaction completion, the mixture was cooled to room temperature and the solvent was removed *in vacuo*. The crude mixture was subjected to the free-base protocol, followed by purification (silica gel, hexanes with 1% EtOAc and 1% Et_3N) to yield imidate **1** (2.46 g, 80%) as a colorless oil. R_f: 0.31 (20% Ethyl acetate/hexanes). ^{1}H NMR (400 MHz, $CDCl_3$) δ: 7.72 (bs, 3H), 7.44 – 7.37 (m, 3H), 4.25 (bs, 2H), 1.82 – 1.77 (m, 2H), 1.47 – 1.35 (m, 4H), 0.94 – 0.91 (t, *J* = 7.3 Hz, 3H). ^{13}C NMR (100 MHz, $CDCl_3$) δ: 168.0, 133.0, 130.8, 128.4, 126.7, 66.3, 28.4, 28.4, 22.5, 14.0.

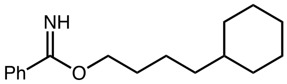

4-cyclohexylbutyl benzimidate (S18)

4-cyclohexylbutan-1-ol (569 mg, 0.607 mL, 4 mmol) was subjected to **GP3**. Upon reaction completion, the mixture was cooled to room temperature and the solvent was removed *in vacuo*. The crude mixture was subjected to the free-base protocol, followed by purification on silica gel (5% Ethyl acetate/hexanes with 1% Et_3N) to yield imidate **S18** (772 mg, 78%) as a colorless oil. R_f: 0.2 (20% Ethyl acetate/hexanes). ^{1}H NMR (400 MHz, $CDCl_3$) δ: 7.74 (bs, 3H), 7.49 – 7.38 (m, 3H), 4.25 (bs, 2H), 1.87 – 1.61 (m, 8H), 1.39 – 1.09 (m, 7H), 0.99 – 0.84 (m, 2H). ^{13}C NMR (100 MHz, $CDCl_3$) δ: 168.3, 133.2, 130.9, 128.6, 126.8, 66.7, 37.6, 34.0, 33.5 (2C), 26.8, 26.5, 26.3.

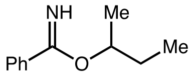

***Sec*-butyl benzimidate (S19)**

Butan-2-ol (8.60 g, 10.6 mL, 116 mmol) was subjected to **GP2**. Upon reaction completion, the mixture was cooled to room temperature and the solvent was removed *in vacuo*. The crude mixture was subjected to the free-base protocol, followed by purification on silica gel (5% Ethyl acetate/hexanes with 1% Et_3N to yield imidate **S19** (300 mg, 22%) as a colorless oil. R_f: 0.31 (20% Ethyl acetate/hexanes). ^1H NMR (600 MHz, $CDCl_3$) δ: 7.73 (bs, 3H), 7.45 – 7.38 (m, 3H), 5.08 (bs, 1H), 1.82 – 1.75 (m, 1H), 1.35 (d, J = 6.1 Hz, 3H), 1.00 (t, J = 7.5 Hz, 3H). ^{13}C NMR (150 MHz, $CDCl_3$) δ: 167.6, 133.7, 130.7, 128.5, 126.7, 73.0, 29.0, 19.2, 9.9.

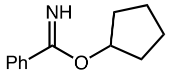

Cyclopentyl benzimidate (S20)

Cyclopentanol (86 mg, 0.090 mL, 1 mmol) was subjected to **GP4**. Upon reaction completion, the mixture was cooled to room temperature and the solvent was removed *in vacuo*. The crude mixture was subjected to the free-base protocol, followed by purification on silica gel (5% Ethyl acetate/hexanes with 1% Et_3N) to yield imidate **S20** (89 mg, 47%) as a colorless oil. R_f: 0.25 (20% Ethyl acetate/hexanes). ^1H NMR (600 MHz, $CDCl_3$) δ: 7.76 – 7.70 (m, 3H), 7.47 – 7.36 (m, 3H), 5.33 (bs, 1H), 2.04 – 1.75 (m, 6H), 1.71 – 1.59 (m, 2H). ^{13}C NMR (150 MHz, $CDCl_3$) δ: 167.4, 133.6, 130.8, 128.4, 126.8, 78.0, 32.8, 24.1.

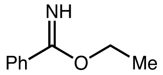

Ethyl benzimidate (S21)

Ethanol (5.36 g, 6.79 mL, 116 mmol) was subjected to **GP2**. After 24 hours, the mixture was concentrated yielding a white solid. This hydrochloride salt (0.5 g) was subjected to basic workup to yield imidate **S21** (0.33 g, 87%) as a colorless oil. R_f: 0.25 (20% Ethyl acetate/hexanes). ^1H NMR (400 MHz, $CDCl_3$) δ: 7.74 (bs, 3H), 7.48 – 7.39 (m, 3H), 4.34 (bs, 2H), 1.43 (t, J = 7.0 Hz, 3H). ^{13}C NMR (100 MHz, $CDCl_3$) δ: 167.7, 132.7, 130.5, 128.1, 126.4, 61.6, 14.0.

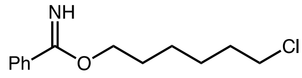

6-chlorohexylbenzimidate (S22)

6-chlorohexan-1-ol (546 mg, 0.534 mL, 4 mmol) was subjected to **GP3**. Upon reaction completion, the mixture was cooled to room temperature and the solvent was removed *in vacuo*. The crude mixture was subjected to the free-base protocol, followed by purification on silica gel (5% Ethyl acetate/hexanes with 1% Et_3N to yield imidate **S22** (852 mg, 89%) as a colorless oil. R_f: 0.30 (20% Ethyl acetate/hexanes). ^1H NMR (600 MHz, $CDCl_3$) δ: 7.73 (bs, 3H), 7.47 – 7.40 (m, 3H), 4.27 (bs, 2H), 3.54 (t, *J* = 6.7 Hz, 2H), 1.85 – 1.79 (m, 4H), 1.53 – 1.51 (m, 4H). ^{13}C NMR (150 MHz, $CDCl_3$) δ: 168.2, 133.0, 130.9, 128.6, 126.8, 66.0, 45.1, 32.6, 28.7, 26.8, 25.7; HRMS (ESI-TOF) *m/z*: calc'd for $C_{13}H_{19}ClNO$ $[M+H]^+$ 240.1155, found 240.1153. IR (film) cm–1: 3325, 3076, 2934, 1630, 1331, 1081.

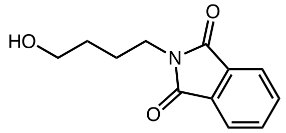

2-(4-hydroxybutyl)isoindoline-1,3-dione (S23A)

A mixture of 4-amino-1-butanol (445 mg, 5.0 mmol) and phthalic anhydride (740 mg, 5.0 mmol) in toluene (20 mL) was heated to reflux for 3 h. After cooling and removal of solvent, the crude product was purified (50% Ethyl acetate/hexanes) to yield alcohol **S23A** as a white crystalline solid (1.1 g, 99% yield). R_f: 0.25 (50% Ethyl acetate/hexanes). ^1H NMR (400 MHz, CD_3CN) δ: 7.87 – 7.81 (m, 2H), 7.74 – 7.68 (m, 2H), 3.74 (t, *J* = 7.1 Hz, 2H), 3.70 (t, *J* = 6.4 Hz, 2H), 1.83 – 1.74 (m, 2H), 1.67 – 1.57 (m, 2H). ^{13}C NMR (100 MHz, CD_3CN) δ: 168.6, 134.1, 132.3, 123.4, 62.5, 37.8, 29.9, 25.3

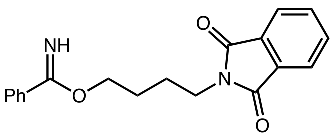

4-(1,3-dioxoisoindolin-2-yl)butyl benzimidate (S23)

2-4-hydroxybutyl)isoindoline-1,3-dione **S23A** (575 mg, 2.4 mmol) and benzimidate salt (575 mg, 2.4 mmol) were subjected to **GP4**. Upon reaction completion, the mixture was cooled to room temperature and the solvent was removed *in vacuo*. The crude mixture was subjected to the free-base protocol, followed by purification on silica gel (hexanes to 30% ethyl acetate in hexanes) to yield imidate **S23** (352 mg, 91%) as a colorless solid. R_f: 0.31 (hexanes to 30% Ethyl acetate/hexanes). ^1H NMR (400 MHz, $CDCl_3$) δ: 7.84 (dd, *J* = 5.4, 3.0 Hz, 2H), 7.77 (bs, 1H), 7.70 (dd, *J* = 5.4, 3.0 Hz, 4H), 7.45 – 7.43 (m, 1H), 7.40 – 7.38 (m, 2H), 4.31 (bs, 2H), 3.78 (t, *J* = 6.8 Hz, 2H), 1.91 – 1.85 (m, 4H). ^{13}C NMR (100 MHz, $CDCl_3$) δ: 168.5, 168.3, 134.0, 132.9, 132.3, 131.0, 128.6, 126.8, 123.3, 65.6, 37.9, 26.3, 25.7.

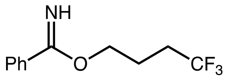

4,4,4-trifluorobutyl benzimidate (S24)

4,4,4-trifluorobutan-1-ol (512 mg, 0.423 mL, 4 mmol) was subjected to **GP3**. Upon reaction completion, the mixture was cooled to room temperature and the solvent was removed *in vacuo*. The crude mixture was subjected to the free-base protocol, followed by purification on silica gel (5% Ethyl acetate/hexanes with 1% Et_3N) to yield imidate **S24** (485 mg, 53%) as a colorless oil. R_f: 0.13 (20% Ethyl acetate/hexanes). ^{1}H NMR (400 MHz, $CDCl_3$) δ: 7.87 (bs, 1H), 7.70 (bs, 2H), 7.53 – 7.41 (m, 3H), 4.36 (bs, 2H), 2.37 – 2.25 (m, 2H), 2.12 – 2.05 (m, 2H). ^{13}C NMR (100 MHz, $CDCl_3$) δ: 168.1, 132.5, 131.2, 128.7, 126.7, 125.9 (q, $^1J_{CF}$ = 276.0 Hz), 64.5, 31.1 (q, $^2J_{CF}$ = 29.2 Hz), 21.8 (d, $^3J_{CF}$ = 2.9 Hz). ^{19}F (376 MHz, $CDCl_3$) δ: – 66.4 (t, J = 10.8 Hz). HRMS (ESI-TOF) *m/z*: calc'd for $C_{11}H_{13}F_3NO$ [M+H]$^+$ 232.0949, found 232.0962. IR (film) cm^{-1}: 3313, 3071, 2950, 1631, 1328, 1250.

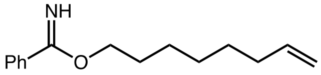

oct-7-en-1-yl benzimidate (S25)

oct-7-en-1-ol (128 mg, 0.152 mL, 1 mmol) was subjected to **GP4**. Upon reaction completion, the mixture was cooled to room temperature and the solvent was removed *in vacuo*. The crude mixture was subjected to the free-base protocol, followed by purification on silica gel (5% Ethyl acetate/hexanes with 1% Et_3N) to yield imidate **S25** (185 mg, 80%) as a colorless oil. R_f: 0.28 (20% Ethyl acetate/hexanes). ^{1}H NMR (400 MHz, $CDCl_3$) δ: 7.79 – 7.73 (m, 3H), 7.68 – 7.38 (m, 3H), 5.88 – 5.75 (m, 1H), 5.04 – 4.91 (m, 2H), 4.27 – 4.24 (t, J = 7.0 Hz, 2H), 2.10 – 2.02 (m, 2H), 1.86 – 1.77 (m, 2H), 1.54 – 1.35 (m, 6H). ^{13}C NMR (100 MHz, $CDCl_3$) δ: 163.5, 138.9, 131.3, 128.41, 128.35, 128.1, 114.6, 72.7, 66.9, 36.0, 33.8, 29.0, 25.5. HRMS (ESI-TOF) *m/z*: calc'd for $C_{15}H_{22}NO$ [M+H]$^+$ 232.1701, found 232.1701. IR (film) cm^{-1}: 3296, 3063, 2966, 2902, 1634, 1336, 1255.

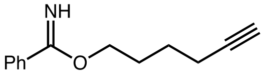

hex-5-yn-1-yl benzimidate (S26)

hex-5-yn-1-ol (98 mg, 0.110 mL, 1 mmol) was subjected to **GP4**. Upon reaction completion, the mixture was cooled to room temperature and the solvent was removed *in vacuo*. The crude mixture was subjected to the free-base protocol, followed by purification on silica gel (5% Ethyl acetate/hexanes with 1% Et_3N) to yield imidate **S26** (219 mg, 100%) as a colorless solid. R_f: 0.13 (20% Ethyl acetate/hexanes). ^{1}H NMR (400 MHz, $CDCl_3$) δ: 7.80 – 7.66 (m, 3H), 7.49 – 7.39 (m, 3H), 4.30 (t, J = 6.1 Hz, 2H), 2.29 (td, J = 10.7, 2.8 Hz, 2H), 1.98 – 1.90 (m, 3H), 1.79 – 1.70 (m,

2H). ^{13}C NMR (100 MHz, $CDCl_3$) δ: 164.0, 131.5, 128.44, 128.41, 127.9, 83.8, 72.5, 68.9, 65.9, 34.9, 29.8, 15.6. HRMS (ESI-TOF) *m/z*: calc'd for $C_{13}H_{16}NO$ $[M+H]^+$ 202.1232, found 232.1223. IR (film) cm^{-1}: 3293, 3258, 3072, 2941, 1628, 1333, 1297.

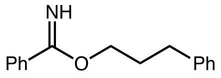

3-phenylpropyl benzimidate (S27)

3-phenyl-propanol was subjected to **GP3**. After filtration, a crude salt was isolated. 500 mg of the triflate salt was free-based to yield imidate **S27** (150 mg, 49%) as a clear oil. R_f: 0.16 (20% Ethyl acetate/hexanes). ^{1}H NMR (600 MHz, $CDCl_3$) δ: 7.75 (bs, 3H), 7.49 – 7.46 (m, 1H), 7.44 – 7.42 (m, 2H), 7.32 – 7.29 (m, 2H), 7.25 – 7.24 (m, 2H), 7.22 – 7.20 (m, 1H), 4.31 (bs, 2H), 2.84 (t, *J* = 7.7 Hz, 2H), 2.18 – 2.14 (m, 2H). ^{13}C NMR (150 MHz, $CDCl_3$) δ: 168.3, 141.7, 133.0, 131.0, 128.59, 128.56, 126.8 (2C), 126.1, 65.4, 32.6, 30.5. HRMS (ESI-TOF) m/z: calc'd for $C_{16}H_{18}NO$ $[M+H]^+$ 240.1388, found 240.1390. IR (film) cm^{-1}: 3337, 3062, 3026, 2949, 2887, 2859, 1633, 1602, 1578.

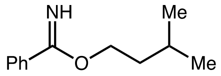

isopentyl benzimidate (S28)

3-methylbutanol (1.71 g, 2.11 mL, 19.4 mmol) and benzonitrile (1 g, 1 mL, 9.7 mmol) were dissolved in 2.4 mL of HCl in dioxanes (4M, 9.6 mmol). The reaction was stirred for 2 days and then concentrated. The resultant salt was suspended in Et_2O and free-based according to **GP2** to yield imidate **S28** (0.31 g, 17%) as a clear oil. R_f: 0.34 (20% Ethyl acetate/hexanes). ^{1}H NMR (400 MHz, $CDCl_3$) δ: 7.73 (bs, 3H), 7.48 – 7.39 (m, 3H), 4.32 (bs, 2H), 1.90 – 1.80 (m, 1H), 1.71 (app q, *J* = 6.8 Hz, 2H), 0.99 (d, *J* = 6.7 Hz, 6H). ^{13}C NMR (150 MHz, $CDCl_3$) δ: 168.4, 133.1, 130.9, 128.5, 126.8, 64.8, 37.6, 25.4, 22.7. HRMS (ESI-TOF) m/z: calc'd for $C_{12}H_{18}NO$ $[M+H]^+$ 192.1388, found 192.1393. IR (film) cm^{-1}: 3337, 2956, 2929, 2870, 1633, 1579.

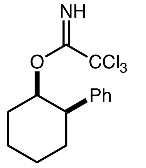

***cis*-2-phenylcyclohexyl 2,2,2-trichloroacetimidate (*cis*-29)**

cis-2-phenylcyclohexanol[1] was subjected to **GP1**. After concentration, the crude mixture was purified (silica gel, hexanes with 1% Et_3N) to yield trichloroacetimidate *cis*-**29** (40 mg, 8%) as a colorless oil. R_f: 0.37 (10% Ethyl acetate/hexanes). ^{1}H NMR (400 MHz, $CDCl_3$) δ: 8.07 (bs, 1H), 7.33 – 7.30 (m, 2H), 7.28 – 7.23 (m, 2H), 7.20 – 7.16 (m, 1H), 5.27 (bs, 1H), 2.87 (dt, *J* = 12.9, 2.9 Hz, 1H), 2.36 – 2.31 (m, 1H), 2.21 (app qd, *J* = 13.0, 3.6 Hz, 1H), 1.98 – 1.93 (m, 1H), 1.84 – 1.80 (m, 1H), 1.72 – 1.57 (m, 3H), 1.54 – 1.43 (m, 1H). ^{13}C NMR (100 MHz, $CDCl_3$) δ: 162.0, 143.1, 128.3, 128.1, 126.5, 92.2, 78.7, 47.2, 29.2, 26.2, 26.1, 20.3.

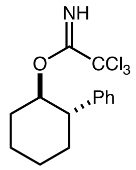

trans-2-phenylcyclohexyl 2,2,2-trichloroacetimidate (_trans_-29)

trans-2-phenylcyclohexanol[1] was subjected to **GP1**. After concentration, the crude mixture was purified (silica gel, hexanes with 1% Et_3N) to yield trichloroacetimidate *trans*-**29** (245 mg, 27%) as a colorless oil. R_f: 0.51 (10% Ethyl acetate/hexanes). ^{1}H NMR (400 MHz, $CDCl_3$) δ: 8.05 (bs, 1H), 7.27 – 7.23 (m, 4H), 7.19 – 7.14 (m, 1H), 5.14 – 5.08 (m, 1H), 2.86 (ddd, J = 12.3, 10.9, 3.8 Hz, 1H), 2.40 – 2.36 (m, 1H), 2.02 – 1.97 (m, 1H), 1.94 – 1.89 (m, 1H), 1.85 – 1.80 (m, 1H), 1.69 – 1.37 (m, 4H). ^{13}C NMR (100 MHz, $CDCl_3$) δ: 162.2, 143.1, 128.3, 127.8, 126.5, 91.9, 81.5, 50.0, 34.0, 30.9, 26.0, 24.8.

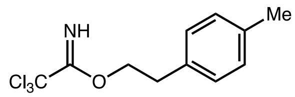

4-methylphenethyl 2,2,2-trichloroacetimidate (S32)

4-methylphenethyl alcohol (719 mg, 0.5 mL, 4.98 mmol) was subjected to **GP1**. Following reaction completion, the solvent was removed *in vacuo* and the crude mixture was purified (silica gel, hexanes with 1% Et_3N) to yield imidate **S32** (0.93 g, 53%) as a colorless solid. R_f: 0.38 (10% Ethyl acetate/hexanes). ^{1}H NMR (400 MHz, $CDCl_3$) δ: 8.27 (bs, 1H), 7.16 (d, J = 7.9 Hz, 2H), 7.11 (d, J = 7.8 Hz, 2H), 4.47 (t, J = 6.9 Hz, 2H), 3.05 (t, J = 6.9 Hz, 2H), 2.32 (s, 3H). ^{13}C NMR (100 MHz, $CDCl_3$) δ: 162.8, 136.1, 134.5, 129.1, 129.0, 91.5, 70.0, 34.3, 21.0. HRMS (ESI-TOF) *m/z*: calc'd for $C_{11}H_{12}Cl_3NONa$ $[M+Na]^+$ 301.9882, found 301.9883. IR (film) cm^{-1}: 3332, 3083, 2957, 1667, 1509, 1341, 1300.

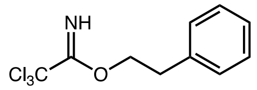

phenethyl 2,2,2-trichloroacetimidate (S33)

4-methoxyphenethyl alcohol (2 g, 1.96 mL, 16.4 mmol) was subjected to **GP1**. Following reaction completion, the solvent was removed *in vacuo* and the crude mixture was purified (silica gel, hexanes with 1% Et_3N) to yield imidate **S33** (4.4 g, 100%) as a colorless solid. R_f: 0.25 (10% Ethyl acetate/hexanes). ^{1}H NMR (400 MHz, $CDCl_3$) δ: 8.34 (bs, 1H), 7.36 – 7.31 (4H, m), 7.29 – 7.26 (m, 1H), 4.54 (t, J = 6.9 Hz, 2H), 3.13 (t, J = 6.9 Hz, 2H).^{13}C NMR (100 MHz, $CDCl_3$) δ: 162.8, 137.7, 129.1, 128.5, 126.7, 91.5, 69.9, 34.8.

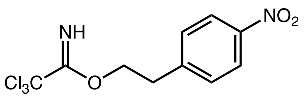

4-nitrophenethyl 2,2,2-trichloroacetimidate (S34)

4-nitrophenethyl alcohol (334 mg, 2 mmol) was subjected to **GP1**. Following reaction completion, the solvent was removed *in vacuo* and the crude mixture was purified (silica gel, hexanes with 1% Et_3N) to yield imidate **S34** (565 mg, 45%) as an off-white solid. R_f: 0.10 (10% Ethyl acetate/hexanes). ^{1}H NMR (400 MHz, $CDCl_3$) δ: 8.33 (bs, 1H), 8.19 – 8.15 (m, 2H), 7.47 – 7.44 (m, 2H), 4.54 (t, J = 6.5 Hz, 2H), 3.20 (t, J = 6.5 Hz, 2H). ^{13}C NMR (100 MHz, $CDCl_3$) δ: 162.7, 147.1, 145.7, 130.1, 123.8, 91.3, 68.8, 34.7. HRMS (ESI-TOF) *m/z*: calc'd for $C_{10}H_9Cl_3N_2O_3Na$ $[M+Na]^+$ 332.9576, found 332.9558. IR (film) cm^{-1}: 3328, 3085, 2963, 1667, 1509, 1341, 1299.

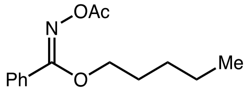

pentyl (*Z*)-*N*-acetoxybenzimidate (S35)

Diethyl azodicarboxylate (297 µL, 2.1 mmol) was added slowly to a solution of N–acetoxybenzamide (340 mg, 1.9 mmol), 1-pentanol (207 µL, 1.9 mmol), and triphenylphosphine (551 mg, 2.1 mmol) in THF (10 mL) at 0 °C. The reaction mixture was then stirred for 1 hour at room temperature. The reaction solvent was removed by evaporation under reduced pressure and the residue was purified by column chromatography on silica gel (20% EtOAc/hexanes) to afford the product as a colorless oil (345 mg, 73%).

R_f: 0.07 (10% Ethyl acetate/hexanes). ^{1}H NMR (400 MHz, $CDCl_3$) δ: 7.71 – 7.69 (m, 2H), 7.49 – 7.36 (m, 3H), 4.20 (t, J = 6.6 Hz, 3H), 2.22 (s, 3H), 1.76 – 1.69 (m, 2H), 1.43 – 1.29 (m, 4H), 0.90 (t, J = 7.1 Hz, 3H). HRMS (ESI-TOF) *m/z*: calc'd for $C_{14}H_{19}NONa_3$ $[M+Na]^+$ 272.1263, found 272.1258. IR (film) cm^{-1}: 2957, 2932, 2871, 1771, 1666, 1613, 1574, 1365, 1318, 1196, 1100.

IV. Optimization

Benzimidate **1** was subjected to **GP5** with the described modifications. After five hours, the mixture was cooled to room temperature, the solvent was removed *in vacuo*, and the crude reaction was analyzed via crude ^{1}H NMR.

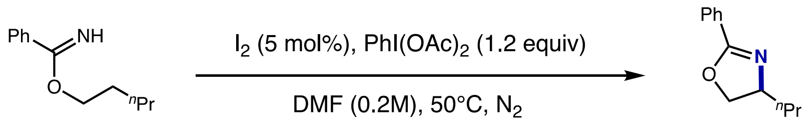

entry	changes from standard conditions	yield (%)[a]
1	none	95
	solvent	
2	CH_2Cl_2 instead of DMF	31
3	PhMe instead of DMF	33
4	MeCN instead of DMF	94
	iodine source	
5	NaI (5 mol%) instead of I_2	67
6	CsI (5 mol%) instead of I_2	76
	initiation	
7	2 x 23 W CFL	50
8	dark, room temperature	40
	atmosphere	
9	air atmosphere	61
	oxidant	
10	NBu_4I (10 mol%), H_2O_2 (2 equiv), MeCN	0
11	NBu_4I (10 mol%), ^{t}BuOOH (2 equiv), MeCN	0
12	I_2 (10 mol%), $K_2S_2O_8$ (1 equiv), DMF	0
	substrate concentration (in DMF)	
13	0.4 M	94
14	0.1 M	91
15	0.05 M	87
	additive	
16	K_3PO_4 (1.2 equiv)	70
17	K_2CO_3 (1.2 equiv)	78
18	$NaHCO_3$ (1.2 equiv)	70

[a] Yields were determined by ^{1}H NMR analysis using 1,2-dichloroethane as an internal standard.

V. Robustness Screen

To determine the tolerance of this reaction in the presence of various functional groups, a robustness screen was conducted. We subjectively chose functional groups that we deemed to be synthetically relevant (alcohols, aldehydes, ketones, alkenes, allyl bromides) or medicinally relevant (heteroarenes, amino acids).[3,4] All additives that were investigated are included below, including those that we were surprised to find were recovered in high yields under both conditions. Only the first five examples are included in the manuscript, since they represent the biggest different between the two conditions. Benzimidate **1** (0.1 mmol) was subjected to **GP5** or the stoichiometric amination procedure,[1] with the addition of 1 equivalent of the corresponding additive (0.1 mmol). After two hours, standard was added to the reaction, an aliquot was removed, and diluted with $CDCl_3$ for ^{1}H NMR analysis. The percent yield of oxazoline and additive remaining were determined with respect to internal standard.

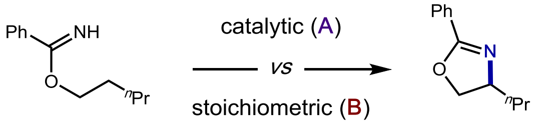

catalytic (A): C-H amination yield, additive remaining

stoichiometric (B): C-H amination yield, additive remaining

Robustness Screen

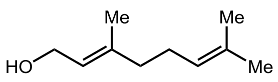
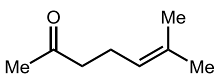
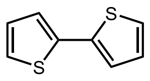
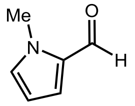

A	**100%,** 95%	**96%,** 93%	**100%,** 92%	**94%,** 70%
B	**34%,** 0%	**35%,** 0%	**70%,** 43%	**82%,** 16%

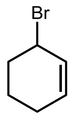
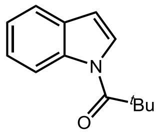
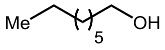
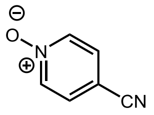

A	**85%,** 35%	**100%**, 88%	**100%**, 100%	**96%**, 93%
B	**0%,** 22%	**54%**, 73%	**85%**, 82%	**76%**, 98%

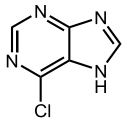
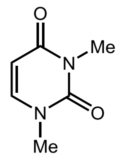
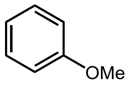
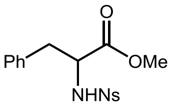

A	**100%**, 86%	**100%**, 97%	**100%**, 100%	**100%**, 100%
B	**77%**, 100%	**71%**, 70%	**91%**, 100%	**74%**, 100%

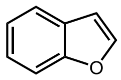
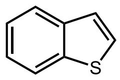
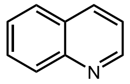
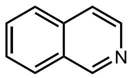

A	**100%**, 100%	**100%**, 100%	**94%**, 70%	**100%**, 69%
B	**82%**, 100%	**86%**, 100%	**87%**, 100%	**100%**, 100%

Yields were determined by ^{1}H NMR analysis using 1,2-dichloroethane as an internal standard.

VI. C-H Amination

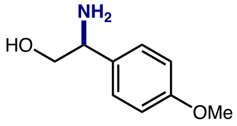

2-amino-2-(4-methoxyphenyl)ethan-1-ol (3)

Trichloroacetimidate **S3** (119 mg, 0.4 mmol) was subjected to **GP5**. Upon reaction completion, the mixture was cooled to room temperature and the solvent was removed *in vacuo*. The crude mixture was subjected to the hydrolysis protocol to yield amino alcohol **3** (67.0 mg, 100%) as a colorless solid. Oxazoline yield by ^{1}H NMR: 97%. R$_f$: 0.13 (30% Isopropanol/dichloromethane). ^{1}H NMR (400 MHz, CD_3CN) δ: 7.28 – 7.26 (m, 2H), 6.88 – 6.86 (m, 2H), 3.86 (dd, *J* = 8.2, 4.6 Hz, 1H), 3.76 (s, 3H), 3.52 (dd, *J* = 10.5, 4.6 Hz, 1H), 3.33 (dd, *J* = 10.5, 8.2 Hz, 1H). ^{13}C NMR (100 MHz, CD_3CN) δ: 159.7, 137.1, 128.8, 114.5, 69.1, 57.8, 55.8.

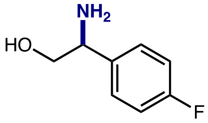

2-amino-2-(4-fluorophenyl)ethan-1-ol (4)

Trichloroacetimidate **S4** (114 mg, 0.4 mmol) was subjected to **GP5**. Upon reaction completion, the mixture was cooled to room temperature and the solvent was removed *in vacuo*. The crude mixture was subjected to the hydrolysis protocol to yield amino alcohol **4** (47.1 mg, 77%) as a colorless solid. Oxazoline yield by ^{1}H NMR: 99%. R$_f$: 0.20 (30% Isopropanol/dichloromethane). ^{1}H NMR (400 MHz, CD_3CN) δ: 7.39 – 7.36 (m, 2H), 7.11 – 7.04 (m, 2H), 3.94 (bs, 1H), 3.55 – 3.52 (m, 1H), 3.38 – 3.34 (m, 1H). ^{13}C NMR (100 MHz, CD_3CN) ^{19}F NMR (564 MHz, CD_3CN) δ: –112.8 (m, *J* = 4.9 Hz).

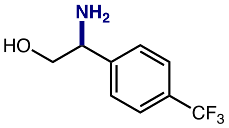

2-amino-2-(4-(trifluoromethyl)phenyl)ethan-1-ol (5)

Trichloroacetimidate **S5** (134 mg, 0.4 mmol) was subjected to **GP5**. Upon reaction completion, the mixture was cooled to room temperature and the solvent was removed *in vacuo*. The crude mixture was subjected to the hydrolysis protocol to yield amino alcohol **5** (45.1 mg, 90%) as a off-white solid. Oxazoline yield by ^{1}H NMR: 91%. ^{1}H NMR (600 MHz, $CDCl_3$) δ: 7.61 (d, *J* = 8.0 Hz, 2H), 7.47 (m, *J* = 8.3 Hz, 2H), 4.14 (dd, *J* = 7.7, 4.4 Hz, 1H), 3.80 (dd, *J* = 10.8, 4.4 Hz, 1H), 3.60 (dd, *J* = 10.7, 7.9 Hz, 1H), 1.89 (bs, 3H). ^{13}C NMR (150 MHz, $CDCl_3$) δ 147.0, 130.0 ($^2J_{CF}$ = 32.5 Hz), 127.1, 125.8 ($^3J_{CF}$ = 3.7 Hz), 124.3 ($^1J_{CF}$ = 271.1 Hz), 68.1, 57.2. ^{19}F NMR (564 MHz, $CDCl_3$) δ: –62.5.

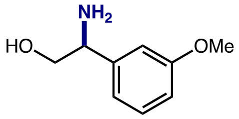

2-amino-2-(*m*-methoxyphenyl)ethan-1-ol (6)

Trichloroacetimidate **S6** (119 mg, 0.4 mmol) was subjected to **GP5**. Upon reaction completion, the mixture was cooled to room temperature and the solvent was removed *in vacuo*. The crude mixture was subjected to the hydrolysis protocol to yield amino alcohol **6** (46.0 mg, 69%) as a colorless solid. Oxazoline yield by ^{1}H NMR: 96%. R_f: 0.10 (30% ^{i}PrOH in CH_2Cl_2). ^{1}H NMR (600 MHz, $CDCl_3$) δ: 7.29 – 7.27 (m, 1H), 6.92 – 6.90 (m, 2H), 6.83 – 6.82 (m, 1H), 4.02 (dd, *J* = 8.1, 4.3 Hz, 1H), 3.82 (s, 3H), 3.74 (dd, *J* = 10.8, 4.3 Hz, 1H), 3.56 (dd, *J* = 10.8, 8.2 Hz, 1H), 2.40 (bs, 3H). ^{13}C NMR (150 MHz, $CDCl_3$) δ: 159.9, 144.5, 129.7, 118.9, 112.8, 112.4, 68.0, 57.5, 55.3.

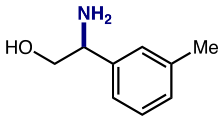

2-amino-2-(*m*-tolyl)ethan-1-ol (7)

Trichloroacetimidate **S7** (119 mg, 0.4 mmol) was subjected to **GP5**. Upon reaction completion, the mixture was cooled to room temperature and the solvent was removed *in vacuo*. The crude mixture was subjected to the hydrolysis protocol to yield amino alcohol **7** (67.0 mg, 83%) as a colorless solid. Oxazoline yield by ^{1}H NMR: 93%. R_f: 0.26 (10% MeOH in CH_2Cl_2). ^{1}H NMR (400 MHz, $CDCl_3$) δ: 7.24 (t, *J* = 7.6 Hz, 1H), 7.14 – 7.08 (m, 3H), 4.00 (dd, *J* = 8.1, 4.4 Hz, 1H), 3.72 (dd, *J* = 10.8, 4.5 Hz, 1H), 3.54 (dd, *J* = 10.7, 8.3 Hz, 1H), 2.35 (s, 3H), 2.06 (bs, 3H). ^{13}C NMR (100 MHz, $CDCl_3$) δ: 142.9, 138.4, 128.7, 128.4, 127.3, 123.6, 68.2, 57.4, 21.6.

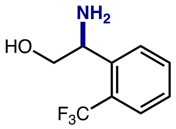

2-amino-2-(2-(trifluoromethyl)phenyl)ethan-1-ol (8)

Trichloroacetimidate **S8** was subjected to **GP5**. Upon reaction completion, the mixture was cooled to room temperature and the solvent was removed *in vacuo*. The crude mixture was subjected to the hydrolysis protocol to yield amino alcohol **8** (72 mg, 88%) as a white solid. Oxazoline yield by ^{1}H NMR: 95%. R_f: 0.31 (30% isopropanol/CH_2Cl_2). ^{1}H NMR (400 MHz, $CDCl_3$) δ: 7.68 (d, *J* = 7.9 Hz, 1H), 7.65 (d, *J* = 7.9 Hz, 1H), 7.56 (d, *J* = 7.6 Hz, 1H), 7.37 (d, *J* = 7.6 Hz, 1H), 4.47 (dd, *J* = 7.9, 3.4 Hz, 1H), 3.75 (dd, *J* = 10.8, 4.0 Hz, 1H), 3.57 (dd, *J* = 10.7, 8.3 Hz, 1H), 2.12 (bs, 3H). ^{13}C NMR (150 MHz, $CDCl_3$) δ: 142.0, 132.4, 128.1 (q, $^{2}J_{CF}$ = 29.8 Hz), 127.9, 127.5, 126.0 (q, $^{3}J_{CF}$ = 5.9 Hz), 124.5 (q, $^{1}J_{CF}$ = 274.0 Hz), 67.4, 52.5 (q, $^{4}J_{CF}$ = 2.2 Hz).

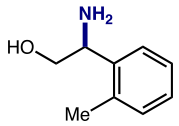

2-amino-2-(*o*-tolyl)ethan-1-ol (9)

Trichloroacetimidate **S9** was subjected to **GP5**. Upon reaction completion, the mixture was cooled to room temperature and the solvent was removed *in vacuo*. The crude mixture was subjected to the hydrolysis protocol to yield amino alcohol **9** (47 mg, 87%) as a white solid. Oxazoline yield by ^{1}H NMR: 100%. R_f: 0.08 (30% isopropanol/CH_2Cl_2). ^{1}H NMR (400 MHz, $CDCl_3$) δ: 7.38 (d, *J* = 7.4 Hz, 1H), 7.25 – 7.15 (m, 3H), 4.30 (dd, *J* = 8.0, 4.0 Hz, 1H), 3.70 (dd, *J* = 10.3, 3.9 Hz, 1H), 3.51 (dd, *J* = 10.8, 8.4 Hz, 1H), 2.37 (s, 3H), 2.02 (bs, 3H). ^{13}C NMR (100 MHz, $CDCl_3$) δ: 140.9, 135.4, 130.7, 127.3, 126.5, 125.3, 67.1, 53.1, 19.4.

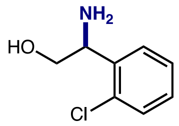

2-amino-2-(2-chlorophenyl)ethan-1-ol (10)

Trichloroacetimidate **S10** was subjected to **GP5**. Upon reaction completion, the mixture was cooled to room temperature and the solvent was removed *in vacuo*. The crude mixture was subjected to the hydrolysis protocol to yield amino alcohol **10** (52 mg, 82%) as a white solid. Oxazoline yield by ^{1}H NMR: 95%. R_f: 0.17 (30% isopropanol/CH_2Cl_2). ^{1}H NMR (400 MHz, $CDCl_3$) δ: 7.47 (dd, *J* = 7.7, 1.7 Hz, 1H), 7.36 (dd, *J* = 7.9, 1.3 Hz, 1H), 7.31 – 7.27 (m, 1H), 7.21 (td, *J* = 7.5, 1.7 Hz, 1H), 4.51 (dd, *J* = 7.8, 4.0 Hz, 1H), 3.83 (dd, *J* = 10.7, 4.0 Hz, 1H), 3.57 (dd, *J* = 10.7, 7.8 Hz, 1H), 1.85 (bs, 3H). ^{13}C NMR (100 MHz, $CDCl_3$) δ: 140.1, 133.2, 129.9, 128.6, 127.5, 127.3, 66.2, 53.8.

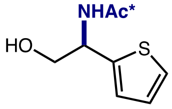

2-amino-2-(thiophen-2-yl)ethan-1-ol (11)

Trichloroacetimidate **S11** (109 mg, 0.4 mmol) was subjected to **GP5** with the following changes. To a 2-dram vial equipped with PTFE septa cap and magnetic stir bar was added imidate (0.4 mmol, 1 equiv.), $PhI(OAc)_2$ (258 mg, 0.8 mmol, 2 equiv.). This vial was evacuated and backfilled with N_2 (3x). A degassed stock solution of I_2 in dry DMF (2 mL, 0.01 M, 0.05 equiv.) was added to the vial under N_2. The reaction was heated to 50°C (by placing vial in an aluminum heating block) and stirred for 4 hours. *Hydrolysis and acid/base extraction.* Upon completion, the mixture was diluted with H_2O (1 mL) and *p*-toluenesulfonic acid monohydrate (2.0 mmol, 5 equiv) was added and the reaction was allowed to stir for 1 hr. Upon completion, the reaction was poured into

a separatory funnel containing a 1:1 mixture of saturated $NaHCO_3$ and 20% $Na_2S_2O_3$ (15 mL). The aqueous phase was washed with ethyl acetate (3 x 15 mL) and the combined organic solution was dried over $MgSO_4$ and concentrated. The crude material was purified (silica gel, Hexanes → 10% ethyl acetate/hexanes) to yield trichloroacetamide **11** (80 mg, 69%) as a white solid. Oxazoline yield by ^{1}H NMR: 96%. R_f: 0.49 (50% ethyl acetate in hexanes). ^{1}H NMR (400 MHz, $CDCl_3$) δ: 7.37 (bs, 1H), 7.30 (dd, *J* = 5.1, 1.2 Hz, 1H), 7.08 (td, *J* = 3.5, 1.0 Hz, 1H), 7.02 (dd, *J* = 3.5, 5.1 Hz, 1H), 5.38 – 5.34 (m, 1H), 4.06 (dd, *J* = 6.1, 4.0 Hz, 1H), 1.90 (t, *J* = 6.1 Hz, 1H). ^{13}C NMR (100 MHz, $CDCl_3$) δ: 161.7, 140.4, 127.4, 125.8, 125.7, 92.6, 65.4, 52.9.

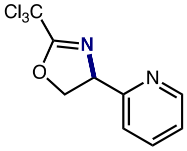

4-(pyridin-2-yl)-2-(trichloromethyl)-4,5-dihydrooxazole (12)

Trichloroacetimidate **S12** (107 mg, 0.4 mmol) was subjected to **GP5** with the following changes. To a 2-dram vial equipped with PTFE septa cap and magnetic stir bar was added imidate (107 mg, 0.4 mmol, 1 equiv.), $PhI(OAc)_2$ (258 mg, 0.8 mmol, 2 equiv.) and I_2 (51 mg, 0.2 mmol, 0.5 equiv). This vial was quickly evacuated and backfilled with N_2 (3x). A freeze-pump-thawed dry MeCN (2 mL) was added to the vial under N_2. The reaction was heated to 50 °C and stirred for 4 hours under inert atmosphere. Upon completion, the reaction was diluted with CH_2Cl_2 (10 mL) and aqueous $Na_2S_2O_3$ (10% w/w) was added. The aqueous layer was washed with CH_2Cl_2 (3 x 25 mL). The combined organic layer was dried over $MgSO_4$ and concentrated. The crude product was purified (silica gel, Hexanes → 50% ethyl acetate/hexanes) to yield oxazoline **12** (75 mg, 71%) as a yellow oil. Oxazoline yield by ^{1}H NMR: 70%. R_f: 0.46 (50% ethyl acetate in hexanes). ^{1}H NMR (400 MHz, $CDCl_3$) δ: 8.56 (ddd, *J* = 4.8, 1.7, 0.9 Hz, 1H), 7.71 (td, *J* = 11.6, 1.8 Hz, 2H), 7.43 (d, *J* = 7.9 Hz, 2H), 7.14 (m, 1H), 5.59 (dd, *J* = 10.5, 8.1 Hz, 1H), 5.05 (dd, *J* = 10.3, 8.6 Hz, 1H), 4.87 (t, *J* = 8.4 Hz, 1H). ^{13}C NMR (100 MHz, $CDCl_3$) δ: 164.3, 159.2, 150.0, 137.3, 123.1, 121.7, 86.7, 76.7, 71.1. HRMS (ESI): m/z calculated for $C_9H_7Cl_3N_2ONa$ $[M+Na]^+$ 286.9522, found 286.9523. IR (film) cm^{-1}: 3010, 1733, 1658, 1590, 1571, 1472, 1435, 1351, 1230, 992.

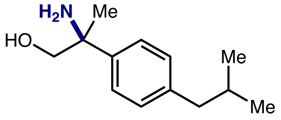

2-amino-2-(4-isobutylphenyl)propan-1-ol (13)

Trichloroacetimidate **S13** was subjected to **GP5**. Upon reaction completion, the mixture was cooled to room temperature and the solvent was removed *in vacuo*. The crude mixture was subjected to the hydrolysis protocol to yield amino alcohol **13** (65 mg, 79%) as a white solid. Oxazoline yield by ^{1}H NMR: 85%. R_f: 0.1 (30% isopropanol/CH_2Cl_2). ^{1}H NMR (400 MHz, $CDCl_3$) δ: 7.33 (d, *J* = 8.2 Hz, 2H), 7.13 (d, *J* = 8.1 Hz, 2H), 3.60 (bs, 2H), 2.45 (d, *J* = 7.3 Hz, 2H), 2.12 (bs, 3H), 1.90 – 1.80 (m, 1H), 1.44 (s, 3H), 0.9 (d, *J* = 7.3 Hz, 1H). ^{13}C NMR (150 MHz, $CDCl_3$) δ: 143.8, 140.4, 129.3, 125.1, 72.0, 56.2, 45.1, 30.3, 27.3, 22.6.

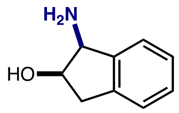

1-amino-2,3-dihydro-1*H*-inden-2-ol (14)

Trichloroacetimidate **S14** was subjected to **GP5**. Upon reaction completion, the mixture was cooled to room temperature and the solvent was removed *in vacuo*. The crude mixture was subjected to the hydrolysis protocol to yield amino alcohol **14** (46 mg, 78%) as a white solid. Oxazoline yield by ^{1}H NMR: 89%. R_f: 0.05 (30% isopropanol/CH_2Cl_2). ^{1}H NMR (400 MHz, $CDCl_3$) δ: 7.31 – 7.29 (m, 1H), 7.27 – 7.23 (m, 3H), 4.38 (bs, 1H), 4.33 (bs, 1H), 3.10 (dd, *J* = 16.4, 5.5 Hz, 1H), 2.95 (dd, *J* = 16.4, 2.7 Hz, 1H), 2.31 (bs, 3H). ^{13}C NMR (150 MHz, $CDCl_3$) δ: 144.1, 141.1, 128.1, 127.1, 125.6, 124.0, 72.8, 58.6, 39.5.

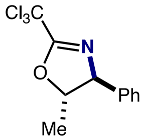

5-methyl-4-phenyl-2-(trichloromethyl)-4,5-dihydrooxazole (15)

Trichloroacetimidate **S15** was subjected to **GP5**. Analysis of the crude ^{1}H NMR (with 1 equiv. DCE standard) indicates 88% of the target oxazoline **15** (3:1 d.r.). R_f: (major) 0.39 (minor) 0.33 (10% ethyl acetate/hexanes). ^{1}H NMR (400 MHz, $CDCl_3$): *major diastereomer* δ: 7.39 – 7.37 (m, 2H), 7.34 – 7.31 (m, 1H), 7.25 – 7.24 (m, 2H), 4.88 – 4.82 (m, 2H), 1.59 (d, *J* = 6.1 Hz, 3H). *Minor diastereomer* δ: 7.42 – 7.29 (m, 3H), 7.19 – 7.17 (m, 2H), 5.47 (d, *J* = 9.6 Hz, 1H), 5.33 (dq, *J* = 9.6, 6.6 Hz, 1H), 1.01 (d, *J* = 6.6 Hz, 3H). ^{13}C NMR (150 MHz, $CDCl_3$) δ: 162.9, 140.1, 129.1, 128.4, 126.6, 88.4, 20.6.

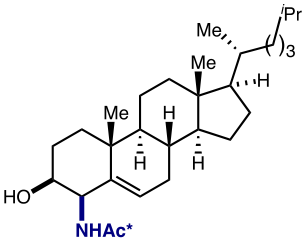

4- trichloroacetamidyl cholesterol (16)

Trichloroacetimidate **S16** (212 mg, 0.4 mmol) was subjected to **GP5** with the following changes. To a 2-dram vial equipped with PTFE septa cap and magnetic stir bar was added imidate (0.4 mmol, 1 equiv.) and $PhI(OAc)_2$ (258 mg, 0.8 mmol, 2 equiv.) This vial was evacuated and backfilled with N_2 (3x). A degassed stock solution of I_2 in dry DMF (2 mL, 0.01 M, 0.05 equiv.) was added to the vial under N_2. The reaction was heated to 50°C (by placing vial in an aluminum heating block) and stirred for 4 hours. *Hydrolysis and acid/base extraction.* Upon completion, the mixture was concentrated in a round-bottom flask, then methanol (4 mL), 2M HCl (0.8 mL), tetrabutylammonium chloride (111 mg, 0.4 mmol) were added. After stirring for 24 hours, the

reaction was diluted with CH_2Cl_2 (10 mL) and aqueous $Na_2S_2O_3$(10% w/w) was added. The aqueous layer was washed with CH_2Cl_2 (3 x 25 mL). The combined organic layer was dried over $MgSO_4$ and concentrated. The crude product was purified (silica gel, Hexanes → 20% ethyl acetate/hexanes) to yield trichloroacetamide **16** (166 mg, 76%) as a white solid. Oxazoline yield by ^{1}H NMR: 100%. R_f: 0.26 (10% MeOH in CH_2Cl_2). ^{1}H NMR (400 MHz, $CDCl_3$) δ: 7.07 (bs, 1H), 5.89 – 5.87 (m, 1H), 4.48 – 4.45 (m, 1H), 3.87 – 3.83 (m, 1H), 2.20 – 2.13 (m, 2H), 2.05 – 2.00 (m, 1H), 1.88 – 1.80 (m, 3H), 1.75 – 1.57 (m, 4H), 1.53 – 1.11 (m, 15H), 0.91 (d, *J* = 6.6 Hz, 3H), 0.87 (d, *J* = 2.3 Hz, 3H), 0.86 (d, *J* = 1.8 Hz, 3H), 0.67 (s, 3H). ^{13}C NMR (100 MHz, $CDCl_3$) δ: 162.5, 137.9, 131.7, 93.1, 71.6, 60.9, 57.0, 56.2, 50.4, 42.3, 39.7, 39.6, 36.3, 36.1, 35.9, 32.3, 31.8, 28.3, 28.2, 25.7, 24.3, 24.0, 23.0, 22.7, 21.0, 20.6, 18.9, 11.9.

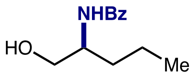

N-(1-hydroxypentan-2-yl)benzamide (17)

Benzimidate **1 (**77 mg, 0.4 mmol) was subjected to **GP5**. Upon reaction completion, the mixture was cooled to room temperature and the solvent was removed *in vacuo*. The crude mixture was subjected to the hydrolysis protocol to yield amino alcohol **17** (72.2 mg, 95%) as a colorless solid. Oxazoline yield by ^{1}H NMR: 95%. R_f: 0.63 (10% Methanol/dichloromethane). ^{1}H NMR (400 MHz, CD_3CN) δ: 7.81 – 7.79 (m, 2H), 7.53 – 7.50 (m, 1H), 7.47 – 7.44 (m, 2H), 6.79 (bs, 1H), 4.08 – 4.02 (m, 1H), 3.58 – 3.52 (m, 2H), 3.01 (t, *J* = 6.0 Hz, 1H) 1.62 – 1.33 (m, 4H), 0.93 (t, *J* = 7.3 Hz, 3H). ^{13}C NMR (100 MHz, CD_3CN) δ: 168.2, 136.1, 132.1, 129.4, 65.3, 52.8, 34.0, 20.1, 14.3.

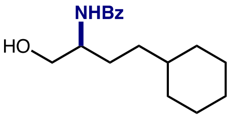

***N*-(4-cyclohexyl-1-hydroxybutan-2-yl)benzamide (18)**

Benzimidate **S18** (104 mg, 0.4 mmol) was subjected to **GP5**. Upon reaction completion, the mixture was cooled to room temperature and the solvent was removed *in vacuo*. The crude mixture was subjected to the hydrolysis protocol to yield amino alcohol **18** (90.4 mg, 82%) as a colorless solid. Oxazoline yield by ^{1}H NMR: 95%. R_f: 0.69 (10% Methanol/dichloromethane). ^{1}H NMR (400 MHz, CD_3CN) δ: 7.74 (bs, 3H), 7.49 – 7.38 (m, 3H), 4.25 (bs, 2H), 1.87 – 1.61 (m, 8H), 1.39 – 1.09 (m, 7H), 0.99 – 0.84 (m, 2H). ^{13}C NMR (150 MHz, CD_3CN) δ: 168.0, 136.1, 132.1, 129.4, 128.1, 65.8, 50.4, 39.5, 35.2, 34.7, 33.5, 27.3, 27.1, 27.0. HRMS (ESI-TOF) *m/z*: calc'd for $C_{17}H_{25}NO_2Na$ $[M+Na]^+$ 284.1626, found 284.1607. IR (film) cm^{-1}: 3213, 3075, 2924, 2850, 1634, 1553.

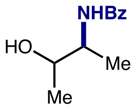

N-(3-hydroxybutan-2-yl)benzamide (19)

Benzimidate **S19** (134 mg, 0.4 mmol) was subjected to **GP5**. Upon reaction completion, the mixture was cooled to room temperature and the solvent was removed *in vacuo*. The crude mixture was subjected to the hydrolysis protocol to yield amino alcohol **19** (75.3 mg, 97%, 1:1 d.r.) as a colorless solid that was an inseparable mixture of diastereomers. Oxazoline yield by ^{1}H NMR: 99%. R_f: 0.56 (10% Methanol/dichloromethane). ^{1}H NMR (600 MHz, CD_3CN), diastereomer A δ: 7.80 – 7.79 (m, 2H), 7.54 – 7.51 (m, 1H), 7.47 – 7.44 (m, 2H), 6.86 (bs, 1H), 4.05 – 3.96 (m, 1H), 3.82 – 3.87 (m, 1H), 3.21 (d, J = 5.4 Hz, 1H), 1.19 (d, J = 6.9 Hz, 3H), 1.12 (d, J = 6.4 Hz, 3H). diastereomer B δ: 7.80 – 7.79 (m, 2H), 7.54 – 7.51 (m, 1H), 7.47 – 7.44 (m, 2H), 6.78 (bs, 1H), 4.05 – 3.96 (m, 1H), 3.78 – 3.73 (m, 1H), 3.09 (d, J = 5.4 Hz, 1H), 1.17 (d, J = 6.9 Hz, 3H), 1.12 (d, J = 6.4 Hz, 3H). ^{13}C NMR (150 MHz, CD_3CN) δ: 167.93, 167.88, 136.1, 136.0, 132.20, 133.16, 129.39, 129.37, 128.1, 128.0, 70.7, 52.2, 52.0, 20.5, 19.7, 17.6, 15.1.

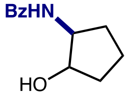

N-(2-hydroxycyclopentyl)benzamide (20)

Benzimidate **S20** (75.7 mg, 0.4 mmol) was subjected to **GP5**. Upon reaction completion, the mixture was cooled to room temperature and the solvent was removed *in vacuo*. The crude mixture was subjected to the hydrolysis protocol to yield amino alcohol **20** (51.7 mg, >20:1 d.r., 63%) as a colorless solid. Oxazoline yield by ^{1}H NMR: 70%. R_f: 0.63 (10% Methanol/dichloromethane). ^{1}H NMR (400 MHz, CD_3CN) δ: 7.83 – 7.80 (m, 2H), 7.54 – 7.50 (m, 1H), 7.48 – 7.43 (m, 2H), 6.92 (bs, 1H), 4.19 – 4.11 (m, 2H), 2.01 – 1.77 (m, 3H), 1.73 – 1.52 (m, 3H). ^{13}C NMR (100 MHz, CD_3CN) δ: 167.7, 136.0, 132.1, 129.4, 128.0, 72.8, 55.6, 33.4, 29.5, 21.1.

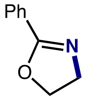

2-phenyl-4,5-dihydrooxazole (21)

Benzimidate **S21** (59.7 mg, 0.4 mmol) was subjected to **GP5**. Upon reaction completion, the mixture was cooled to room temperature and the solvent was removed *in vacuo*. The crude NMR yield showed 48% amination, but the oxazoline proved volatile when DMF was being removed on rotary evaporator. Oxazoline **21** yield by ^{1}H NMR: 48%.

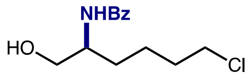

***N*-(6-chloro-1-hydroxyhexan-2-yl)benzamide (22)**

Benzimidate **S22** (96 mg, 0.4 mmol) was subjected to **GP5**. Upon reaction completion, the mixture was cooled to room temperature and the solvent was removed *in vacuo*. The crude mixture was subjected to the hydrolysis protocol to yield amino alcohol **22** (91.7 mg, 90%) as an off-white solid. Oxazoline yield by ^{1}H NMR: 92%. R_f: 0.63 (10% Methanol/dichloromethane). ^{1}H NMR (400 MHz, CD_3CN) δ: 7.82 (m, 2H), 7.54 – 7.51 (m, 1H), 7.47 – 7.44 (m, 1H), 6.81 (bs, 1H), 4.10 – 4.02 (m, 1H), 3.59 (t, J = 6.7 Hz, 2H), 3.58 – 3.53 (m, 2H), 3.01 (t, J = 5.9 Hz, 1H), 1.87 – 1.73, (m, 2H), 1.70 – 1.62 (m, 1H), 1.59 – 1.42 (m, 3H). ^{13}C NMR (100 MHz, CD_3CN) δ: 168.1, 136.1, 132.2, 129.4, 128.1, 65.2, 52.8, 46.1, 33.2, 31.1, 24.2. HRMS (ESI-TOF) *m/z*: calc'd for $C_{13}H_{18}ClNO_2Na$ [M+Na]$^+$ 278.0924, found 278.0909. IR (film) cm^{-1}: 3305, 3245, 3081, 2921, 1728, 1637.

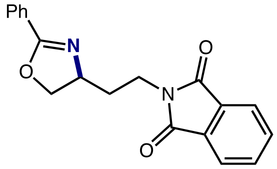

2-(2-(2-phenyl-4,5-dihydrooxazol-4-yl)ethyl)isoindoline-1,3-dione (23)

Benzimidate **S23** (64.5 mg, 0.2 mmol) was subjected to **GP5**. Upon reaction completion, the mixture was cooled to room temperature and the solvent was removed *in vacuo*. The crude mixture was purified via column chromatography (silica gel, 50% Ethyl acetate/hexanes) to give oxazoline **23** (65.1 mg, 100%) as a colorless solid. Oxazoline yield by ^{1}H NMR: 100%. R_f: 0.47 (50% Ethyl acetate/hexanes). ^{1}H NMR (400 MHz, CD_3CN) δ: 7.82 – 7.77 (m, 4H), 7.66 – 7.64 (m, 2H), 7.48 – 7.45 (m, 1H), 7.35 – 7.33 (m, 2H), 4.52 – 4.49 (m, 1H), 4.32 – 4.26 (m, 1H), 4.03 (t, J = 8.2 Hz, 1H), 3.92 – 3.87 (m, 1H), 3.79 – 3.75 (m, 1H), 2.14 (bs, 1H), 1.97 – 1.95 (m, 1H). ^{13}C NMR (100 MHz, CD_3CN) δ: 169.5, 164.0, 135.0, 133.5, 132.2, 129.4, 128.9, 128.8, 123.8, 73.3, 66.0, 36.3, 34.8.

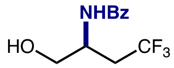

***N*-(4,4,4-trifluoro-1-hydroxybutan-2-yl)benzamide (24)**

Benzimidate **S24** (96 mg, 0.4 mmol) was subjected to **GP5**. Upon reaction completion, the mixture was cooled to room temperature and the solvent was removed *in vacuo*. The crude mixture was subjected to the hydrolysis protocol to yield amino alcohol **24** (46.6 mg, 47%) as a yellow solid. Oxazoline yield by ^{1}H NMR: 77%. R_f: 0.63 (10% Methanol/dichloromethane). ^{1}H NMR (400 MHz, CD_3CN) δ: 7.81 – 7.78 (m, 2H), 7.56 – 7.52 (m, 1H), 7.49 – 7.45 (m, 2H), 7.04 (bs, 1H), 4.45 – 4.37 (m, 1H), 3.69 – 3.54 (m, 2H), 3.18 (bs, 1H), 2.65 – 2.47 (m, 2H). ^{13}C NMR (100 MHz, CD_3CN) δ: 167.7, 135.6, 132.4, 129.5, 128.1, 128.0 (q, $^{1}J_{CF}$ = 276.1 Hz), 64.2, 47.5 (q, $^{3}J_{CF}$ = 3.8

Hz), 39.6 (q, ${}^2J_{CF}$ = 27.6 Hz). ^{19}F NMR (564 MHz, CD_3CN) δ: –59.2 (t, J = 11.2 Hz). HRMS (ESI) *m/z*: calc'd for $C_{11}H_{12}F_3NO_2Na$ $[M+Na]^+$ 270.0718, found 270.0713. IR (film) cm^{-1}: 3370, 3296, 3070, 2918, 2850, 1637, 1537.

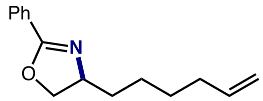

4-(hex-5-en-1-yl)-2-phenyl-4,5-dihydrooxazole (25)

Benzimidate **S25** (23 mg, 0.1 mmol) was subjected to **GP5**. Upon reaction completion, the mixture was cooled to room temperature and the solvent was removed *in vacuo*. The crude mixture was purified via column chromatography (silica gel, 10% EtOAc, Hexanes) to give oxazoline **25** (17.4 mg, 76%) as a colorless oil. Oxazoline yield by ^{1}H NMR: 88%. R_f: 0.38 (20% Ethyl acetate/hexanes). ^{1}H NMR (400 MHz, CD_3CN) δ: 7.95 – 7.93 (m, 2H), 7.49 – 7.44 (m, 1H), 7.42 – 7.38 (m, 2H), 5.81 (ddt, J = 17.1, 10.2, 6.7 Hz, 1H), 5.03 – 4.93 (m, 2H), 4.48 (dd, J = 9.4, 8.1 Hz, 1H), 4.33 – 4.21 (m, 1H), 4.02 (t, J = 7.9 Hz, 1H), 2.11 – 2.06 (m, 2H), 1.81 – 1.71 (m,1 H), 1.61 –1.36 (m, 5H); ^{13}C NMR (100 MHz, CD_3CN) δ: 163.5, 138.9, 131.3, 128.41, 128.35, 128.1, 114.6, 72.7, 66.9, 36.0, 33.8, 29.0, 25.5. HRMS (ESI-TOF) *m/z*: calc'd for $C_{15}H_{20}NO$ $[M+H]^+$ 230.1545, found 230.1545.

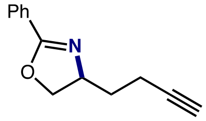

4-(but-3-yn-1-yl)-2-phenyl-4,5-dihydrooxazole (26)

Benzimidate **S26** (20 mg, 0.1 mmol) was subjected to **GP5**. Upon reaction completion, the mixture was cooled to room temperature and the solvent was removed *in vacuo*. The crude mixture was purified via column chromatography (silica gel, 10% EtOAc/hexanes) to give oxazoline **26** (11.2 mg, 56%) as a colorless oil. Oxazoline yield by ^{1}H NMR: 63%. R_f: 0.38 (20% Ethyl acetate/hexanes). ^{1}H NMR (400 MHz, CD_3CN) δ: 7.95 – 7.93 (m, 2H), 7.49 –7. 45 (m, 1H), 7.42 – 7.38 (m, 2H), 4.53 (dd, J = 9.5, 8.2 Hz, 1H), 4.45 – 4.37 (m, 1H), 4.09 (t, J = 7.9 Hz, 1H), 2.41 (td, J = 7.2, 2.6 Hz, 2H). ^{13}C NMR (100 MHz, CD_3CN) δ: 164.0, 131.5, 128.44, 128.41, 127.9, 83.8, 72.5, 68.9, 65.9, 34.9, 29.8, 15.6. HRMS (ESI-TOF) *m/z*: calc'd for $C_{13}H_{14}NO$ $[M+H]^+$ 200.1075, found 200.1086. IR (film) cm^{-1}: 3308, 3225, 3086, 2929, 2851, 2244, 1731, 1627.

VI I. Mechanistic Studies

a. Time Studies

To study product formation over the course of the reaction, side-by-side reactions of the amination of pentyl benzimidate **1** were quenched at time points and analyzed by ^{1}H NMR.

Catalytic Amination Procedure:

To obtain this data, pentyl benzimidate **1** was subjected to a modified **GP5**.

To a 2-dram vial equipped with PTFE septa cap and magnetic stir bar was added imidate **1** (0.4 mmol, 1 equiv.), $PhI(OAc)_2$ (154.6 mg, 0.48 mmol, 1.2 equiv.). This vial was evacuated and backfilled with N_2 (3x). 2 mL of a degassed stock solution of I_2 in dry DMF (5 mol%, 10 mol%, or 20 mol%) was added to the vial under N_2. The reaction was heated to 50°C (by placing vial in an aluminum heating block). At the indicated time points, the reaction was quenched with 10% aqueous $Na_2S_2O_3$ (2 mL) and then extracted with Et_2O (4 x 4 mL). The crude reaction was concentrated and analyzed by ^{1}H NMR using an internal standard, dichloroethane (31.6 μL, 1 equiv.).

Stoichiometric amination procedure was conducted according to ref. 1.

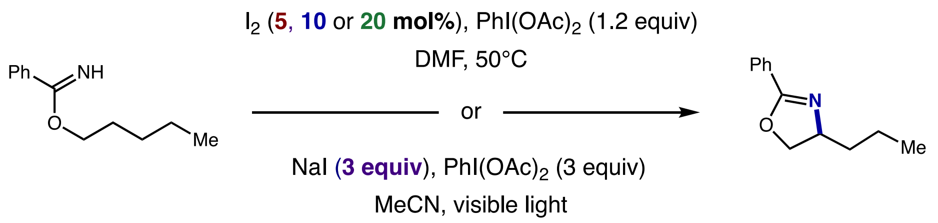

Time (minutes)	Yield (%)			
	Stoich.	I_2 (5 mol%)	I_2 (10 mol%)	I_2 (20 mol%)
2	–	27	43	50
4	–	38	55	65
6	–	46	60	70
8	–	53	66	80
10	10	64	70	85
30	25	80	86	90
50	53	93	90	96

Table S1: Crude percent yield of oxazoline determined by ^{1}H NMR.

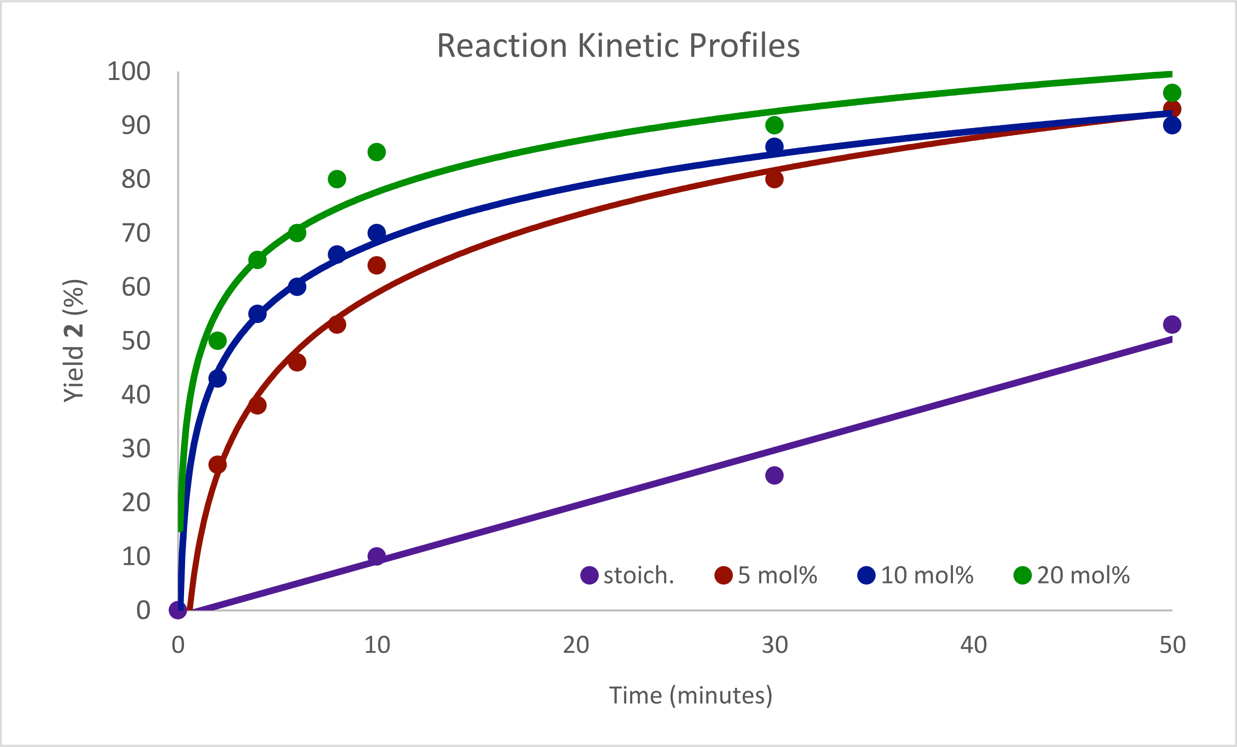

Figure S1: Kinetic profiles of various reaction conditions determined by ^{1}H NMR.

b. HAT Selectivity

To probe the selectivity of HAT with various electronic perturbations, we synthesized two benzimidate substrates with γ-benzylic and γ-tertiary hydrogen atoms. These substrates were subjected to both the catalytic conditions, and our previously reported stoichiometric conditions. In both cases, more efficient amination was observed in the catalytic system. Using superstiochiometric NaI and $PhI(OAc)_2$ leads to competitive formation of a geminal di-iodide product—suppressing the amination yield.

1. β vs γ competition: secondary vs benzylic C-H

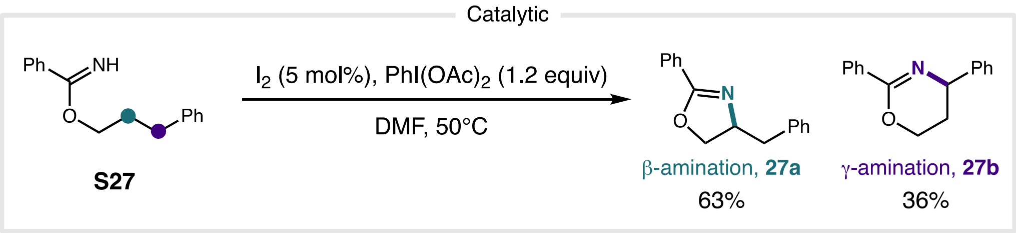

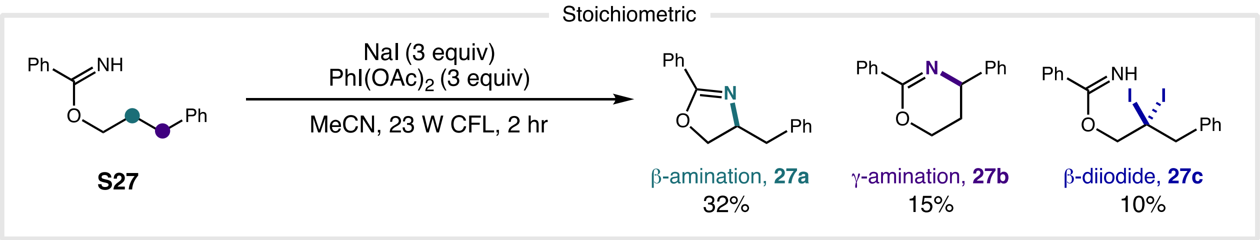

Catalytic: Imidate **S27** was subjected to **GP5**. Analysis of the crude ^{1}H NMR (with 1 equiv. DCE standard) indicates 63% of the desired oxazoline **27a** with 36% of the corresponding γ aminated product **27b**.

Stoichiometric: Imidate **S27** was subjected to the stoichiometric reaction conditions.[1] Analysis of the crude ^{1}H NMR (with 1 equiv. DCE standard) indicates 32% of the desired oxazoline **27a** with 15% of the corresponding γ aminated product **27b** and 10% of the di-iodinated imidate **27c**.

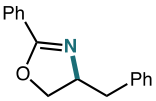

4-benzyl-2-phenyl-4,5-dihydrooxazole (27a)

R_f: 0.31 (20% Ethyl acetate/hexanes). ^{1}H NMR (400 MHz, $CDCl_3$) δ: 7.97 – 7.94 (m, 2H), 7.51 – 7.39 (m, 4H), 7.33 – 7.21 (m, 4H), 4.63 – 4.55 (m, 1H), 4.35 (dd, J = 9.1, 8.5 Hz, 1H), 4.15 (dd, J = 8.4, 7.4 Hz, 1H), 3.25 (dd, J = 13.7, 5.1 Hz, 1H), 2.74 (dd, J = 13.7, 8.9 Hz, 1H). ^{13}C NMR (100 MHz, $CDCl_3$) δ: 164.1, 138.2, 131.5, 129.4, 128.7, 128.5, 128.4, 127.9, 126.7, 72.0, 68.1, 42.0. HRMS (ESI-TOF) m/z: calc'd for $C_{16}H_{16}NO$ $[M+H]^+$ 238.1232, found 238.1232. IR (film) cm^{-1}: 3062, 2923, 2895, 2851, 1644, 1603.

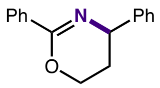

2,4-diphenyl-5,6-dihydro-4*H*-1,3-oxazine (27b)

R_f: 0.49 (20% Ethyl acetate/hexanes). ^{1}H NMR (400 MHz, $CDCl_3$) δ: 8.05 – 8.02 (m, 2H), 7.47 – 7.34 (m, 7H), 7.29 – 7.24 (m, 1H), 4.81 (dd, J = 7.9, 5.1 Hz, 1H), 4.44 – 4.33 (m, 2H), 2.37 – 2.30 (m, 1H), 1.97 – 1.88 (m, 1H). ^{13}C NMR (100 MHz, $CDCl_3$) δ: 155.8, 144.5, 134.1, 130.6, 128.5, 128.2, 127.4, 126.9, 126.8, 63.8, 55.1, 30.6. HRMS (ESI-TOF) m/z: calc'd for $C_{16}H_{16}NO$ $[M+H]^+$ 238.1232, found 238.1229. IR (film) cm^{-1}: 3060, 3029, 2924, 2851, 1651, 1599.

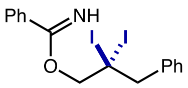

2,2-diiodo-3-phenylpropyl benzimidate (27c)

HRMS (ESI-TOF) m/z: calc'd for $C_{16}H_{16}I_2NO$ $[M+H]^+$ 491.9321, found 491.9324.

2. β vs γ competition: secondary vs tertiary C-H

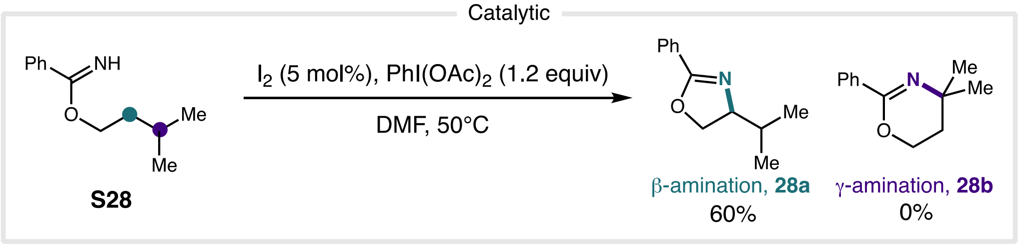

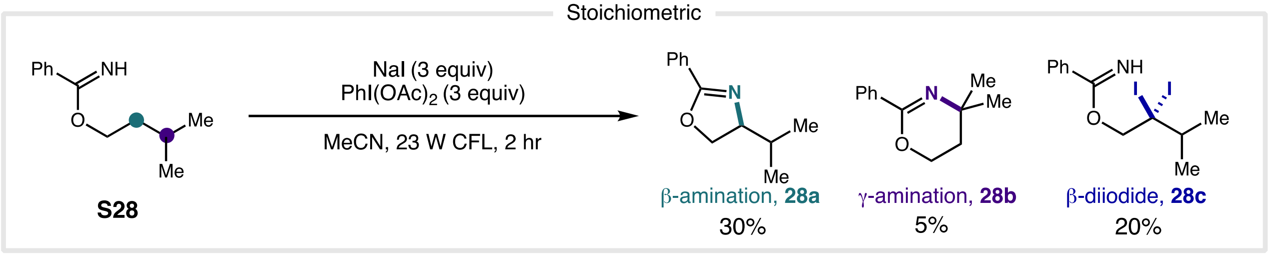

Catalytic: Imidate **S28** was subjected to **GP5**. Analysis of the crude ^{1}H NMR (with 1 equiv. DCE standard) indicates 60% of the desired oxazoline **28a** with none of the corresponding γ-aminated product **28b** (identified by analogy to a previously reported compound).[5]

Stoichiometric: Imidate **S28** was subjected to the stoichiometric reaction conditions.[1] Analysis of the crude ^{1}H NMR (with 1 equiv. DCE standard) indicates 30% of the desired oxazoline **28a** with 5% of the corresponding γ-aminated product **28b** which could not be cleanly isolated and 20% of the geminal di-iodinated imidate **28c**.

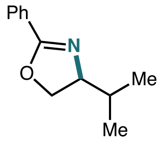

4-isopropyl-2-phenyl-4,5-dihydrooxazole (28a)

R_f: 0.44 (20% ethyl acetate/hexanes). ^{1}H NMR (400 MHz, $CDCl_3$) δ: 7.96 – 7.94 (m, 2H), 7.47 – 7.38 (m, 3H), 4.43 – 4.37 (m, 1H), 4.16 – 4.07 (m, 2H), 1.91 – 1.85 (m, 1H), 1.03 (d, J = 6.8 Hz, 3H), 0.93 (d, J = 6.8 Hz, 3H). ^{13}C NMR (100 MHz, $CDCl_3$) δ: 163.5, 131.3, 128.41, 128.39, 128.1, 72.8, 70.2, 33.0, 19.1, 18.2. HRMS (ESI-TOF) m/z: calc'd for $C_{12}H_{16}NO$ $[M+H]^+$ 190.1232, found 190.1242. IR (film) cm^{-1}: 2970, 2925, 2856, 1643.

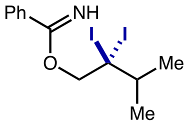

2,2-diiodo-3-methylbutyl benzimidate (28c)

R_f: 0.36 (20% Ethyl acetate/hexanes). ^{1}H NMR (400 MHz, $CDCl_3$) δ: 8.03 (bs, 1H), 7.82 (d, J = 7.0 Hz, 2H), 7.53 – 7.41 (m, 3H), 4.82 (bs, 2H), 1.75 – 1.69 (m, 1H), 1.10 (d, J = 6.4 Hz, 6H). HRMS (ESI-TOF) m/z: calc'd for $C_{12}H_{16}I_2NO$ $[M+H]^+$ 443.9321, found 443.9309.

c. Stereochemical Probe

To probe intermediates in the reaction, *cis* and *trans* stereochemical probes were synthesized to understand both the HAT and amination. The catalytic amination results were compared to those previously observed using the stoichiometric conditions.[1] First, subjection of the *cis* substrate led to material decomposition with none of the desired oxazoline presumably due to the inaccessible benzylic hydrogen. This result mirrors the previous observations. For the *trans* diastereomer, a notable difference in diastereoselectivity was observed in the isolated oxazoline. Interestingly, in the catalytic system the alkyl iodide intermediate can be observed which was previously not present in the photolytic conditions.

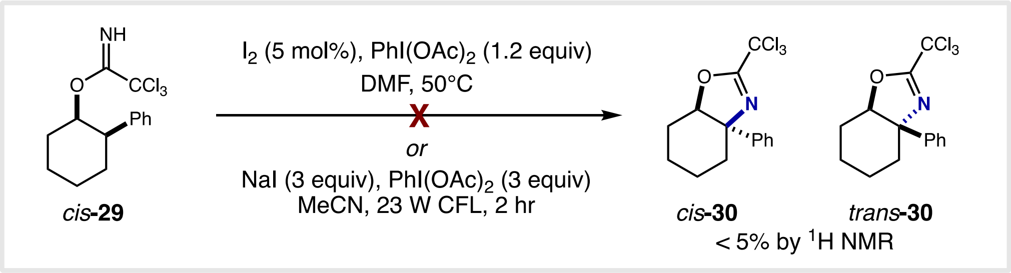

Trichloroacetimidate *cis*-**29** was subjected to **GP5** and the stoichiometric reaction conditions.[1] Analysis of the crude ^{1}H NMR (with 1 equiv. DCE standard) indicates < 5% of the desired oxazoline.

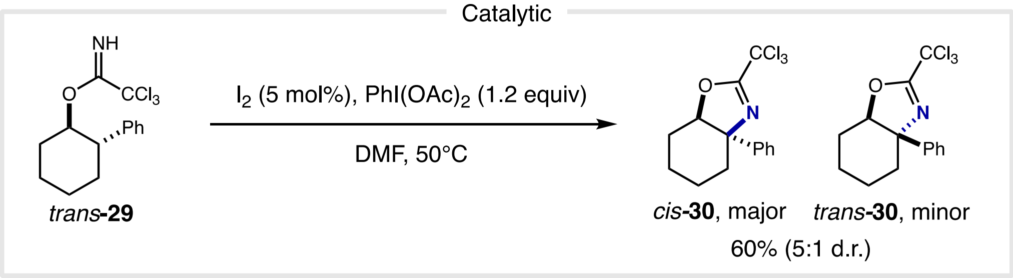

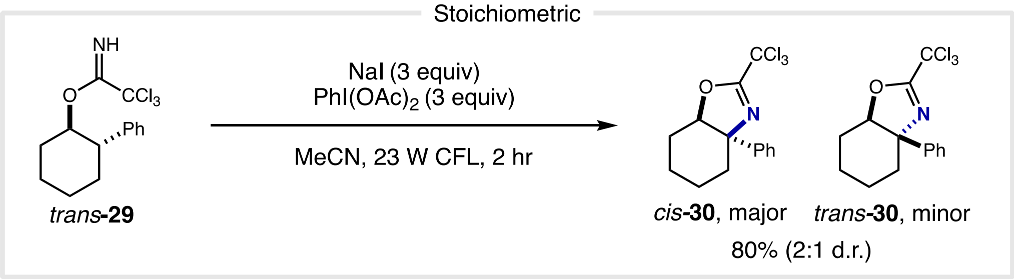

Catalytic: Trichloroacetimidate *trans*-**29** was subjected to **GP5**. Analysis of the crude ^{1}H NMR (with 1 equiv. DCE standard) indicates 60% (5:1 d.r.) of the desired oxazoline **30**.

Stoichiometric: Trichloroacetimidate *trans*-**29** was subjected to the stoichiometric reaction conditions.[1] Analysis of the crude ^{1}H NMR (with 1 equiv. DCE standard) indicates 80% (2:1 d.r.) of the desired oxazoline **30**.

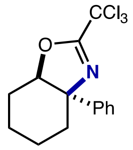

***cis*-2-(trichloromethyl)-3a,4,5,6,7,7a-hexahydrobenzo[*d*]oxazole (*cis*-30)**
R_f: 0.55 (10% Ethyl acetate/hexanes). ^1H NMR (400 MHz, $CDCl_3$) δ: 7.43 – 7.36 (m, 4H), 7.31 – 7.27 (m, 1H), 5.02 (t, J = 3.5 Hz, 1H), 2.23 – 2.17 (m, 1H), 2.11 – 2.04 (m, 1H), 1.99 – 1.90 (m, 2H), 1.75 – 1.61 (m, 4H). ^{13}C NMR (100 MHz, $CDCl_3$) δ: 161.6, 145.6, 128.8, 127.5, 125.4, 88.8, 87.3, 74.3, 34.0, 25.3, 17.8, 16.1.

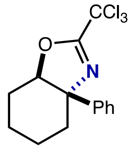

***trans*-2-(trichloromethyl)-3a,4,5,6,7,7a-hexahydrobenzo[*d*]oxazole (*trans*-30)**
R_f: 0.30 (10% Ethyl acetate/hexanes). ^1H NMR (400 MHz, $CDCl_3$) δ: 7.62 – 7.60 (m, 2H), 7.38 – 7.24 (m, 3H), 4.56 – 4.51 (m, 1H), 3.05 – 3.02 (m, 1H), 2.41 – 2.35 (m, 2H), 1.94 – 1.83 (m, 2H), 1.75 – 1.68 (m, 1H), 1.58 – 1.46 (m, 1H), 1.40 – 1.29 (m, 1H).

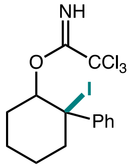

2-iodo-2-phenylcyclohexyl 2,2,2-trichloroacetimidate (31)
^1H NMR (400 MHz, $CDCl_3$) δ: 5.66 (bs) (diagnostic peak)
HRMS (ESI-TOF) m/z: calc'd for $C_{17}H_{22}Cl_3N_2O_2$ [M–I+DMF]$^+$ 391.0747, found 391.0742.

d. Hammett Plot

To determine the stabilization effect of substituents in the transition state of the 1,5-hydrogen atom transfer, a Hammett Plot analysis was conducted. Each data point in **Figure S3** is represented as an average of two trials. To identify the pseudo-first order regime, the early reaction time points from **Figure S1** were re-examined. Based on the data depicted in **Figure S2**, a 5-minute time point was selected for Hammett analysis.

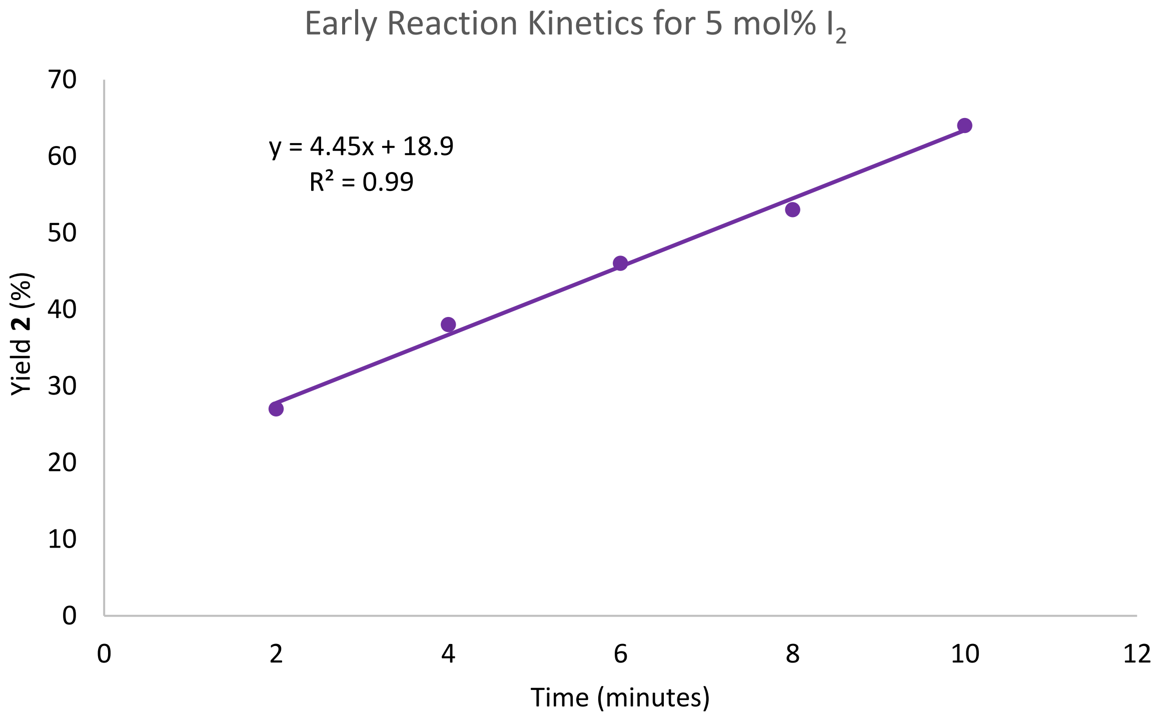

Figure S2: Expanded kinetic profile for the first ten minutes of a reaction with 5 mol% I_2 determined by ^{1}H NMR.

Catalytic Amination Procedure:

To a 2-dram vial equipped with PTFE septa cap and magnetic stir bar was added imidate (0.4 mmol, 1 equiv.) and PhI(OAc)$_2$ (154.6 mg, 0.48 mmol, 1.2 equiv.). This vial was evacuated and backfilled with N_2 (3x). A degassed stock solution of I_2 in dry DMF (2 mL, 0.01 M, 0.05 equiv.) was added to the vial under N_2. The reaction was heated to 50 °C (by placing vial in an aluminum heating block). After 5 minutes, the reaction was quenched with 10% aqueous $Na_2S_2O_3$ (2 mL) and then extracted with Et_2O (4 x 4 mL). The crude reaction was concentrated and analyzed by ^{1}H NMR using an internal standard, dichloroethane (31.6 µL, 1 equiv.).

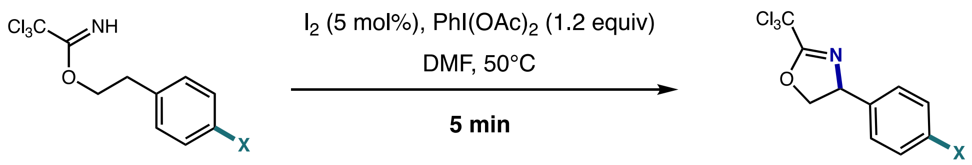

X	σ_p	Trial 1 Oxazoline (%)	Trial 2 Oxazoline (%)	Average (%)	R = yield X/ yield H	log(R)
OMe	-0.27	70	68	69	69/37	**0.271**
Me	-0.17	61	55	58	58/37	**0.195**
H	0	39	35	37	37/37	**0**
CF_3	0.54	20	25	22.5	22.5/37	**-0.216**
NO_2	0.78	17	21	18.5	18.5/37	**-0.301**

Table S2: Crude percent yield of substituted oxazolines determined by ^{1}H NMR.

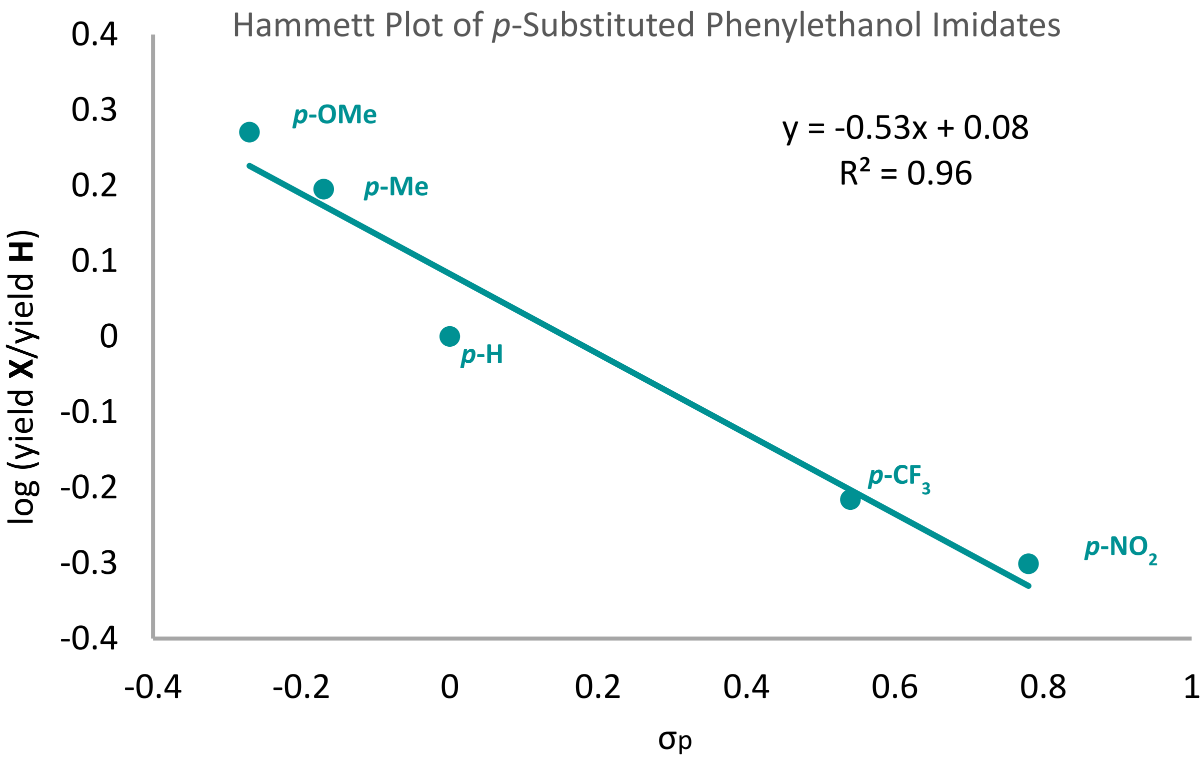

Figure S3: Hammett Plot of substituted phenylethanol derivatives.

e. **Intermediate Probe**

To probe the mechanism of this reaction, possible intermediates were synthesized. In the event that N-OAc, instead of N–I benzimidate, is formed via AcO–I, *N*-acetoxybenzimidate **S35** was synthesized. The oxime imidate **S35** was subjected to our catalytic amination condition, which resulted in full recovery of **S35**, indicating this is unlikely to be an intermediate. Additionally, **S35** is stable in refluxing DMF for 2 hrs.

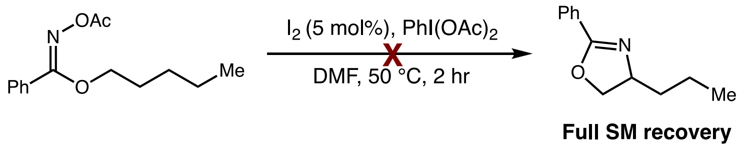

Oxime Imidate S35 was subjected to **GP5**. Analysis of the crude ^{1}H NMR (with 1 equiv. DCE standard) indicates no oxazoline and full recovery of oxime imidate starting material.

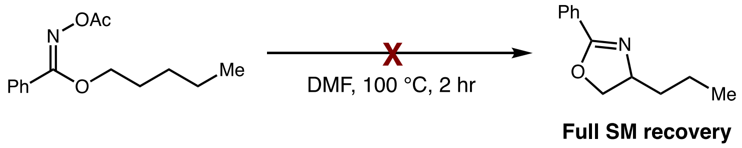

Oxime Imidate S35 was heated in DMF at 100 °C for 2 hrs. Analysis of the crude ^{1}H NMR (with 1 equiv. DCE standard) indicates no oxazoline and full recovery of oxime imidate starting material.

References:

1. Wappes, E. A.; Nakafuku, K. M.; Nagib, D. A. *J. Am. Chem. Soc.* **2017**, *139*, 10204.
2. Mou, X.-Q.; Chen, X.-Y.; Chen, G.; He, G. *Chem. Commun*. **2018**, *54*, 515.
3. Smith, B. R.; Eastman, C. M.; Njardarson, J. T. *J. Med. Chem.* **2014**, *57*, 9764.
4. Vitaku, E.; Smith, D. T.; Njardarson, J. T. *J. Med. Chem*. **2014**, *57*, 10257.
5. Ie, Y.; Chatani, N.; Ogo, T.; Marshall, D. R.; Fukuyama, T.; Kaiuchi, F.; Murai, S. *J. Org. Chem.* **2000**, *65*, 1475.
